# Parkinson’s disease in China: a forty-year growing track of bedside work

**DOI:** 10.1186/s40035-019-0162-z

**Published:** 2019-07-31

**Authors:** Gen Li, Jianfang Ma, Shishuang Cui, Yixi He, Qin Xiao, Jun Liu, Shengdi Chen

**Affiliations:** 1 Department of Neurology & Collaborative Innovation Center for Brain Science, Ruijin Hospital Affiliated to Shanghai Jiaotong University School of Medicine, Shanghai, China; 2 Co-innovation Center of Neuroregeneration, Nantong University, Jiangsu Province, China

**Keywords:** Parkinson’s disease, Tertiary network, Clinical research, Diagnosis

## Abstract

The number and health burden of Parkinson’s disease increase rapidly in China. It is estimated that China will have nearly half of the Parkinson’s disease population in the world in 2030. In this review, we present an overview of epidemiology and health economics status of Parkinson’s disease across China and discuss the risk factors of Parkinson’s disease and related complications. From the view of clinical research, we also discuss the current status of clinical trials, diagnostic biomarkers, treatment of Parkinson’s disease, tertiary network and post-occupation education in Chinese Parkinson’s disease clinics.

**Electronic supplementary material:**

The online version of this article (10.1186/s40035-019-0162-z) contains supplementary material, which is available to authorized users.

## Background

China, a multiethnic developing country with the largest population of the world, is stepping into an aging era [1]. It is estimated that nearly 23.9–26.9% of the population will be over 65 years old in 2050, due to a surge of aging population [2]. As the result, the population of neurodegenerative disease will increase as along and probably bring a huge burden to Chinese economics and healthcare system [3]. It is estimated that by 2030, Chinese Parkinson’s disease (PD) patients will increase to 4.94 million, accounting for a half of the worldwide PD patients [4].

**Fig. 1:**
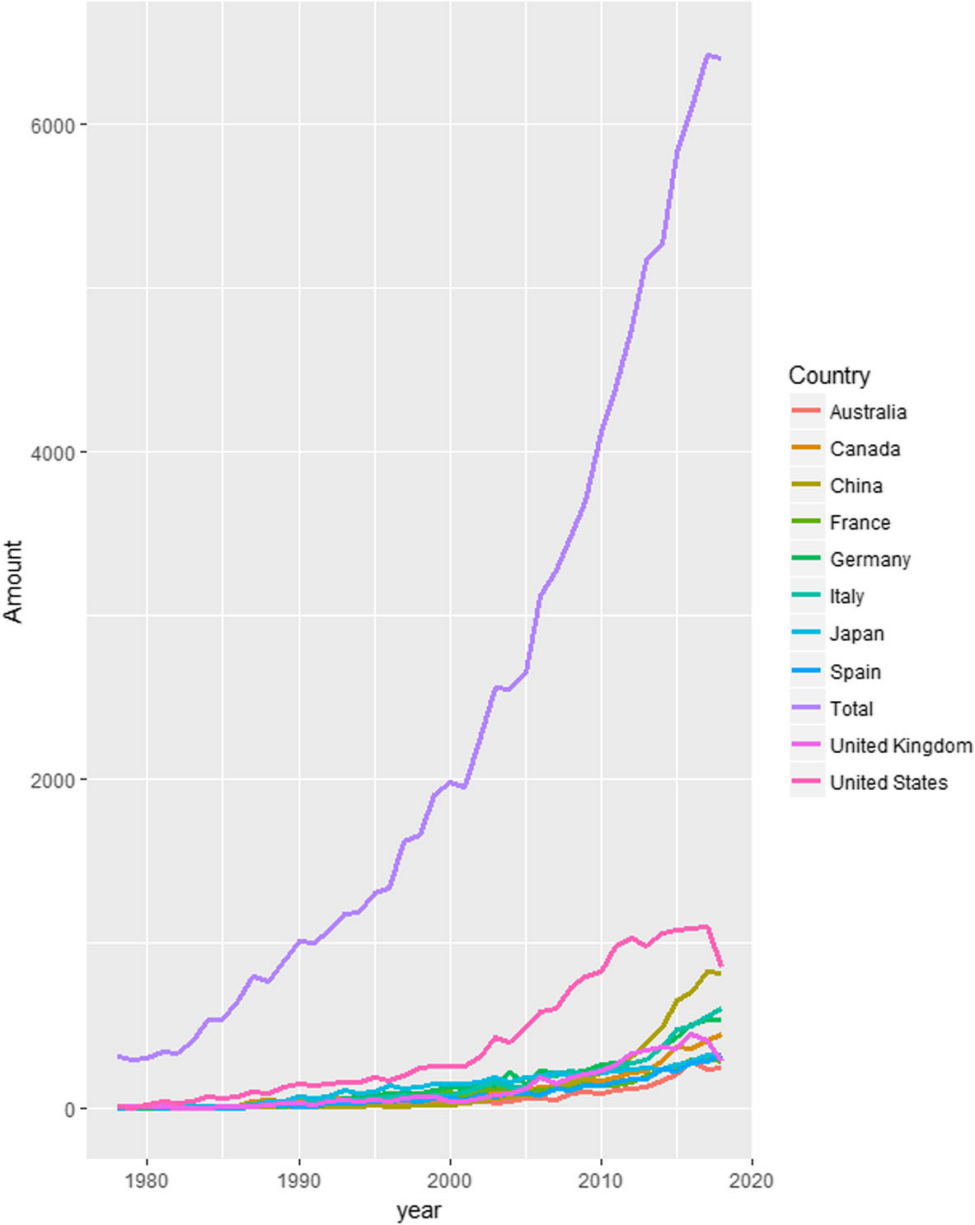
Publications related with Parkinson’s disease. Publications related to Parkinson’s disease since January 1, 1978 to November 1, 2018 listed on PubMed with the medical subject heading “Parkinson’s disease” and country in “all fields”

Since 1978, the year of “reform and opening”, economy has boomed in China. The researches in PD have also developed rapidly both in clinical and basic science, with the number of publications increasing from zero to the second largest country in the world. (Fig. 1) Chinese government has paid more and more attention to PD. For example, severe PD insurance has been covered by major disease insurance from Chinese government insurance system since 2007 [5]. Thanks to this policy, the economic burden of families with PD patients has been relieved significantly. For mild or moderate PD, diagnosis and treatment processes has been standardized in PD specialist training and costs of PD patients has been reduced since 2016 [6–8].

In this review, we discuss several facts of PD in China, including prevalence, incidence, mortality and risk factors, and health economics. We also discuss the status of clinical PD research in the recent 40 years, aiming to improve the future work of PD in China.

## Epidemiological study

### Prevalence, incidence, mortality

**Fig. 2:**
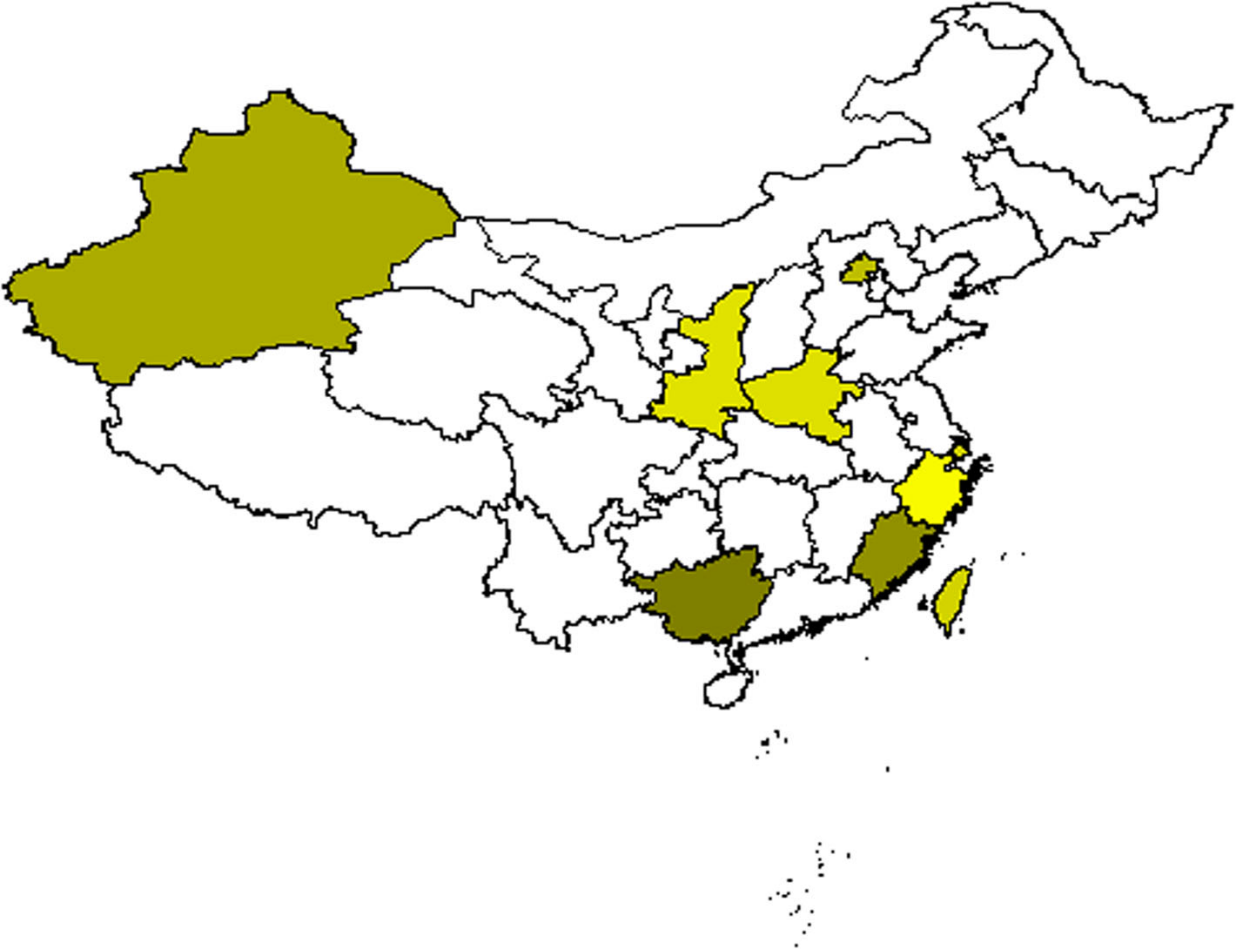
Statistic map of prevalence in China. Statistic map of prevalence in China. Darker color indicated higher prevalence of PD

Many PD prevalence studies have been performed in China, covering 9 provinces [9–27] (Fig. 2, Additional file 1: Table S1). The average prevalence of PD in China was about 3.8756‰ (≥50 years) in Han population. The prevalence was 1.234% in Uygur ethnicity [18, 22, 28], 1.208% in Kazak, and 1.224% in Hui ethnicity respectively [22]. In veterans over 60 years old, prevalence of PD increased to a much higher figure of 2.237%, which was much higher than 4.048‰ (≥60 years) [29–31]. However, only one incidence study was performed in China, which is in the Ilan county of Taiwan. In this study, the incidence was 10.4 per 100, 000, 11.1 per 100,000 in men and 9.8 per 100,000 in women [12].

PD mortality study in China is rare. In Ilan county of Taiwan, 5-year cumulative survival rate was 78.85%. [12] In a Shanghai PD cohort, the 5-year and 10-year standardized mortality ratio (SMR) was 0.62 and 0.87 respectively [32, 33]. In Hong Kong, the 10-year SMR was 1.1 [34]. In addition, it was found that older age at onset and postural instability gait disorder (PIGD) type had a negative impact of survival of Chinese PD patients [33, 34]. It is a remarkable fact that most of these researches are in Han ethnicity which accounts for 91.60% of population in mainland China (6th Nationwide census, National Bureau of Statistics) [35]. The rest 55 minor ethnicities are scattered in different areas and not covered in current epidemiological studies.

Several prevalence studies focused on motor and non-motor complications of PD. A multi-center survey of 28 movement disorders clinics in tertiary hospitals investigated PD patients on levodopa treatment and reported that the prevalence of wearing-off phenomenon and dyskinesia was 46.5 and 10.3% respectively [36]. Another single center study reported that the prevalence of wearing-off phenomenon were only 29.0% (≤ 1 year), 33.5% (1–2.5 years), 50.2% (2.5–5 years), 60.3% (5–10 years) and 68.3% (≥ 10 years), respectively. For dyskinesia, disease duration related prevalence was 3.1, 5.5, 13.1, 12.8 and 19.3% respectively. Overall, 70.8% PD patients suffered non-motor symptoms [37]. The prevalence of depression and anxiety in PD patients were 11.17–21.7% and 25.81–33.3% respectively [38, 39]. For visual hallucinations, the prevalence was about 19.4% [40]. In patients with visual hallucinations, 26.4% of them experienced minor hallucinations, and the rest of them had complex visual hallucinations. The presence of visual hallucinations was associated with longer disease duration, dopaminergic agonist usage, poor sleep quality and cognitive dysfunction [40]. For cognitive dysfunction, 21.4% of PD patients met the criteria for PD dementia, and 22.8% met the criteria for PD--mild cognitive impairment [41]. About 54.10% of PD patients suffered from constipation, and 47.59% of them reported constipation before onset of the motor symptoms [42]. A cross-sectional survey in Chinese PD patients in Hoehn-Yahr stage I-III revealed that 29.9% patients suffered from PD-related pain, associated with dyskinesias, and/or depression [43]. Screened by interview according to the criteria of international restless legs syndrome study group, the prevalence of restless legs syndrome in PD was 10.7% [44].

### Health economics, costs

In the year of 2004–2005, the overall mean annual cost of PD in Shanghai was 925 USD, and the direct medical care cost was 519 USD (56.1%) [45]. In the year of 2015, the overall mean annual cost of PD increased to 3,225.94 USD, and the direct medical care cost was 1,737.93 USD (53.9%) in Tianjin [46]. However, The percentage of overall mean annual cost of PD in GDP per capita was only increased slightly (2004: 16.21%; 2015: 17.46%). Sourcing from China Entrepreneur Investment Club (CEIC, https://www.ceicdata.com/en), a database of macroeconomics, the gross domestic product (GDP) per capita in Shanghai in 2004 was 5,708.00 USD (47,244 RMB, 1 USD = 8.2768 RMB) and GDP per capita in Tianjin in 2015 was 18,472.32 USD (115,053 RMB, 1USD = 6.2284 RMB). Many anti-PD drugs have been covered in the government health insurance, and deep brain stimulation (DBS) has been covered partly in a few cities recently.

### Risk factors

Common risk factors have been found, such as rapid eye movement sleep behavior disorder (RBD), olfactory dysfunction, constipation, family history of PD and pesticide exposure [47]. In addition, regular tea drinking habit and smoking were protective factors of PD [48].

Several studies focused on risk factors for motor and non-motor complications of PD. Young age of onset, severe depressive symptoms, female, RBD, autonomic dysfunction and muscle cramps were risk factors of anxiety in PD patients [49]. Tumor history, currently single marital status, severe motor dysfunction, dyskinesia, poor sleep quality, anxiety, AA genotype of rs1545843 and AC genotype of rs78162420 were risk factors of depression in PD patients [38, 50]. Long duration of PD and levodopa treatment, high daily levodopa dosage and high scores in the 39-item PD Questionnaire were risk factors of dyskinesia and motor fluctuations [51]. Patient without psychiatric symptoms, younger age and more education were shown less cognitive impairment [41].

## Clinical research

The number of PD scientific publications is being a steady increased in China (Fig. 1), exceeding over other countries except for the United States. However, poor quality might come along with the growing amount of publications.

### Clinical trials

According to ClinicalTrials.gov, there are 110 studies in China registered on this website, 56 of them in mainland, 2 in Hong Kong and 52 studies in Taiwan. Due to historical reasons, the development of clinical trials are uneven among mainland China, Hong Kong, Macau, and Taiwan. The number of clinical trials that we took part in was much less than that in developed countries, such as United States, Canada, United Kingdom and Japan. ClinicalTrials.gov is not widely used in mainland China. With the socioeconomic and medical care developments in past 40 years, clinical trials in China have been paid more attention than before. More and more Chinese hospitals have been invited to participate high-quality international multicentral clinical trials (Table 1, Additional file 1: Table S2).

**Table 1:** Number of clinical trials and publications in progress for Parkinson’s disease

Country	Ongoing trials	Publications^a^	Publications in 2017
China	40	5471	823
The United States	290	16208	1105
Canada	41	4354	408
Japan	8	5142	318
The United Kingdom	32	4598	411

^a^Retrieved from PubMed (November 1, 2018) with “Parkinson’s disease” in “all fields” and country name

### Clinical research

#### Diagnostic biomarkers

Diagnostic biomarker study is an important part in clinical PD research. Genetic associations of single nucleotide polymorphisms (SNPs) in Chinese Han population were identified and replicated quickly. However, how these genetic risk loci contribute to the adult-onset PD pathogenesis remains largely unknown. In Table 2, odds ratios of confirmed genes were depicted (Table 2, Additional file 1: Data S3). Genetic techniques, such as Next-Generation Sequencing (NGS), genetic testing, which could pinpoint the culprit gene, have been put into use to assist the diagnosis of juvenile parkinsonism.

**Table 2:** Risk variants validated in Parkinson’s disease in Chinese population

Models	Polymorphisms
Allele	*A2M* rs669; *A2M* rs3832852; *BST1* rs4698412; *BST1* rs11931532; *CHCHD2* rs142444896; *CCDC62/HIP1R* rs12817488; *GAK/PARK17* rs1564282; *GBA* L444P; *GPNMB* rs156429; *LINGO1* rs11856808; *LRRK2* rs34594498; *LRRK2* rs34778348; *LRRK2* rs33949390; *LRRK2* rs7304279; *LRRK2* rs2046932; *NUCKS1* rs823114; *PARK16* rs823156; *PITX3* rs3758549; *SIPA1L2* rs10797576; *PARK16* rs947211; *RIT2* rs12456492; *NQO1* C609T; *SNCA* rs11931074; *SNCA* rs2736990; *SNCA* rs356165; *SNCA* rs356219; *SNCA* rs3822086;
Dominant	*A2M* rs669; *BST1* rs4698412; *CHCHD2* rs142444896; *CCDC62/HIP1R* rs12817488; *GAK/PARK17* rs1564282; *GBA* L444P; *GPNMB* rs156429; *LINGO1* rs11856808; *LRRK2* rs34594498; *LRRK2* rs34778348; *LRRK2* rs33949390; *PITX3* rs2281983; *PITX3* rs3758549; *PITX3* rs4919621; *SIPA1L2* rs10797576; *PARK16* rs947211; *RIT2* rs12456492; *SLC41A1* rs11240569; *SNCA* rs11931074; *SNCA* rs2736990; *SNCA* rs356165; *SNCA* rs356219; *SNCA* rs3822086;
Recessive	*BST1* rs4698412; *BST1* rs11931532; *CCDC62/HIP1R* rs12817488; *GAK/PARK17* rs1564282; *LRRK2* rs7304279; *LRRK2* rs2046932; *NUCKS1* rs823114; *PARK16* rs823156; *PARK16* rs947211; *SNCA* rs11931074; *SNCA* rs2301135; *SNCA* rs2736990; *SNCA* rs356165; *SNCA* rs356219; *SNCA* rs3822086;
Overdominant	*A2M* rs669; *CHCHD2* rs142444896; *GAK/PARK17* rs1564282; *GBA* L444P; *GPNMB* rs156429; *LINGO1* rs11856808; *LRRK2* rs34594498; *LRRK2* rs34778348; *LRRK2* rs33949390; *LRRK2* rs7304279; *LRRK2* rs2046932; *NUCKS1* rs823114; *PARK16* rs823156; *PITX3* rs2281983; *PITX3* rs3758549; *SIPA1L2* rs10797576; *VPS13C* rs2414739; *SLC41A1* rs11240569; *SNCA* rs11931074; *SNCA* rs356165;

The regulations of several non-coding RNAs, such as down-regulation of miR-15b, up-regulation of miR-24, have been validated as biomarkers of PD [52–57]. Besides non-coding RNAs, α-synuclein oligomer level in erythrocytes, P2Y6R mRNA level in peripheral blood mononuclear cells and several epigenetic changes (e.g. NPAS2 hypomethylation) were also confirmed as diagnostic biomarkers in Chinese population [58–60]. In other body fluids, potential biomarkers for making a diagnosis or monitoring disease progression were also found. In cerebrospinal fluid, exosomal miRNAs were found valuable for making a diagnosis of PD, such as miR-1, miR-153, miR-409-3p, miR-19b-3p, and miR-10a-5p or the combination of miR-153 and miR-409-3p [61]. Salivary DJ-1 was proven as a potential biomarker for monitoring disease progression [62].

Besides rapid eye movement sleep behavior disorder, constipation and hyposmia, clinical biomarkers are paid attention by Chinese neurologists. Radiological methods, such as ^99m^Tc-TRODAT-1 single photon emission computed tomography (SPECT) [63], ^18^F-fluorodeoxyglucose positron emission tomography imaging (^18^F-FDG-PET) [64], functional magnetic resonance imaging [65] and Iodine-123-meta-iodobenzylguanidine (^131^I-MIBG) [66] have been put into use. The combinations of several biomarkers in PD have been set up, such as the combination of ‘swallow-tail’ sign and putaminal hypointensity [67]. Transcranial sonography (TCS), a non-invasive method of diagnostic test for its high positive predictive value, has been used into assisting clinical diagnosis [68].

However, the validation, sensitivity and specificity of these biomarkers among different ethnicities and the combination of these biomarkers are warranted. Biomarkers in prodromal stage are needed, as well as a nationwide multi-center Chinese prodromal PD cohort.

#### Treatments

##### Drug therapy

Most drugs for PD are available except for apomorphine and Levodopa-carbidopa intestinal gel, inhalation form of L-dopa, droxydopa, pimavanserin, safinamide, extended release amantadine in mainland China. The Chinese PD and Movement Disorders Society (CMDS) published guidelines of PD and PDD treatment in 2006, and updated in 2009 and 2014 [69–71]. Treatment concepts from CMDS are different compared to other guidelines. Firstly, high-risk population screened by prodromal PD biomarkers is recommended to non-drug therapy first, aiming for possible delaying the disease progression. Secondly, combined therapy is highly recommended, such as drug and non-drug therapies (exercise, rehabilitation, surgery and psychotherapy etc.), aiming to improve motor symptoms and non-motor symptoms simultaneously and probably delay the progress of PD to some degree. Thirdly, as for drug therapy, compared to treatment concepts of “full and rapid dose” in western countries, combination of multiple drugs with minimal dosage to achieve optimal clinical effect is widely accepted, which might explain the reason why the prevalence of short-term and long-term complications is much lower than western countries, as well as the prevalence of dyskinesia (China, 6.2%; US, 27.8%) [72, 73]. This treatment regimen is also reported with less side-effect and better tolerance. For example, severity of motor and non-motor PD complications was milder than patients in western countries [72].

##### Surgical treatment

In 1950s, stereotactic surgeries such as posteroventral pallidotomy (PVP) and unilateral subthalamotomy were adopted in clinical treatment of PD. However, they were abandoned because of severe adverse effects since 1968, the year of levodopa introduction in China [74]. With the development of medical imaging and technique of micro-electronode records, and the complications of long-term dopaminergic medication, stereotactic surgery was back to the stage in 1980s [75]. Due to loose management of indications and contraindications of stereotactic surgery, especially the use of bilateral stereotactic surgery, new severe adverse effects emerged, which taught us a lesson of the importance for the control of indications and contraindications. DBS was put into use since the beginning of twenty-first century. Expert consensus for DBS and programming brought up by CMDS has been established and put into use to regulate the use of DBS from pre-surgery to post-surgery. In several hospitals, multidisciplinary collaboration has been performed for DBS treatment, such as department of neurology, department of functional neurosurgery and department of rehabilitation. However, high cost of DBS implantation and unable to improve all symptoms restrict the wide use of DBS. Now, the focused ultrasound therapy has just been introduced for PD.

##### Traditional Chinese medicine

In traditional Chinese medicine (TCM), PD can be categorized into three syndromes based on the main symptoms. Most tremorous PD belongs to “tremor syndrome”. [76]. Treatment to these syndromes are different in TCM. For motor fluctuations, Yanggan Xifeng recipe could provide the synergistic effect along with levodopa and reduce the side effect of levodopa [77]. Zishenpingchan granules could alleviate motor symptoms and side effects of dopaminergic agents [78]. The striatal TH activity and expression of TH mRNA were significantly higher in PD rat models who received levodopa accompanied with Bushen Yanggan recipe than PD rat models who just received levodopa treatment [79]. Besides recipes, acupuncture and massage are two complementary ways to alleviate PD symptoms. The seven acupoints of the cranial base (SACB) can alleviate rigidity [80]. Acupoint massage can alleviate constipation and sleep dysfunction [81, 82].

##### Rehabilitation

Rehabilitation is considered as a complementary therapy in PD. It can improve the functional abilities and decrease complications. Physical therapy, occupational therapy and speech therapy are major parts of rehabilitation. Traditional Chinese activities, such as Qigong, Tai Chi and Baduanjin, have been drawn attention because of their benefits, probably due to rehabilitation effect. Tai Chi could improve motor function, balance and gait in PD [83]. Blood HIP2 mRNA, a biomarker for PD, could be reversed after Tai Chi training [84]. Baduanjin improved the gait performance, functional mobility, sleep quality and prevented falls [85, 86]. Rhythmic auditory stimulation with visual stimuli to PD were effective to Chinese population [87]. There are several classes of Tai Chi or dancing for PD in China. Recently, several multi-center cohorts on traditional Chinese rehabilitation were established in different cities.

## Clinical work

### Clinical diagnosis

A survey conducted in Shanghai revealed that the median time from the onset of motor symptom to the first clinical visit was 1.0 month (0–51.5 months) [88]. The median time from the onset of motor symptom to the clinical diagnosis was 10.0 months (0.5–118.0 months). The median time from the onset of motor symptom to the treatment of dopaminergic drugs was 12.00 months (0.5–146.0 months). The misdiagnosis rate was 23.53%. The onset motor symptom was tremor, rigidity, bradykinesia and gait abnormalities sequentially. PD patients with the onset of rigidity or gait abnormalities were more easily misdiagnosed (misdiagnosis rate: 34.78, 35.71%, respectively). As for the retired elderly, the time from the onset of motor symptom to the clinical diagnosis was shorter compared to people who were at work. Compared to UK, the median time from the onset of motor symptom to clinical visit was shorter (UK: 11 months) [89]. Different health management and healthcare system may contribute to this difference.

A study aiming to compare UK-Criteria versus MDS-PD Criteria in the clinical diagnosis of Parkinson disease in a single center was conducted [90]. In this study, 66 subjects (66%) were diagnosed as PD according to the UK-Criteria and 81 subjects (81%) according the MDS-PD Criteria from 100 subjects with parkinsonism, showing higher sensitivity of MDS-PD Criteria. The differences between the diagnostic results of these two criteria were statistically significant tested by paired Chi-square test. Besides, it was found that levodopa-induced dyskinesia had a good positive predictive value, while early bulbar impairment and inspiratory dysfunction had a negative predictive value. However, this study did not evaluate the diagnostic value of olfactory test, TCS, MIBG and DAT-PET due to high missing data of these tests.

### Patient education and education of PD in general

Like other chronic diseases such as diabetes, hypertension and cerebral vascular disease, it is important to educate physicians and patients for PD management. Health education is an effective way for local physician and patients to acquire knowledge of PD, which could improve the early diagnosis and management of PD [91]. Tan et al. performed a study on awareness status of PD among elderly veterans in Beijing. In this study, 80.3% subjects heard the name of PD. As for the prevention knowledge, the awareness rate was 62.1%. In those veterans with prevention knowledge of PD, 52.7% of them learned from media, 72.1% of them learned from word of mouth, and only 11.8% learned from health care professionals [92]. Thanks to communication tools through smartphone, such as Weibo and WeChat, it is much easier to perform patient education. Personal health education and health education to caregivers are effective and need more attention [93, 94].

Palliative care has a late start in China. There is no currently available palliative care for PD in China. Caregivers often regard palliative care as hospice care [95]. Besides, there are limited amount of hospitals providing palliative care. Effective palliative care for PD patients can reduce burden of caregivers and medicals. Palliative care methods, such as support to caregivers and multidisciplinary approach, are warranted [95]. To increase the awareness of palliative care for PD, it is warranted to encourage the medical education of palliative care to patients and caregivers.

### Tertiary network of China

The health system of China, i.e. the tertiary network, is different from the health system of western countries. The primary hospitals, which are mainly located in communities, are for prevention, diagnosis and treatment of common illness. The secondary hospitals serve for several communities and are in charge of teaching and researching. The tertiary hospitals are for the diagnosis and treatment of difficult cases and are in charge of teaching and researching. As patients are self-referred in Chinese health system and there is an inequivalent development between the gross economic growth and the service of hospitals, patients prefer to choose tertiary hospitals for the first clinical visit rather than primary or secondary hospitals. Dual referral system of this network is not well developed. Experienced neurologists are overworked in tertiary hospitals [96]. In contrast with that, doctors in primary hospitals have less chance to deal with the first clinical visit of patients, and neither do in secondary hospitals. Compared to specialists, general practitioners usually see PD patients earlier. They should recognize the symptoms of PD and refer them to PD specialists for confirming diagnosis. Actually, health education and follow-up to PD patients could be easily done in communities [97]. It is warranted to train more experienced family doctors in primary and secondary hospitals, and follow the dual referral system in tertiary network. Health document of PD patient information needs to be shared in all tertiary networks.

### PD clinics

Clinics to specific diseases have been set up to help the patients see specialists. The first PD clinics, started in seven hospitals in China, including Beijing Hospital, Ruijin Hospital and other 5 hospitals in 1978. Later, the Chinese Movement Disorder Study Group was established. Specialists in PD clinics are experienced doctors in the PD diagnosis and treatment. Besides, it is much easier to perform health education to PD patients and caregivers in the PD clinics. Clinics of neuromodulation and neurorehabilitation were set up recently. In April 2007, club of PD patients were organized by Ruijin Hospital affiliated to Shanghai Jiaotong University School of Medicine, for the purpose of better education and management [98].

Wearable devices improved the quality of PD patients’ life and helped PD diagnosis to some extent. Researches on wearable devices in China have just started. In PD clinics, more and more wearable devices could be applied. PD-Monitor applied with evolutionary algorithm could differentiate early stage PD patients with normality [99]. Spoons to help people with tremor eat food have been on the market. Visual aiding devices have been applied into use to prevent the freezing of gait in PD.

### Post-occupation education

#### Team of PD and movement disorders, Chinese Society of Neurology

The Chinese PD and Movement Disorders Society was established in 2002, which is affiliated to Chinese Medical Association (CMA). The diagnostic criteria and therapeutic guidelines of PD have been published by this society, including Chinese diagnostic criteria of PD [100], guideline of PD treatment in China [69, 70], diagnosis and treatment of PD dementia [101], Chinese consensus of diagnosis and treatment of vascular parkinsonism [102], Chinese diagnostic and treatment guideline of PD depression, anxiety and psychosis [103], Chinese expert consensus on clinical database construction of PD and movement disorders [104], Chinese expert consensus on DBS and programming of DBS of PD [105, 106], and Chinese expert consensus document on the therapeutic uses of botulinum toxin [107]. These guidelines are useful to guide clinical diagnosis and treatment of PD in China.

#### Post-occupation educational class and online training

Besides adaptive reading literatures, guidelines and expert consensus, many meetings and post-occupation educational classes were organized every year to update the knowledge of PD. There are 3 meetings related to PD held by national association, i.e. annual conference of the CMDS, annual conference of Chinese Neurology Society, annual conference of Chinese Neurologist Association [108, 109]. Senior movement disorder specialists share the update of PD and experience of PD on these conferences.

There are only about 150 board-certified movement disorder specialists in CMDS currently among nearly 100,000 neurologists in China. Thanks to the development of Internet, online learning courses have been a common way for practitioners who do not have enough grant for attending the meetings. The biggest medical forum, Dingxiangyuan, provides a lot of open online courses for PD. Mobile learning via applications such as WeChat has been introduced recently and soon becomes popular among doctors.

## Conclusions and recommendations

Since 1978, the development of clinical research and bedside work of PD in China is fascinating. The amount of publications increases from zero to the second place in the world. The bedside work develops from personal experience to standardized diagnosis and treatment. By 2030, there will be a half of worldwide PD patients in China. There are more and more doctors paying attention to PD. Clinical researches provide important data for amending health policies of PD, such as cost for DBS could be reimbursed in health insurance for PD. However, several problems remain to be solved. Huge burden of tertiary hospital doctors, lack of experienced doctors in primary and secondary hospitals in PD, and the lack of dual referral system in tertiary network are the current major issues. Lack of standardized PD database is another issue. Chinese expert consensus on clinical database construction of PD and movement disorders brought up by CMDS has been published recently to standardize the construction on clinical database [104]. Due to uneven disease education level of PD, visiting rate and treatment rate are low and imbalanced among different areas. High quality clinical research articles are few currently. Noted, most articles are focused in Han ethnicity. The 55 minor ethnicities are seldomly covered in most researches. There are slightly different beliefs and lifestyles of these minor ethnicities. But few researches focused on minor ethnicities. More researches focused on PD in minor ethnicities are warranted.

## Additional file

Additional file 1: **Table S1.** Epidemiology of PD in China. **Table S2.** Status of Clinical Trials. **Data S3.** Meta analysis of genetic variants for Parkinson’s disease in Chinese population. (DOCX 38298 kb)

## Data Availability

The datasets used and analyzed during the current study are available from the corresponding author on reasonable request.

## Abbreviations

CEIC: China entrepreneur Investment Club
CMA: Chinese Medical Association
CMDS: Chinese Parkinson’s disease and Movement Disorders Society
DBS: Deep brain stimulation
FDG: Fluorodeoxyglucose
GDP: Gross domestic product
MDS: Movement disorders society
MIBG: Meta-iodobenzylguanidine
NGS: Next-generation sequencing
NHC: National Health Commission
PD: Parkinson’s disease
PET: Positron emission tomography
PIGD: Postural instability gait disorder
PVP: Posteroventral pallidotomy
RBD: Rapid eye movement sleep behavior disorder
SACB: Seven acupoints of the cranial base
SMR: Standardized mortality ratio
SNP: Single nucleotide polymorphisms
SPECT: Single photon emission computed tomography
TCM: Traditional Chinese medicine
TCS: Transcranial sonography
UK: United Kingdom
