# Supplementary material for: Parkinson’s disease in China: a forty-year growing track of bedside work
Source: Transl Neurodegener. 2019 Jul 31;8:22. doi: 10.1186/s40035-019-0162-z (PMC6668186; doi:10.1186/s40035-019-0162-z)
Supplement: Supplementary file 1 — Table S1. Epidemiology of PD in China. Table S2. Status of Clinical Trials. Data S3. Meta analysis of genetic variants for Parkinson’s disease in Chinese population. (DOCX 38298 kb) [file 40035_2019_162_MOESM1_ESM.docx]

**Additional file 1:Table S1**: Epidemiology of PD in China. Several diagnostic criteria were used in prevalence survey: ≥ 2 of the 4 cardinal signs, Brainbank diagnosis of Parkinson’s disease, Parkinson’s disease and movement disorders group of Chinese Society of Neurology, Huashan Hospital of Fudan University, movement disorders society (MDS) and diagnosed by neurologists (detailed diagnostic criteria were not given). Incidence and mortality studies were rare.

| Location | Time of survey | Prevalence | Reference |
| --- | --- | --- | --- |
| Diagnostic criteria: ≥ 2 of the 4 cardinal signs: resting tremor, bradykinesia, rigidity and postural-reflex impairment; with detailed exclusion criteria^1-3^ | | | |
| Kin-Hu, Kinmen, Taiwan | August, 1992 | 50 - 59 years: Men: 0/55, Women: 0/66  60 - 69 years: Men: 1/68, Women: 0/61  70 - 79 years: Men: 1/52, Women: 1/62  ≥ 80 years: Men: 3/51, Women: 0/67 | Wang SJ, Fuh JL, Liu CY, et al. Parkinson's disease in Kin-Hu, Kinmen: a community survey by neurologists. Neuroepidemiology 1994; 13(1-2): 69-74. |
| Ilan County, Taiwan | January 1, 1993 - May 31, 1995 | 40 - 49 years: Men: 1/1277, Women: 0/1372  50 - 59 years: Men: 4/1584, Women: 0/1680  60 - 69 years: Men: 3/1334, Women: 11/1227  70 - 79 years: Men: 4/620, Women: 6/600  ≥ 80 years: Men: 3/149, Women: 5/215 | ^Chen RC, Chang SF, Su CL, et al. Prevalence, incidence, and mortality of PD: a door-to-door survey in Ilan county, Taiwan. Neurology 2001; 57(9): 1679-86.^ |
| Kinmen, Taiwan | August 1, 1993 - September 17, 1994 | 50 - 59 years: Men: 3/824, Women: 1/639  60 - 69 years: Men: 4/675, Women: 3/634  70 - 79 years: Men: 2/311, Women: 2/397  ≥ 80 years: Men: 3/156, Women: 5/279 | Wang SJ, Fuh JL, Teng EL, et al. A door-to-door survey of Parkinson's disease in a Chinese population in Kinmen. Archives of neurology 1996; 53(1): 66-71. |
| Beijing | October 1996 - October 1997 | 55 – 64 years: Men: 4/1207, Women: 4/1554  65 – 74 years: Men: 12/1006, Women: 12/1068  75 – 84 years: Men: 12/319, Women: 15/445  85 – 94 years: Men: 2/51, Women: 3/84  ≥95 years: Men: 0/1, Women: 0/8 | Zhang ZX, Anderson DW, Huang JB, et al. Prevalence of Parkinson's disease and related disorders in the elderly population of greater Beijing, China. Movement disorders : official journal of the Movement Disorder Society 2003; 18(7): 764-72. |
| Beijing | 1997-98 | 55 – 64 years: Men: 6/1825, Women: 7/2378  65 – 74 years: Men: 19/1587, Women: 15/1599  75 – 84 years: Men: 18/497, Women: 17/620  ≥85 years: Men: 2/65, Women: 3/123 | Zhang ZX, Roman GC, Hong Z, et al. Parkinson's disease in China: prevalence in Beijing, Xian, and Shanghai. Lancet (London, England) 2005; 365(9459): 595-7. |
| Xian | 1997-98 | 55 – 64 years: Men: 0/845, Women: 0/1377  65 – 74 years: Men: 9/789, Women: 9/991  75 – 84 years: Men: 11/375, Women: 4/394  ≥85 years: Men: 2/31, Women: 2/48 | Zhang ZX, Roman GC, Hong Z, et al. Parkinson's disease in China: prevalence in Beijing, Xian, and Shanghai. Lancet (London, England) 2005; 365(9459): 595-7. |
| Shanghai | 1997-98 | 55 – 64 years: Men: 8/2509, Women: 0/2222  65 – 74 years: Men: 21/3126, Women: 18/1780  75 – 84 years: Men: 29/1326, Women: 15/769  ≥85 years: Men: 5/222, Women: 4/79 | Zhang ZX, Roman GC, Hong Z, et al. Parkinson's disease in China: prevalence in Beijing, Xian, and Shanghai. Lancet (London, England) 2005; 365(9459): 595-7. |
| a housing estate close to the teaching hospital of the Faculty of Medicine of the University, Hong Kong | September to  October, 2000 | ≥55 years: 2/415 | Woo J, Lau E, Ziea E, Chan DK. Prevalence of Parkinson's disease in a Chinese population. Acta neurologica Scandinavica 2004; 109(3): 228-31. |
| Keelung, Taiwan | January 1, 2001 to December 31, 2001 | 40 - 49 years: Men: 0/1141, Women: 0/2376  50 - 59 years: Men: 1/793, Women: 2/1695  60 - 69 years: Men: 7/1062, Women: 12/1704  70 - 79 years: Men: 25/1093, Women: 14/1029  ≥ 80 years: Men: 11/260, Women: 8/179 | Chen CC, Chen TF, Hwang YC, et al. Different prevalence rates of Parkinson's disease in urban and rural areas: a population-based study in Taiwan. Neuroepidemiology 2009; 33(4): 350-7. |
| Shijiazhuang, Hebei | August 2004 – August 2006 | Source: elderly veterans  65 – 69 years: Men: 2/145, Women: 3/459  70 – 79 years: Men: 31/1280, Women: 7/546  ≥80 years: Men: 6/205, Women: 1/39 | Baocheng Yu, Jianzheng He, Peng Cheng, et al. Prevalence of Parkinson Disease in elderly veterans. China Healthcare Innovation 2007; (19): 102-3. (于宝成, 何建政, 程鹏, 方海珍, 魏冉, 王哲. 军队干休所老年人帕金森病患病率调查. 中国医疗前沿（上半月） 2007; (19): 102-3.) |
| Yulin & Yanan, Shanxi Province | January – March 2013 | 50 – 59 years: Men: 2/2658, Women: 2/2787  65 – 69 years: Men: 5/1826, Women: 6/2012  70 – 79 years: Men: 3/875, Women: 4/1110  ≥80 years: Men: 1/251, Women: 1/264 | Xiaorong Gao, Na Li, Lifang Wu et al. Prevalence Survey of Parkinson’s disease in Northern Shanxi. Chinese Journal of Practical Nervous Diseases 2016; (3): 82-3. (高晓嵘, 李娜, 武丽芳, 薛艺东. 陕北地区帕金森病现状调查. 中国实用神经疾病杂志 2016; (3): 82-3.) |
| Prevalence: Diagnostic criteria: Brain Bank diagnosis of Parkinson’s disease | | | |
| Linxian County, Henan Province | November 8, 1999 to April 30, 2000 | 50 - 59 years: Men: 4/2262, Women: 2/3575  60 - 69 years: Men: 19/2616, Women: 23/4145  70 - 79 years: Men: 15/1645, Women: 17/1901  ≥ 80 years: Men: 3/152, Women: 3/192 | Zhang L, Nie ZY, Liu Y, et al. The prevalence of PD in a nutritionally deficient rural population in China. Acta neurologica Scandinavica 2005; 112(1): 29-35. |
| Veterans communities in Beijing | December 2005 - August 2007 | ≥ 60 years: 70/3473 | Wang LN, Tan JP, Xie HG, et al. [A cross-sectional study of neurological disease in the veterans of military communities in Beijing]. Zhonghua nei ke za zhi 2010; 49(6): 463-8. |
| Hetian, Xinjiang Uygur Autonomous Region* | November - December 2010 | 45 – 54 years: Men: 5/894, Women: 6/1118  55 – 64 years: Men: 16/1033, Women: 11/930  65 – 74 years: Men: 20/792, Women: 13/655  75 – 84 years: Men: 8/252, Women: 5/166  85 – 94 years: Men: 2/49, Women: 1/19  ≥95 years: Men: 0/14, Women: 1/10 | Yang XL, Luo Q, Song HX, Wang YL, Yao YN, Xia H. Related factors and prevalence of Parkinson's disease among Uygur residents in Hetian, Xinjiang Uygur Autonomous Region. Genetics and molecular research : GMR 2015; 14(3): 8539-46. |
| veterans’ communities in China | December 2009 to December 2012 | 70 - 79 years: Men: 44/2345, Women: 3/213  80 - 89 years: Men: 164/6242, Women: 3/176  ≥90 years: Men: 11/293, Women: 0/6 | Zou YM, Tan JP, Li N, et al. The prevalence of Parkinson's disease continues to rise after 80 years of age: a cross-sectional study of Chinese veterans. European review for medical and pharmacological sciences 2014; 18(24): 3908-15. |
| Yili, Xinjiang Uygur Autonomous Region | June - July, 2012 | Ethnicity: Kazak: 28/2318, Chinese: 16/1474, Uygur: 15-968, Hui: 3/245, Other ethnicities: 0/46  45 – 54 years: 15/2269, 55 – 64 years: 18/1400  65 – 74 years: 19/919, 75 – 84 years: 9/308  ≥85 years: 1/47  Men: 31/2277, Women: 31/2774 | Jianlong Zhang, Yuling Wang, Yani Yao, et al. Analysis of prevalence and related factors in different national Parkinson’s disease in Yili of Xinjiang area. Journal of Xinjiang Medical University 2013; (3): 273-7. (张建龙, 王玉玲, 姚亚妮, 杨新玲. 疆伊犁地区不同民族帕金森病患病率及相关因素分析. 新疆医科大学学报 2013; (3): 273-7.) |
| Urumqi, Xinjiang Uygur Autonomous Region | Unknown. Published in March 2013 | 35 – 44 years: 10/1786  45 – 54 years: 34/3551  55 – 64 years: 32/1807  65 – 74 years: 34/1762  75 – 84 years: 27/692  ≥85 years: 4/134 | Luning Wang, Taifang Zhang, Yuling Wang, et al. Study on the prevalence and relative factors of the Parkinson’s disease in residents aged 35 years or older in Urumqi city. Journal of Xinjiang Medical University 2013; (3): 278-81,86. (王鲁宁, 张太芳, 王玉玲, 杨新玲. 乌鲁木齐市年龄≥35岁人群帕金森病患病率及相关因素分析. 新疆医科大学学报 2013; (3): 278-81,86.) |
| Prevalence: Diagnostic criteria: Criteria brought up by Parkinson’s disease and movement disorders group of Chinese Society of Neurology ^4^ | | | |
| Kashi,  Xinjiang Uygur Autonomous Region | April 2008 – July 2009 | 55 – 59 years: Chinese: 12/1055, Uygur: 7/1338  60 – 64 years: Chinese: 2/507, Uygur: 6/660  65 – 69 years: Chinese: 0/568, Uygur: 4/447  70 – 74 years: Chinese: 3/355, Uygur: 6/359  75 – 79 years: Chinese: 3/221, Uygur: 5/224  80 – 84 years: Chinese: 0/106, Uygur: 3/142  85 – 89 years: Chinese: 1/49, Uygur: 2/62  90 – 94 years: Chinese: 0/10, Uygur: 2/24  95 – 99 years: Chinese: 0/0, Uygur: 1/9 | Yan Liu, Xiaoying Zhang, Ying He. et al. Investigation on prevalence rate of Parkinson's disease in population aged 55 years old and above in Kashi, Xinjiang between 2008 and 2009. Chinese Journal of Neurology 2010; (12): 863-5. (刘燕, 张晓莺, 何瑛, et al. 2008-2009年新疆喀什地区55岁及以上人群帕金森病患病率调查. 中华神经科杂志 2010; (12): 863-5.) |
| Prevalence: Diagnostic criteria: brought up by Huashan Hospital of Fudan University (2005) ^5^ | | | |
| Beihai, Guangxi | October – December 2014 | ≥40 years: Men: 28/1422, Women: 19/1689  40 – 49 years: 3/1013  50 – 59 years: 9/997  60 – 69 years: 18/649  70 – 79 years: 12/329  ≥80 years: 5/123 | Yingxu Mo, Shuwang, Fang Peng. et al. Analysis of related factors Parkinson’s disease in Beihai of Guagnxi area. Journal of Brain and Nervous Diseases 2018; (8): 503-6. (莫英绪, 王姝, 彭芳, et al. 广西北海地区帕金森病相关危险因素的分析. 脑与神经疾病杂志 2018; (8): 503-6.) |
| Prevalence: Diagnostic criteria: brought up by movement disorders society (MDS) ^6^ | | | |
| Aerospace center Hospital Adjacent Communities, Beijing | August 2015 – January 2016 | ≥40 years: Men: 24/1154, Women: 20/1172 | Mengqing Shang, Zhirong Wan, Tao Feng. Et al. Analysis of the Prevalence and Risk Factors for Parkinson's Disease in middle-aged and Elderly Residents of Aerospace Center Hospital Adjacent Communities. Neural Injury and Functional Reconstruction 2017; (1): 28-31. (商梦晴, 万志荣, 冯涛, 杜继臣. 航天中心医院周边社区中老年帕金森病患病率现况分析. 神经损伤与功能重建 2017; (1): 28-31.) |
| Prevalence: diagnosed by neurologists, diagnostic criteria: not given | | | |
| Changsha, Chengdu, Guangzhou, Harbin, Shanghai, Yinchuan | January – March, 1983 | 28/63195 (Crude prevalence: 44/100000, age-adjusted prevalence: 57/100000) | Li SC, Schoenberg BS, Wang CC, et al. A prevalence survey of Parkinson's disease and other movement disorders in the People's Republic of China. Archives of neurology 1985; 42(7): 655-7. |
| Baoshan District, Shanghai | February – May, 1999 | ≥55 years: Men: 2/597, Women: 0/589 | Guochuan Zhang, Baolong Zhao, Suzhen Xu, et al. A survey of prevalence of senile dementia and Parkinson disease. Linchuang Jingshen Yixue Zazhi 2001; (3): 143-5. (张国川, 赵宝龙, 许素珍, 施永斌. 老年期痴呆和帕金森病患病率调查. 临床精神医学杂志 2001; (3): 143-5.) |
| Shanghai | Unknown. Published in 2011 | 55 – 64 years: Men: 8/2509, Women: 3/3013  65 – 74 years: Men: 22/3126, Women: 42/3617  75 – 84 years: Men: 27/1326, Women: 42/1704  ≥85 years: Men: 5/222, Women: 9/393 | Fen Zhou, Zhen Hong, Maosheng Huang, et al.. Prevalence of Parkinson’s Disease in Shanghai urban and rural area. Journal of Brain and Nervous Disease 2001; (6): 330-2. (周玢, 洪震, 黄茂盛, 曾军, 金美华, 吕传真. 上海城乡帕金森病患病率研究. 脑与神经疾病杂志 2001; (6): 330-2.) |
| Jiaxing, Zhejiang Province | January 2004 - June 2006 | Source: Documents from local hospitals  ≥60 years: Men: 59/65031; Women: 55/63384  60 - 69 years: 64/71141  70 - 79 years: 40/44831  ≥80 years: 10/12443 | Yuxia Chu. Research on Parkinson's Disease Morbidity and Correlation between Treatment Time and Rehabilitation of Elderly Patients in Jiaxing. Journal of Jiaxing University 2007; (3): 90-2. (褚玉霞. 嘉兴市老年人帕金森病的研究. 嘉兴学院学报 2007; (3): 90-2.) |
| Luoxing Street Mawei District, Fuzhou, Fujian Province | 2015 – 2017 | ≥65 years: Men: 22/928, Women: 10/843  65 – 69 years: 16/898  70 – 74 years: 6/465  75 – 79 years: 7/276  80 – 84 years: 2/104  ≥85 years: 1/28 | Hongqing Yang, Guangqing Liu, Longhui Zhang, et al. Study on the prevalence of Parkinson's disease and its relationship with metabolic syndrome among the elderly in Luoxing Street Mawei District. Chinese Journal of Practical Nervous Diseases 2017; (24): 79-82. (杨红清, 刘光庆, 张龙惠, et al. 马尾区罗星街道老年人帕金森病患病率与代谢综合征的关系研究. 中国实用神经疾病杂志 2017; (24): 79-82.) |
| Incidence (per 100,000) | | | |
| Ilan County, Taiwan | December, 1997 | 40 - 49 years: Men: 0.0, Women: 0.0, Total: 0.0  50 - 59 years: Men: 25.4, Women: 12.0, Total: 18.5  60 - 69 years: Men: 45.4, Women: 49.6, Total: 47.4  70 - 79 years: Men: 98.1, Women: 102.5, Total: 100.2  ≥ 80 years: Men: 0.0, Women: 0.0, Total: 0.0  Total (40+): Men: 32.5, Women: 27.8, Total: 30.1  Age-adjusted (40+): Men: 30.5, Women: 27.0, Total: 28.7  Age-adjusted (for all age): Men: 11.1, Women: 9.8, Total: 10.4 | Chen RC, Chang SF, Su CL, et al. Prevalence, incidence, and mortality of PD: a door-to-door survey in Ilan county, Taiwan. Neurology 2001; 57(9): 1679-86. |
| Mortality | | | |
| Ilan County, Taiwan | December, 1999 | 5-year cumulative survival rate: 78.85% | Chen RC, Chang SF, Su CL, et al. Prevalence, incidence, and mortality of PD: a door-to-door survey in Ilan county, Taiwan. Neurology 2001; 57(9): 1679-86. |
| Hong Kong | 2002 - 2012 | 10-year standardized mortality ratio: 1.1 | Auyeung M, Tsoi TH, Mok V, et al. Ten year survival and outcomes in a prospective cohort of new onset Chinese Parkinson's disease patients. Journal of neurology, neurosurgery, and psychiatry 2012; 83(6): 607-11. |
| Shanghai | 2006 – 2011 | 5-year standardized mortality ratio: 0.62 | Wang G, Li XJ, Hu YS, et al. Mortality from Parkinson's disease in China: Findings from a five-year follow up study in Shanghai. The Canadian journal of neurological sciences Le journal canadien des sciences neurologiques 2015; 42(4): 242-7. |
| Shanghai | 2006 - 2016 | 10-year standardized mortality ratio: 0.87 | Zhang Y, Wang C, Wang Y, et al. Mortality from Parkinson's disease in China: Findings from a ten-year follow up study in Shanghai. Parkinsonism & related disorders 2018. |
| *The ethnicity of source population of the study was Uygur ethnicity, not Chinese ethnicity. | | | |

**Reference**

1. Morgante L, Rocca WA, Di Rosa AE, et al. Prevalence of Parkinson's disease and other types of parkinsonism: a door-to-door survey in three Sicilian municipalities. The Sicilian Neuro-Epidemiologic Study (SNES) Group. Neurology 1992; 42(10): 1901-7.

2. Tanner CM, Chen B, Wang W, et al. Environmental factors and Parkinson's disease: a case-control study in China. Neurology 1989; 39(5): 660-4.

3. Calne DB, Snow BJ, Lee C. Criteria for diagnosing Parkinson's disease. Annals of neurology 1992; 32 Suppl: S125-7.

4. CMDS. Diagnostic Criteria of Parkinson’s disease. Chinese Journal of Neurology 2006; (6): 408-9.

5. Yuping Jian, Jian Wan, et al. Diagnostic criteria for primary Parkinson’s disease (2005). Chinese Journal of Clinical Neurosciences 2006; (1): 40.

6. Postuma RB, Berg D, Stern M, et al. MDS clinical diagnostic criteria for Parkinson's disease. Movement disorders : official journal of the Movement Disorder Society 2015; 30(12): 1591-601.

**Table S2 Status of Clinical Trials**

| Country | total | Status | | | | international cooperation | Study Type | |
| --- | --- | --- | --- | --- | --- | --- | --- | --- |
|  |  | ongoing | completed | suspended/terminated/  withdrawn | unknown |  | interventional | observational |
| Total | 2150 | 627 | 1126 | 163 | 231 | - | 1645 | 502 |
| China  Mainland China  Hong Kong  Macau  Taiwan | 110  56  2  0  52 | 40  23  1  0  16 | 49  26  0  0  23 | 3  1  0  0  2 | 18  6  1  0  11 | 12  1  0  0  11 | 84  37  2  0  45 | 26  19  0  0  7 |
| the United States | 935 | 290 | 509 | 91 | 45 | 139 | 698 | 237 |
| Canada | 139 | 41 | 72 | 14 | 12 | 77 | 118 | 21 |
| Japan | 43 | 8 | 33 | 2 | 0 | 7 | 35 | 8 |
| the United Kingdom | 106 | 32 | 63 | 7 | 4 | 61 | 91 | 15 |
| India | 17 | 0 | 16 | 1 | 0 | 16 | 17 | 0 |

We searched ClinicalTrials.gov with following phrases: “Parkinson disease” for the blank of “condition or disease”, Country name for the blank of “country”. We divided status of clinical trials into four kind of status: ongoing, completed, suspended/terminated/withdrawn and unknown. Status of those clinical trials including “Not yet recruiting”, “Recruiting”, “Enrolling by invitation”, “Active, not recruiting” were regarded as ongoing status. Clinical trials marked completed were regarded as completed clinical trials. Suspended clinical trials, terminated clinical trials and withdrawn clinical trials were set in one group. Sources from more than one countries were regarded as international cooperation with any kind of status, which could be calculated in “On Map”. As for study type, “interventional” or “observational” was chosen in the list of “study type”. We did not select any kind of eligibility criteria, study phase, funder type or study documents.

Since there was no official demographics data of China in 2017 till November 1^st^, 2018, we took the demographics of China in 2016 as reference. According to the demographics of China in 2016, the population of mainland China was 1,403,500,365 people. The population of Hong Kong was 7,336,585 people. The population of Taiwan was 2,353,9816 people. So the number of clinical trials were 0.0399 per million people in mainland China, 0.2726 per million people in Hong Kong and 22.0902 per million people in Taiwan. Similarly, the number of clinical trials were 2.8938 per million people in the United States (reference to 2016 estimation of population), 3.9543 per million people in Canada (reference to 2016 population), 0.3393 per million people in Japan (reference to 2017 estimation of population), 1.6147 per million people in the United Kingdom (reference to 2016 population), and 0.0128 per million people in India (reference to 2016 estimation of population).

**Data S3**: **Meta analysis of genetic variants for Parkinson’s disease in Chinese population.** PubMed, Embase, Wanfang and China National Knowledge Infrastructure (CNKI) were searched. 3 researchers (Gen Li, Shishuang Cui, Yixi He) screened and collected those data. Any doubts were discussed at the meeting along with senior researcher (Shengdi Chen). We excluded studies whose controls were not selected from healthy population. Studies which did not obey Hardy–Weinberg equilibrium were also excluded. Studies without detailed genotype information or genotype information that could not be inferred were also excluded. Information of single nucleotide polymorphisms (SNPs) were inquired at dbSNP database (<https://www.ncbi.nlm.nih.gov/snp/>). Since patients of Parkinson’s disease were majorly collected from clinics of hospitals, the location of population is referred to the location of hospital. Usually, control group were collected from health management clinics of each hospital, or communities in the same city with hospitals. Source of control group will be declared if there is different population source of control group. Both patients and controls were restricted in Chinese population in China. Chinese ethnicity in other countries, such as Singapore, Malaysia were excluded due to possible genetic shift since few international marriages were taken into place. Studies focused on specific type of PD, such as early onset PD or familial PD were also excluded due to possible different genetic distribution to general PD and it is impossible to approach to real genetic distribution with mathematical approach. SNPs with single homozygous results in Chinese populations were not on this list.

1. **rs25531, *5-HTTLPR*, A>G**

| Articles | PD subjects | | | Controls | | | Methods | Location of Population |
| --- | --- | --- | --- | --- | --- | --- | --- | --- |
|  | AA | AG | GG | AA | AG | GG |  |  |
| Xiong Zhang et al., 2014 ^a, 1^ | 380 | 114 | 10 | 355 | 137 | 12 | PCR-RFLP | Wenzhou, Zhejiang Province |
| Weiwei Liu et al., 2016 ^a, 2^ | 226 | 2 | 5 | 243 | 3 | 6 | PCR-RFLP | Qingdao, Shandong Province |
| PCR: Polymerase chain reaction; PD: Parkinson’s disease; RFLP: restricted fragment length polymorphism  a: diagnostic criteria: the United Kingdom brain bank criteria ^3^ | | | | | | | | |

**Allele model**: The forest plot and result of allele model of rs25531 is shown below. We regarded allele G as risk allele. Since there was no heterogeneity observed, results of fixed effect model were adopted. After meta-analysis to 737 PD patients and 756 controls, OR was 0.81 (0.64 – 1.03) compared to allele A. *p* value: 0.083.


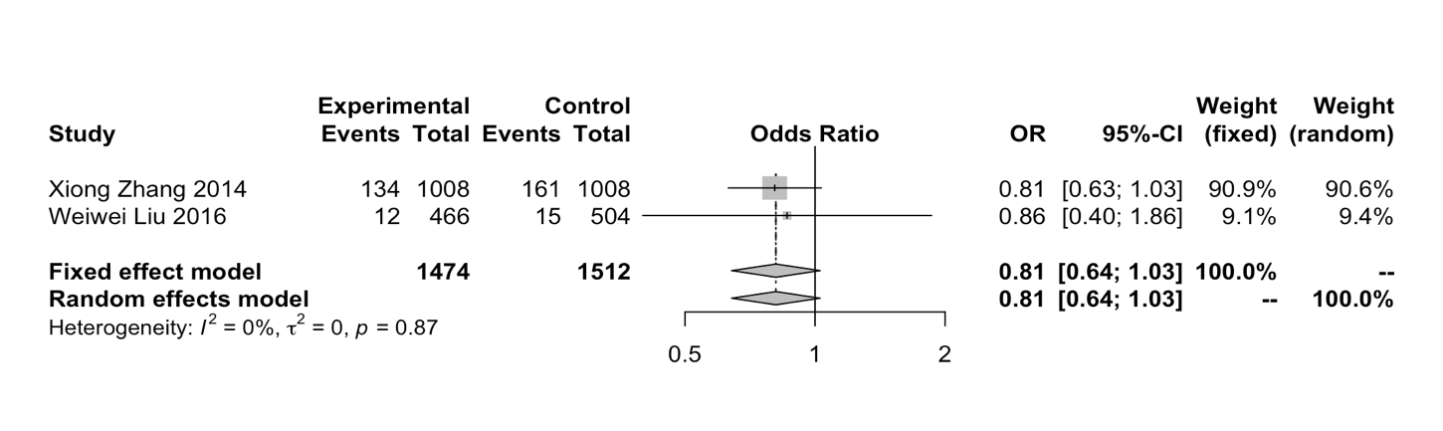


Appendix Figure 3.1 Allele model of *5-HTTLPR* rs25531

**Dominant model**: The forest plot and result of dominant model of rs25531 is shown below. Since there was no heterogeneity observed, results of fixed effect model were adopted. After meta-analysis to 737 PD patients and 756 controls, OR was 0.78 (0.60 – 1.02), *p* value: 0.072.


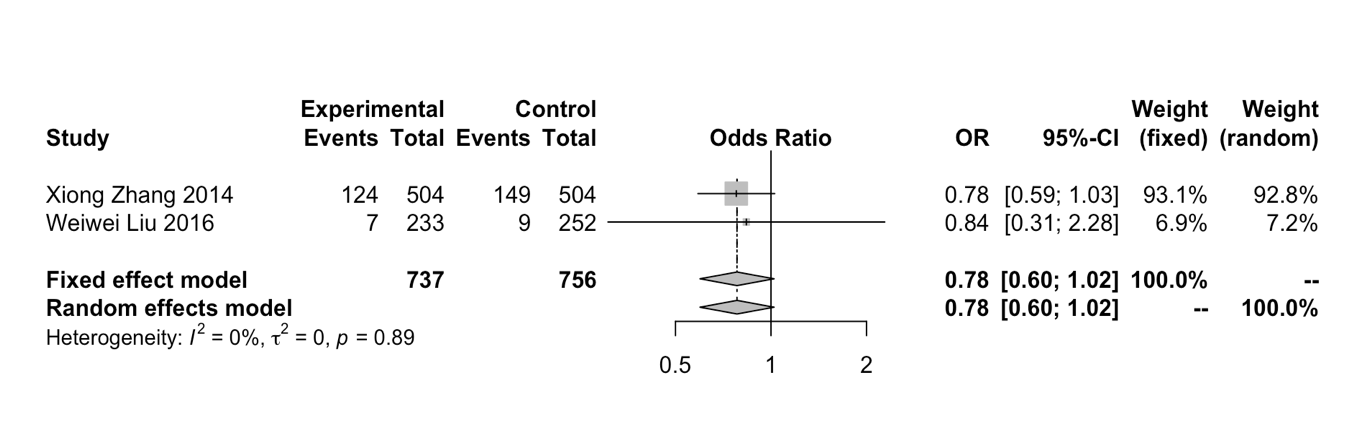


Appendix Figure 3.2 Dominant model of *5-HTTLPR* rs25531

**Recessive model**: The forest plot and result of recessive model of rs25531 is shown below. Since there was no heterogeneity observed, results of fixed effect model were adopted. After meta-analysis to 737 PD patients and 756 controls, OR was 0.85 (0.43 – 1.70), *p* value: 0.651.


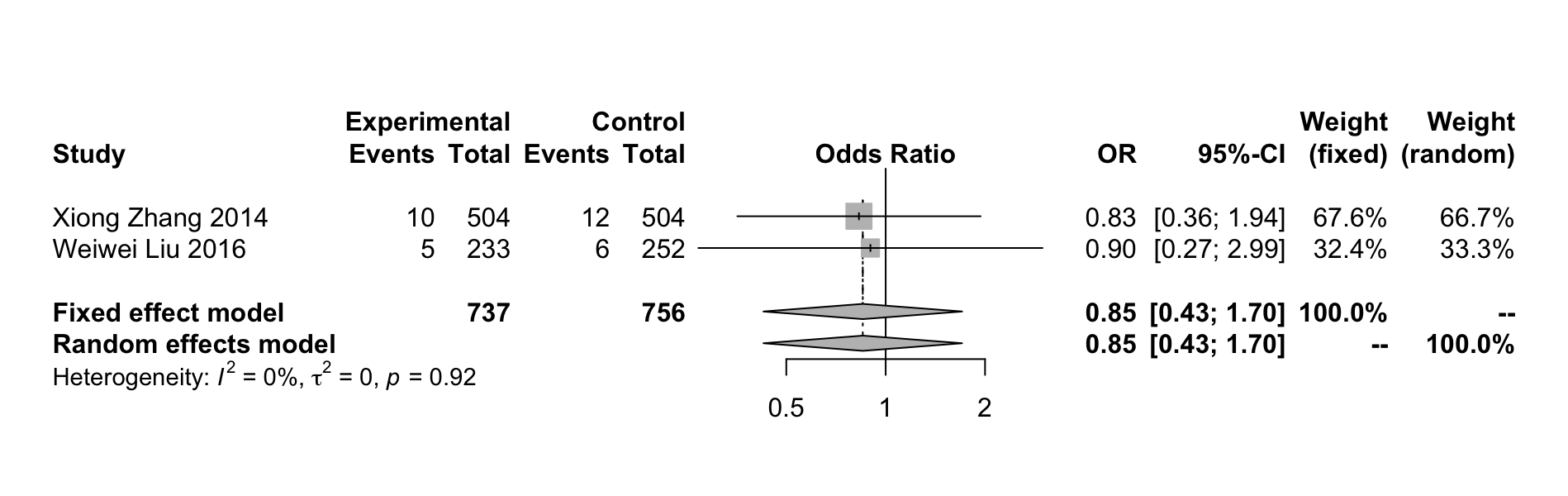


Appendix Figure 3.3 Recessive model of *5-HTTLPR* rs25531

**Overdominant model**: The forest plot and result of recessive model of rs25531 is shown below. Since there was no heterogeneity observed, results of fixed effect model were adopted. After meta-analysis to 737 PD patients and 756 controls, OR was 1.28 (0.96 – 1.70), *p* value: 0.087.


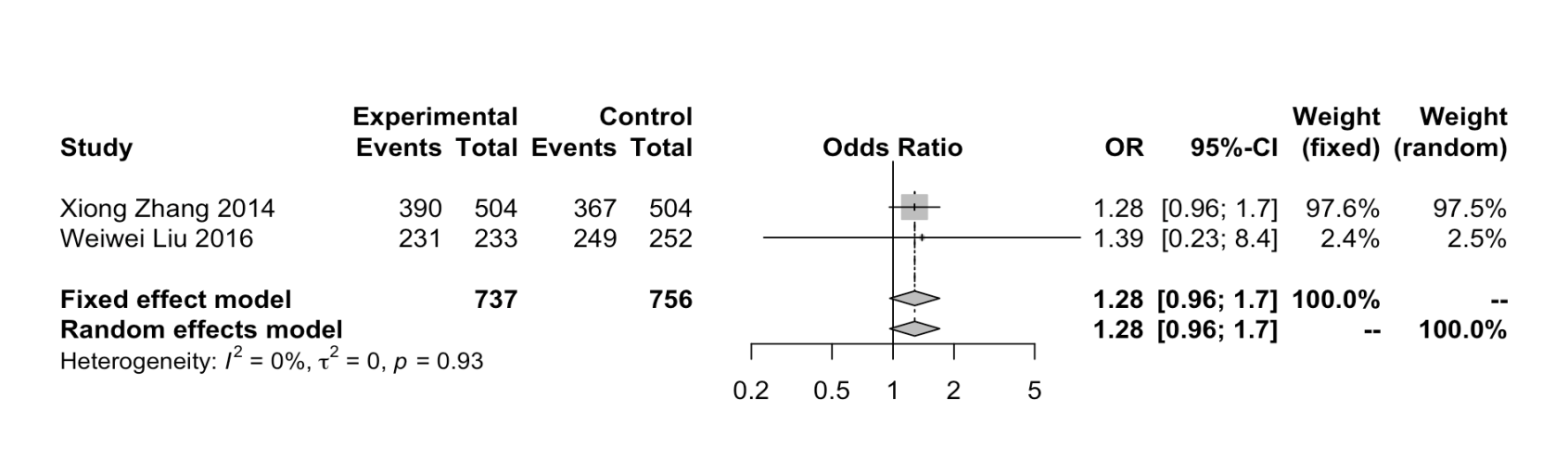


Appendix Figure 3.4 Overdominant model of *5-HTTLPR* rs25531

1. **rs669, *A2M*, A>G, I100V**

| Articles | PD Subjects | | | Controls | | | Methods | Location of Population |
| --- | --- | --- | --- | --- | --- | --- | --- | --- |
|  | AA | AG | GG | AA | AG | GG |  |  |
| Guomei Tang et al., 2002a, ^4^ | 54 | 12 | 0 | 176 | 13 | 1 | PCR-RFLP | Shanghai |
| Ying Xiao et al., 2006b, ^5^ | 66 | 19 | 2 | 90 | 8 | 2 | PCR-RFLP | Tianjin |
| PCR: Polymerase chain reaction; PD: Parkinson’s disease; RFLP: restricted fragment length polymorphism  a: diagnostic criteria: the presence of two or more cardinal features of PD (tremor, rigidity, and bradykinesia) and confirmed by neurologists  b: diagnostic criteria: CAPIT criteria^6^ | | | | | | | | |

**Allele model**: The forest plot and result of allele model of rs669 is shown below. We regarded allele G as risk allele. Since there was no heterogeneity observed, results of fixed effect model were adopted. After meta-analysis to 153 PD patients and 290 controls, OR was 2.41 (1.41 – 4.12) compared to allele A. *p* value: 0.0014.


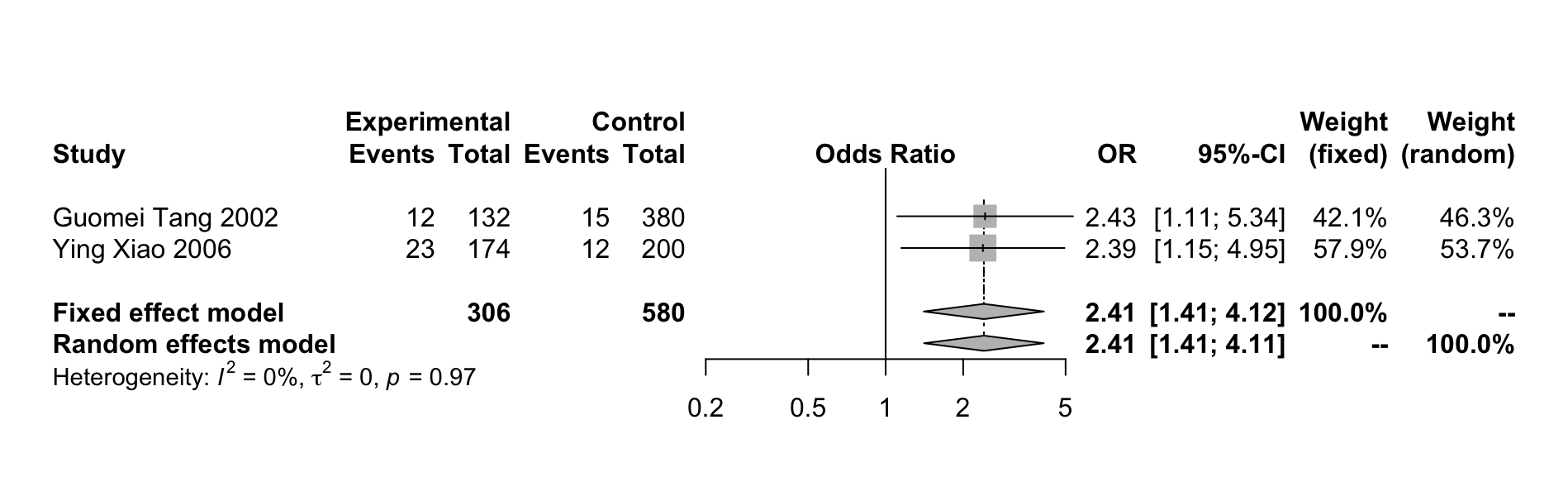


Appendix Figure 3.5 Allele model of *A2M* rs669

**Dominant model**: The forest plot and result of dominant model of rs669 is shown below. Since there was no heterogeneity observed, results of fixed effect model were adopted. After meta-analysis to 153 PD patients and 290 controls, OR was 2.83 (1.58 – 5.08), *p* value: 0.0005.


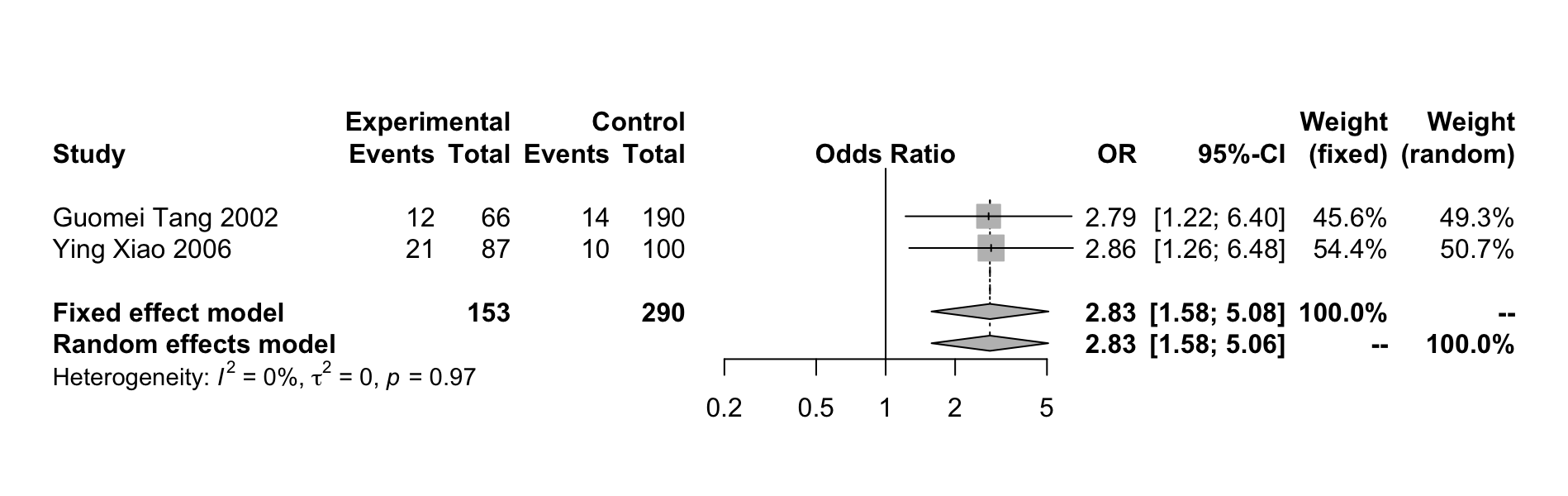


Appendix Figure 3.6 Dominant model of *A2M* rs669

**Recessive model**: The forest plot and result of recessive model of rs669 is shown below. Since there was no heterogeneity observed, results of fixed effect model were adopted. After meta-analysis to 153 PD patients and 290 controls, OR was 1.09 (0.20 – 5.85), *p* value: 0.918.


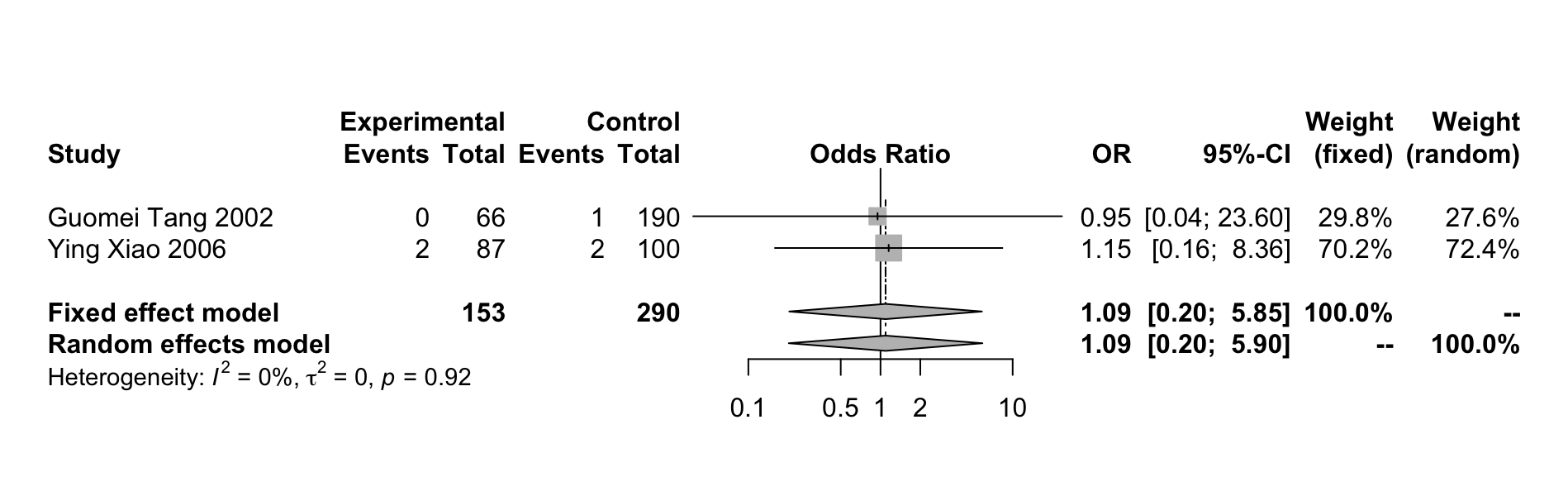


Appendix Figure 3.7 Recessive model of *A2M* rs669

**Overdominant model**: The forest plot and result of recessive model of rs669 is shown below. Since there was no heterogeneity observed, results of fixed effect model were adopted. After meta-analysis to 153 PD patients and 290 controls, OR was 0.32 (0.17 – 0.59), *p* value: 0.0003.


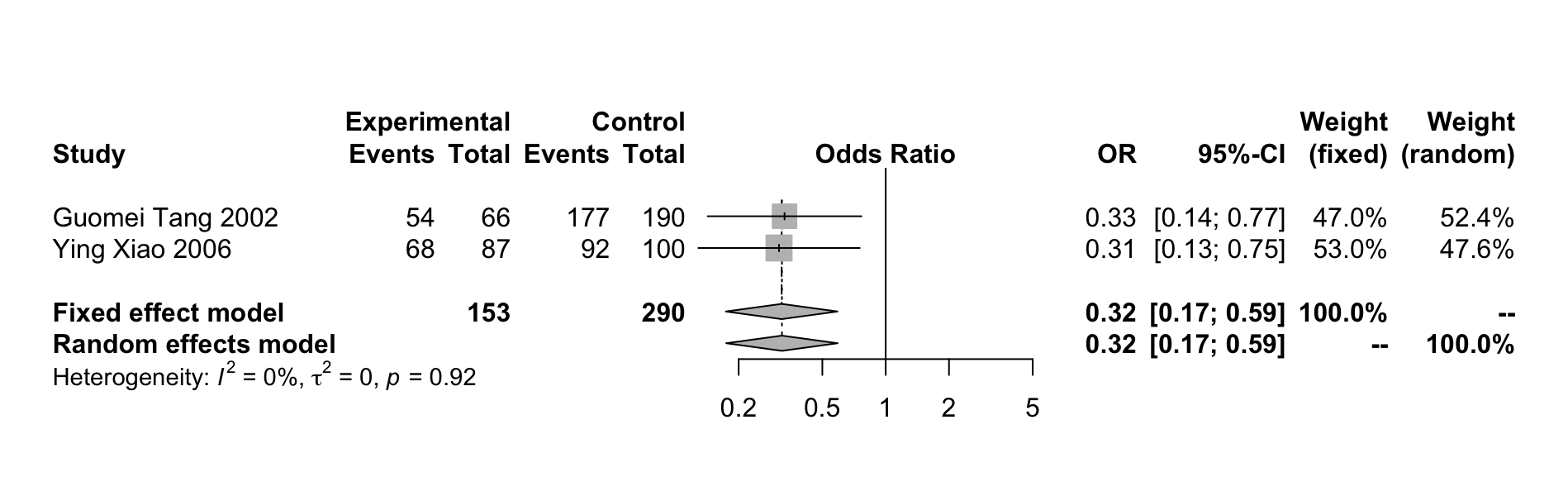


Appendix Figure 3.8 Overdominant model of *A2M* rs669

1. **rs3832852, A2M, D/Ins**

| Articles | PD subjects | | | Controls | | | Methods | Location of Population |
| --- | --- | --- | --- | --- | --- | --- | --- | --- |
|  | DD | DI | II | DD | DI | II |  |  |
| Yixin Hao et al., 2002^a, 7^ | 0 | 1 | 65 | 3 | 8 | 173 | PCR-RFLP | Shanghai |
| Ying Xiao et al., 2006 ^b, 5^ | 1 | 4 | 82 | 3 | 8 | 89 | PCR-RFLP | Tianjin |
| PCR: Polymerase chain reaction; PD: Parkinson’s disease; RFLP: restricted fragment length polymorphism  a: diagnostic criteria: The criteria made in the first Chinese extrapyramidal disorders meeting  b: diagnostic criteria: CAPIT criteria ^6^ | | | | | | | | |

**Allele model**: The forest plot and result of allele model of rs3832852 is shown below. We regarded allele Ins as risk allele. Since there was no heterogeneity observed, results of fixed effect model were adopted. After meta-analysis to 153 PD patients and 284 controls, OR was 2.70 (1.13 – 6.44) compared to allele D. *p* value: 0.026.


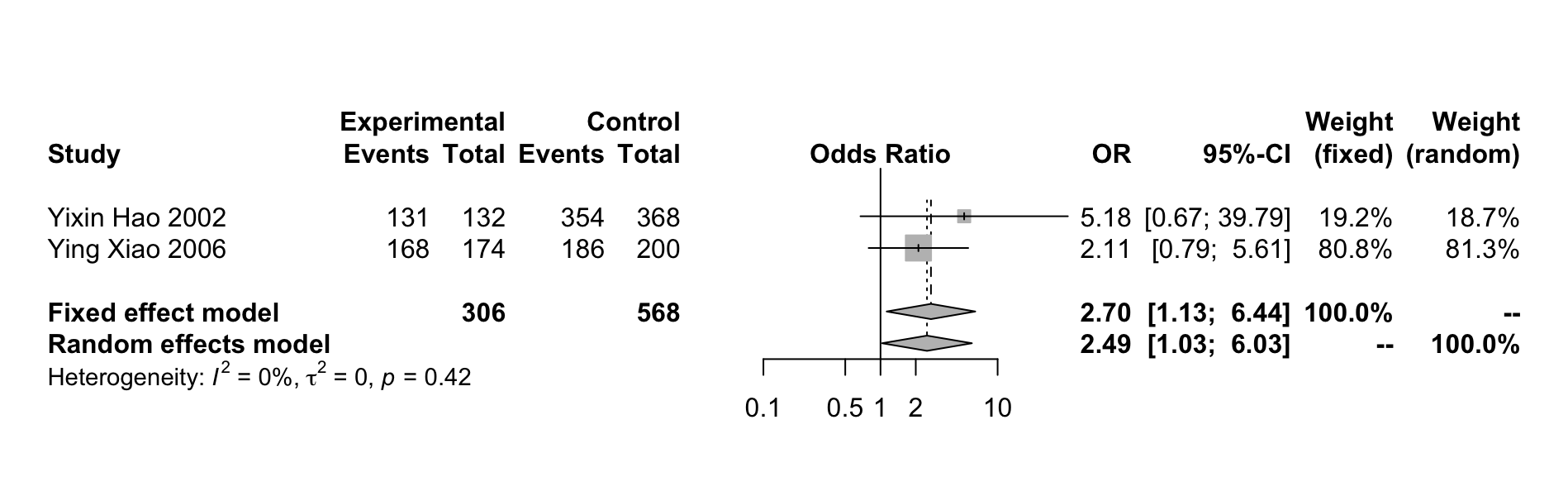


Appendix Figure 3.9 Allele model of *A2M* rs3832852

**Dominant model**: The forest plot and result of dominant model of rs3832852 is shown below. Since there was no heterogeneity observed, results of fixed effect model were adopted. After meta-analysis to 153 PD patients and 284 controls, OR was 2.62 (0.43 – 16.13), *p* value: 0.299.


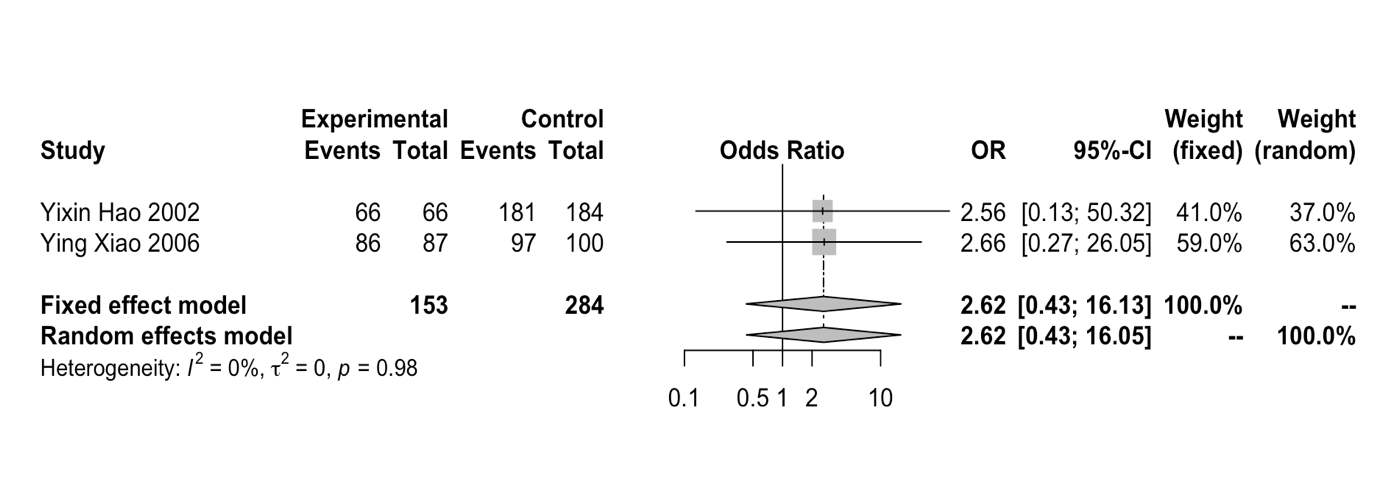


Appendix Figure 3.10 Dominant model of *A2M* rs3832852

**Recessive model**: The forest plot and result of recessive model of rs3832852 is shown below. Since there was no heterogeneity observed, results of fixed effect model were adopted. After meta-analysis to 153 PD patients and 284 controls, OR was 2.50 (0.96 – 6.53), *p* value: 0.061.


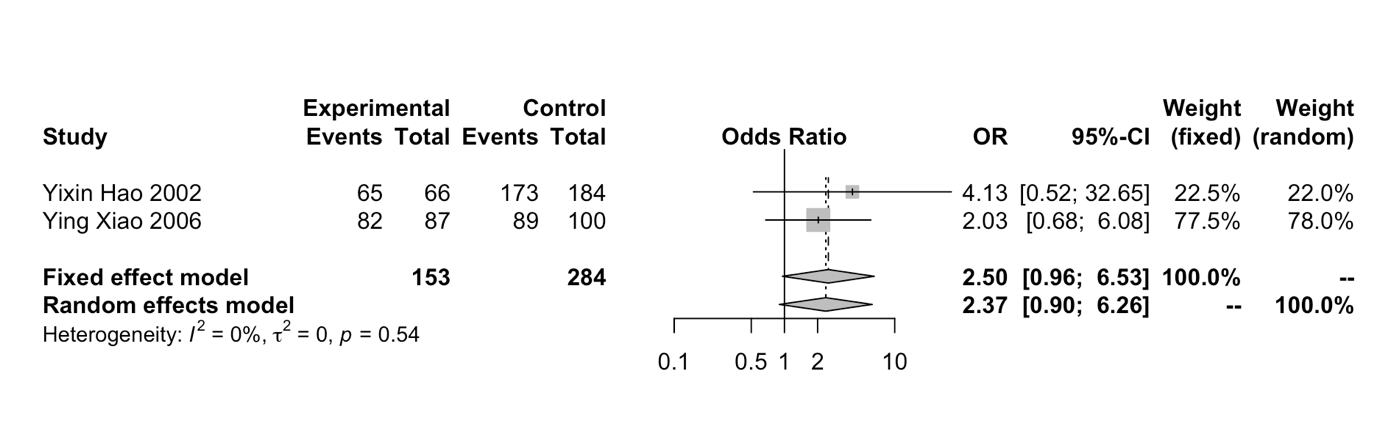


Appendix Figure 3.11 Recessive model of *A2M* rs3832852

**Overdominant model**: The forest plot and result of recessive model of rs3832852 is shown below. Since there was no heterogeneity observed, results of fixed effect model were adopted. After meta-analysis to 153 PD patients and 284 controls, OR was 2.11 (0.73 – 6.06), *p* value: 0.167.


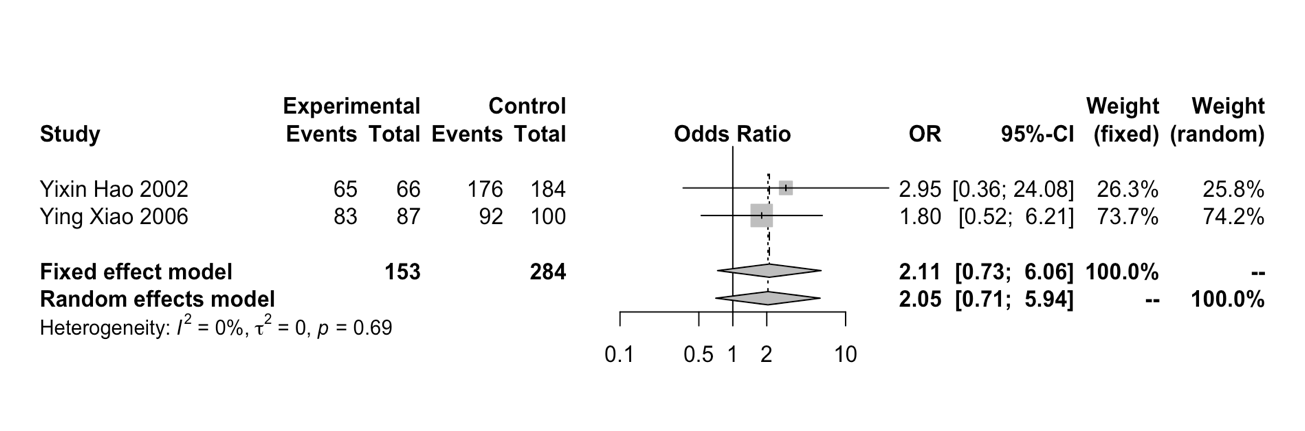


Appendix Figure 3.12 Overdominant model of *A2M* rs3832852

1. **rs671, *ALDH2*, Glu487Lys, G>A**

| Articles | PD subjects | | | Controls | | | Methods | Location of Population |
| --- | --- | --- | --- | --- | --- | --- | --- | --- |
|  | AA | AG | GG | AA | AG | GG |  |  |
| Xiong Zhang et al., 2015^a, 8^ | 27 | 236 | 321 | 35 | 208 | 339 | PCR-RFLP | Wenzhou, Zhejiang Province |
| C.C.Zhao et al., 2016^a, 9^ | 12 | 31 | 71 | 5 | 52 | 157 | PCR-RFLP | Lanzhou, Gansu Province |
| PCR: Polymerase chain reaction; PD: Parkinson’s disease; RFLP: restricted fragment length polymorphism  a: diagnostic criteria: the United Kingdom brain bank criteria ^3^ | | | | | | | | |

**Allele model**: The forest plot and result of allele model of rs671 is shown below. We regarded allele A as risk allele. Since there was heterogeneity observed (*I*^2^ 84.4%), results of random effect model were adopted. After meta-analysis to 698 PD patients and 796 controls, OR was 1.37 (0.78 – 2.40) compared to allele G. *p* value: 0.279.


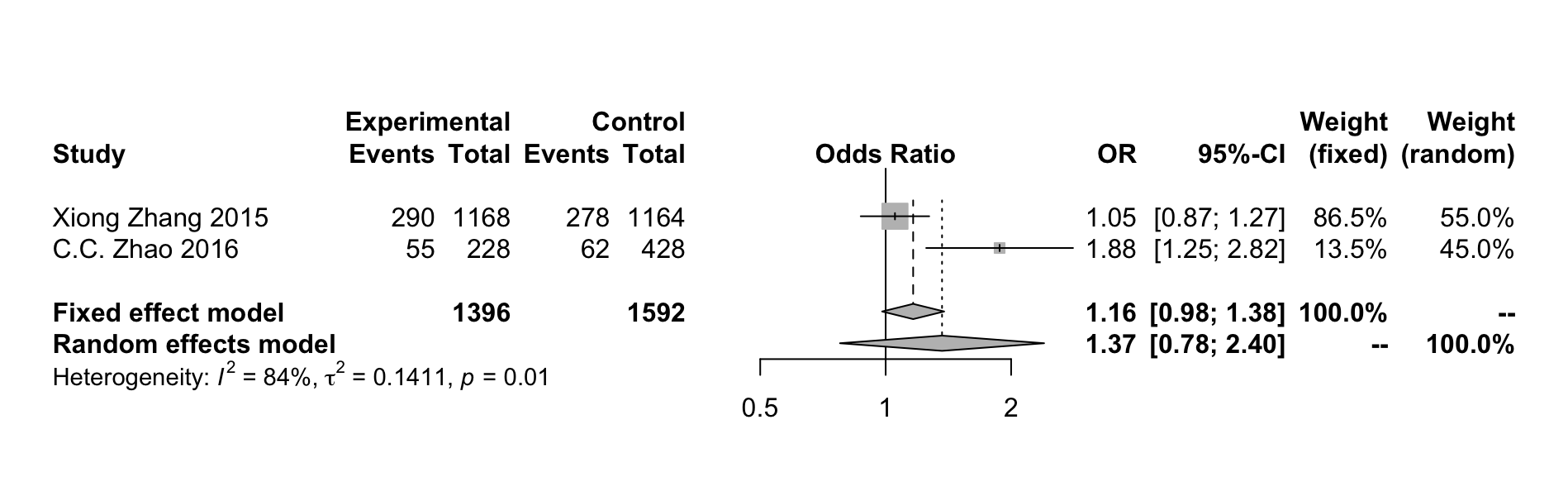


Appendix Figure 3.13 Allele model of *ALDH2* rs671

**Dominant model**: The forest plot and result of dominant model of rs671 is shown below. Since there was no heterogeneity observed (*I*^2^ 47.4%), results of fixed effect model were adopted. After meta-analysis to 698 PD patients and 796 controls, OR was 1.22 (0.99 – 1.51), *p* value: 0.057.


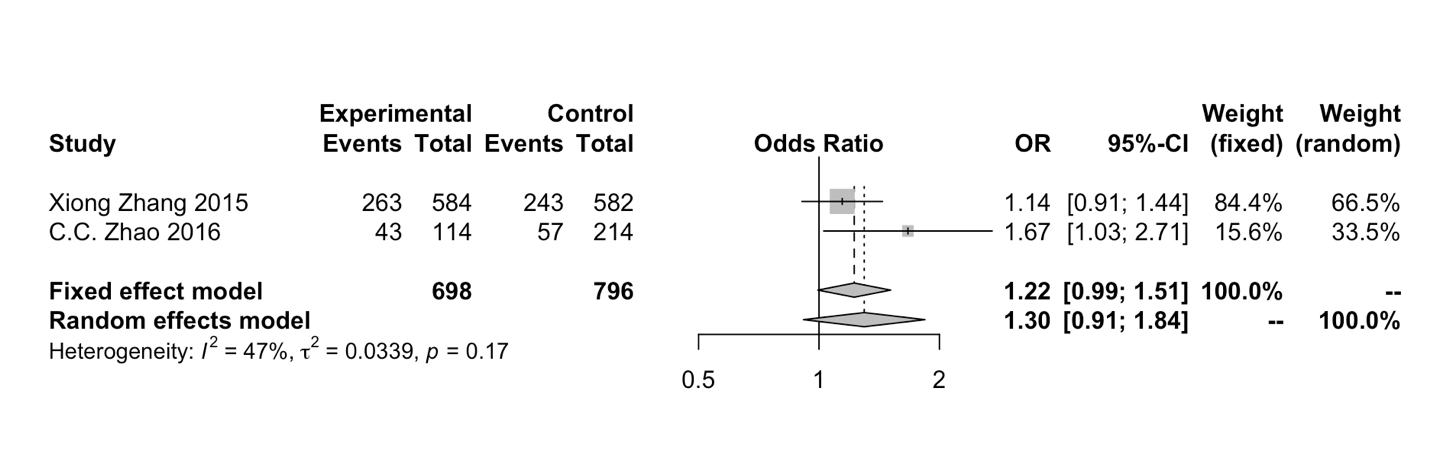


Appendix Figure 3.14 Dominant model of *ALDH2* rs671

**Recessive model**: The forest plot and result of recessive model of rs671 is shown below. Since there was heterogeneity observed (*I*^2^ 89.5%), results of random effect model were adopted. After meta-analysis to 698 PD patients and 796 controls, OR was 1.82 (0.29 – 11.33), *p* value: 0.523.


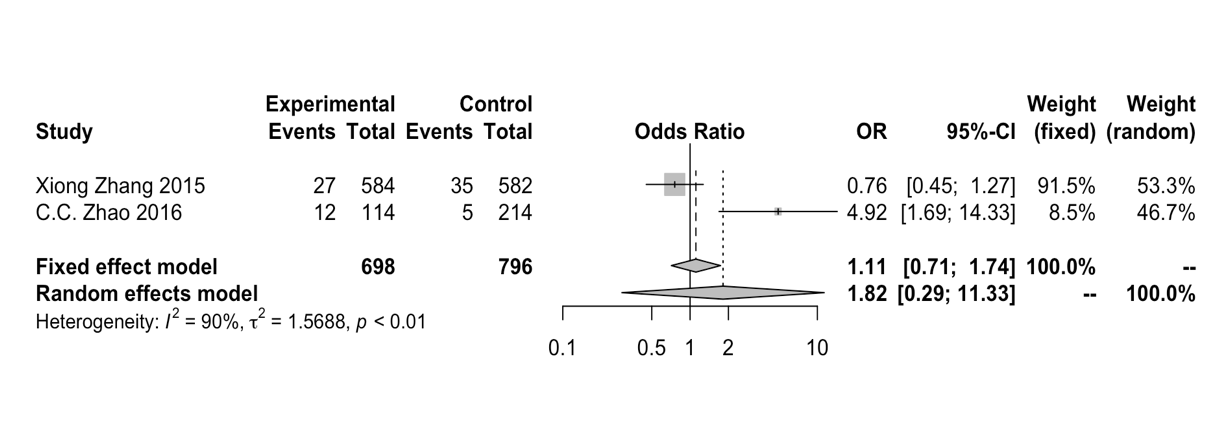


Appendix Figure 3.15 Recessive model of *ALDH2* rs671

**Overdominant model**: The forest plot and result of recessive model of rs671 is shown below. Since there was no heterogeneity observed, results of fixed effect model were adopted. After meta-analysis to 698 PD patients and 796 controls, OR was 0.83 (0.67 – 1.03), *p* value: 0.083.


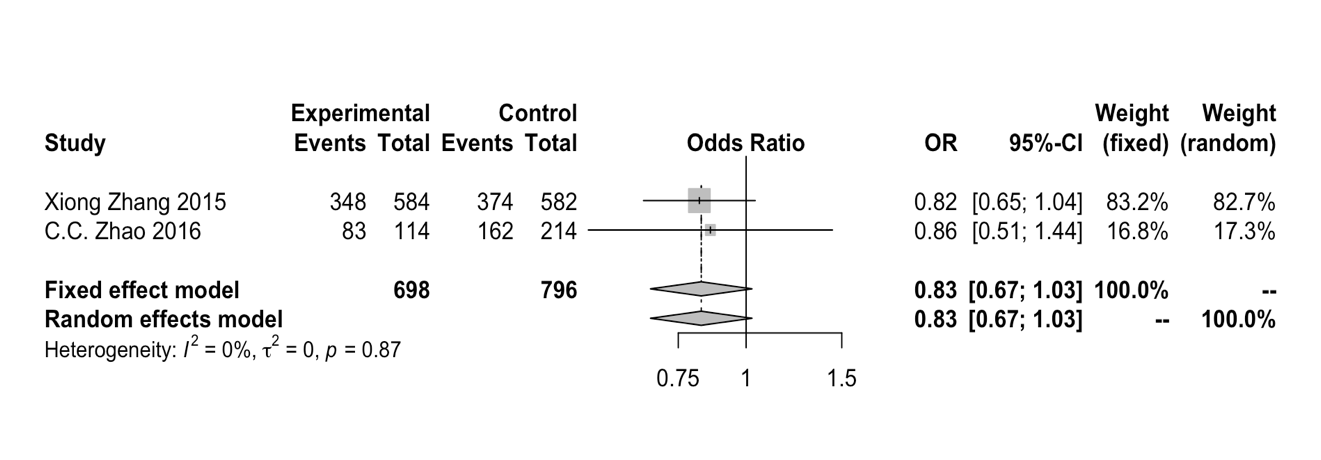


Appendix Figure 3.16 Overdominant model of *ALDH2* rs671

1. ***ATP13A2*, rs147277743, Ala746Thr, c.2236G>A**

| Articles | PD subjects | | | Controls | | | Methods | Location of Population |
| --- | --- | --- | --- | --- | --- | --- | --- | --- |
|  | AA | AG | GG | AA | AG | GG |  |  |
| C.H. Lin et al., 2008^a, 10^ | 0 | 2 | 69 | 0 | 0 | 220 | PCR-RFLP | Taiwan |
| Qing-Zhou Fei et al., 2010^b, 11^ | 0 | 1 | 531 | 0 | 1 | 479 | PCR-RFLP | Shanghai |
| C.M. Chen et al., 2011^a, 12^ | 0 | 2 | 491 | 0 | 4 | 581 | PCR | Taiwan |
| Anne Y.Y. Chan et al., 2013^b, 13^ | 0 | 2 | 259 | 0 | 1 | 179 | PCR | Hong Kong |
| Fang Wang et al., 2013^b, 14^ | 0 | 1 | 234 | 0 | 0 | 216 | PCR-RFLP | Xinjiang |
| Fang Wang et al., 2013^b,*, 14^ | 0 | 0 | 194 | 0 | 0 | 174 | PCR-RFLP | Xinjiang |
| PCR: Polymerase chain reaction; PD: Parkinson’s disease; RFLP: restricted fragment length polymorphism  a: diagnostic criteria: brought up by Gelb et al. ^15^  b: diagnostic criteria: the United Kingdom brain bank criteria^3^  * population sourced from Uygur ethnicity and this part was not included into meta-analysis due to different ethnicity source | | | | | | | | |

**Allele model**: The forest plot and result of allele model of Ala746Thr is shown below. We regarded allele A as risk allele. Since there was no heterogeneity observed, results of fixed effect model were adopted. After meta-analysis to 1592 PD patients and 1681 controls, OR was 1.50 (0.57 – 3.96) compared to allele G. *p* value: 0.4157.


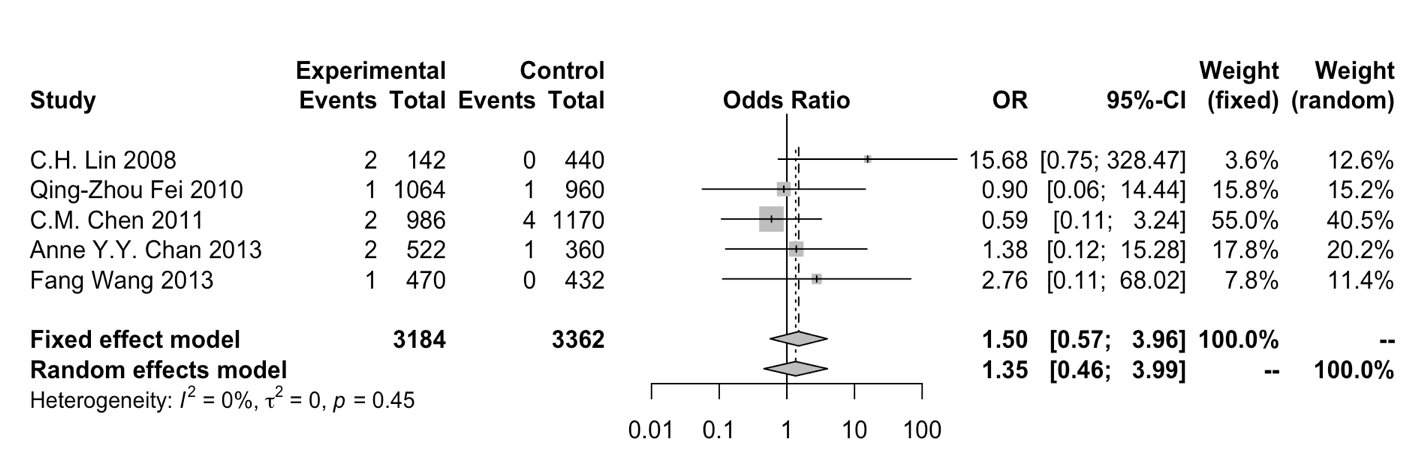


Appendix Figure 3.17 Allele model of *ATP13A2* Ala746Thr

**Dominant model**: The forest plot and result of dominant model of Ala746Thr is shown below. Since there was no heterogeneity observed, results of fixed effect model were adopted. After meta-analysis to 1592 PD patients and 1681 controls, OR was 1.50 (0.57 – 3.97), *p* value: 0.4158.


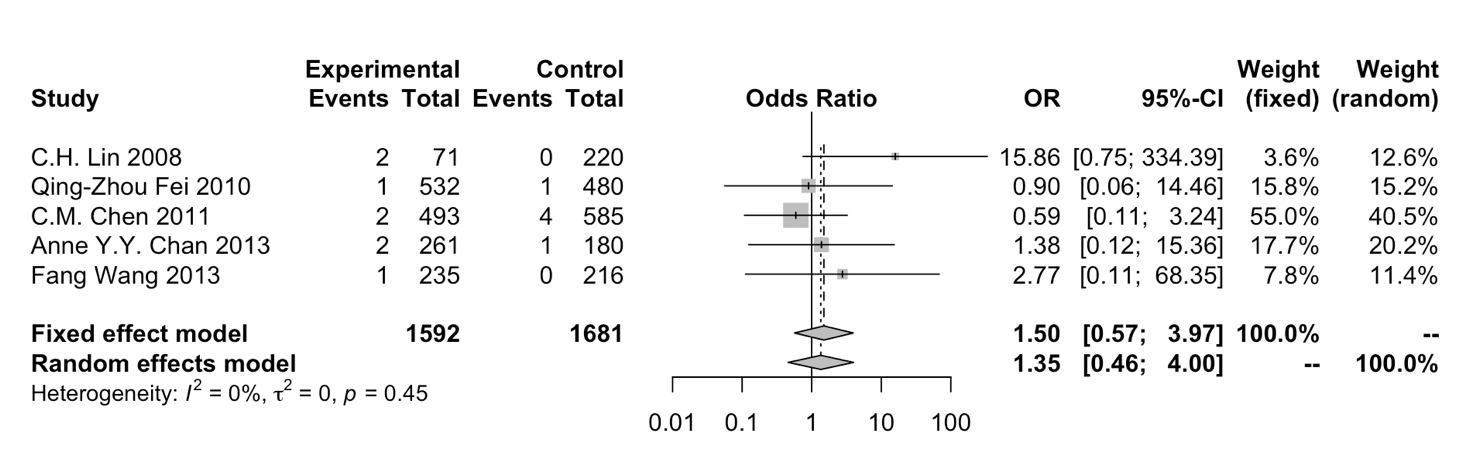


Appendix Figure 3.18 Dominant model of *ATP13A2* Ala746Thr

**Recessive model**: It was not applied since there was no homozygous of AA was observed.

**Overdominant model**: The forest plot and result of recessive model of Ala746Thr is shown below. Since there was no heterogeneity observed, results of fixed effect model were adopted. After meta-analysis to 1592 PD patients and 1681 controls, OR was 0.67 (0.25 – 1.77), *p* value: 0.4158.


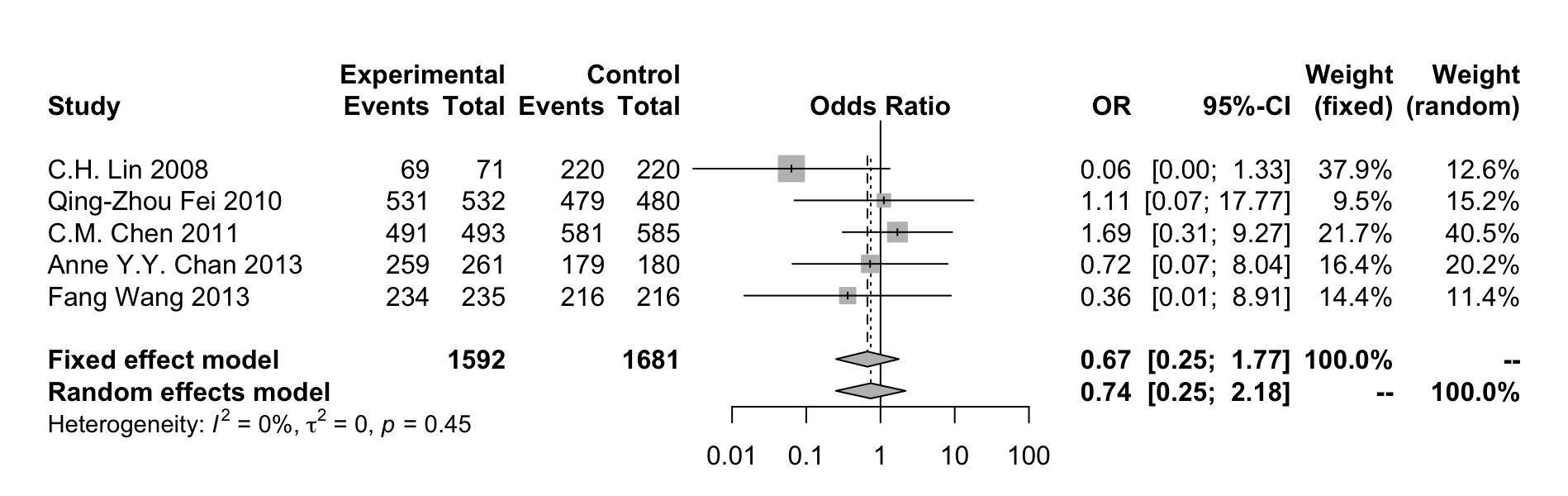


Appendix Figure 3.19 Overdominant model of *ATP13A2* Ala746Thr

1. **rs6265, *BDNF*, V66M, G196A**

| Articles | PD subjects | | | Controls | | | Methods | Location of Population |
| --- | --- | --- | --- | --- | --- | --- | --- | --- |
|  | AA | AG | GG | AA | AG | GG |  |  |
| Dan Hu et al., 2005^a, 16^ | 15 | 43 | 27 | 36 | 102 | 58 | PCR-RFLP | Wuhan, Hubei Province |
| Ling Chen et al., 2011^a, 17^ | 70 | 133 | 63 | 96 | 197 | 107 | PCR-RFLP | Nanjing, Jiangsu Province |
| Jia Liu et al., 2012^a,^ ^18^ | 109 | 245 | 110 | 120 | 278 | 151 | TaqMan | Beijing |
| Caiyou Hu et al., 2015^b,*, 19^ | 17 | 52 | 43 | 27 | 75 | 54 | ARMS-PCR | Nanning, Guangxi |
| ARMS: amplification refractory mutation system; PCR: Polymerase chain reaction; PD: Parkinson’s disease; RFLP: restricted fragment length polymorphism  a: diagnostic criteria: the United Kingdom brain bank criteria ^3^  b: diagnosed by neurologists  * population sourced from Zhuang ethnicity and this part was not included into meta-analysis due to different ethnicity source | | | | | | | | |

**Allele model**: The forest plot and result of allele model of rs6265 is shown below. We regarded allele A as risk allele. Since there was no heterogeneity observed, results of fixed effect model were adopted. After meta-analysis to 815 PD patients and 1145 controls, OR was 1.09 (0.96 – 1.24) compared to allele G. *p* value: 0.180.


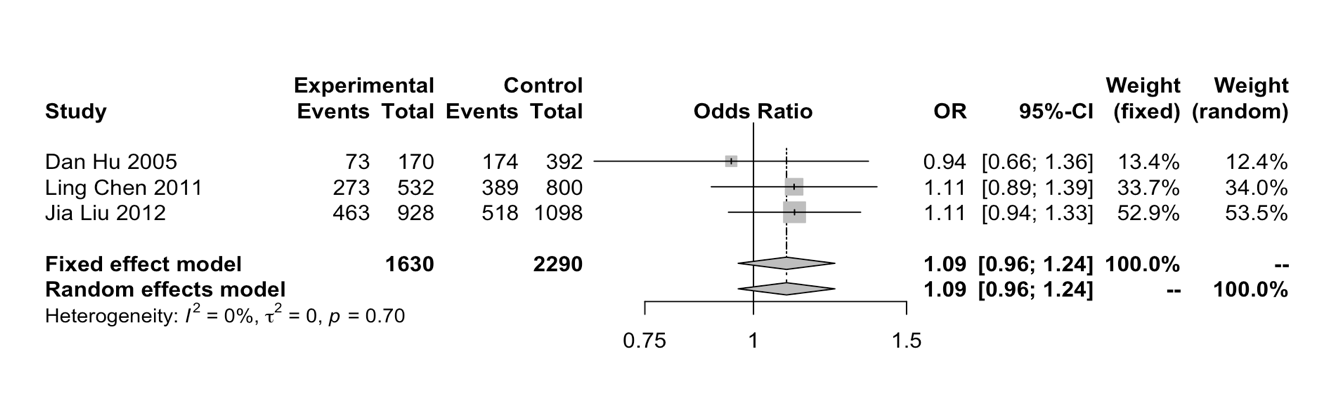


Appendix Figure 3.20 Allele model of *BDNF* rs6265

**Dominant model**: The forest plot and result of dominant model of rs6265 is shown below. Since there was no heterogeneity observed, results of fixed effect model were adopted. After meta-analysis to 815 PD patients and 1145 controls, OR was 1.16 (0.94 – 1.42), *p* value: 0.168.


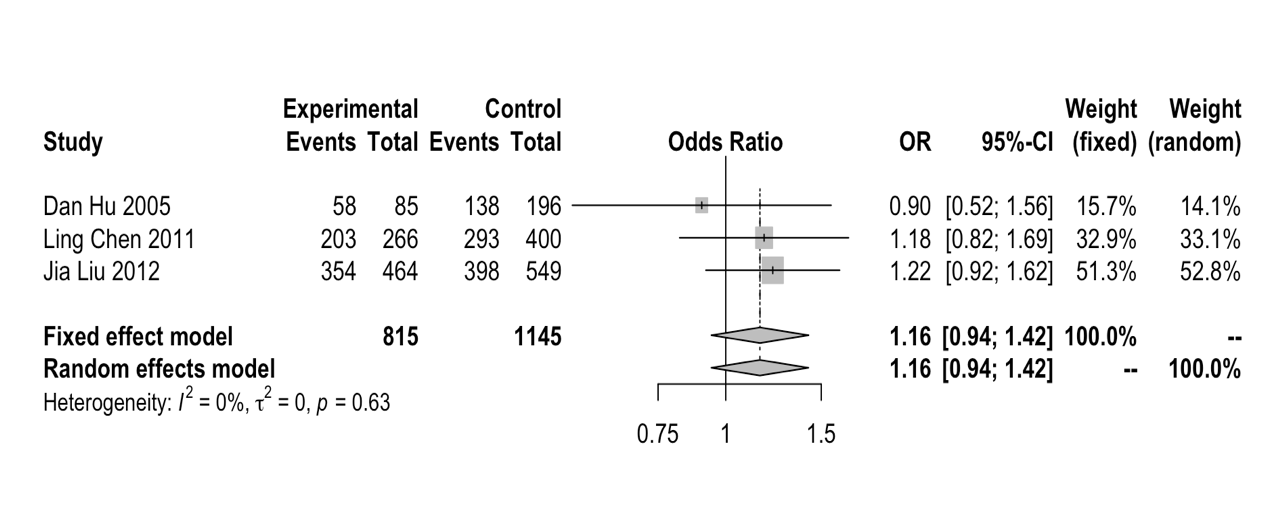


Appendix Figure 3.21 Dominant model of *BDNF* rs6265

**Recessive model**: The forest plot and result of recessive model of rs6265 is shown below. Since there was no heterogeneity observed, results of fixed effect model were adopted. After meta-analysis to 815 PD patients and 1145 controls, OR was 1.09 (0.88 – 1.36), *p* value: 0.417.


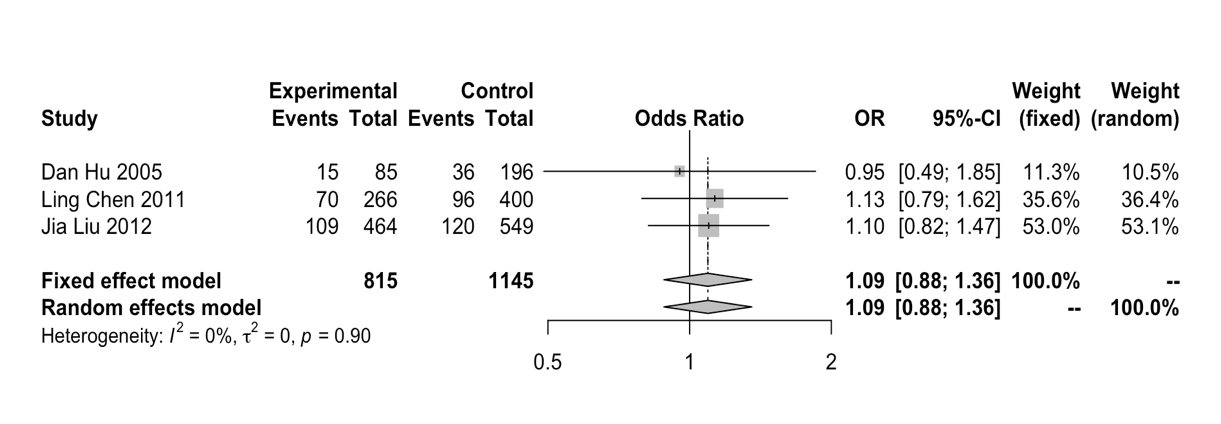


Appendix Figure 3.22 Recessive model of *BDNF* rs6265

**Overdominant model**: The forest plot and result of recessive model of rs6265 is shown below. Since there was no heterogeneity observed, results of fixed effect model were adopted. After meta-analysis to 815 PD patients and 1145 controls, OR was 0/95 (0.79 – 1.14), *p* value: 0.594.


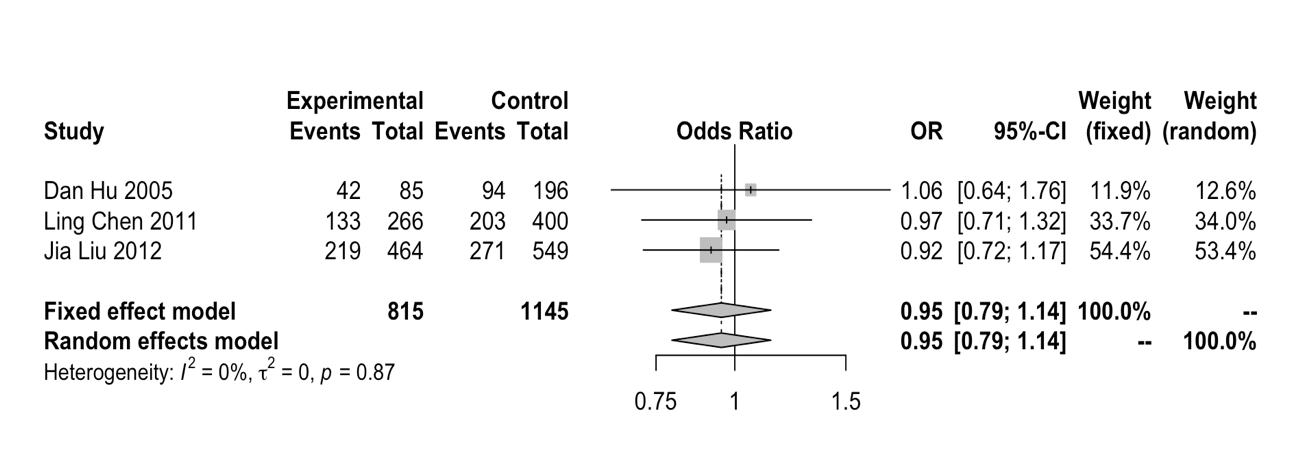


Appendix Figure 3.23 Overdominant model of *BDNF* rs6265

1. ***BDNF*, C270T**

| Articles | PD subjects | | | Controls | | | Methods | Location of Population |
| --- | --- | --- | --- | --- | --- | --- | --- | --- |
|  | CC | CT | TT | CC | CT | TT |  |  |
| Dan Hu et al., 2005^a,16^ | 85 | 0 | 0 | 196 | 0 | 0 | PCR-RFLP | Wuhan, Hubei Province |
| Ling Chen et al., 2011ª^, 17^ | 237 | 29 | 0 | 359 | 41 | 0 | PCR-RFLP | Nanjing, Jiangsu Province |
| PCR: Polymerase chain reaction; PD: Parkinson’s disease; RFLP: restricted fragment length polymorphism  a: diagnostic criteria: the United Kingdom brain bank criteria ^3^ | | | | | | | | |

Since all participants were CC homozygote, there was not applied for meta-analysis.

1. **rs4698412, BST1, G>A**

| Articles | PD subjects | | | Controls | | | Methods | Location of Population |
| --- | --- | --- | --- | --- | --- | --- | --- | --- |
|  | AA | AG | GG | AA | AG | GG |  |  |
| Di Xie et al., 2011ª^, 20^ | 182 | 346 | 216 | 146 | 338 | 259 | PCR-RFLP | Changsha, Hunan Province |
| Xue-Li Chang et al., 2011^a,21^ | 88 | 342 | 189 | 69 | 248 | 185 | MassARRAY | Chengdu, Sichuan Province |
| Ji-Feng Guo et al., 2015^a,22^ | 227 | 487 | 305 | 172 | 477 | 381 | PCR | Changsha, Hunan Province |
| PCR: Polymerase chain reaction; PD: Parkinson’s disease; RFLP: restricted fragment length polymorphism  a: diagnostic criteria: the United Kingdom brain bank criteria ^3^ | | | | | | | | |

**Allele model**: The forest plot and result of allele model of rs4698412 is shown below. We regarded allele A as risk allele. Since there was no heterogeneity observed, results of fixed effect model were adopted. After meta-analysis to 2382 PD patients and 2275 controls, OR was 1.24 (1.14 – 1.35) compared to allele G. *p* value: <0.0001.


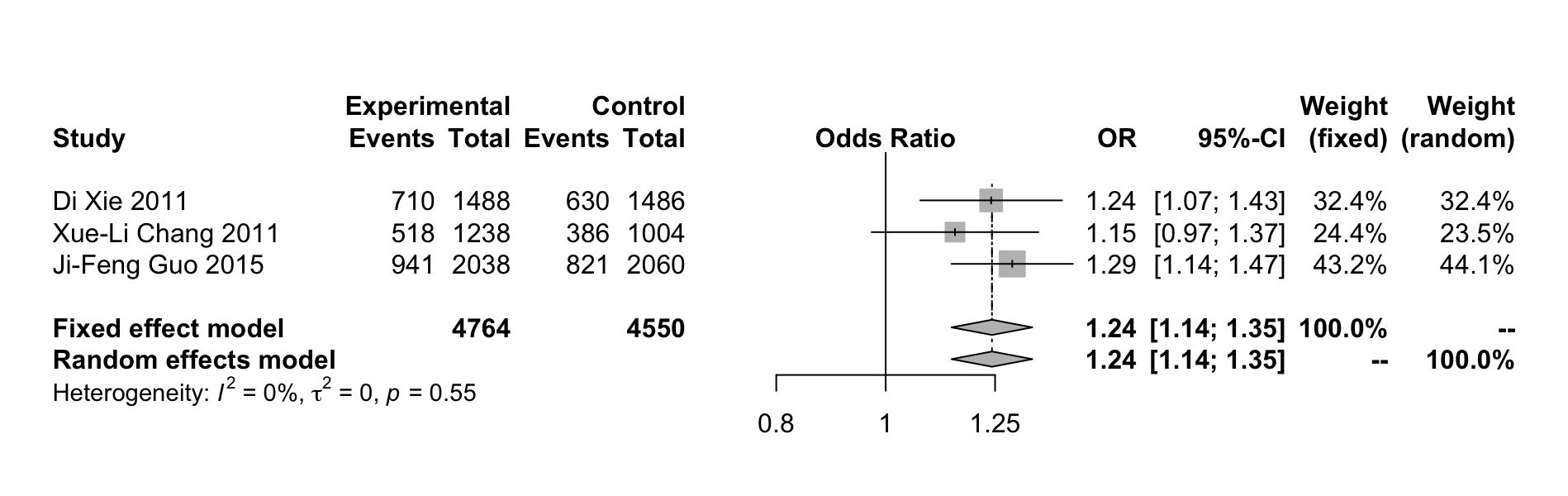


Appendix Figure 3.24 Allele model of *BST1* rs4698412

**Dominant model**: The forest plot and result of dominant model of rs4698412 is shown below. Since there was no heterogeneity observed, results of fixed effect model were adopted. After meta-analysis to 2382 PD patients and 2275 controls, OR was 1.34 (1.19 – 1.52), *p* value: <0.0001.


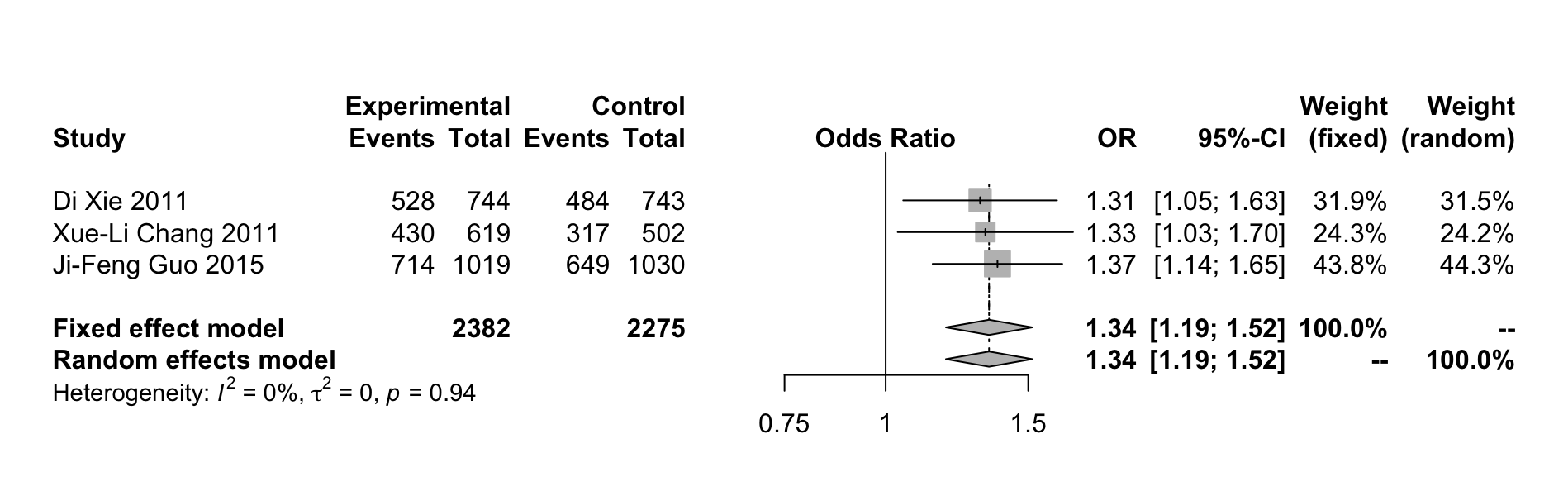


Appendix Figure 3.25 Dominant model of *BST1* rs4698412

**Recessive model**: The forest plot and result of recessive model of rs4698412 is shown below. Since there was no heterogeneity observed, results of fixed effect model were adopted. After meta-analysis to 2382 PD patients and 2275 controls, OR was 1.31 (1.13 – 1.52), *p* value: 0.0004.


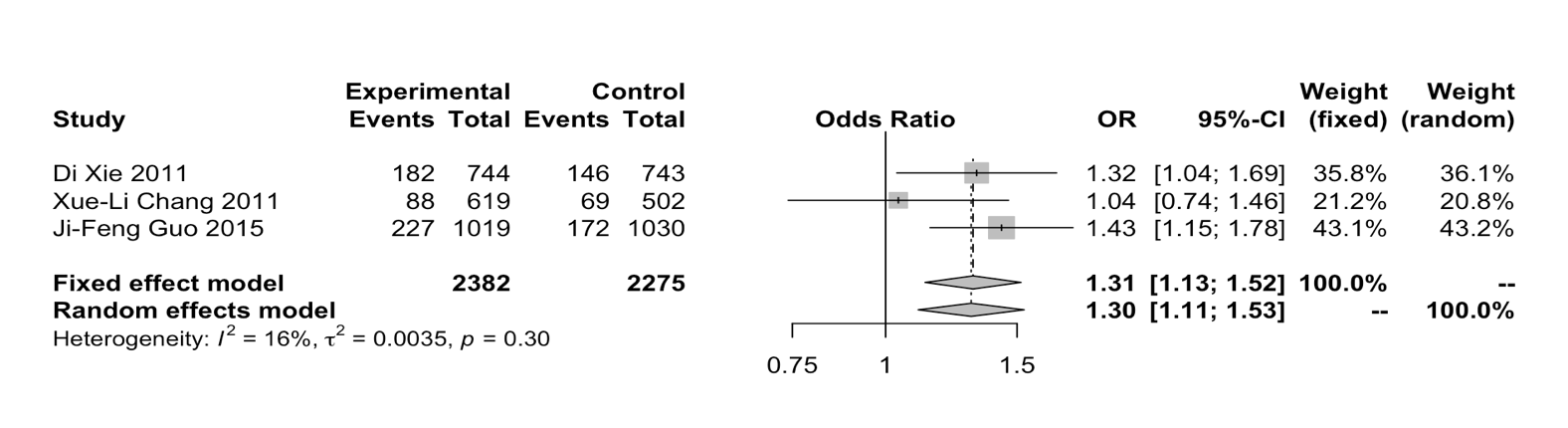


Appendix Figure 3.26 Recessive model of *BST1* rs4698412

**Overdominant model**: The forest plot and result of recessive model of rs4698412 is shown below. Since there was no heterogeneity observed, results of fixed effect model were adopted. After meta-analysis to 2382 PD patients and 2275 controls, OR was 0.91 (0.81 – 1.02), *p* value: 0.105.


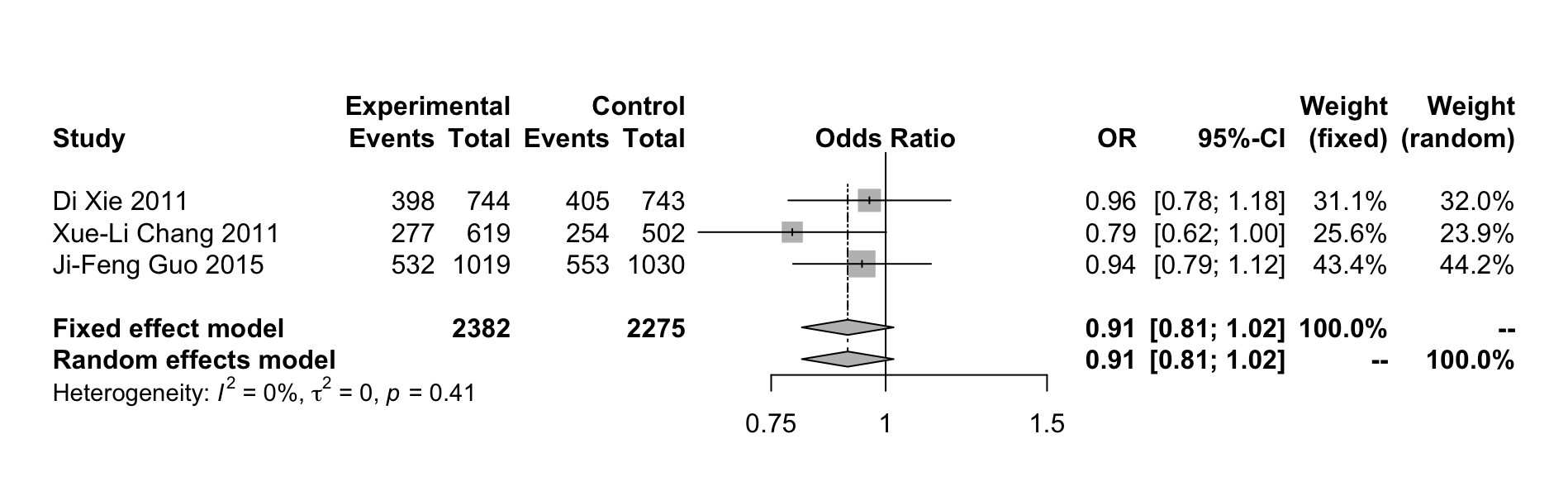


Appendix Figure 3.27 Overdominant model of *BST1* rs4698412

1. **rs11931532, *BST1*, T>C**

| Articles | PD subjects | | | Controls | | | Methods | Location of Population |
| --- | --- | --- | --- | --- | --- | --- | --- | --- |
|  | CC | CT | TT | CC | CT | TT |  |  |
| Xue-Li Chang et al., 2011^a,21^ | 126 | 336 | 158 | 123 | 271 | 110 | MassARRAY | Chengdu, Sichuan Province |
| Ji-Feng Guo et al., 2015^a,22^ | 201 | 576 | 242 | 237 | 550 | 243 | PCR | Changsha, Hunan Province |
| PCR: Polymerase chain reaction; PD: Parkinson’s disease  a: diagnostic criteria: the United Kingdom brain bank criteria ^3^ | | | | | | | | |

**Allele model**: The forest plot and result of allele model of rs11931532 is shown below. We regarded allele C as risk allele. Since there was no heterogeneity observed, results of fixed effect model were adopted. After meta-analysis to 1639 PD patients and 1534 controls, OR was 0.91 (0.82 – 1.00) compared to allele T. *p* value: 0.049.


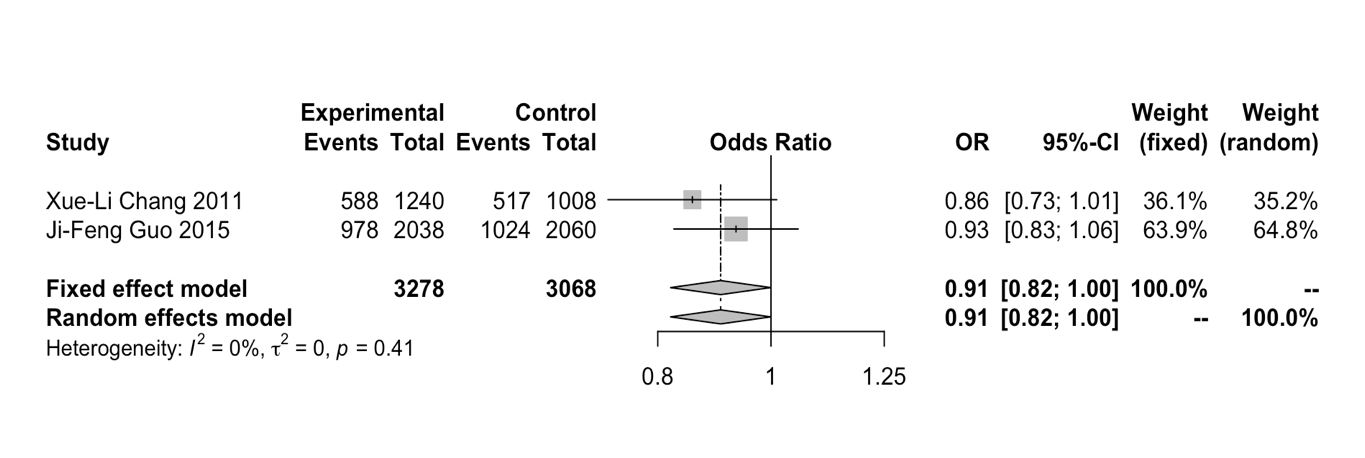


Appendix Figure 3.28 Allele model of *BST1* rs11931532

**Dominant model**: The forest plot and result of dominant model of rs11931532 is shown below. Since there was no heterogeneity observed, results of fixed effect model were adopted. After meta-analysis to 1639 PD patients and 1534 controls, OR was 0.93 (0.79 – 1.09), *p* value: 0.359.


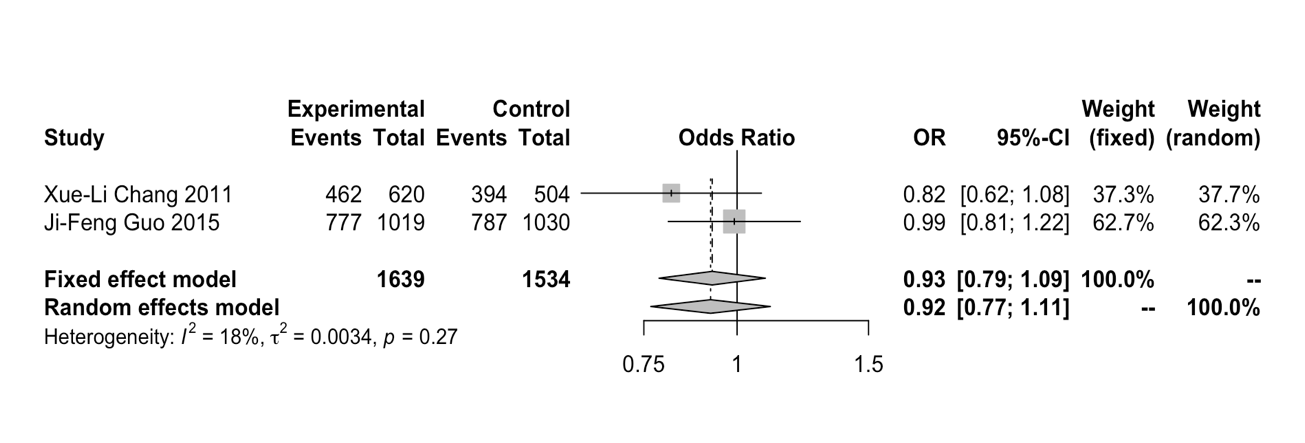


Appendix Figure 3.29 Dominant model of *BST1* rs11931532

**Recessive model**: The forest plot and result of recessive model of rs11931532 is shown below. Since there was no heterogeneity observed, results of fixed effect model were adopted. After meta-analysis to 1639 PD patients and 1534 controls, OR was 0.81 (0.68 – 0.96), *p* value: 0.015.


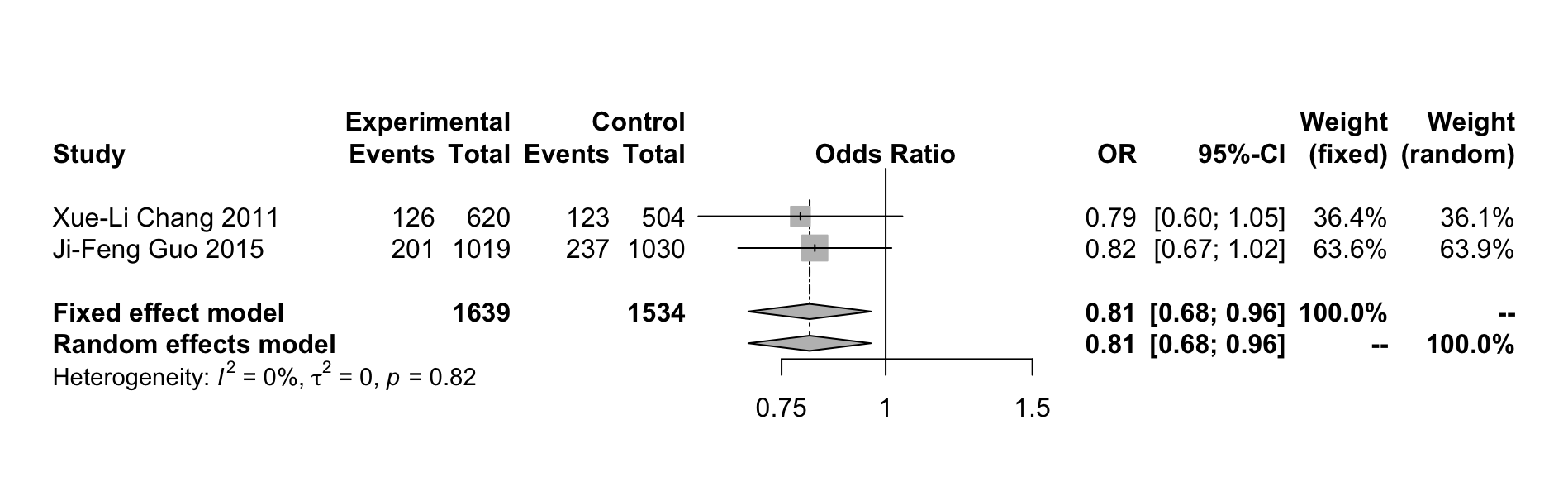


Appendix Figure 3.30 Recessive model of *BST1* rs11931532

**Overdominant model**: The forest plot and result of recessive model of rs11931532 is shown below. Since there was no heterogeneity observed, results of fixed effect model were adopted. After meta-analysis to 1639 PD patients and 1534 controls, OR was 0.92 (0.80 – 1.05), *p* value: 0.219.


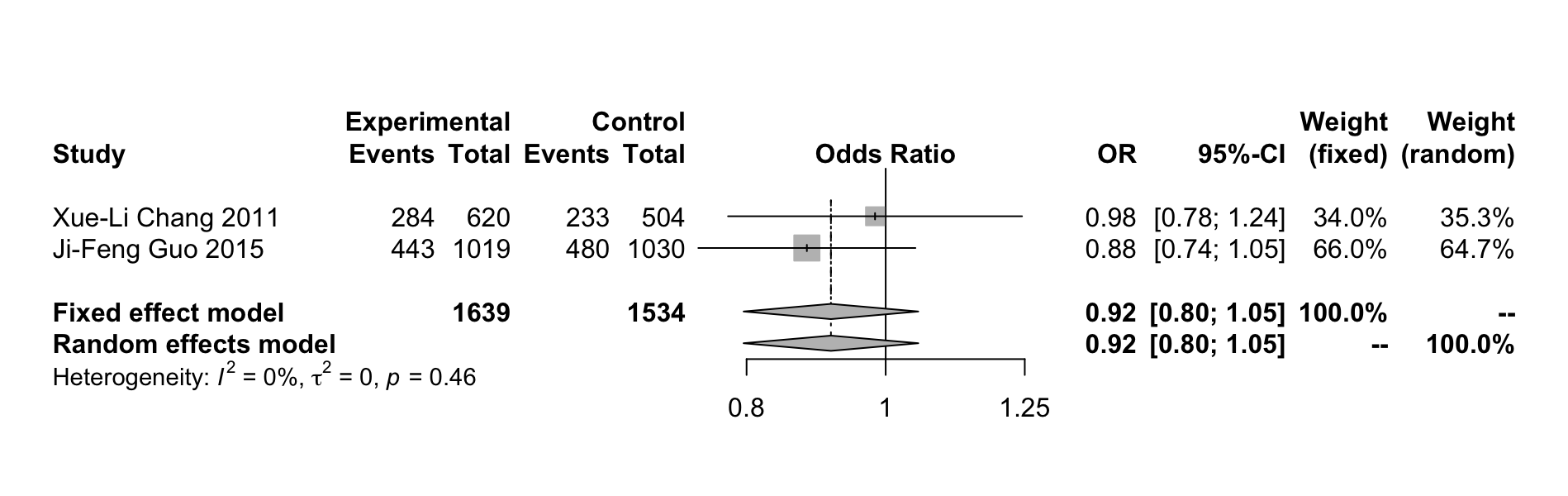


Appendix Figure 3.31 Overdominant model of *BST1* rs11931532

1. **rs10043, *CHCHD2*, c. -9T>G**

| Articles | PD subjects | | | Controls | | | Methods | Location of Population |
| --- | --- | --- | --- | --- | --- | --- | --- | --- |
|  | GG | GT | TT | GG | GT | TT |  |  |
| Qian Lu et al., 2016^a, 23^ | 201 | 42 | 2 | 184 | 35 | 1 | PCR | Changsha, Hunan Province |
| Hongwei Wu et al., 2017^b, 24^ | 148 | 14 | 0 | 83 | 7 | 0 | PCR | Hangzhou, Zhejiang Province |
| Chengyuan Mao et al., 2018^b, 25^ | 1 | 33 | 339 | 1 | 39 | 353 | PCR | Zhengzhou, Henan Province |
| PCR: polymerase chain reaction; PD: Parkinson’s disease  a: diagnosed by neurologists, detailed diagnostic criteria unknown  b: diagnostic criteria: the United Kingdom brain bank criteria ^3^ | | | | | | | | |

**Allele model**: The forest plot and result of allele model of rs10043 is shown below. We regarded allele G as risk allele. Since there was no heterogeneity observed, results of fixed effect model were adopted. After meta-analysis to 780 PD patients and 703 controls, OR was 0.89 (0.66 – 1.21) compared to allele T. *p* value: 0.459.


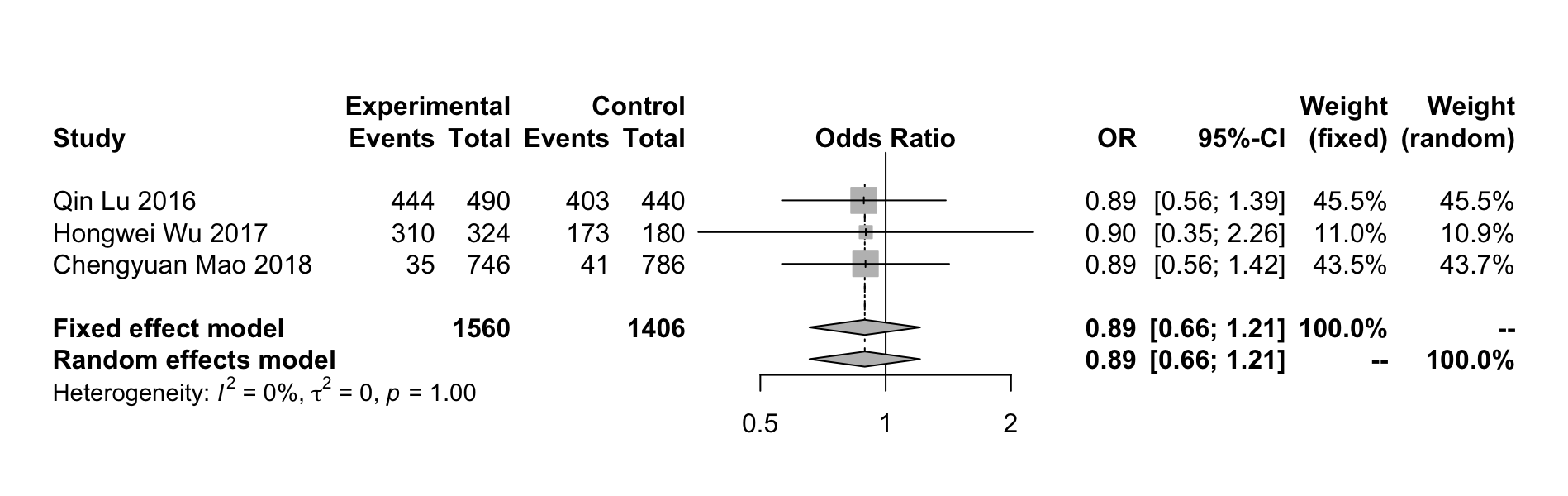


Appendix Figure 3.32 Allele model of *CHCHD2* rs10043

**Dominant model**: The forest plot and result of dominant model of rs10043 is shown below. Since there was no heterogeneity observed, results of fixed effect model were adopted. After meta-analysis to 780 PD patients and 703 controls, OR was 0.87 (0.54 – 1.39), *p* value: 0.557.


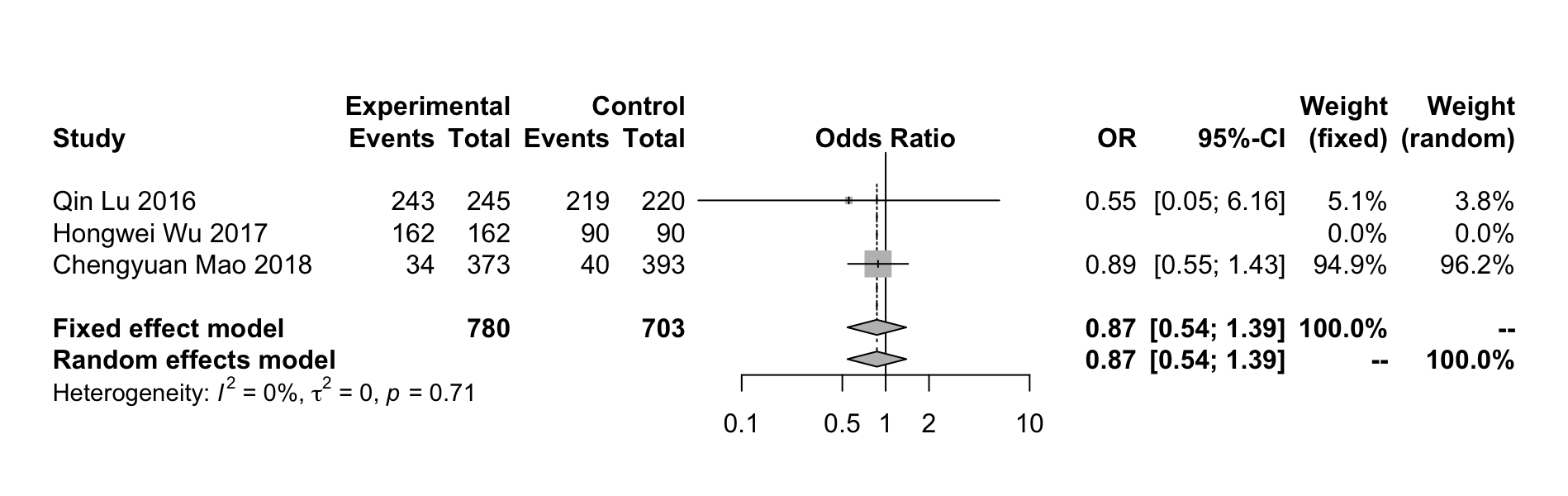


Appendix Figure 3.33 Dominant model of *CHCHD2* rs10043

**Recessive model**: The forest plot and result of recessive model of rs10043 is shown below. Since there was no heterogeneity observed, results of fixed effect model were adopted. After meta-analysis to 780 PD patients and 703 controls, OR was 0.90 (0.59 – 1.37), *p* value: 0.616.


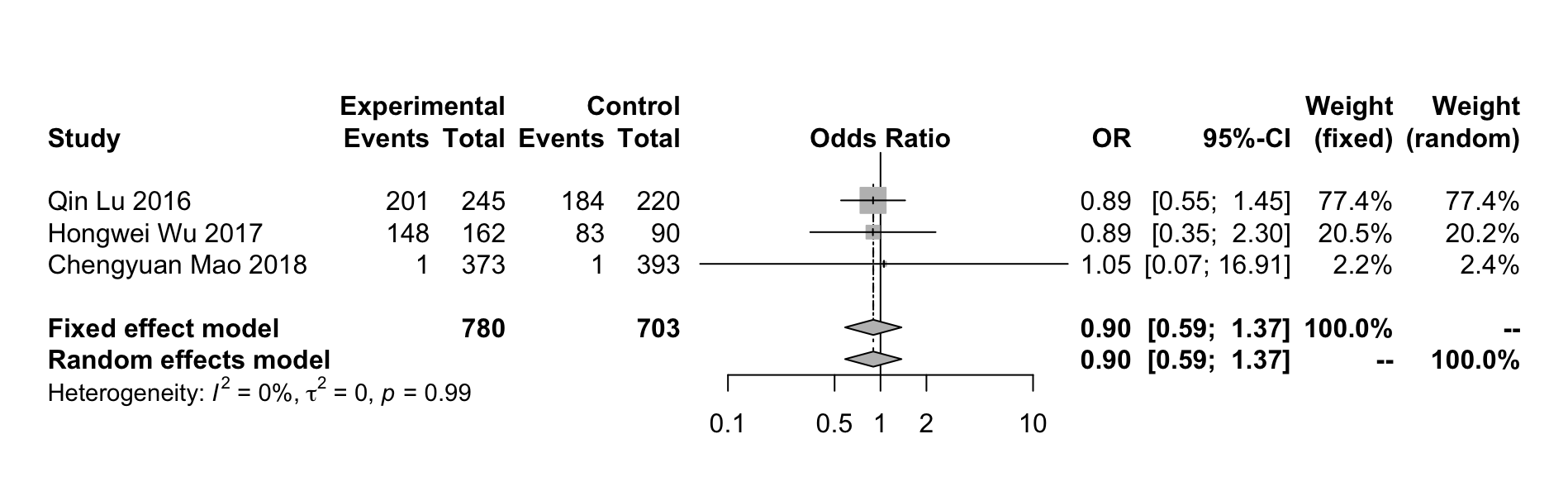


Appendix Figure 3.34 Recessive model of *CHCHD2* rs10043

**Overdominant model**: The forest plot and result of recessive model of rs10043 is shown below. Since there was no heterogeneity observed, results of fixed effect model were adopted. After meta-analysis to 780 PD patients and 703 controls, OR was 1.00 (0.73 – 1.39), *p* value: 0.983.


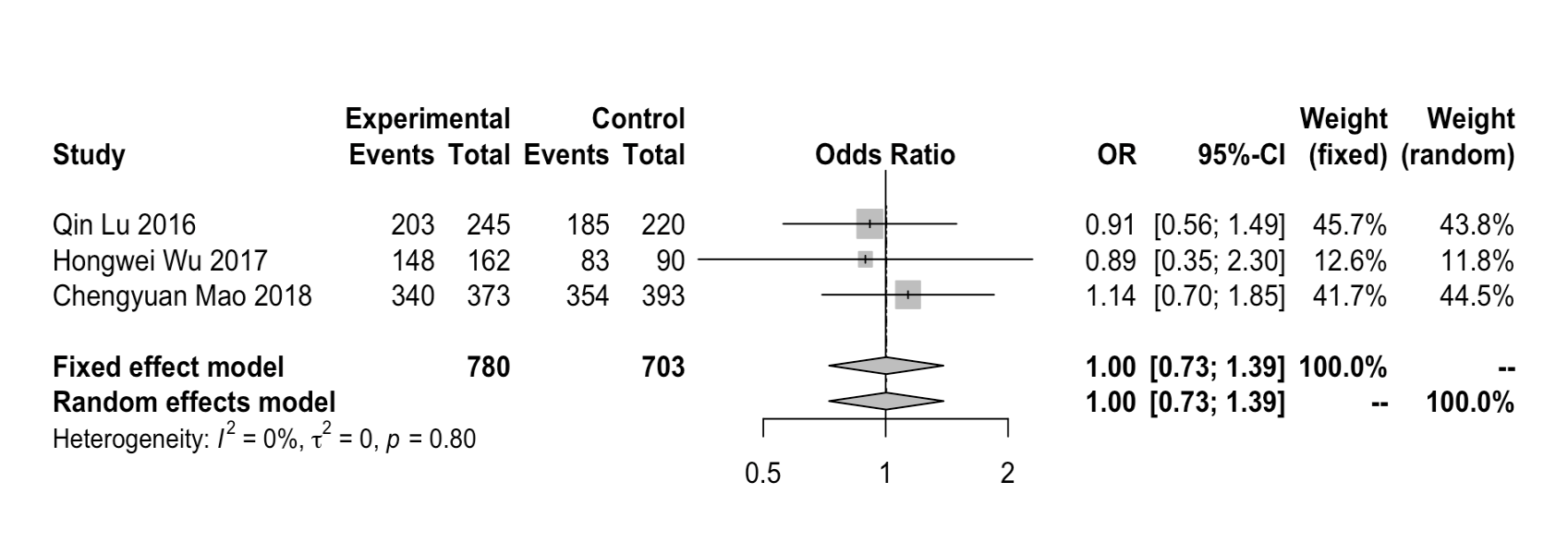


Appendix Figure 3.35 Overdominant model of *CHCHD2* rs10043

However, it is obvious that the distribution presented in the thesis by Chengyuan Mao et al. is totally different compared with others. Though there was no heterogeneity reported, to minimize bias, we excluded his research and performed meta-analysis again.

**Allele model**: The forest plot and result of allele model of rs10043 is shown below. We regarded allele G as risk allele. Since there was no heterogeneity observed, results of fixed effect model were adopted. After meta-analysis to 407 PD patients and 310 controls, OR was 0.89 (0.59 – 1.33) compared to allele T. *p* value: 0.568.


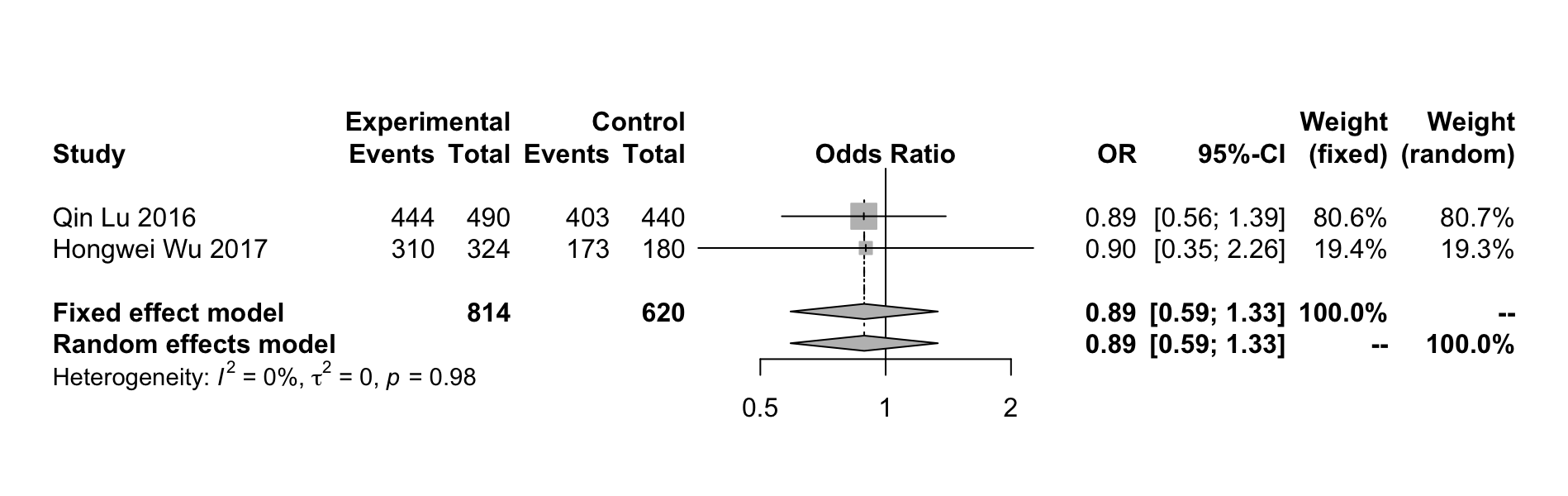


Appendix Figure 3.36 Allele model of *CHCHD2* rs10043

**Dominant model**: This was incalculable due to the distribution of dominant models in the study done by Hongwei Wu et al..

**Recessive model**: The forest plot and result of recessive model of rs10043 is shown below. Since there was no heterogeneity observed, results of fixed effect model were adopted. After meta-analysis to 407 PD patients and 310 controls, OR was 0.90 (0.58 – 1.37), *p* value: 0.608.


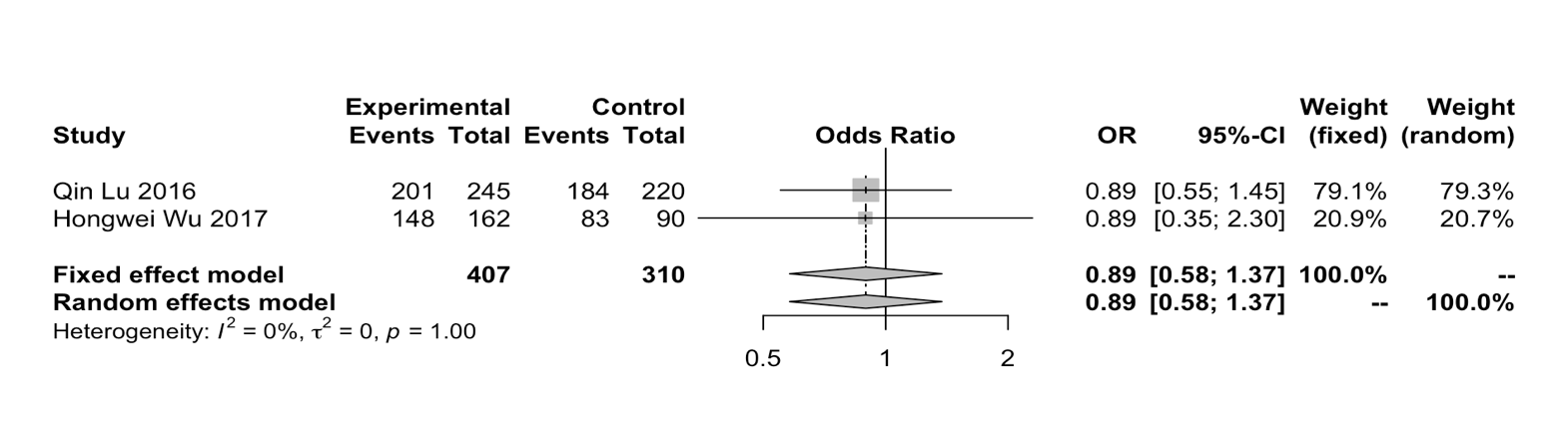


Appendix Figure 3.37 Recessive model of *CHCHD2* rs10043

**Overdominant model**: The forest plot and result of recessive model of rs10043 is shown below. Since there was no heterogeneity observed, results of fixed effect model were adopted. After meta-analysis to 407 PD patients and 310 controls, OR was 0.91 (0.59 – 1.41), *p* value: 0.670.


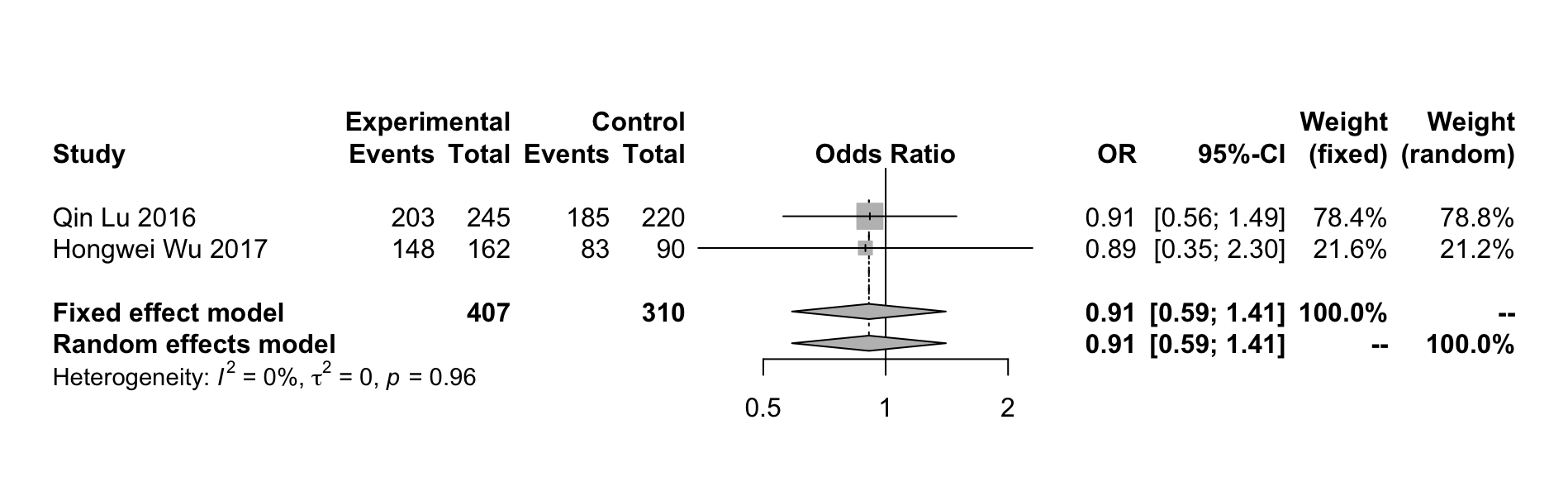


Appendix Figure 3.38 Overdominant model of *CHCHD2* rs10043

1. **rs142444896, *CHCHD2*, Pro2Leu, c.5C>T**

| Articles | PD subjects | | | Controls | | | Methods | Location of Population |
| --- | --- | --- | --- | --- | --- | --- | --- | --- |
|  | CC | CT | TT | CC | CT | TT |  |  |
| Nan-Nan Li et al., 2016^a, 26^ | 1052 | 6 | 0 | 1092 | 3 | 0 | PCR-LDR | Chengdu, Sichuan Province |
| Hongwei Wu et al., 2017^a, 24^ | 355 | 9 | 0 | 382 | 2 | 0 | PCR | Hangzhou, Zhejiang Province |
| Chengyuan Mao et al., 2018^a, 25^ | 161 | 1 | 0 | 90 | 0 | 0 | PCR | Zhengzhou, Henan Province |
| LDR: Ligase detection reaction; PCR: polymerase chain reaction; PD: Parkinson’s disease  a: diagnostic criteria: the United Kingdom brain bank criteria ^3^ | | | | | | | | |

**Allele model**: The forest plot and result of allele model of rs142444896 is shown below. We regarded allele T as risk allele. Since there was no heterogeneity observed, results of fixed effect model were adopted. After meta-analysis to 1584 PD patients and 1569 controls, OR was 2.98 (1.13 – 7.82) compared to allele C. *p* value: 0.027.


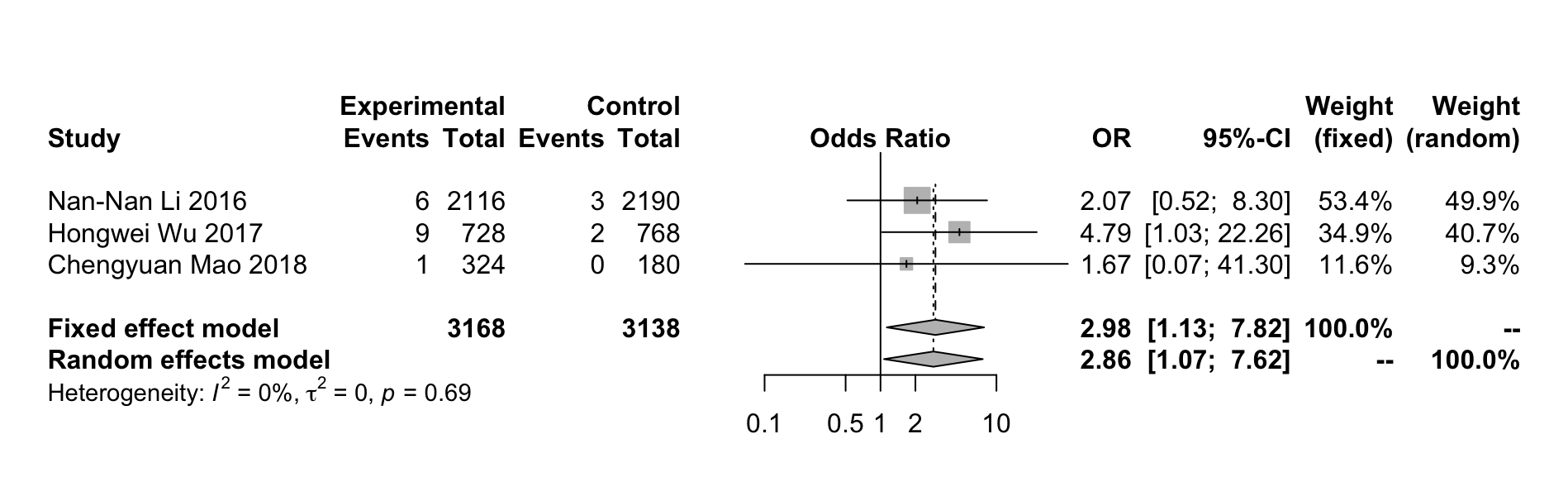


Appendix Figure 3.39 Allele model of *CHCHD2* rs142444896

**Dominant model**: The forest plot and result of dominant model of rs142444896 is shown below. Since there was no heterogeneity observed, results of fixed effect model were adopted. After meta-analysis to 1584 PD patients and 1569 controls, OR was 2.99 (1.14 – 7.87), *p* value: 0.026.


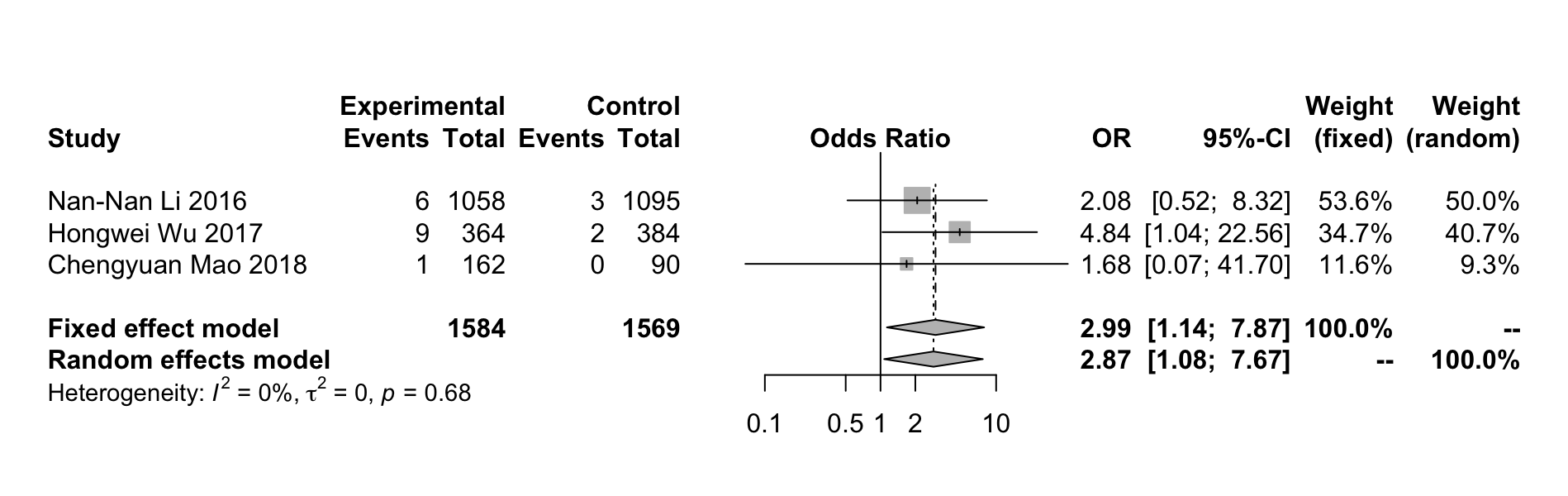


Appendix Figure 3.40 Dominant model of *CHCHD2* rs142444896

**Recessive model**: It was not applied because of no TT homozygote.

**Overdominant model**: The forest plot and result of recessive model of rs142444896 is shown below. Since there was no heterogeneity observed, results of fixed effect model were adopted. After meta-analysis to 1584 PD patients and 1569 controls, OR was 0.33 (0.13 – 0.88), *p* value: 0.026.


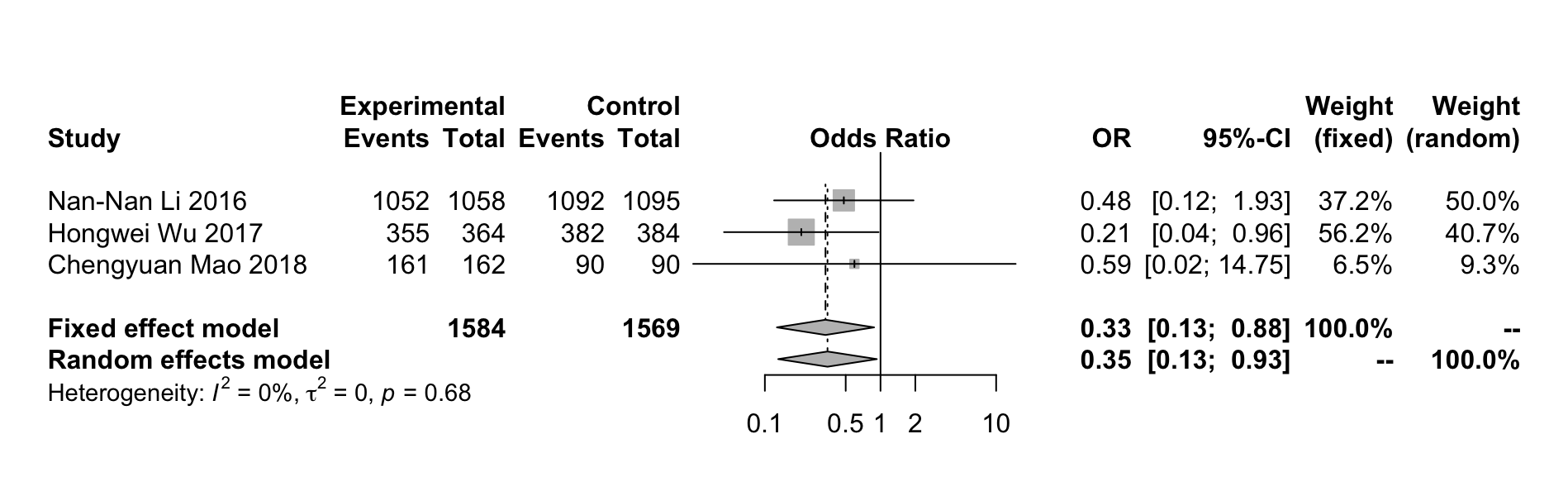


Appendix Figure 3.41 Overdominant model of *CHCHD2* rs142444896

1. **rs200226056, *CHCHD2*, c.-11G>A**

| Articles | PD subjects | | | Controls | | | Methods | Location of Population |
| --- | --- | --- | --- | --- | --- | --- | --- | --- |
|  | AA | AG | GG | AA | AG | GG |  |  |
| Qian Lu et al., 2016^a, 23^ | 0 | 5 | 240 | 0 | 4 | 216 | PCR | Changsha, Hunan Province |
| Chengyuan Mao et al., 2018^b, 25^ | 0 | 2 | 362 | 0 | 8 | 376 | PCR | Zhengzhou, Henan Province |
| PCR: polymerase chain reaction; PD: Parkinson’s disease  a: diagnosed by neurologists, detailed diagnostic criteria unknown  b: diagnostic criteria: the United Kingdom brain bank criteria ^3^ | | | | | | | | |

**Allele model**: The forest plot and result of allele model of rs200226056 is shown below. We regarded allele A as risk allele. Since there was no heterogeneity observed (*I*^2^ 49.7%), results of fixed effect model were adopted. After meta-analysis to 609 PD patients and 604 controls, OR was 0.56 (0.22 -1.45) compared to allele G. *p* value: 0.232.


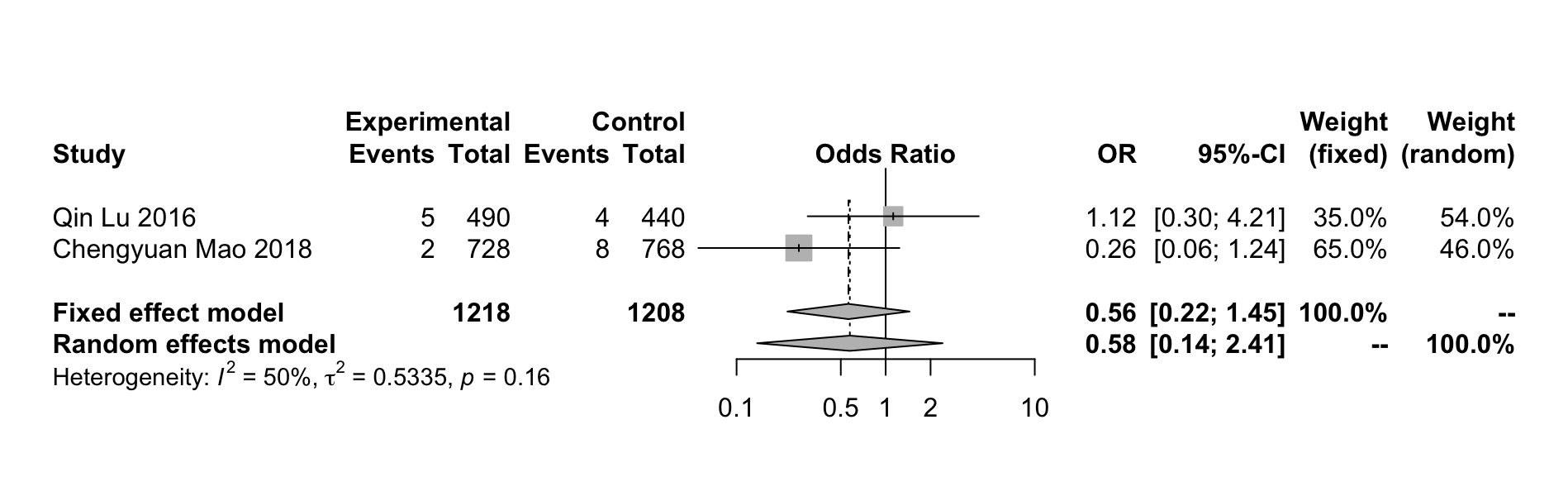


Appendix Figure 3.42 Allele model of *CHCHD2* rs200226056

**Dominant model**: The forest plot and result of dominant model of rs200226056 is shown below. Since there was no heterogeneity observed (*I*^2^ 49.9%), results of fixed effect model were adopted. After meta-analysis to 609 PD patients and 604 controls, OR was 0.56 (0.22 – 1.44), *p* value: 0.231.


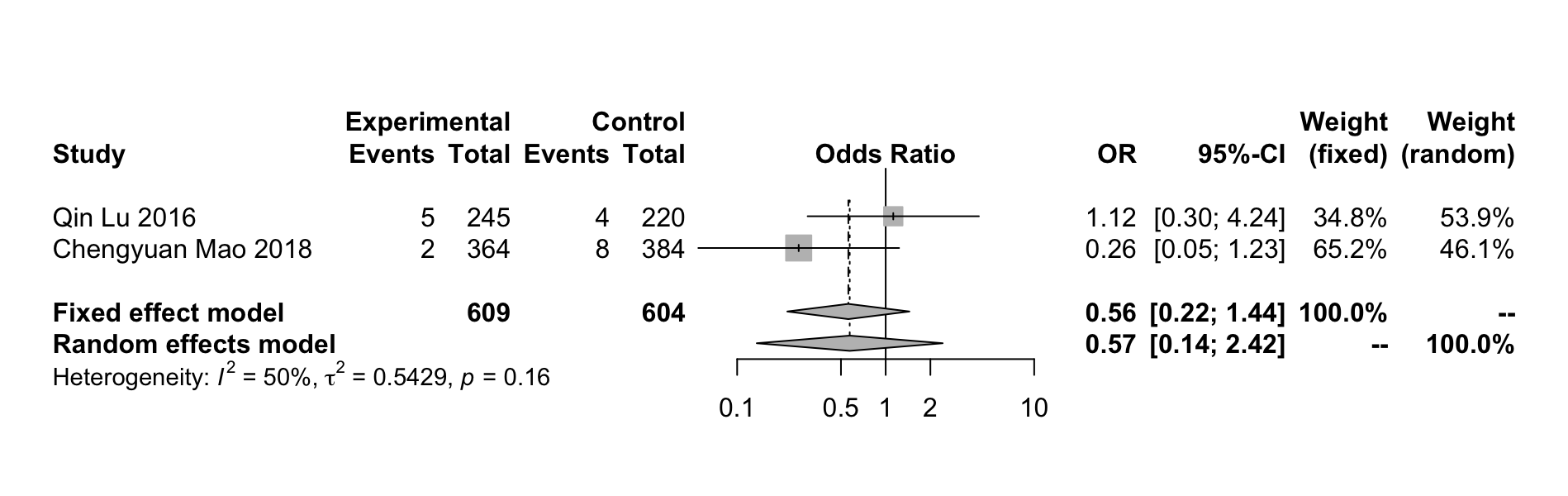


Appendix Figure 3.43 Dominant model of *CHCHD2* rs200226056

**Recessive model**: It was not applied because of no AA homozygote.

**Overdominant model**: The forest plot and result of recessive model of rs200226056 is shown below. Since there was no heterogeneity observed (*I*^2^ 49.9%), results of fixed effect model were adopted. After meta-analysis to 609 PD patients and 604 controls, OR was 1.78 (0.69 – 4.60), *p* value: 0.231.


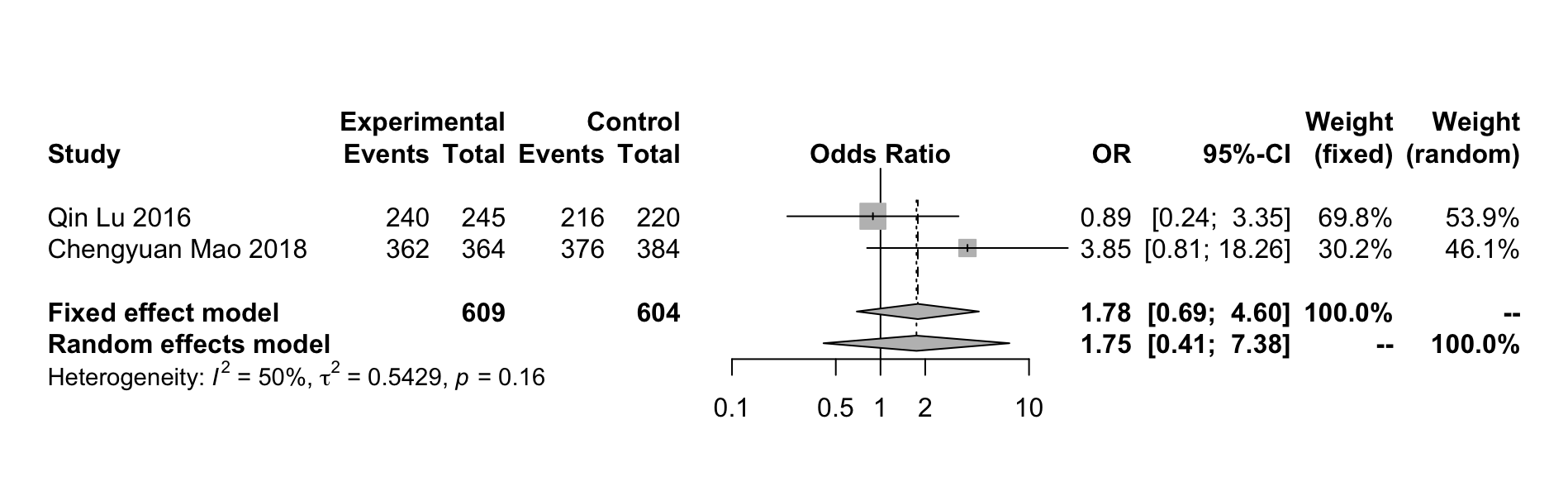


Appendix Figure 3.44 Overdominant model of *CHCHD2* rs200226056

1. **rs816407, *CHCHD2*, c.-34C>A**

| Articles | PD subjects | | | Controls | | | Methods | Location of Population |
| --- | --- | --- | --- | --- | --- | --- | --- | --- |
|  | AA | AC | CC | AA | AC | CC |  |  |
| Qian Lu et al., 2016^a, 23^ | 201 | 42 | 2 | 184 | 35 | 1 | PCR | Changsha, Hunan Province |
| Hongwei Wu et al., 2017^b, 24^ | 148 | 14 | 0 | 83 | 7 | 0 | PCR | Hangzhou, Zhejiang Province |
| PCR: polymerase chain reaction; PD: Parkinson’s disease  a: diagnosed by neurologists, detailed diagnostic criteria unknown  b: diagnostic criteria: the United Kingdom brain bank criteria^3^ | | | | | | | | |

**Allele model**: The forest plot and result of allele model of rs816407 is shown below. We regarded allele A as risk allele. Since there was no heterogeneity observed, results of fixed effect model were adopted. After meta-analysis to 407 PD patients and 310 controls, OR was 0.89 (0.59 – 1.33) compared to allele C. *p* value: 0.568.


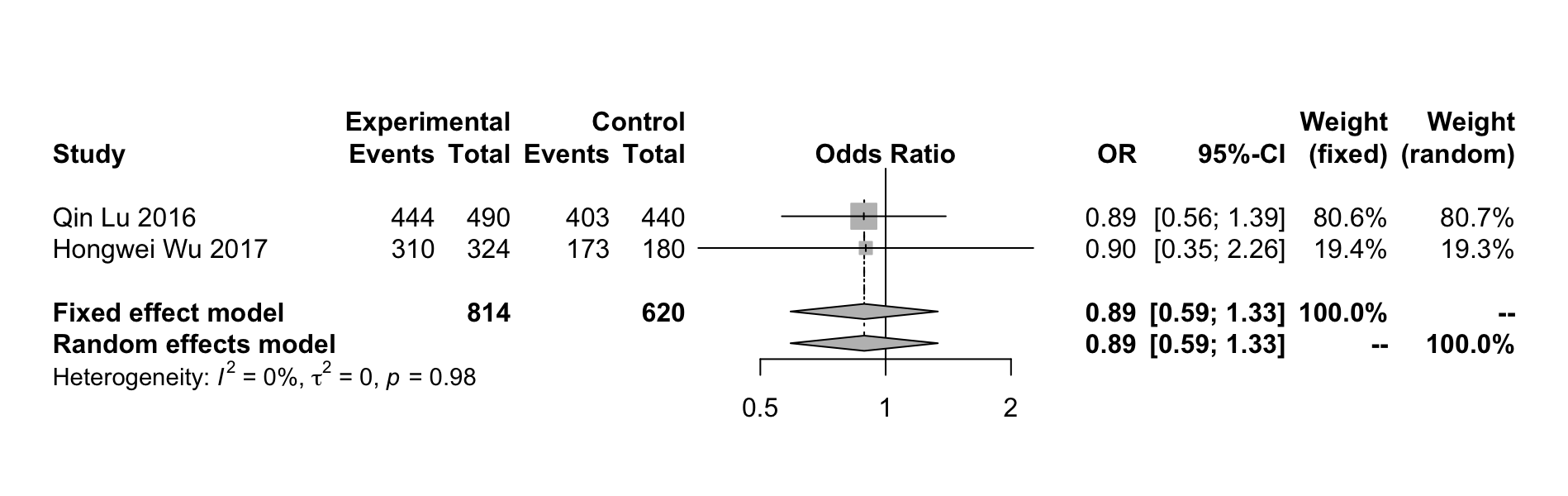


Appendix Figure 3.45 Allele model of *CHCHD2* rs816407

**Dominant model**: It was not applied because of no CC homozygote observed in Hongwei Wu’s study.

**Recessive model**: The forest plot and result of recessive model of rs816407 is shown below. Since there was no heterogeneity observed, results of fixed effect model were adopted. After meta-analysis to 407 PD patients and 310 controls, OR was 0.89 (0.58 – 1.37), *p* value: 0.608.


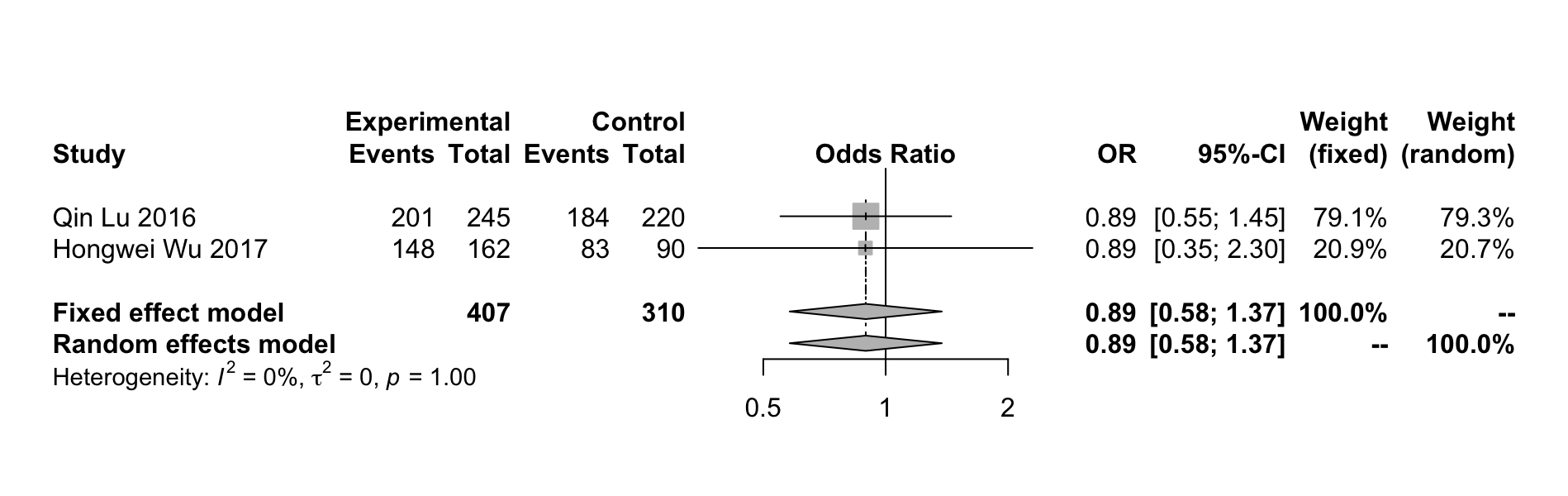


Appendix Figure 3.46 Recessive model of *CHCHD2* rs816407

**Overdominant model**: The forest plot and result of recessive model of rs816407 is shown below. Since there was no heterogeneity observed, results of fixed effect model were adopted. After meta-analysis to 407 PD patients and 310 controls, OR was 0.91 (0.59 – 1.41), *p* value: 0.670.


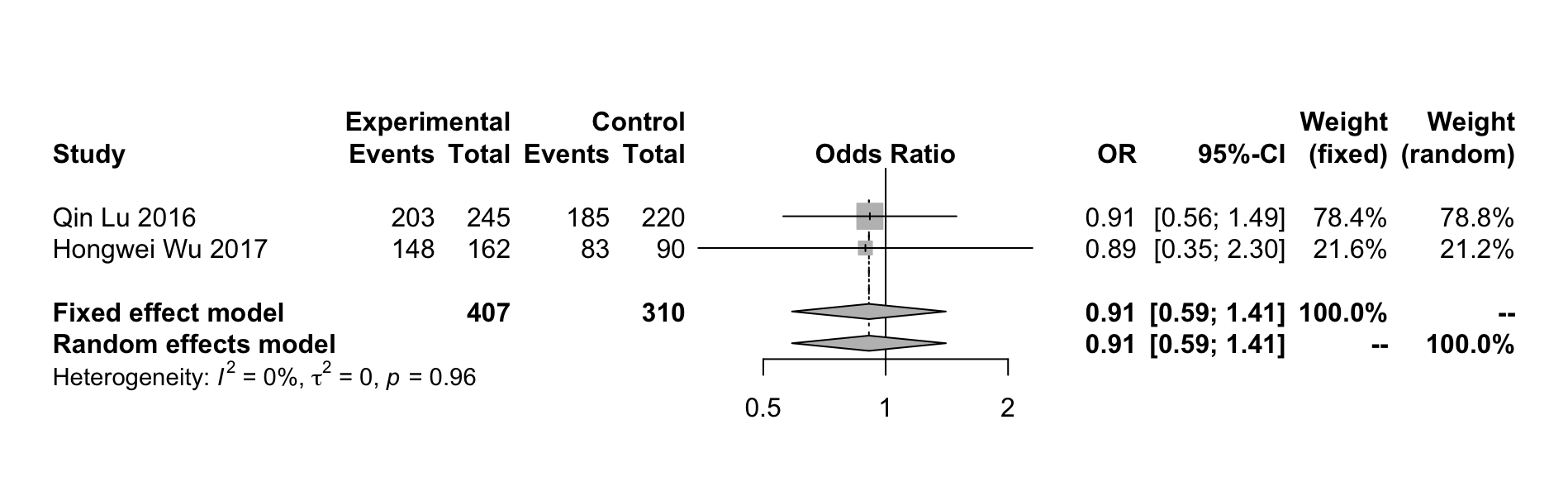


Appendix Figure 3.47 Overdominant model of *CHCHD2* rs816407

1. **rs8406, *CHCHD2*, c.456+125G>A**

| Articles | PD subjects | | | Controls | | | Methods | Location of Population |
| --- | --- | --- | --- | --- | --- | --- | --- | --- |
|  | AA | AG | GG | AA | AG | GG |  |  |
| Hongwei Wu et al., 2017^a, 24^ | 0 | 15 | 147 | 0 | 7 | 83 | PCR | Hangzhou, Zhejiang Province |
| Chengyuan Mao et al., 2018^a, 25^ | 1 | 40 | 323 | 1 | 31 | 352 | PCR | Zhengzhou, Henan Province |
| PCR: Polymerase chain reaction; PD: Parkinson’s disease  a: diagnostic criteria: the United Kingdom brain bank criteria ^3^ | | | | | | | | |

**Allele model**: The forest plot and result of allele model of rs8406 is shown below. We regarded allele A as risk allele. Since there was no heterogeneity observed, results of fixed effect model were adopted. After meta-analysis to 526 PD patients and 474 controls, OR was 1.33 (0.87 – 2.01) compared to allele G. *p* value: 0.183.


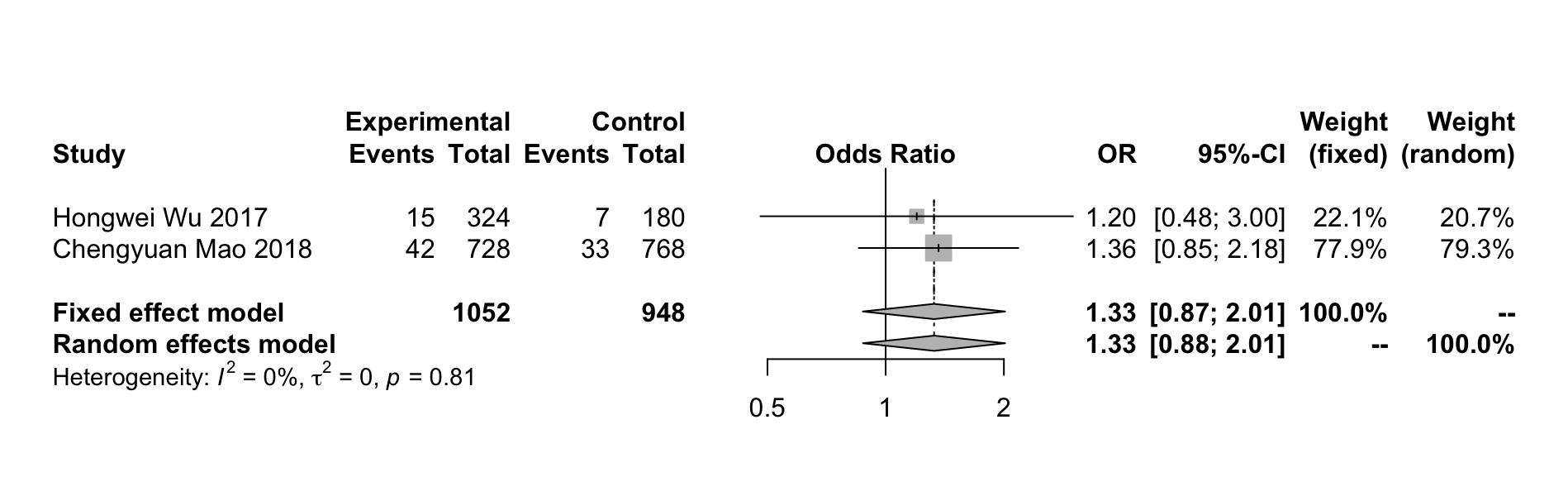


Appendix Figure 3.48 Allele model of *CHCHD2* rs8406

**Dominant model**: The forest plot and result of dominant model of rs8406 is shown below. Since there was no heterogeneity observed, results of fixed effect model were adopted. After meta-analysis to 526 PD patients and 474 controls, OR was 1.35 (0.88 – 2.09), *p* value: 0.170.


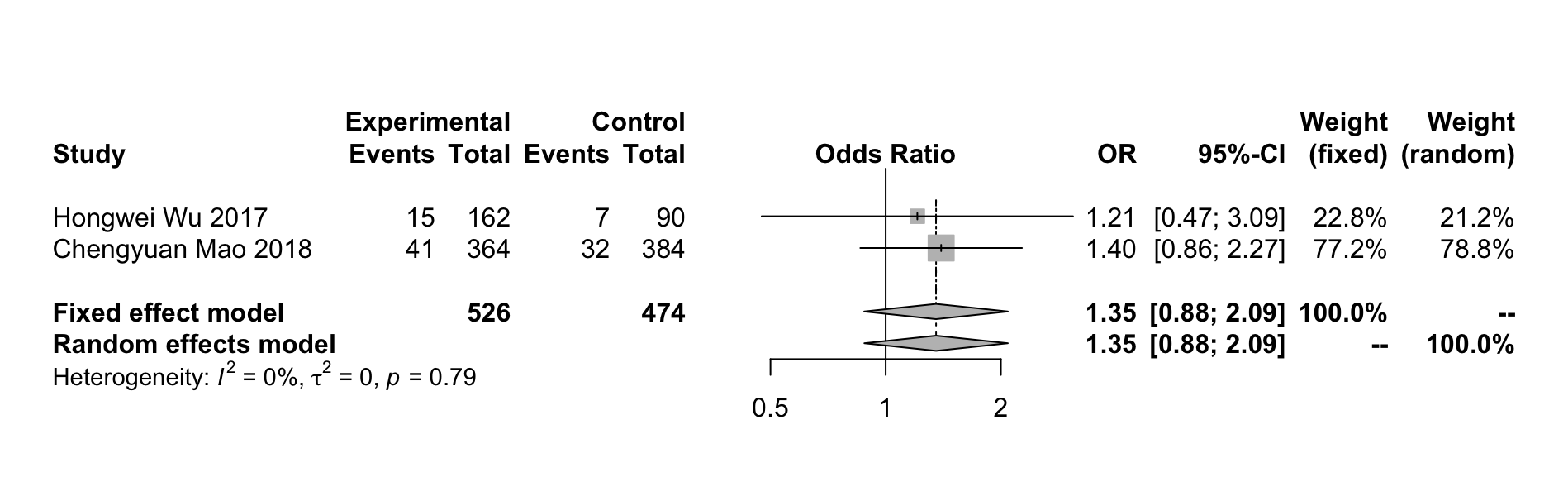


Appendix Figure 3.49 Dominant model of *CHCHD2* rs8406

**Recessive model**: It was not applied because of no AA homozygote observed in Hongwei Wu’s study.

**Overdominant model**: The forest plot and result of recessive model of rs8406 is shown below. Since there was no heterogeneity observed, results of fixed effect model were adopted. After meta-analysis to 526 PD patients and 474 controls, OR was 0.74 (0.48 – 1.14), *p* value: 0.167.


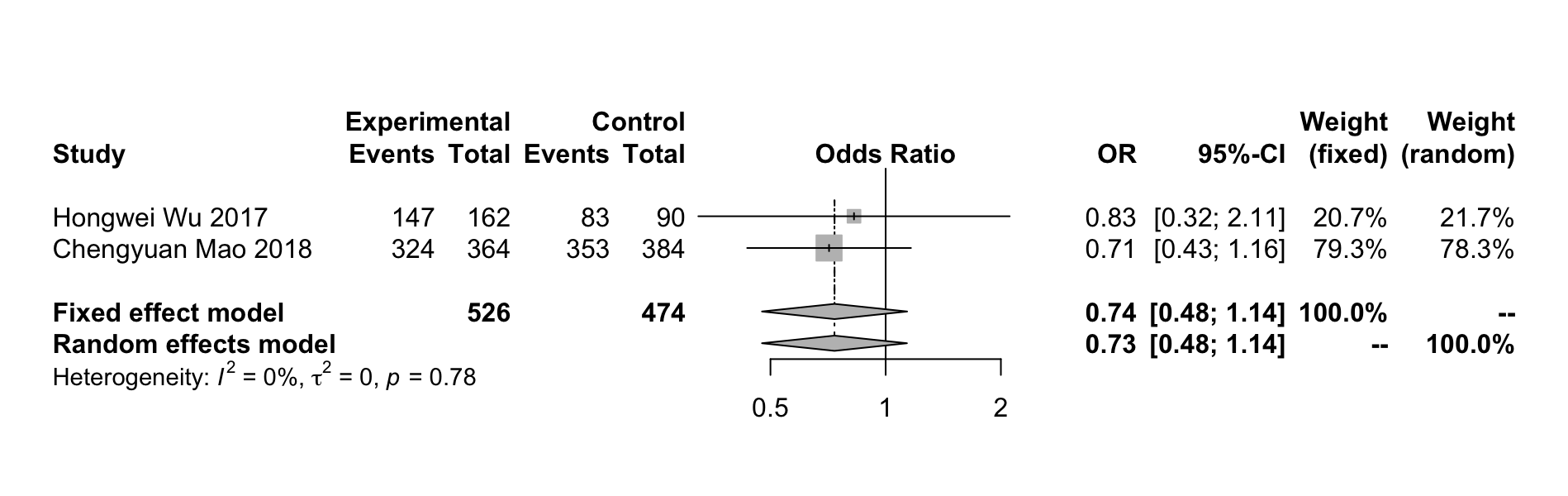


Appendix Figure 3.50 Overdominant model of *CHCHD2* rs8406

1. **rs4680, *COMT*, Val158Met, c.472G>A**

| Articles | PD subjects | | | Controls | | | Methods | Location of Population |
| --- | --- | --- | --- | --- | --- | --- | --- | --- |
|  | AA | AG | GG | AA | AG | GG |  |  |
| T Xie et al., 1997^a,^ ^27^ | 5 | 21 | 44 | 6 | 19 | 37 | PCR | Hong Kong |
| Ming Shao et al., 2001^b, 28^ | 15 | 41 | 84 | 7 | 62 | 75 | PCR-RFLP | Guangzhou, Guangdong Province |
| R.M. Wu et al., 2001^c,^ ^29^ | 18 | 79 | 125 | 12 | 62 | 117 | PCR-RFLP | Taiwan |
| Ling Xu et al., 2002^d, 30^ | 10 | 54 | 80 | 10 | 86 | 105 | PCR-RFLP | Shanghai |
| Xiaoping Zhao et al. 2003^d,^ ^31^ | 10 | 54 | 80 | 9 | 81 | 98 | PCR-RFLP | Shanghai |
| Dan Qi et al., 2013^e,^ ^32^ | 5 | 26 | 59 | 16 | 34 | 45 | PCR-RFLP | Harbin, Heilongjiang Province |
| Caiyou et al., 2015^f,*, 19^ | 4 | 38 | 70 | 7 | 56 | 93 | PCR-RFLP | Nanning, Guangxi |
| Youwen Zhang et al., 2015^g, 33^ | 13 | 173 | 251 | 29 | 210 | 291 | PCR-LDR | Guangzhou, Guangdong Province |
| Qin Xiao et al., 2017^g,34^ | 8 | 56 | 79 | 9 | 57 | 91 | PCR | Shanghai |
| LDR: ligase detection reaction; PCR: polymerase chain reaction; PD: Parkinson’s disease; RFLP: restricted fragment length polymorphism  a: Diagnostic criteria: the presence of three following features: resting tremor, rigidity, bradykinesia, postural instability, gait disturbance and assessed by two neurologists  b: diagnostic criteria: brought up on the first extrapyramidal disease conference in China.^35^  c: diagnostic criteria: included the presence of two of the three cardinal signs (tremor at rest, bradykinesia, and rigidity), improvement of symptoms with L-dopa therapy, and no evidence of secondary parkinsonism caused by other neurological diseases or known drugs or toxins, or of atypical parkinsonism  d: diagnostic criteria: brought up by the second extrapyramidal disease conference in China.  e: brought up by CMDS^36^  f: diagnosed by neurologists  g: diagnostic criteria: the United Kingdom brain bank criteria ^3^  *: focused on Zhuang ethinicity. This study was not included due to different population ethnicity | | | | | | | | |

**Allele model**: The forest plot and result of allele model of rs4680 is shown below. We regarded allele A as risk allele. Since there was no heterogeneity observed, results of fixed effect model were adopted. After meta-analysis to 1502 PD patients and 1724 controls, OR was 0.92 (0.82 – 1.03) compared to allele G. *p* value: 0.151.


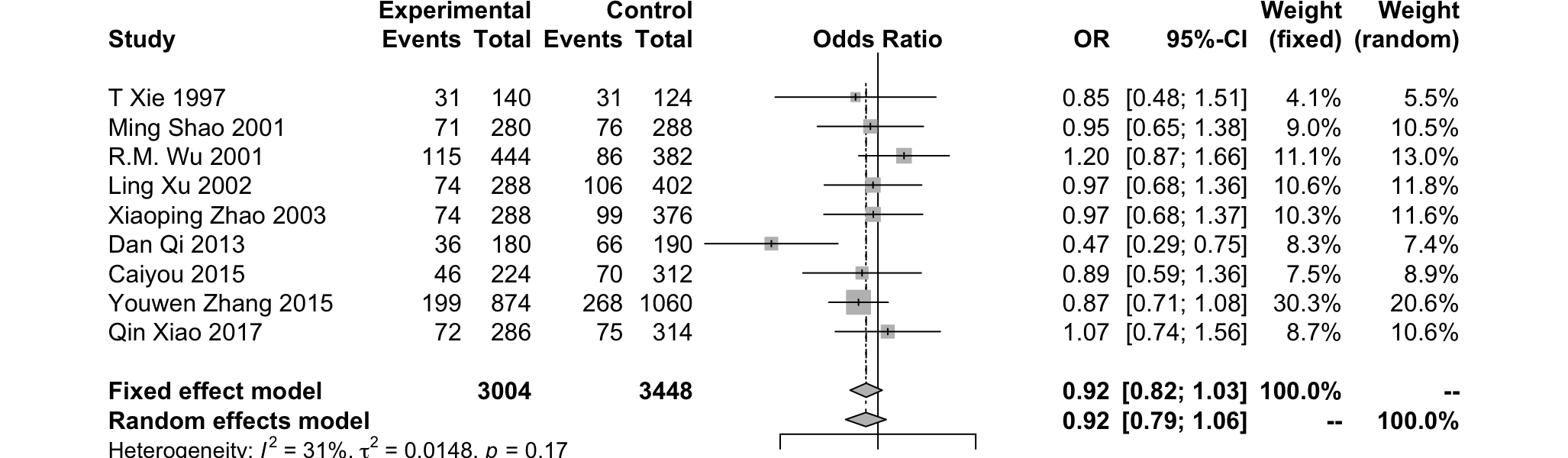


Appendix Figure 3.51 Allele model of *COMT* rs4680

**Dominant model**: The forest plot and result of dominant model of rs4680 is shown below. Since there was no heterogeneity observed, results of fixed effect model were adopted. After meta-analysis to 1502 PD patients and 1724 controls, OR was 0.90 (0.78 – 1.03), *p* value: 0.125.


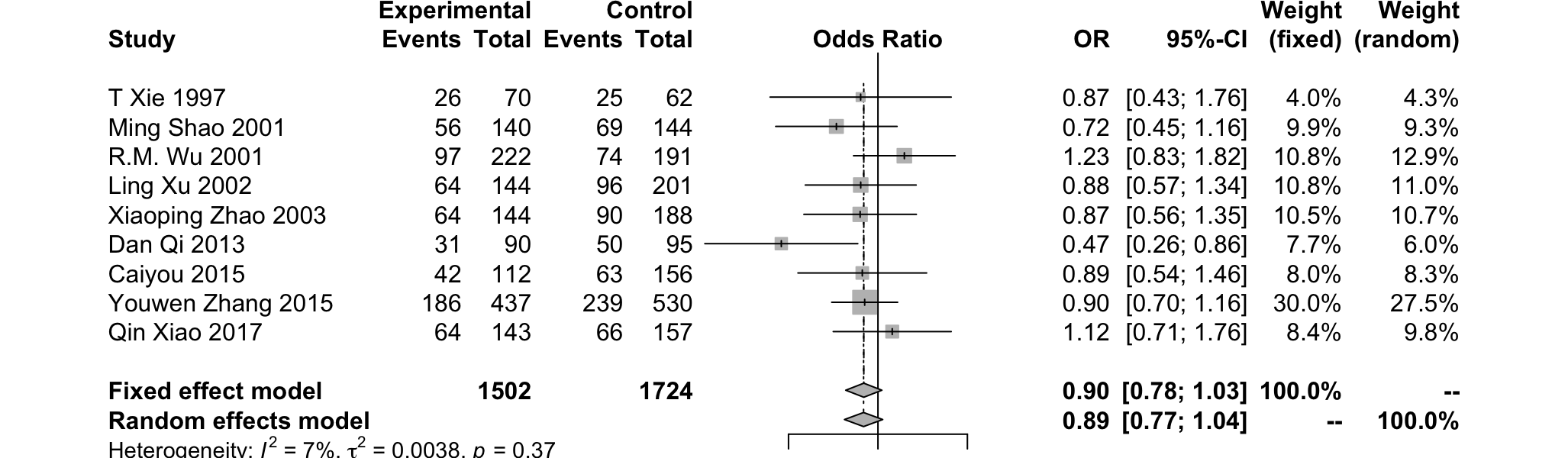


Appendix Figure 3.52 Dominant model of *COMT* rs4680

**Recessive model**: The forest plot and result of recessive model of rs4680 is shown below. Since there was no heterogeneity observed, results of fixed effect model were adopted. After meta-analysis to 1502 PD patients and 1724 controls, OR was 0.93 (0.69 – 1.25), *p* value: 0.623.


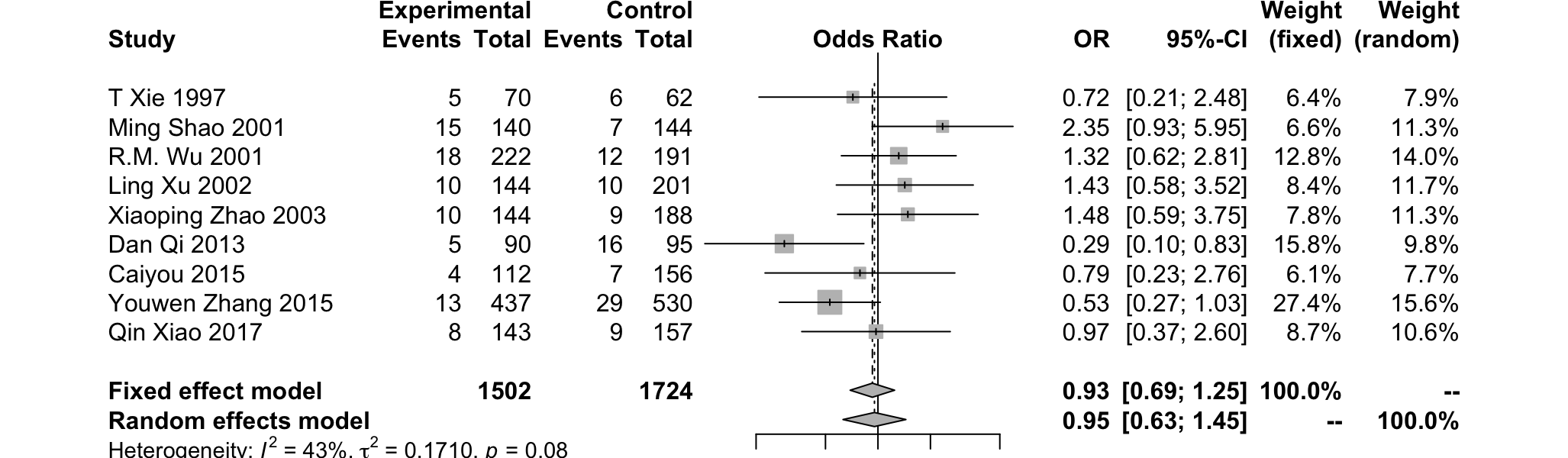


Appendix Figure 3.53 Recessive model of *COMT* rs4680

**Overdominant model**: The forest plot and result of recessive model of rs4680 is shown below. Since there was no heterogeneity observed, results of fixed effect model were adopted. After meta-analysis to 1502 PD patients and 1724 controls, OR was 1.10 (0.95 – 1.27), *p* value: 0.183.


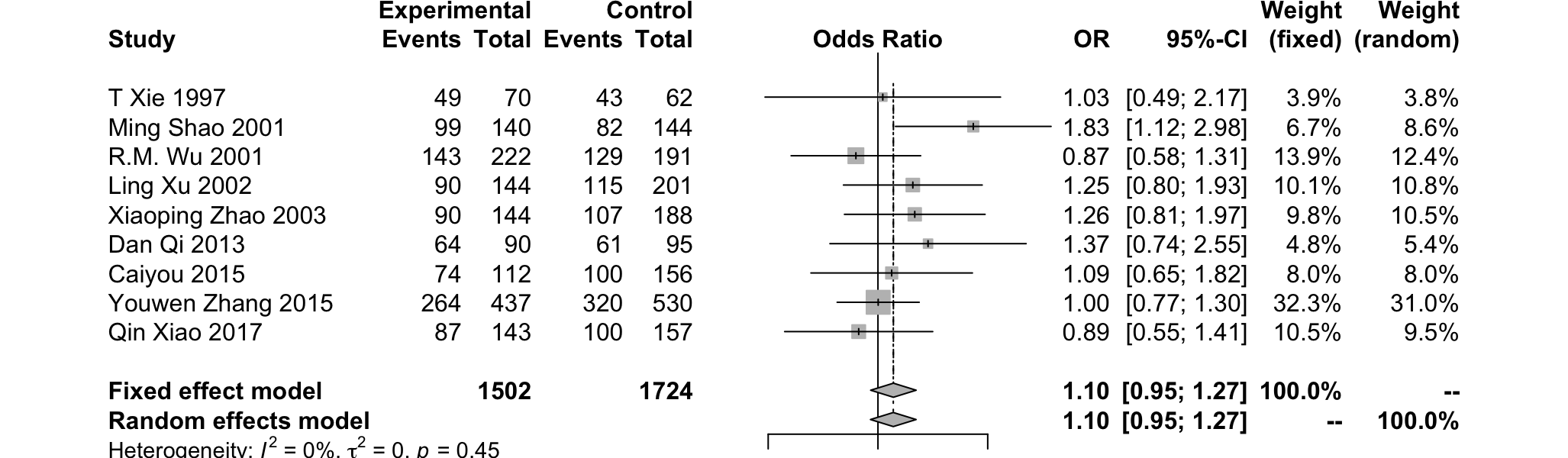


Appendix Figure 3.54 Overdominant model of *COMT* rs4680

1. **rs4633, *COMT*, His62=, c.186C>T**

| Articles | PD subjects | | | Controls | | | Methods | Location of Population |
| --- | --- | --- | --- | --- | --- | --- | --- | --- |
|  | CC | CT | TT | CC | CT | TT |  |  |
| Yanping Dai et al., 2012^a, 37^ | 32 | 23 | 6 | 23 | 25 | 6 | PCR | Harbin, Heilongjiang Province |
| Bowen Yin et al., 2014^a, 38^ | 45 | 45 | 7 | 62 | 37 | 3 | PCR | Harbin, Heilongjiang Province |
| Qin Xiao et al., 2017^a,34^ | 78 | 56 | 9 | 89 | 57 | 11 | PCR | Shanghai |
| PCR: polymerase chain reaction; PD: Parkinson’s disease  a: diagnostic criteria: the United Kingdom brain bank criteria ^3^ | | | | | | | | |

**Allele model**: The forest plot and result of allele model of rs4633 is shown below. We regarded allele T as risk allele. Since there was heterogeneity observed (*I*^2^ 56%), results of random effect model were adopted. After meta-analysis to 301 PD patients and 313 controls, OR was 1.11 (0.75 – 1.65) compared to allele C. *p* value: 0.590.


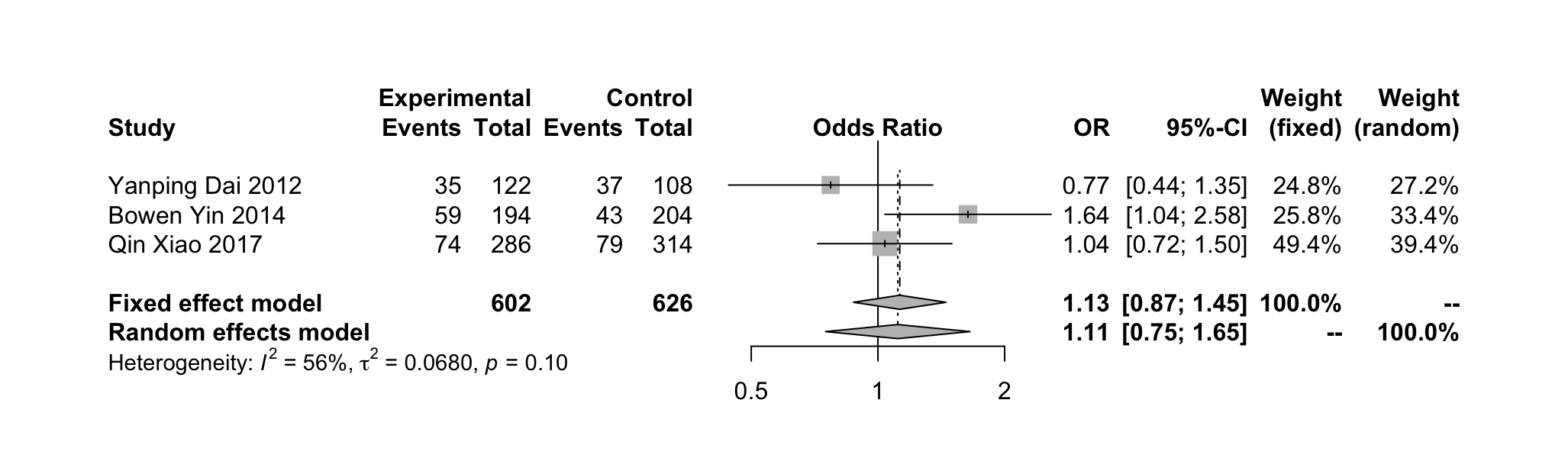


Appendix Figure 3.55 Allele model of *COMT* rs4633

**Dominant model**: The forest plot and result of dominant model of rs4633 is shown below. Since there was heterogeneity observed (*I*^2^ 55%), results of random effect model were adopted. After meta-analysis to 301 PD patients and 313 controls, OR was 1.14 (0.69 – 1.87), *p* value: 0.606.


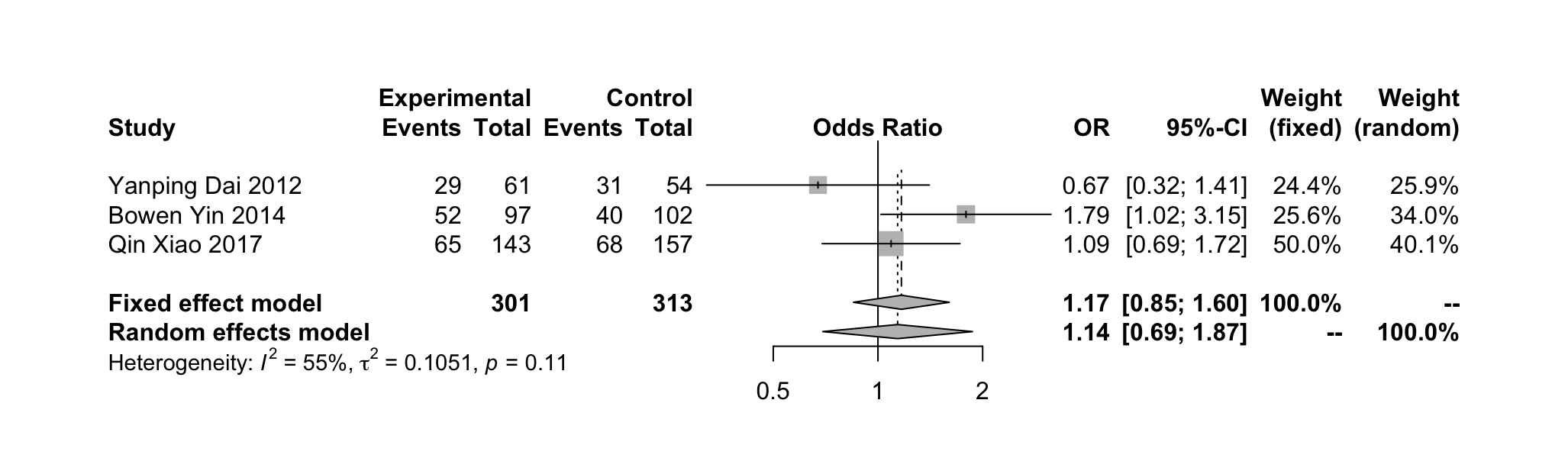


Appendix Figure 3.56 Dominant model of *COMT* rs4633

**Recessive model**: The forest plot and result of recessive model of rs4633 is shown below. Since there was no heterogeneity observed, results of fixed effect model were adopted. After meta-analysis to 301 PD patients and 313 controls, OR was 1.13 (0.60 – 2.13), *p* value: 0.694.


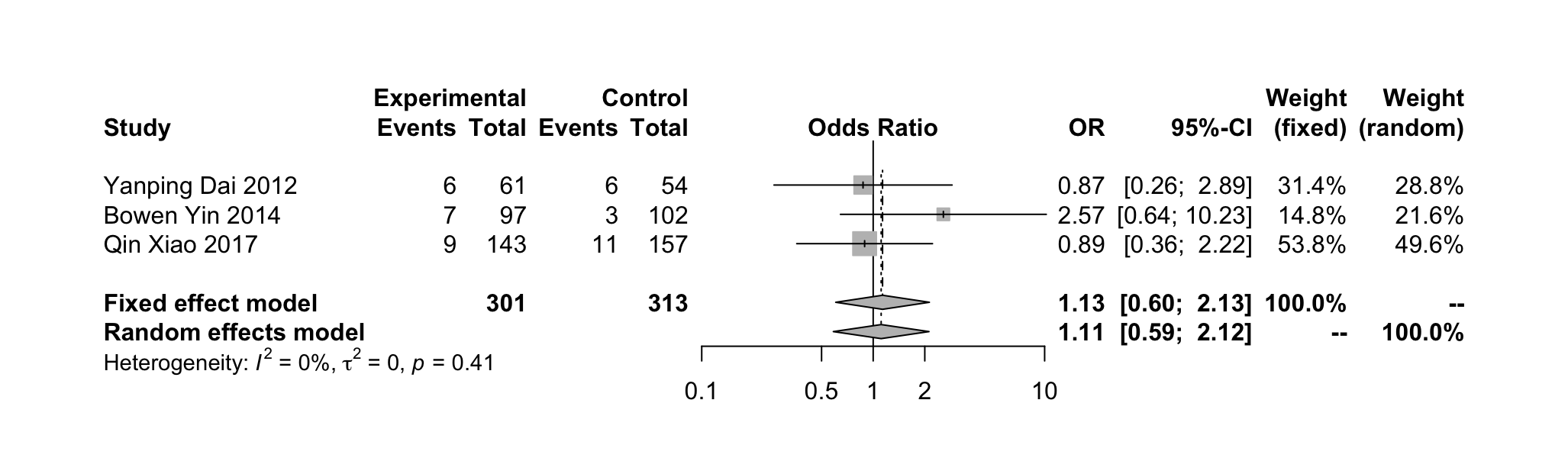


Appendix Figure 3.57 Recessive model of *COMT* rs4633

**Overdominant model**: The forest plot and result of recessive model of rs4633 is shown below. Since there was no heterogeneity observed, results of fixed effect model were adopted. After meta-analysis to 301 PD patients and 313 controls, OR was 0.88 (0.64 – 1.22), *p* value: 0.438.


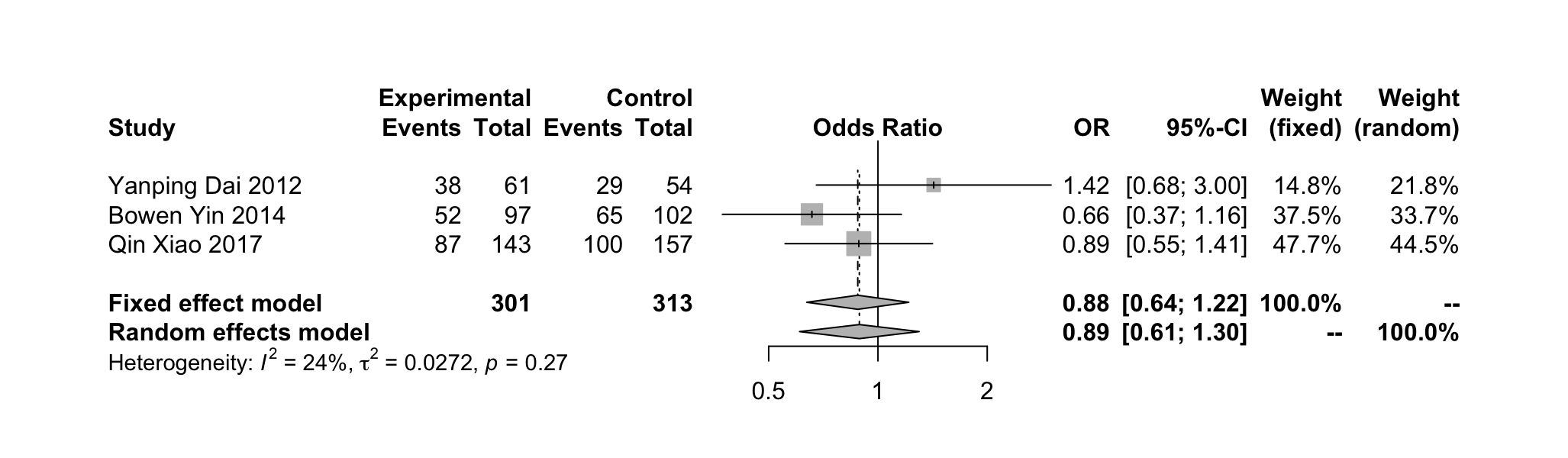


Appendix Figure 3.58 Overdominant model of *COMT* rs4633

1. **rs6267, *COMT*, Ala72Ser, c.214G>T**

| Articles | PD subjects | | | Controls | | | Methods | Location of Population |
| --- | --- | --- | --- | --- | --- | --- | --- | --- |
|  | GG | GT | TT | GG | GT | TT |  |  |
| Yanping Dai et al., 2012^a, 37^ | 45 | 7 | 9 | 39 | 10 | 5 | PCR | Harbin, Heilongjiang Province |
| Bowen Yin et al., 2014^a, 38^ | 74 | 22 | 1 | 84 | 15 | 3 | PCR | Harbin, Heilongjiang Province |
| PCR: Polymerase chain reaction; PD: Parkinson’s disease  a: diagnostic criteria: the United Kingdom brain bank criteria ^3^ | | | | | | | | |

**Allele model**: The forest plot and result of allele model of rs6267 is shown below. We regarded allele T as risk allele. Since there was no heterogeneity observed, results of fixed effect model were adopted. After meta-analysis to 158 PD patients and 156 controls, OR was 1.18 (0.75 – 1.86) compared to allele G. *p* value: 0.464.


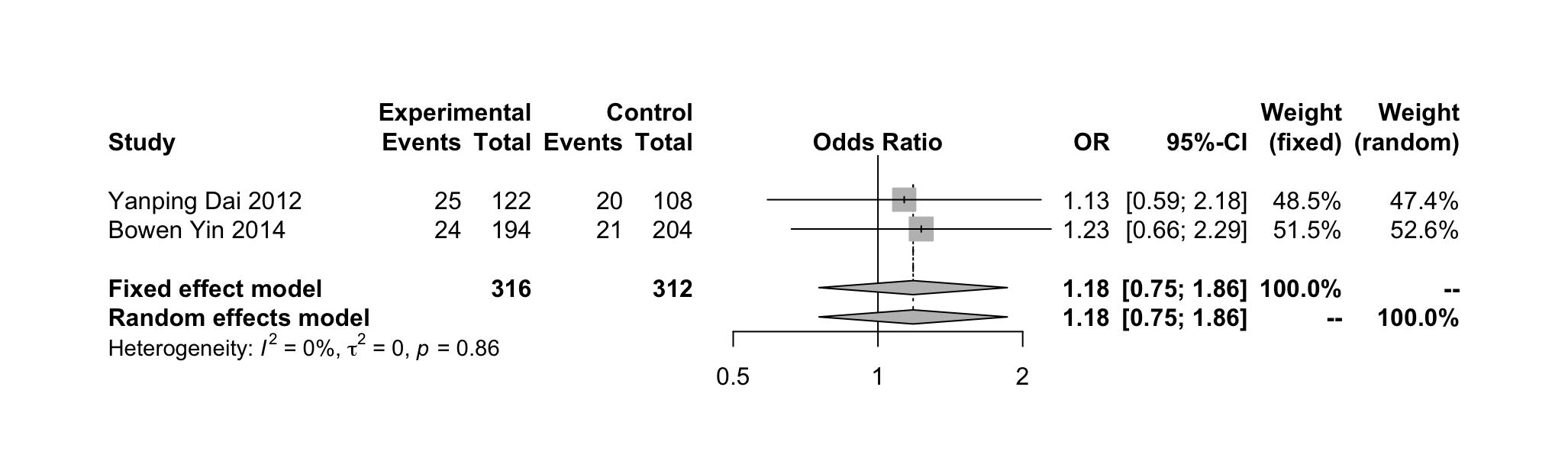


Appendix Figure 3.59 Allele model of *COMT* rs6267

**Dominant model**: The forest plot and result of dominant model of rs6267 is shown below. Since there was no heterogeneity observed, results of fixed effect model were adopted. After meta-analysis to 158 PD patients and 156 controls, OR was 1.20 (0.71 – 2.04), *p* value: 0.490.


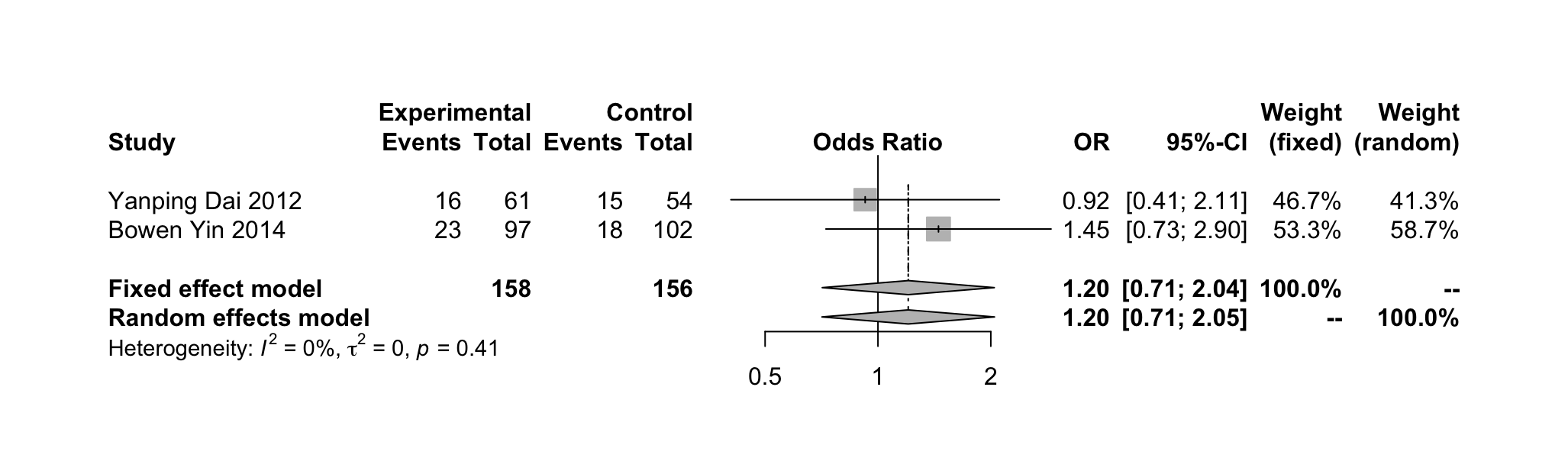


Appendix Figure 3.60 Dominant model of *COMT* rs6267

**Recessive model**: The forest plot and result of recessive model of rs6267 is shown below. Since there was no heterogeneity observed, results of fixed effect model were adopted. After meta-analysis to 158 PD patients and 156 controls, OR was 1.17 (0.44 – 3.11), *p* value: 0.756.


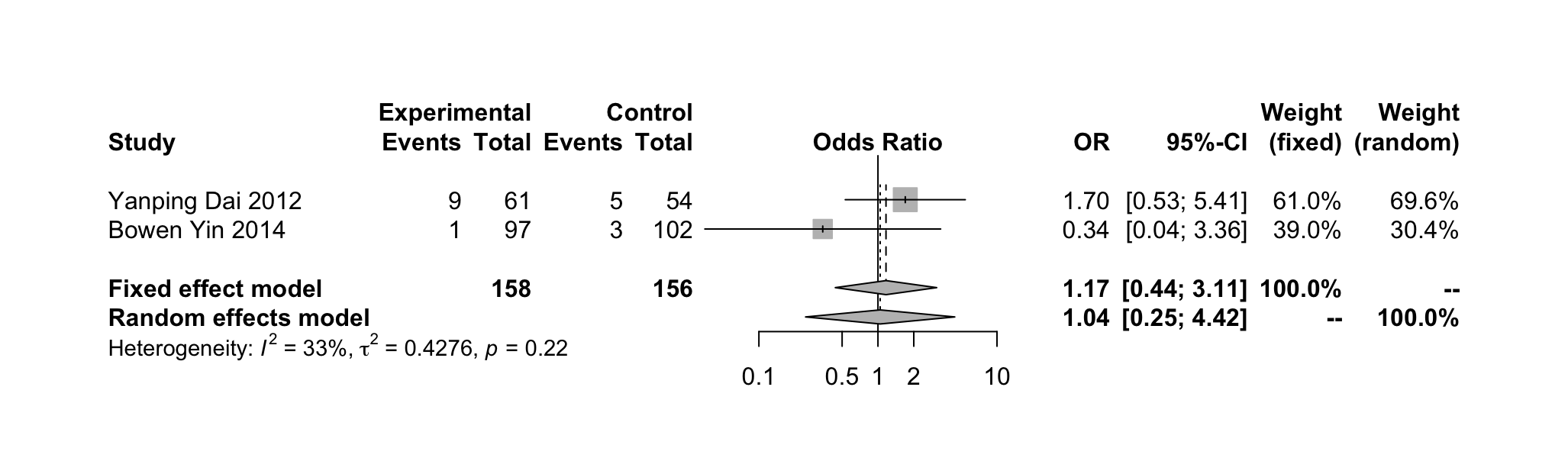


Appendix Figure 3.61 Recessive model of *COMT* rs6267

**Overdominant model**: The forest plot and result of recessive model of rs6267 is shown below. Since there was heterogeneity observed (*I*^2^ 65%), results of random effect model were adopted. After meta-analysis to 158 PD patients and 156 controls, OR was 0.95 (0.33 – 2.75), *p* value: 0.923.


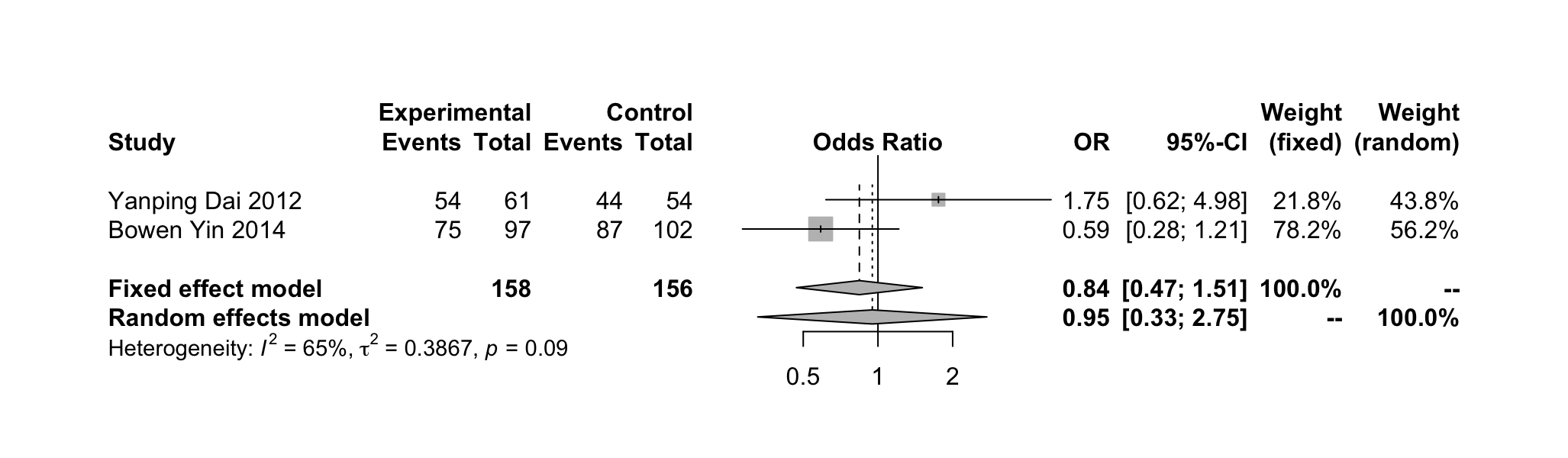


Appendix Figure 3.62 Overdominant model of *COMT* rs6267

1. ***CCDC62/HIP1R*, rs12817488, G>A**

| Articles | PD subjects | | | Controls | | | Methods | Location of Population |
| --- | --- | --- | --- | --- | --- | --- | --- | --- |
|  | AA | AG | GG | AA | AG | GG |  |  |
| Nan-Nan Li et al., 2013^a,39^ | 234 | 366 | 160 | 200 | 330 | 178 | PCR-RFLP | Chengdu, Sichuan Province |
| Rong-Rong Liu et al., 2013^a,40^ | 121 | 154 | 66 | 108 | 216 | 99 | PCR-RFLP | Wenzhou, Zhejiang Province |
| Ri-li Yu et al., 2015^a,41^ | 164 | 249 | 94 | 133 | 247 | 138 | PCR-RFLP | Changsha, Hunan province |
| PCR: Polymerase chain reaction; PD: Parkinson’s disease; RFLP: restricted fragment length polymorphism  a: diagnostic criteria: the United Kingdom brain bank criteria ^3^ | | | | | | | | |

**Allele model**: The forest plot and result of allele model of rs12817488 is shown below. We regarded allele A as risk allele. Since there was no heterogeneity observed, results of fixed effect model were adopted. After meta-analysis to 1608 PD patients and 1649 controls, OR was 1.25 (1.13 – 1.37) compared to allele G. *p* value:<0.0001.


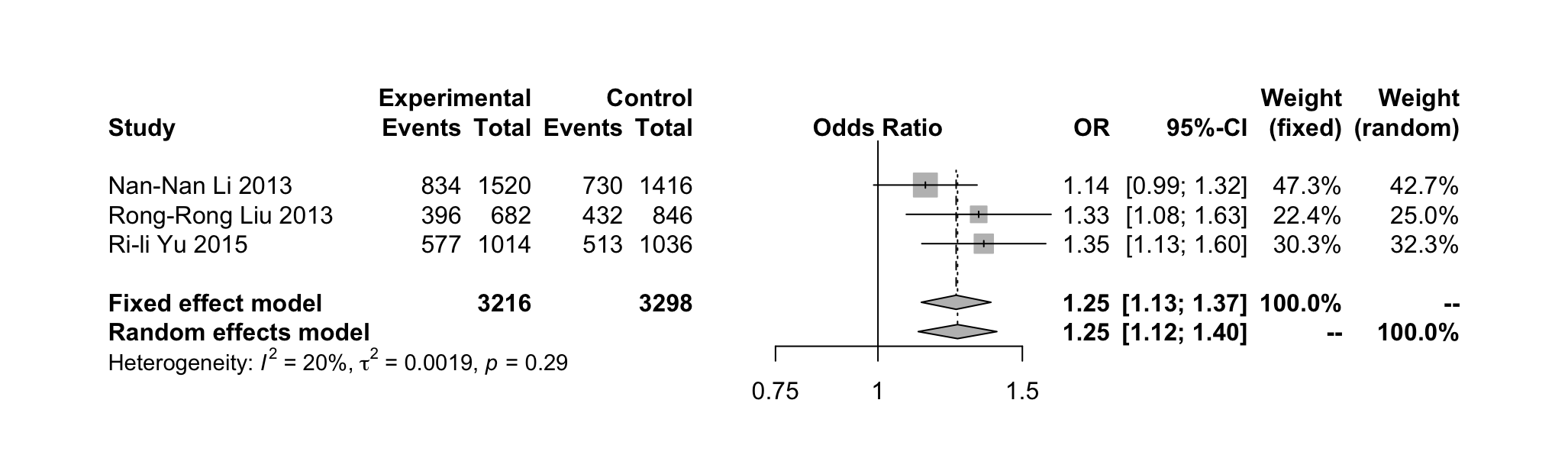


Appendix Figure 3.63 Allele model of *CCDC62/HIP1R* rs12817488

**Dominant model**: The forest plot and result of dominant model of rs12817488 is shown below. Since there was no heterogeneity observed, results of fixed effect model were adopted. After meta-analysis to 1608 PD patients and 1649 controls, OR was 1.36 (1.15 – 1.60), *p* value: 0.0003.


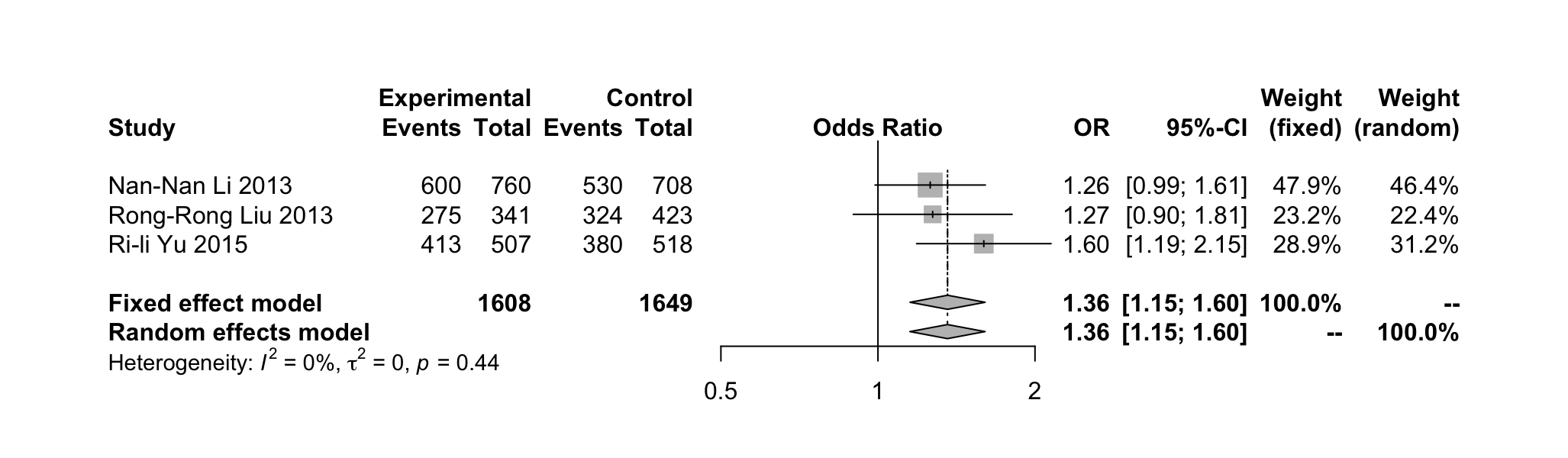


Appendix Figure 3.64 Dominant model of *CCDC62/HIP1R* rs12817488

**Recessive model**: The forest plot and result of recessive model of rs12817488 is shown below. Since there was no heterogeneity observed, results of fixed effect model were adopted. After meta-analysis to 1608 PD patients and 1649 controls, OR was 1.31 (1.12 – 1.52), *p* value: 0.0005.


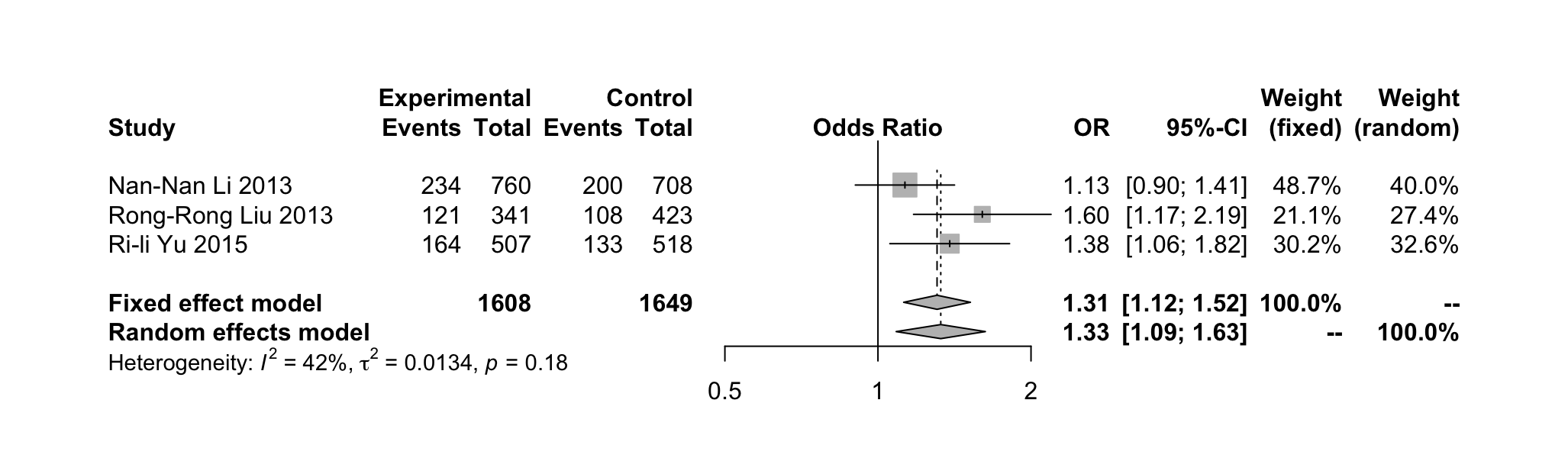


Appendix Figure 3.65 Recessive model of *CCDC62/HIP1R* rs12817488

**Overdominant model**: The forest plot and result of recessive model of rs12817488 is shown below. Since there was no heterogeneity observed, results of fixed effect model were adopted. After meta-analysis to 1608 PD patients and 1649 controls, OR was 1.01 (0.88 – 1.16), *p* value: 0.899.


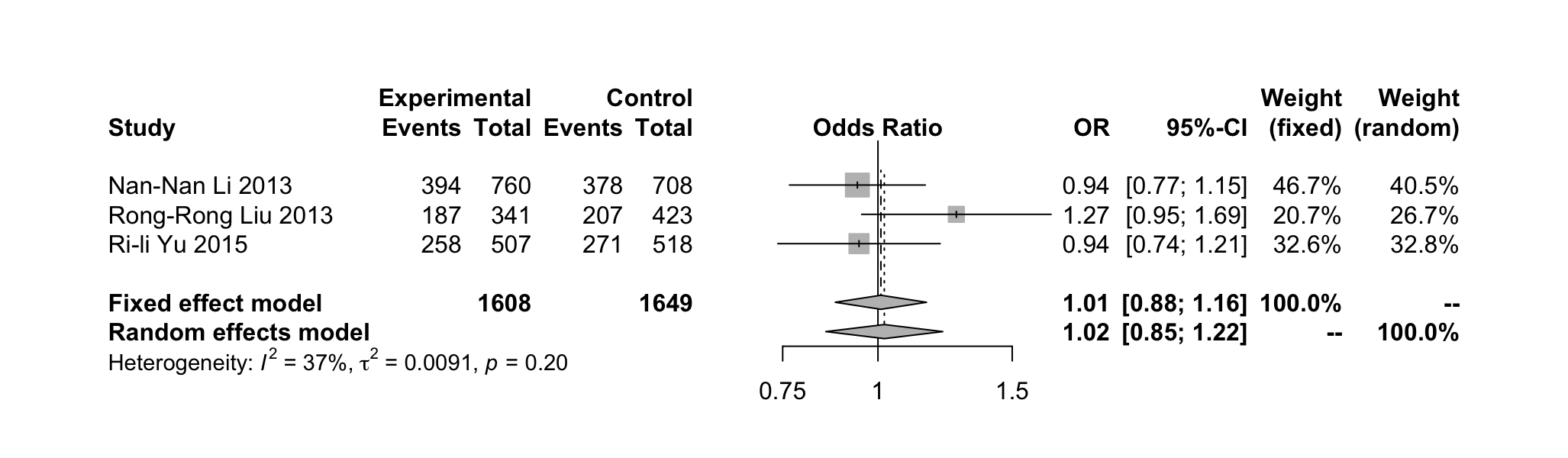


Appendix Figure 3.66 Overdominant model of *CCDC62/HIP1R* rs12817488

1. ***CTNNA3*, rs7903491, A>G**

| Articles | PD subjects | | | Controls | | | Methods | Location of Population |
| --- | --- | --- | --- | --- | --- | --- | --- | --- |
|  | AA | AG | GG | AA | AG | GG |  |  |
| Yuan Zhang et al., 2017^a,42^ | 174 | 246 | 90 | 182 | 265 | 89 | PCR | Changsha, Hunan Province |
| Chang-he Shi et al., 2018^a,43^ | 178 | 260 | 108 | 178 | 276 | 96 | PCR | Chengdu, Sichuan Province |
| PCR: Polymerase chain reaction; PD: Parkinson’s disease  a: diagnostic criteria: the United Kingdom brain bank criteria ^3^ | | | | | | | | |

**Allele model**: The forest plot and result of allele model of rs7903491 is shown below. We regarded allele G as risk allele. Since there was no heterogeneity observed, results of fixed effect model were adopted. After meta-analysis to 1056 PD patients and 1086 controls, OR was 1.03 (0.91 – 1.16) compared to allele A. *p* value: 0.620.


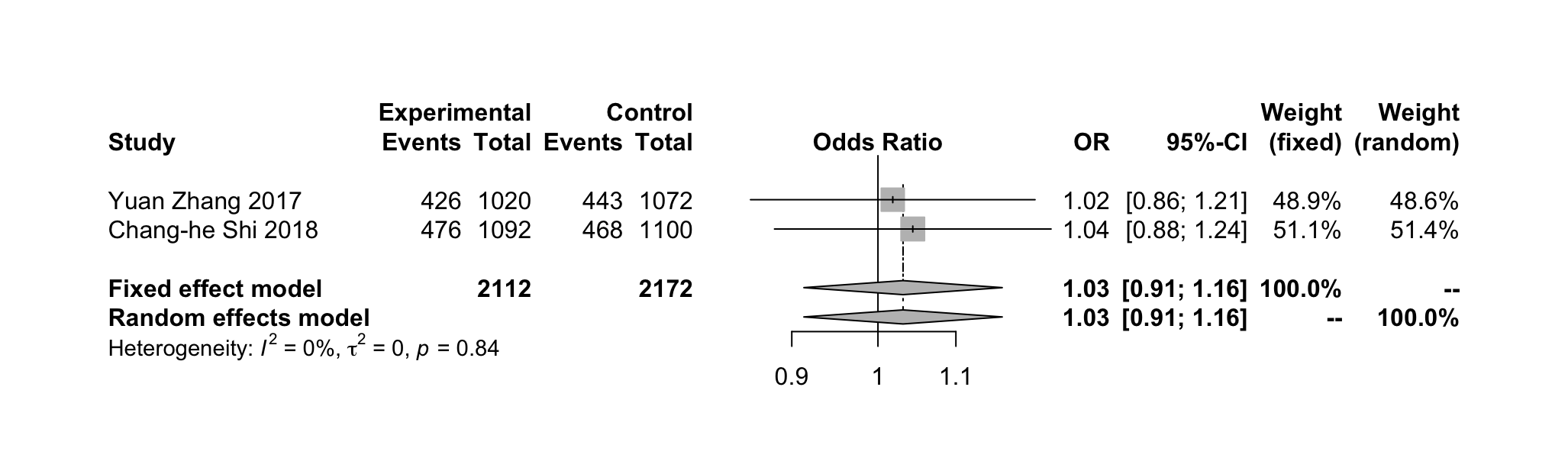


Appendix Figure 3.67 Allele model of *CTNNA3* rs7903491

**Dominant model**: The forest plot and result of dominant model of rs7903491 is shown below. Since there was no heterogeneity observed, results of fixed effect model were adopted. After meta-analysis to 1056 PD patients and 1086 controls, OR was 0.99 (0.83 – 1.19), *p* value: 0.922.


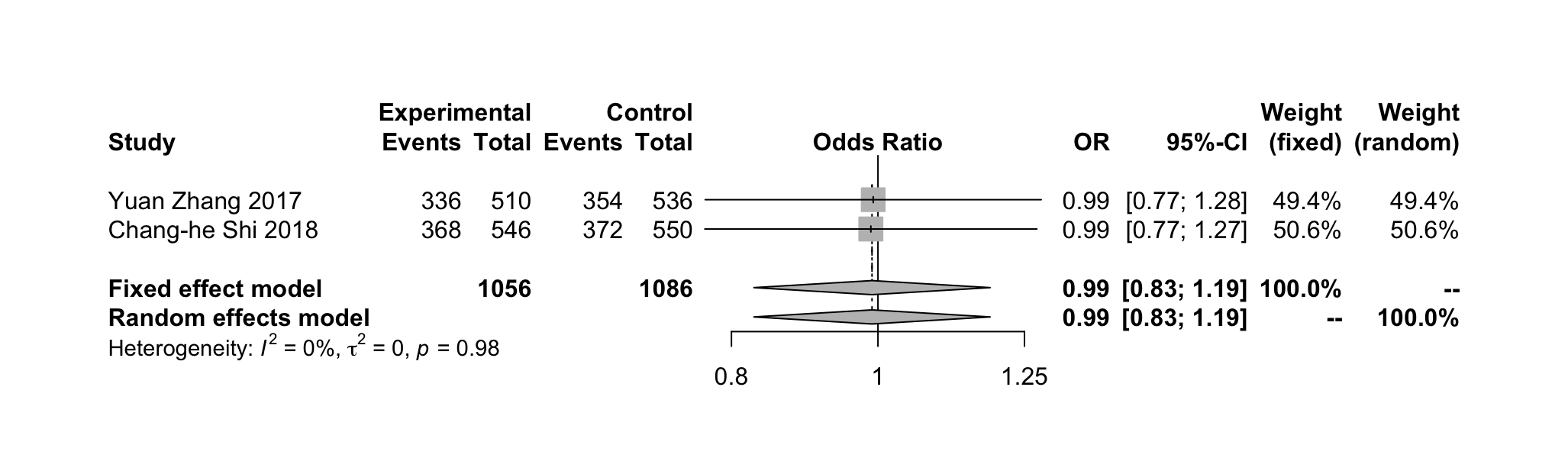


Appendix Figure 3.68 Dominant model of *CTNNA3* rs7903491

**Recessive model**: The forest plot and result of recessive model of rs7903491 is shown below. Since there was no heterogeneity observed, results of fixed effect model were adopted. After meta-analysis to 1056 PD patients and 1086 controls, OR was 1.12 (0.90 – 1.40), *p* value: 0.305.


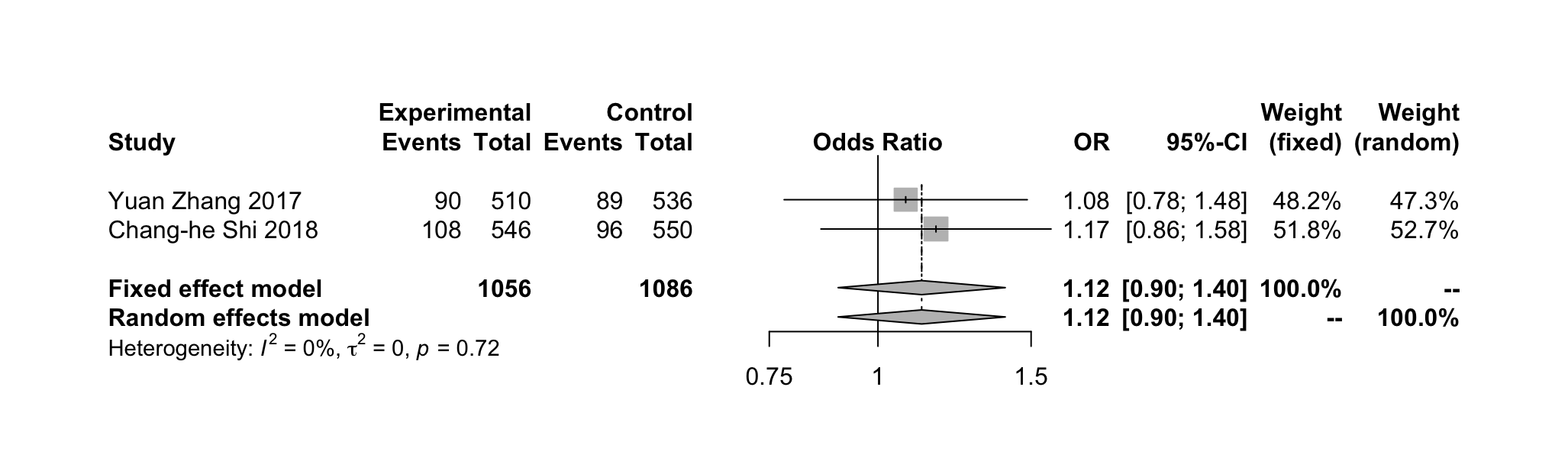


Appendix Figure 3.69 Recessive model of *CTNNA3* rs7903491

**Overdominant model**: The forest plot and result of recessive model of rs7903491 is shown below. Since there was no heterogeneity observed, results of fixed effect model were adopted. After meta-analysis to 1056 PD patients and 1086 controls, OR was 1.08 (0.91 – 1.28), *p* value: 0.379.


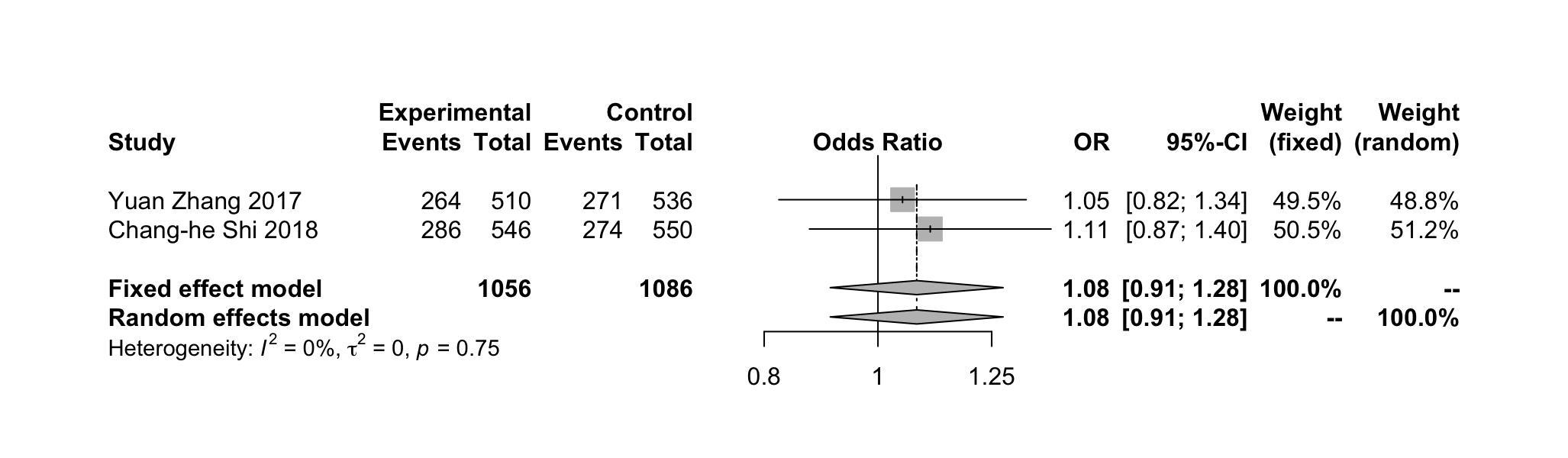


Appendix Figure 3.70 Overdominant model of *CTNNA3* rs7903491

1. ***CTNNA3*, rs10822974, A>G / A>T**

| Articles | PD subjects | | | Controls | | | Methods | Location of Population |
| --- | --- | --- | --- | --- | --- | --- | --- | --- |
|  | AA | AG | GG | AA | AG | GG |  |  |
| Yuan Zhang et al., 2017^a,42^ | 108 | 256 | 146 | 124 | 266 | 146 | PCR | Changsha, Hunan Province |
| Chang-he Shi et al., 2018^a,43^ | 92 | 282 | 172 | 92 | 278 | 180 | PCR | Chengdu, Sichuan Province |
| PCR: Polymerase chain reaction; PD: Parkinson’s disease  a: diagnostic criteria: the United Kingdom brain bank criteria ^3^ | | | | | | | | |

**Allele model**: The forest plot and result of allele model of rs10822974 is shown below. We regarded allele G as risk allele. Since there was no heterogeneity observed, results of fixed effect model were adopted. After meta-analysis to 1056 PD patients and 1086 controls, OR was 1.02 (0.90 – 1.15) compared to allele A. *p* value: 0.756.


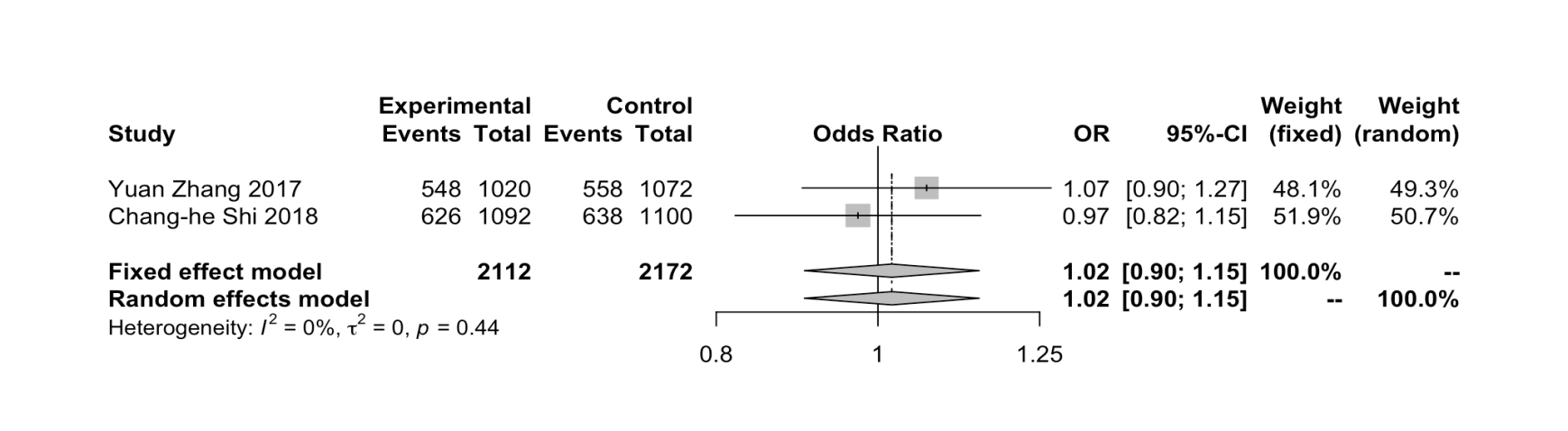


Appendix Figure 3.71 Allele model of *CTNNA3* rs10822974

**Dominant model**: The forest plot and result of dominant model of rs10822974 is shown below. Since there was no heterogeneity observed, results of fixed effect model were adopted. After meta-analysis to 1056 PD patients and 1086 controls, OR was 1.06 (0.85 – 1.31), *p* value: 0.601.


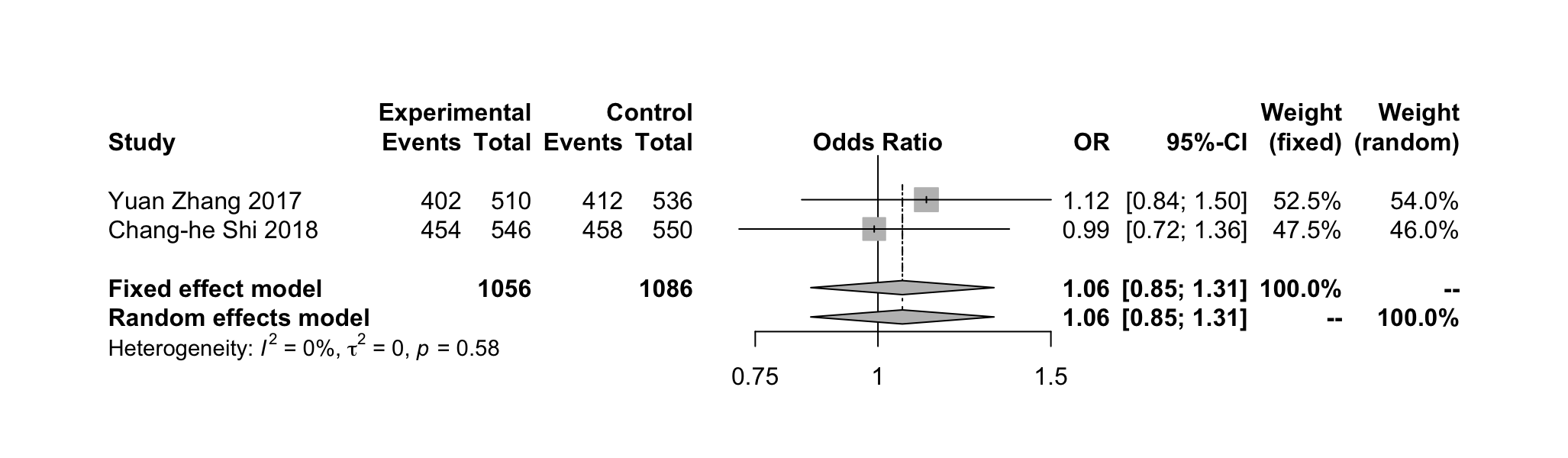


Appendix Figure 3.72 Dominant model of *CTNNA3* rs10822974

**Recessive model**: The forest plot and result of recessive model of rs10822974 is shown below. Since there was no heterogeneity observed, results of fixed effect model were adopted. After meta-analysis to 1056 PD patients and 1086 controls, OR was 1.00 (0.83 – 1.21), *p* value: 0.980.


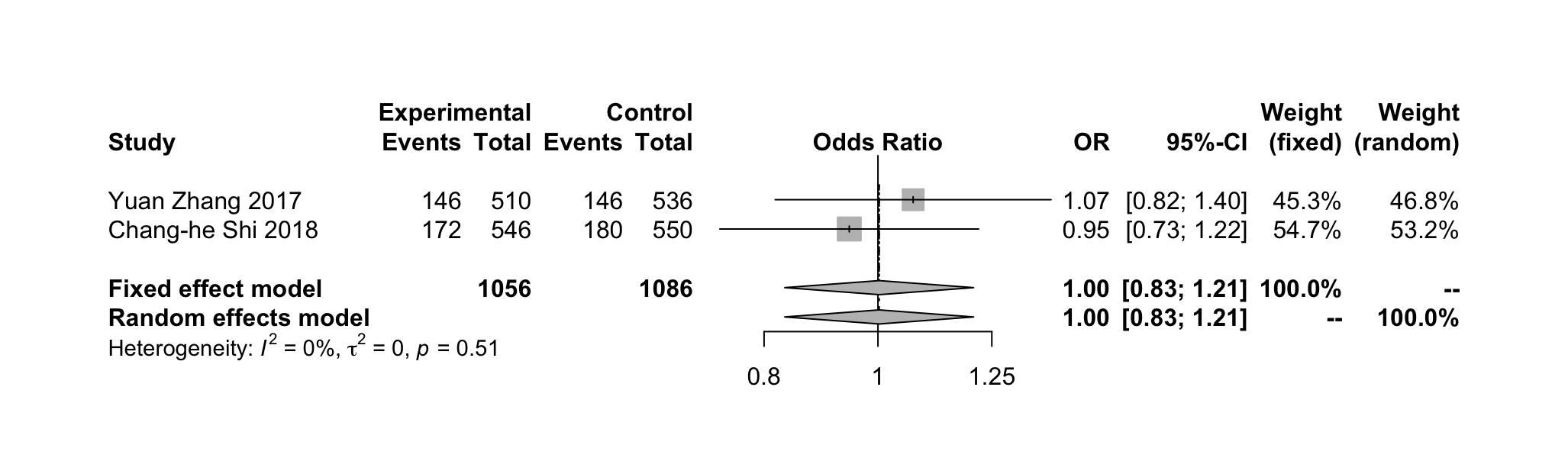


Appendix Figure 3.73 Recessive model of *CTNNA3* rs10822974

**Overdominant model**: The forest plot and result of recessive model of rs10822974 is shown below. Since there was no heterogeneity observed, results of fixed effect model were adopted. After meta-analysis to 1056 PD patients and 1086 controls, OR was 0.97 (0.82 – 1.15), *p* value: 0.697.


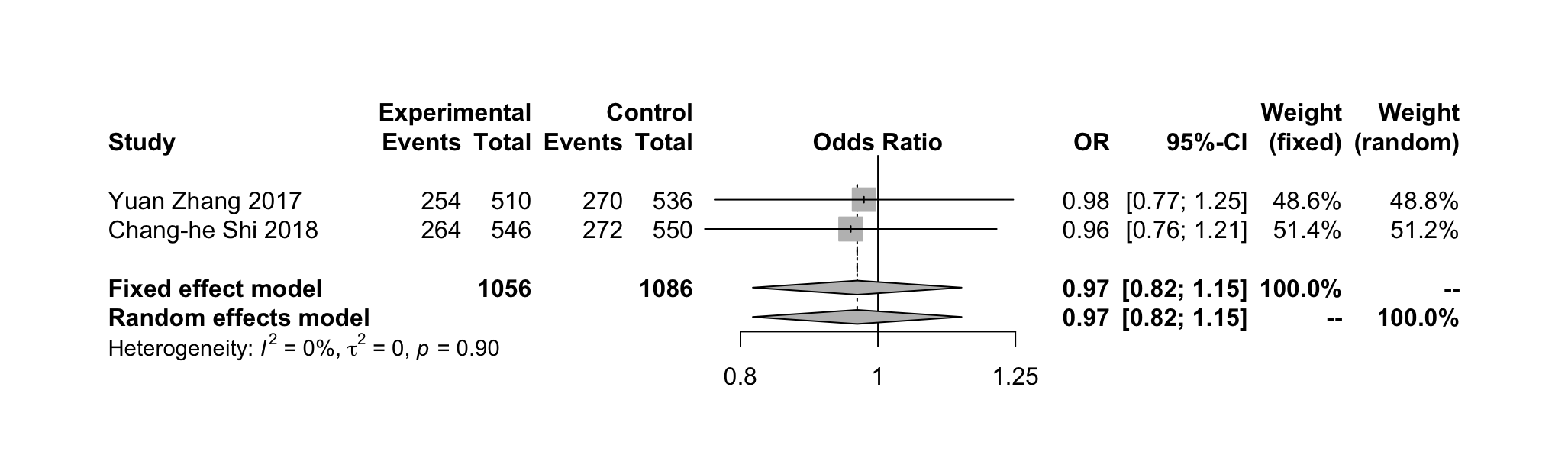


Appendix Figure 3.74 Overdominant model of *CTNNA3* rs10822974

1. ***CTNNA3*, rs12764057, T>G**

| Articles | PD subjects | | | Controls | | | Methods | Location of Population |
| --- | --- | --- | --- | --- | --- | --- | --- | --- |
|  | GG | GT | TT | GG | GT | TT |  |  |
| Yuan Zhang et al., 2017^a,42^ | 34 | 211 | 265 | 36 | 210 | 290 | PCR | Changsha, Hunan Province |
| Chang-he Shi et al., 2018^a,43^ | 50 | 246 | 250 | 50 | 263 | 237 | PCR | Chengdu, Sichuan Province |
| PCR: Polymerase chain reaction; PD: Parkinson’s disease  a: diagnostic criteria: the United Kingdom brain bank criteria ^3^ | | | | | | | | |

**Allele model**: The forest plot and result of allele model of rs12764057 is shown below. We regarded allele G as risk allele. Since there was no heterogeneity observed, results of fixed effect model were adopted. After meta-analysis to 1056 PD patients and 1086 controls, OR was 0.99 (0.83 – 1.13) compared to allele T. *p* value: 0.908.


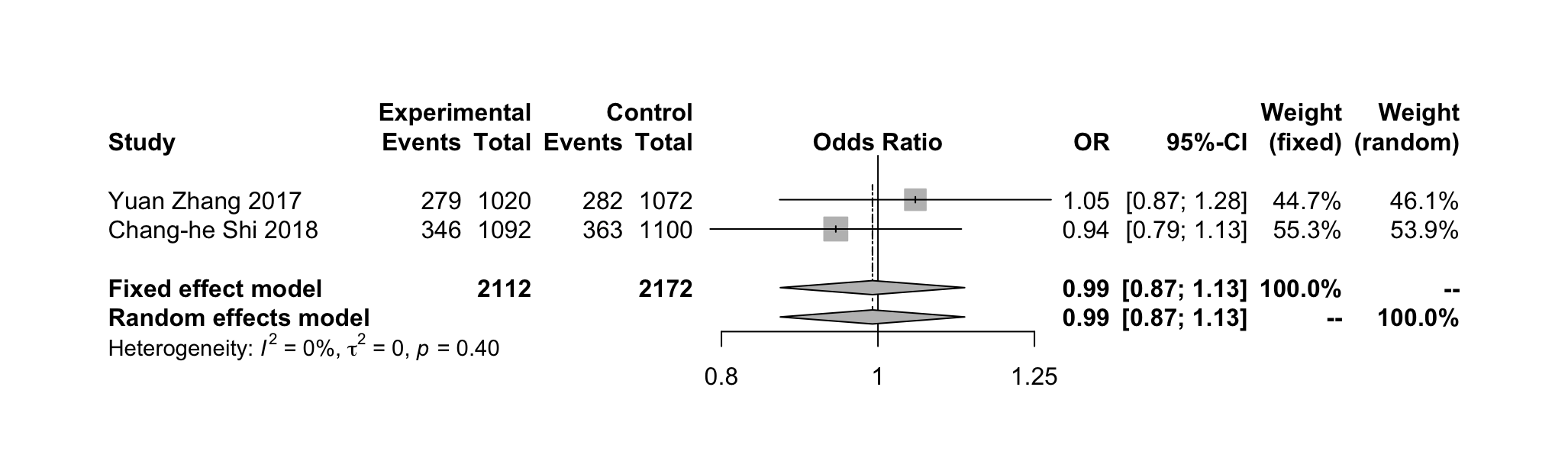


Appendix Figure 3.75 Allele model of *CTNNA3* rs12764057

**Dominant model**: The forest plot and result of dominant model of rs12764057 is shown below. Since there was no heterogeneity observed, results of fixed effect model were adopted. After meta-analysis to 1056 PD patients and 1086 controls, OR was 0.99 (0.83 – 1.17), *p* value: 0.877.


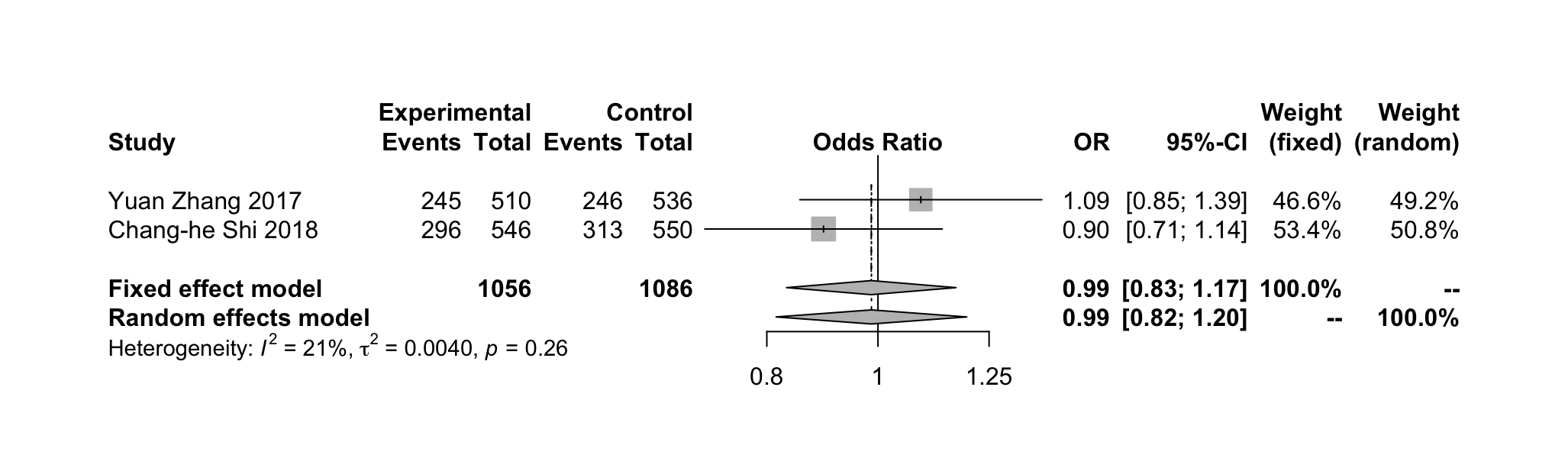


Appendix Figure 3.76 Dominant model of *CTNNA3* rs12764057

**Recessive model**: The forest plot and result of recessive model of rs12764057 is shown below. Since there was no heterogeneity observed, results of fixed effect model were adopted. After meta-analysis to 1056 PD patients and 1086 controls, OR was 1.00 (0.73 – 1.37), *p* value: 0.993.


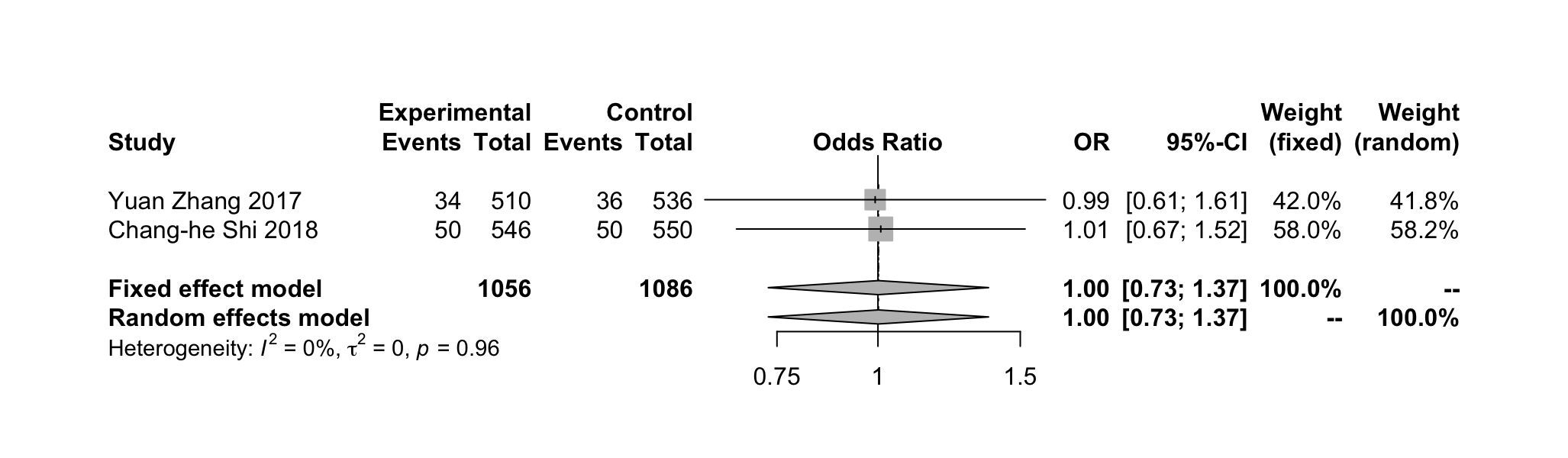


Appendix Figure 3.77 Recessive model of *CTNNA3* rs12764057

**Overdominant model**: The forest plot and result of recessive model of rs12764057 is shown below. Since there was no heterogeneity observed, results of fixed effect model were adopted. After meta-analysis to 1056 PD patients and 1086 controls, OR was 1.01 (0.85 – 1.20), *p* value: 0.872.


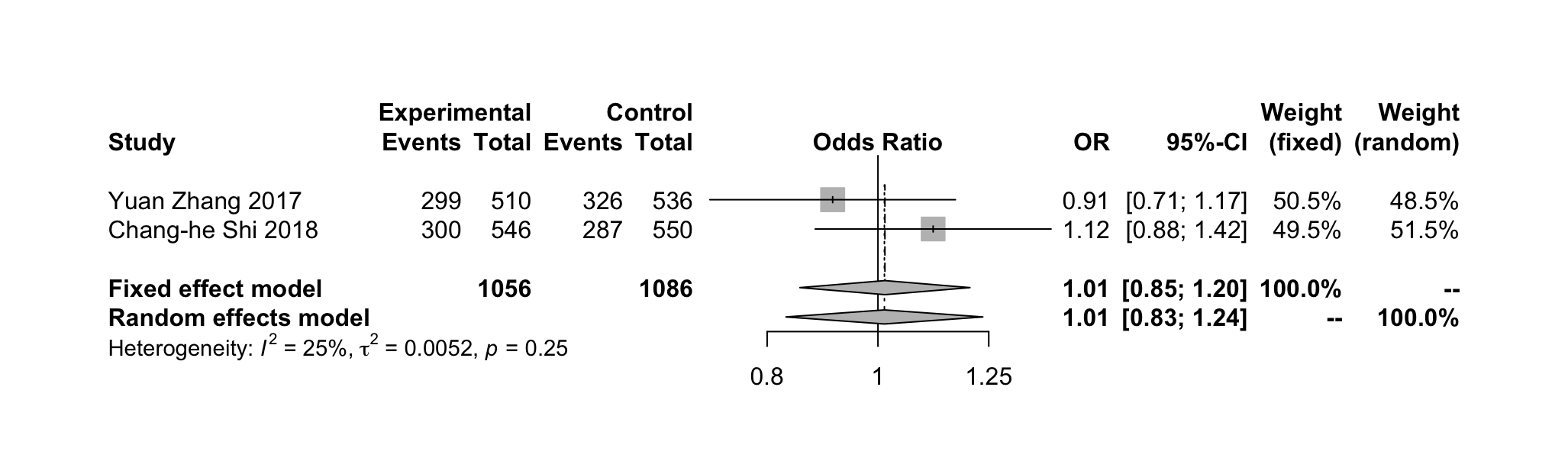


Appendix Figure 3.78 Overdominant model of *CTNNA3* rs12764057

1. ***DJ-1/PARK7*, g.168_185del**

| Articles | PD subjects | | | Controls | | | Methods | Location of Population |
| --- | --- | --- | --- | --- | --- | --- | --- | --- |
|  | D/D | I/D | I/I | D/D | I/D | I/I |  |  |
| Sixin Liu et al., 2008^a,44^ | 0 | 25 | 188 | 1 | 28 | 166 | PCR | Changsha, Hunan Province |
| Wenjun Chen et al., 2008^a, 45^ | 0 | 2 | 190 | 0 | 1 | 196 | PCR | Chengdu, Sichuan Province |
| Hongjuan Li et al., 2012^a, 46^ | 0 | 21 | 168 | 0 | 18 | 165 | PCR | Hetian District & Urumqi, Xinjiang |
| Hongjuan Li et al., 2012^a, *,46^ | 1 | 24 | 150 | 0 | 20 | 143 | PCR | Hetian District & Urumqi, Xinjiang |
| PCR: Polymerase chain reaction; PD: Parkinson’s disease  a: diagnostic criteria: the United Kingdom brain bank criteria ^3^  *: population were sourced from Uygur ethnicity. We did not include this study into meta-analysis due to different ethnicity base. | | | | | | | | |

**Allele model**: The forest plot and result of allele model of g.168_185del is shown below. We regarded del as risk allele. Since there was no heterogeneity observed, results of fixed effect model were adopted. After meta-analysis to 594 PD patients and 575 controls, OR was 0.92 (0.61 – 1.38). *p* value: 0.674.


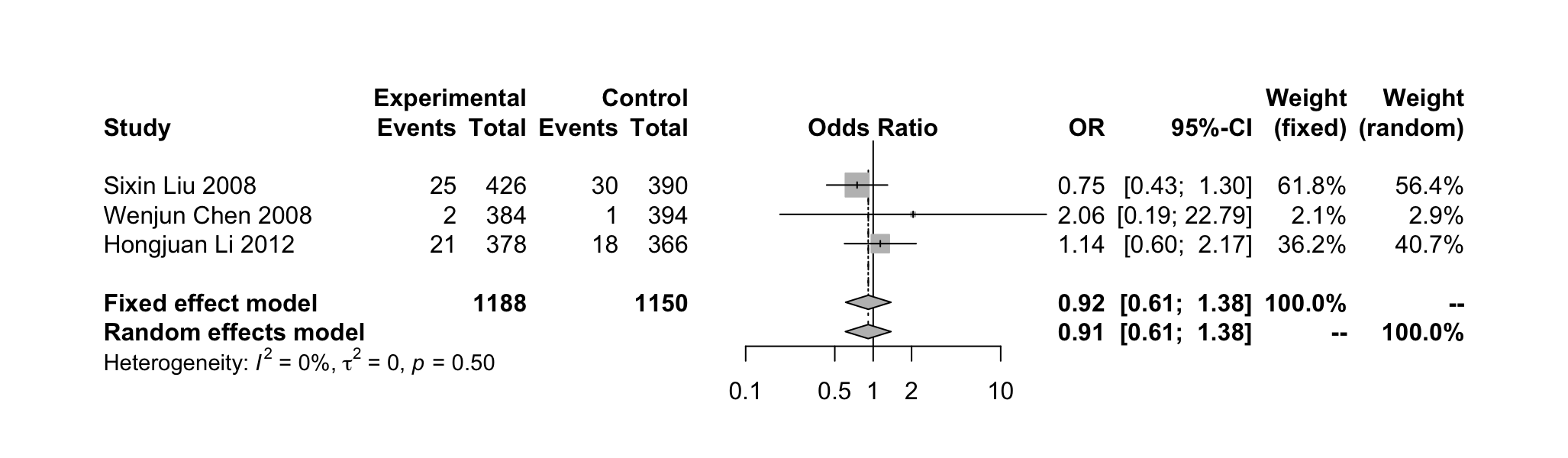


Appendix Figure 3.79 Allele model of *DJ-1/PARK7* g.168_185del

**Dominant model**: The forest plot and result of dominant model of g.168_185del is shown below. Since there was no heterogeneity observed, results of fixed effect model were adopted. After meta-analysis to 594 PD patients and 575 controls, OR was 0.93 (0.61 – 1.43), *p* value: 0.747.


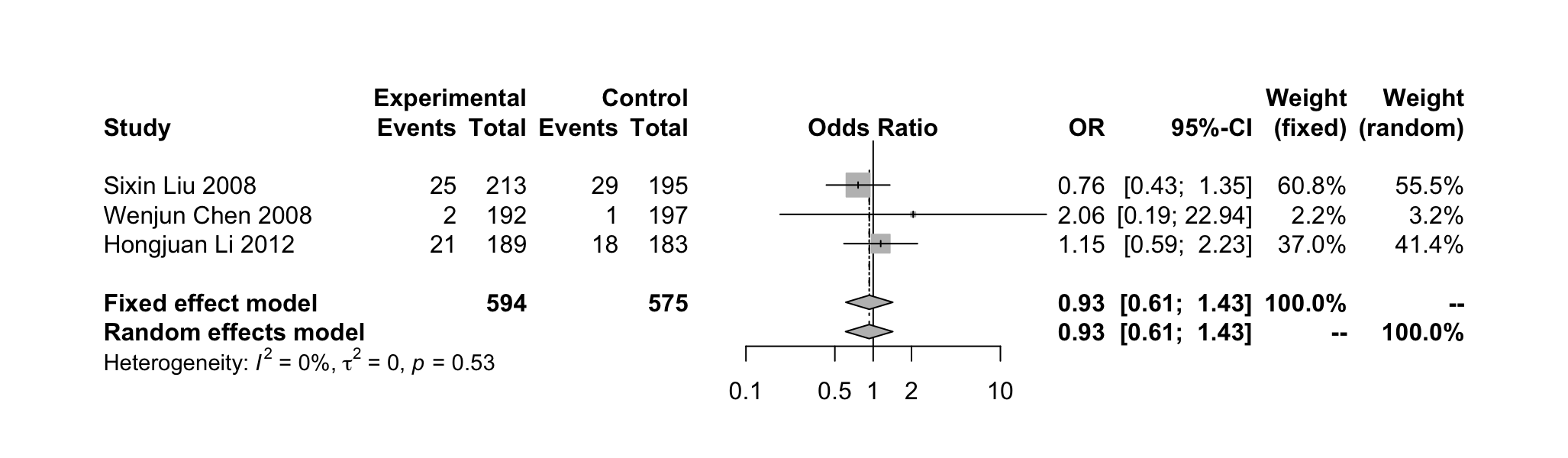


Appendix Figure 3.80 Dominant model of *DJ-1/PARK7* g.168_185del

**Recessive model**: It was not applied due to the absence of D/D in two studies.

**Overdominant model**: The forest plot and result of recessive model of g.168_185del is shown below. Since there was no heterogeneity observed, results of fixed effect model were adopted. After meta-analysis to 594 PD patients and 575 controls, OR was 1.05 (0.68 – 1.61), *p* value: 0.833.


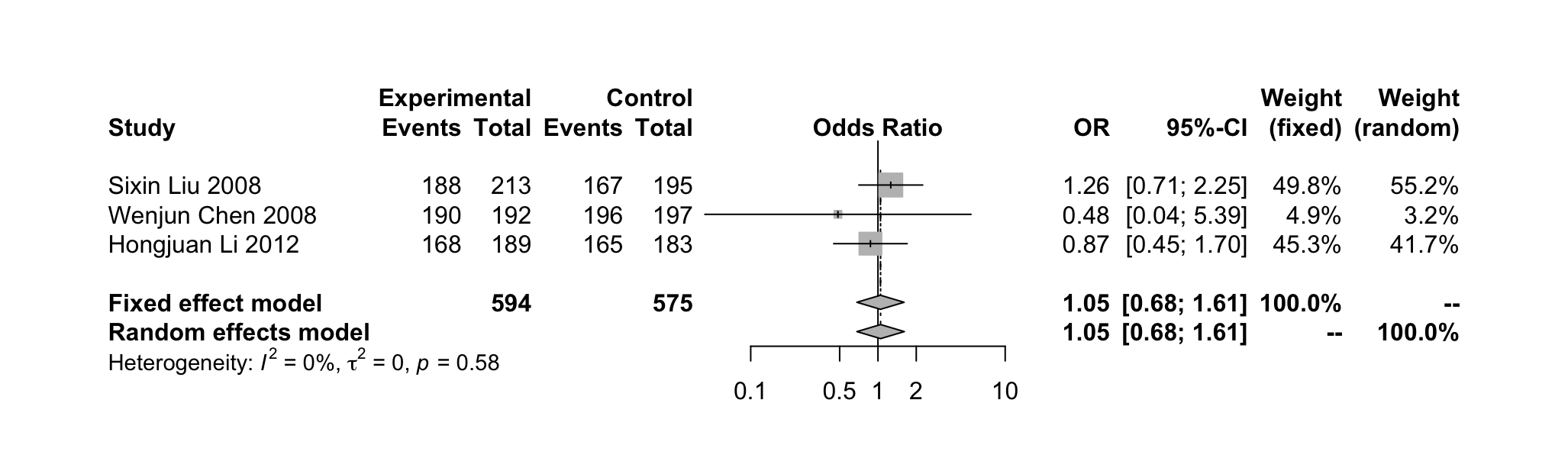


Appendix Figure 3.81 Overdominant model of *DJ-1/PARK7* g.168_185del

1. **rs11107, *PARK15/FBXO7*, M115I, c.345G>A**

| Articles | PD subjects | | | Controls | | | Methods | Location of Population |
| --- | --- | --- | --- | --- | --- | --- | --- | --- |
|  | AA | AG | GG | AA | AG | GG |  |  |
| Zhu Lanhui et al., 2012^a, 47^ | 20 | 95 | 100 | 20 | 87 | 105 | PCR-RFLP | Shenyang, Liaoning Province |
| Chiung-Mei Chen et al., 2014^b, 48^ | 258 | 220 | 38 | 258 | 221 | 37 | PCR-RFLP | Taipei, Taiwan |
| PCR: Polymerase chain reaction; PD: Parkinson’s disease; RFLP: restricted fragment length polymorphism  a: All patients fulfilled the criteria for clinical diagnosis of PD with at least 2 of 3 cardinal signs (bradykinesia, tremor, rigidity) and a positive response to levodopa therapy  b: diagnostic criteria brought up by Gelb et al. ^15^ | | | | | | | | |

**Allele model**: The forest plot and result of allele model of rs11107 is shown below. We regarded allele A as risk allele. Since there was no heterogeneity observed, results of fixed effect model were adopted. After meta-analysis to 731 PD patients and 728 controls, OR was 1.02 (0.87 – 1.19) compared to allele G. *p* value: 0.834.


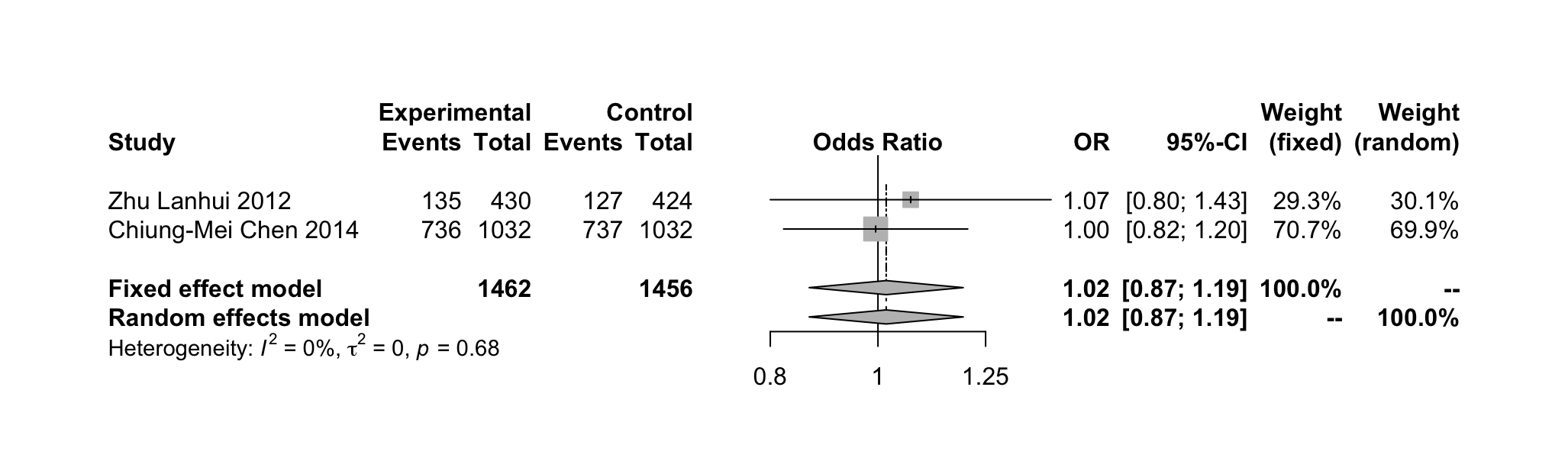


Appendix Figure 3.82 Allele model of *PARK15/FBXO7* rs11107

**Dominant model**: The forest plot and result of dominant model of rs11107 is shown below. Since there was no heterogeneity observed, results of fixed effect model were adopted. After meta-analysis to 731 PD patients and 728 controls, OR was 1.06 (0.79 – 1.43), *p* value: 0.682.


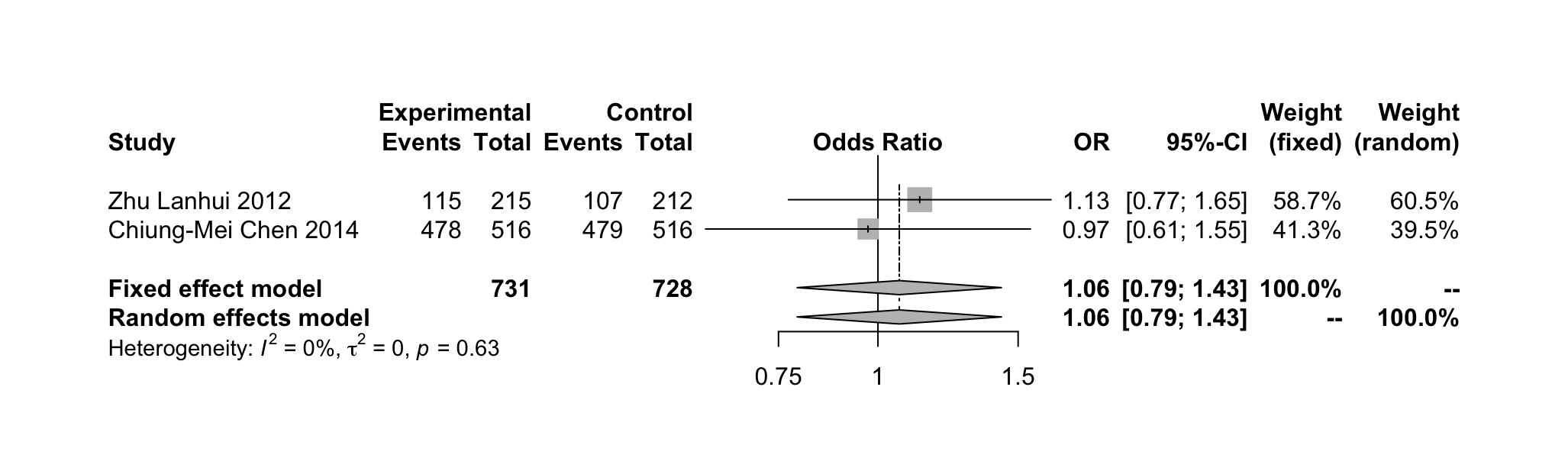


Appendix Figure 3.83 Dominant model of *PARK15/FBXO7* rs11107

**Recessive model**: The forest plot and result of recessive model of rs11107 is shown below. Since there was no heterogeneity observed, results of fixed effect model were adopted. After meta-analysis to 731 PD patients and 728 controls, OR was 1.00 (0.79 – 1.25), *p* value: 0.987.


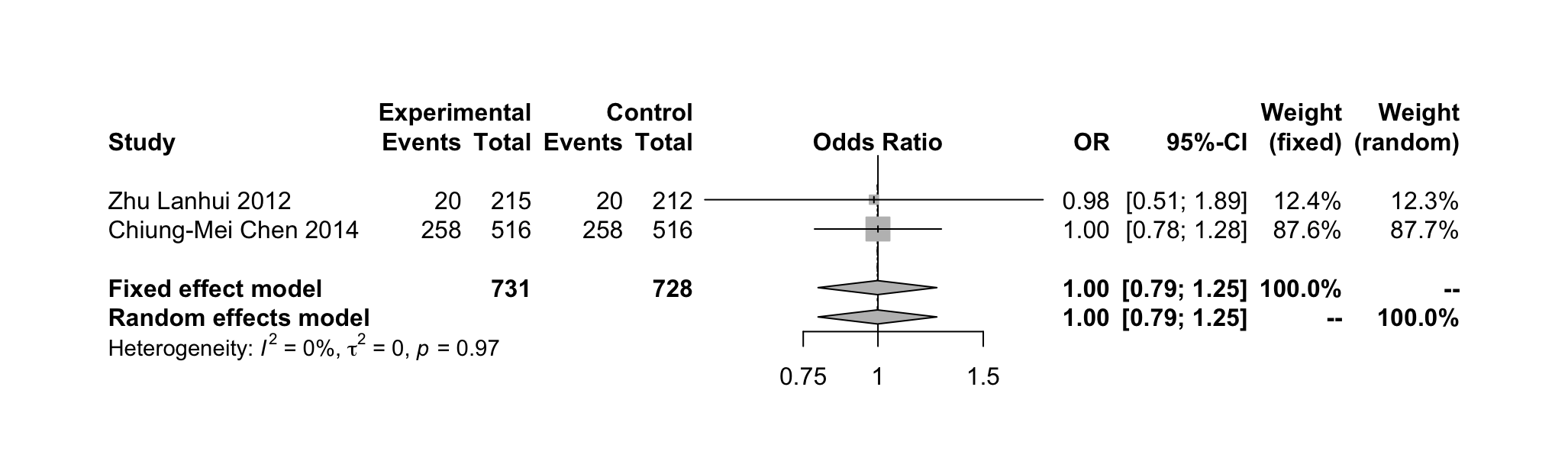


Appendix Figure 3.84 Recessive model of *PARK15/FBXO7* rs11107

**Overdominant model**: The forest plot and result of recessive model of rs11107 is shown below. Since there was no heterogeneity observed, results of fixed effect model were adopted. After meta-analysis to 731 PD patients and 728 controls, OR was 0.97 (0.79 – 1.19), *p* value: 0.762.


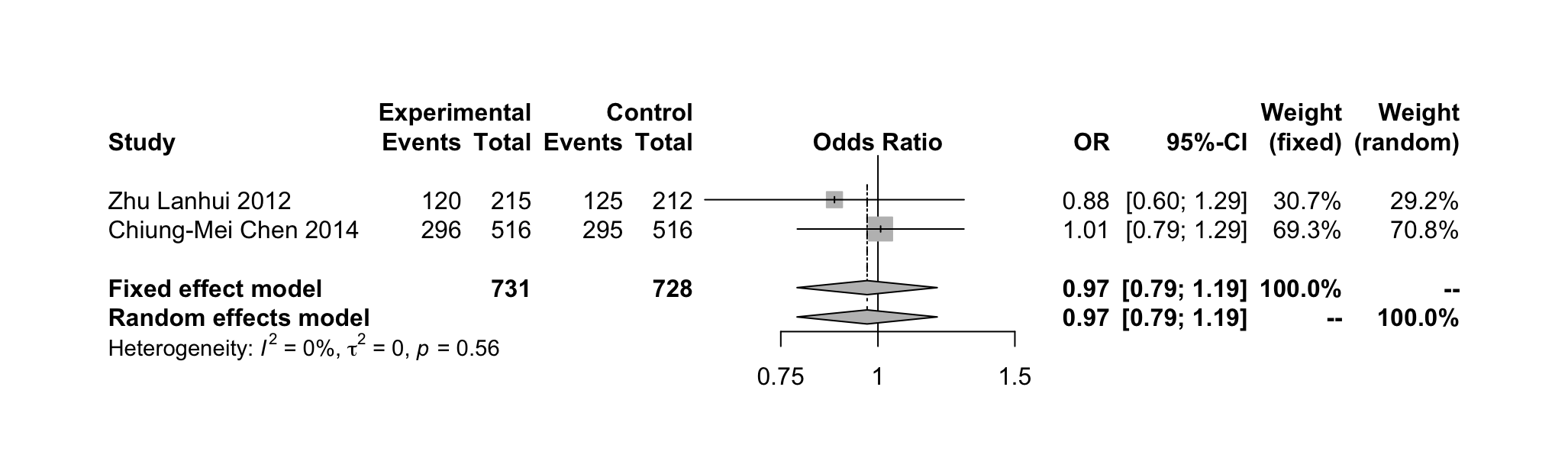


Appendix Figure 3.85 Overdominant model of *PARK15/FBXO7* rs11107

1. ***FGF20*, rs591323, G>A / G>C**

| Articles | PD subjects | | | Controls | | | Methods | Location of Population |
| --- | --- | --- | --- | --- | --- | --- | --- | --- |
|  | AA | AG | GG | AA | AG | GG |  |  |
| Xiao-Yi Sun et al., 2017^a,49^ | 356 | 517 | 178 | 344 | 592 | 233 | MassARRAY | Chengdu, Sichuan Province |
| C.C. Jing et al., 2015^b,50^ | 57 | 155 | 101 | 78 | 159 | 81 | PCR-RFLP | Shenyang, Liaoning Province |
| PCR: Polymerase chain reaction; PD: Parkinson’s disease; RFLP: restricted fragment length polymorphism  a: diagnostic criteria: the United Kingdom brain bank criteria ^3^  b: diagnostic criteria: the clinical diagnosis of PD, presenting at least 2 of the 3 cardinal signs for PD (tremor, rigidity, and bradykinesia) and had a positive response to levodopa therapy | | | | | | | | |

**Allele model**: The forest plot and result of allele model of rs591323 is shown below. We regarded allele A as risk allele. Since there was heterogeneity observed (*I*^2^ 90%), results of random effect model were adopted. After meta-analysis to 1364 PD patients and 1487 controls, OR was 0.96 (0.64 – 1.44) compared to allele G. *p* value: 0.827.


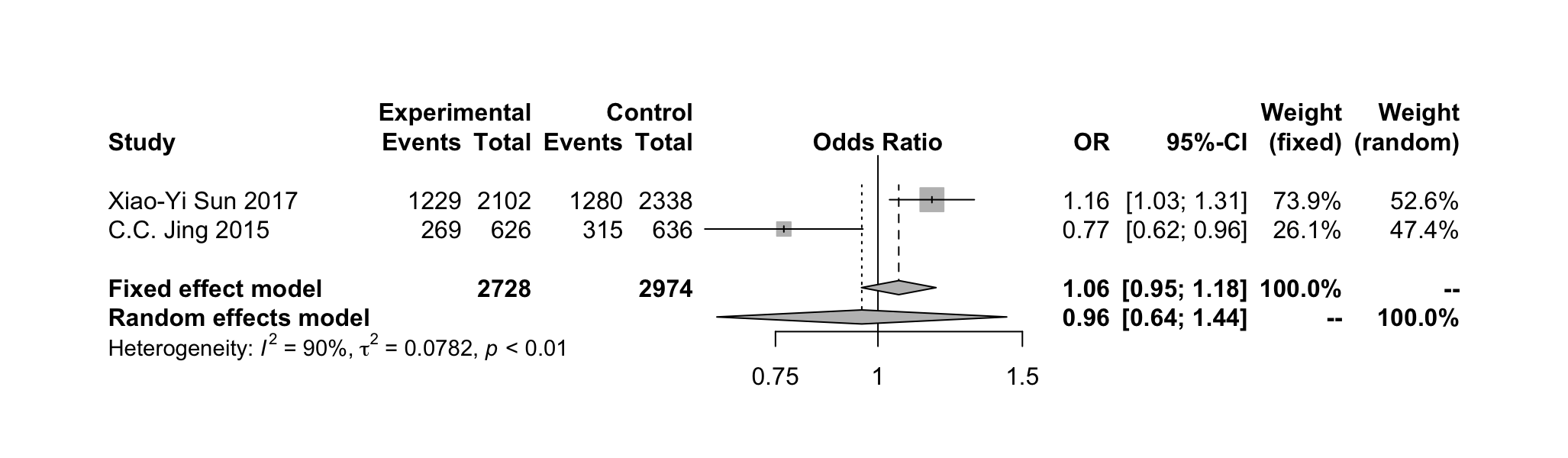


Appendix Figure 3.86 Allele model of *FGF20* rs591323

**Dominant model**: The forest plot and result of dominant model of rs591323 is shown below. Since there was heterogeneity observed (*I*^2^ 85%), results of random effect model were adopted. After meta-analysis to 1364 PD patients and 1487 controls, OR was 0.95 (0.57 – 1.60), *p* value: 0.587.


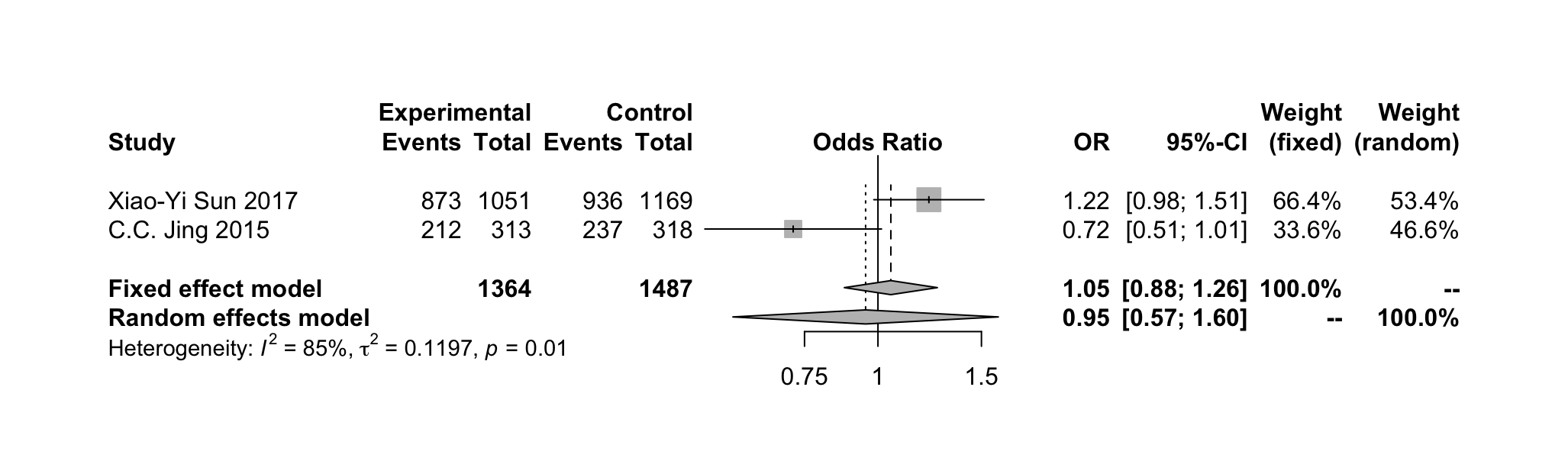


Appendix Figure 3.87 Dominant model of *FGF20* rs591323

**Recessive model**: The forest plot and result of recessive model of rs591323 is shown below. Since there was heterogeneity observed (*I*^2^ 86%), results of random effect model were adopted. After meta-analysis to 1364 PD patients and 1487 controls, OR was 0.94 (0.53 – 1.66), *p* value: 0.835.


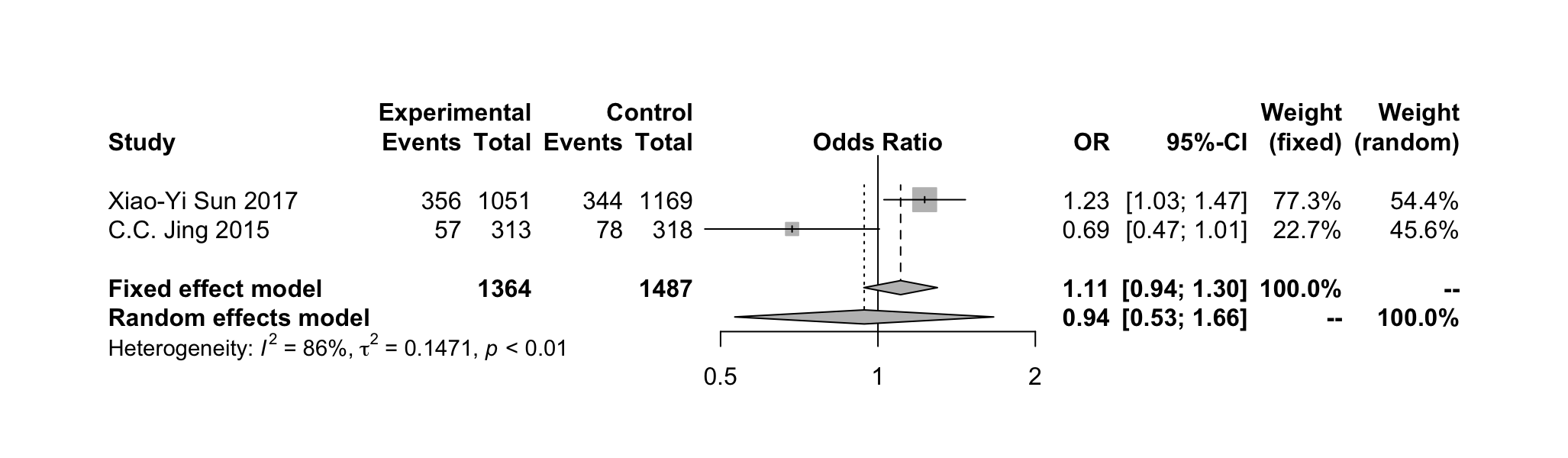


Appendix Figure 3.88 Recessive model of *FGF20* rs591323

**Overdominant model**: The forest plot and result of recessive model of rs591323 is shown below. Since there was no heterogeneity observed, results of fixed effect model were adopted. After meta-analysis to 1364 PD patients and 1487 controls, OR was 1.05 (0.91 – 1.22), *p* value: 0.510.


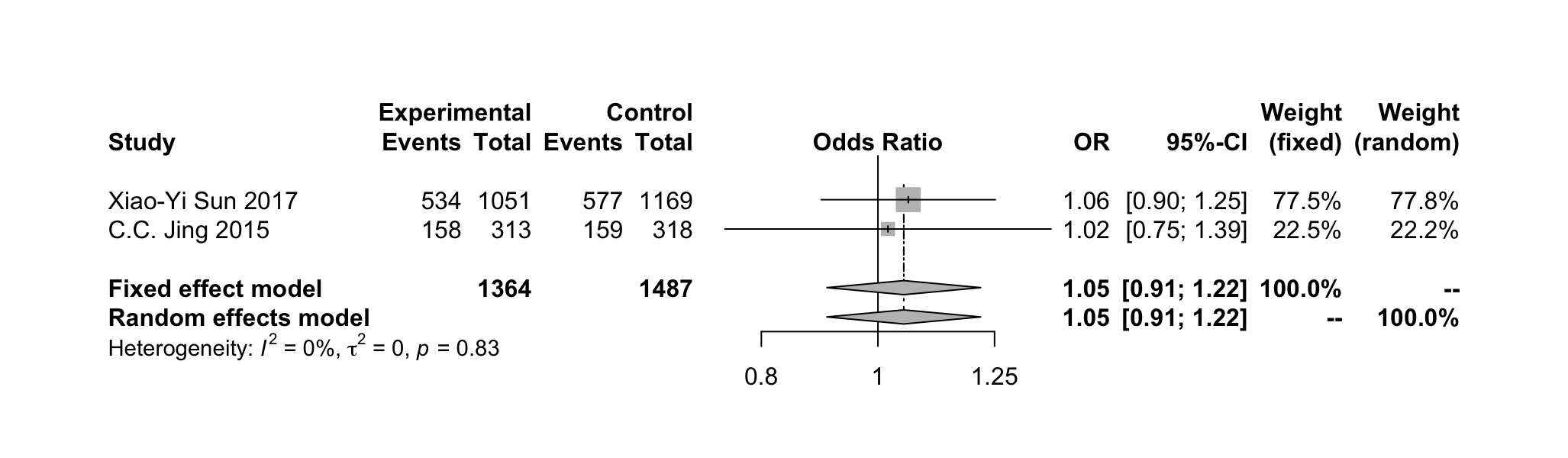


Appendix Figure 3.89 Overdominant model of *FGF20* rs591323

1. ***FGF20*, rs1721100, C>G / C>T**

| Articles | PD subjects | | | Controls | | | Methods | Location of Population |
| --- | --- | --- | --- | --- | --- | --- | --- | --- |
|  | CC | CG | GG | CC | CG | GG |  |  |
| Jing Pan et al., 2012^a,51^ | 85 | 212 | 97 | 104 | 209 | 70 | PCR | Shanghai |
| Xiaofeng Xu et al., 2013^a,52^ | 72 | 90 | 16 | 71 | 93 | 26 | PCR-RFLP | Qingdao, Shandong Province |
| Yang Lei et al., 2014^a,53^ | 30 | 71 | 35 | 44 | 68 | 20 | PCR | Dalian, Liaoning Province |
| Lamei Yuan et al., 2016^b,54^ | 123 | 262 | 127 | 120 | 260 | 132 | MassARRAY | Changsha, Hunan Province |
| PCR: Polymerase chain reaction; PD: Parkinson’s disease; RFLP: restricted fragment length polymorphism  a: diagnostic criteria: the United Kingdom brain bank criteria ^3^  b: diagnosed by neurologists | | | | | | | | |

**Allele model**: The forest plot and result of allele model of rs1721100 is shown below. We regarded allele G as risk allele. Since there was heterogeneity observed (*I*^2^ 73%), results of random effect model were adopted. After meta-analysis to 1220 PD patients and 1217 controls, OR was 1.12 (0.89 – 1.40) compared to allele C. *p* value: 0.354.


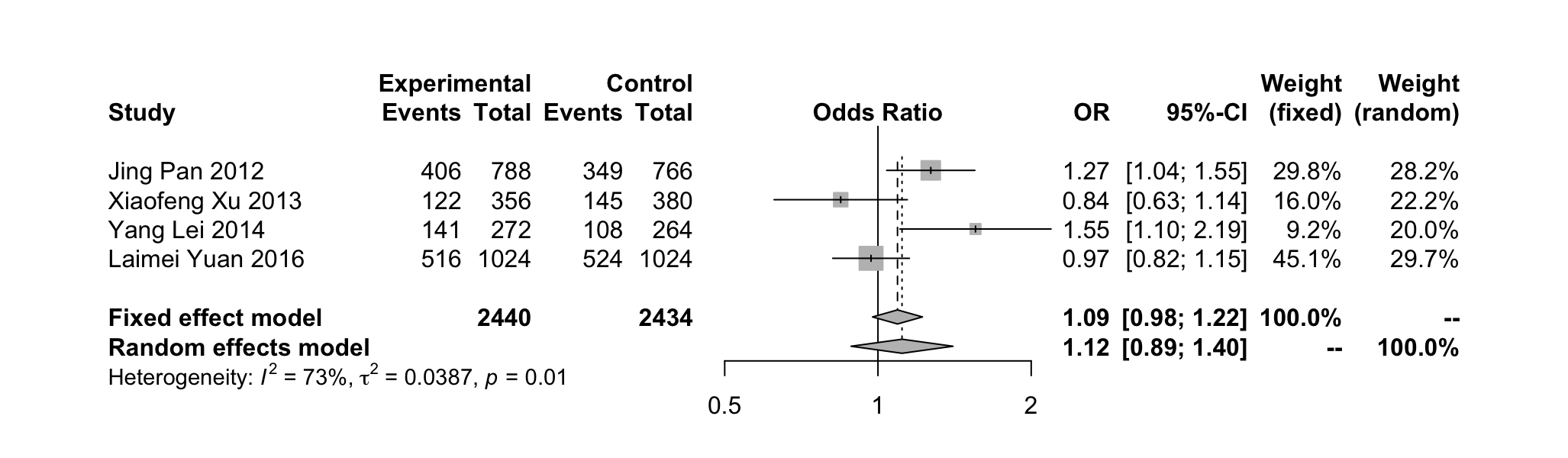


Appendix Figure 3.90 Allele model of *FGF20* rs1721100

**Dominant model**: The forest plot and result of dominant model of rs1721100 is shown below. Since there was heterogeneity observed (*I*^2^ 52%), results of random effect model were adopted. After meta-analysis to 1220 PD patients and 1217 controls, OR was 1.15 (0.88 – 1.52), *p* value: 0.308.


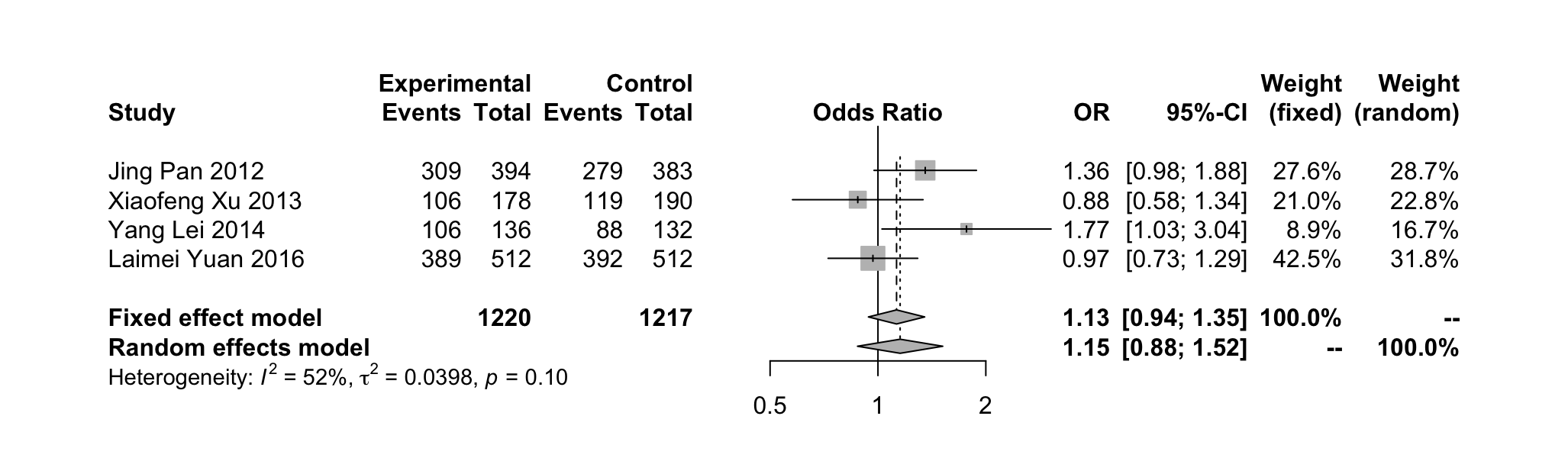


Appendix Figure 3.91 Dominant model of *FGF20* rs1721100

**Recessive model**: The forest plot and result of recessive model of rs1721100 is shown below. Since there was heterogeneity observed (*I*^2^ 69%), results of random effect model were adopted. After meta-analysis to 1220 PD patients and 1217 controls, OR was 1.15 (0.78 – 1.70), *p* value: 0.485.


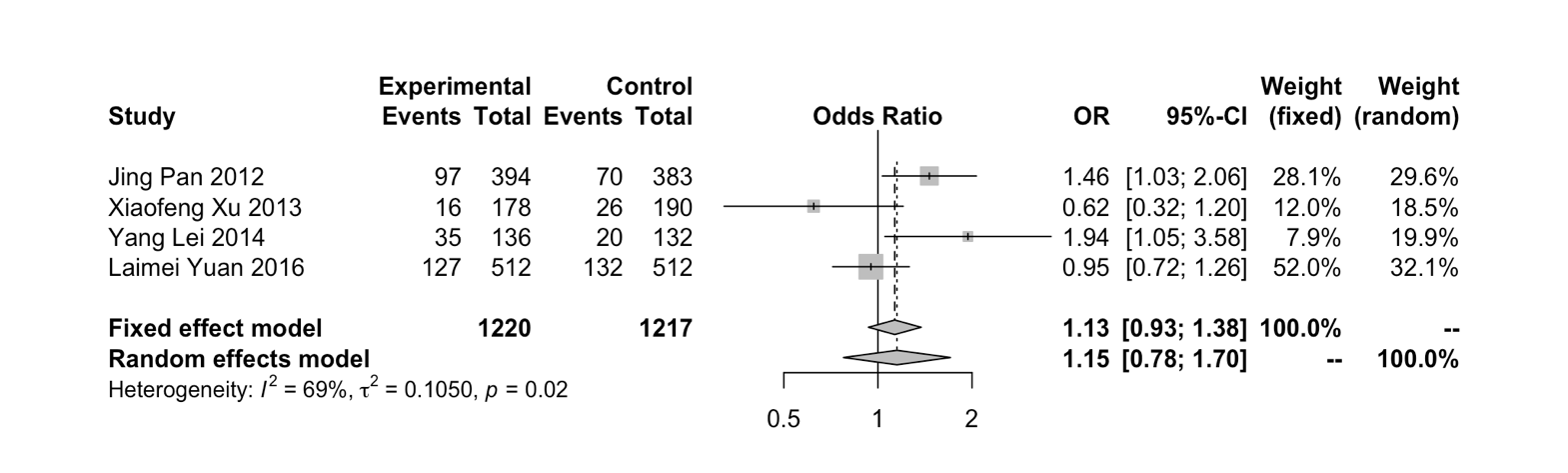


Appendix Figure 3.92 Recessive model of *FGF20* rs1721100

**Overdominant model**: The forest plot and result of recessive model of rs1721100 is shown below. Since there was no heterogeneity observed, results of fixed effect model were adopted. After meta-analysis to 1220 PD patients and 1217 controls, OR was 0.99 (0.84 – 1.16), *p* value: 0.905.


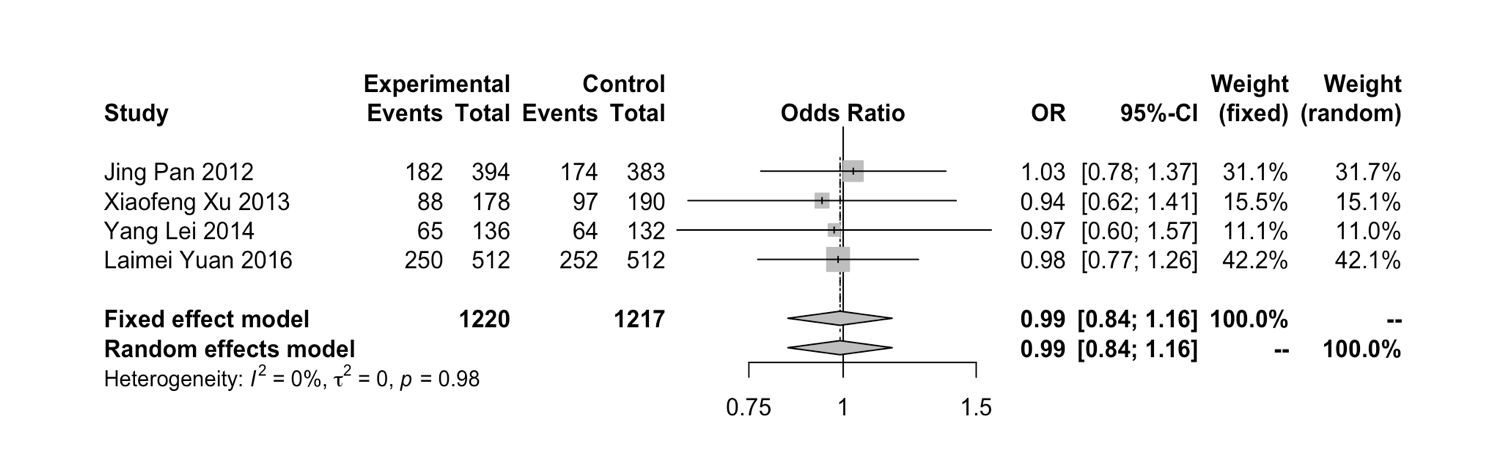


Appendix Figure 3.93 Overdominant model of *FGF20* rs1721100

1. ***FGF20,* rs1989754, G>C**

| Articles | PD subjects | | | Controls | | | Methods | Location of Population |
| --- | --- | --- | --- | --- | --- | --- | --- | --- |
|  | CC | CG | GG | CC | CG | GG |  |  |
| Yang Lei et al., 2014^a,53^ | 25 | 72 | 39 | 40 | 67 | 25 | PCR | Dalian, Liaoning Province |
| Lamei Yuan et al., 2016^b,54^ | 129 | 262 | 121 | 126 | 263 | 123 | MassARRAY | Changsha, Hunan Province |
| PCR: Polymerase chain reaction; PD: Parkinson’s disease  a: diagnostic criteria: the United Kingdom brain bank criteria ^3^  b: diagnosed by neurologists | | | | | | | | |

**Allele model**: The forest plot and result of allele model of rs1989754 is shown below. We regarded allele C as risk allele. Since there was heterogeneity observed (*I*^2^ 82%), results of random effect model were adopted. After meta-analysis to 648 PD patients and 644 controls, OR was 0.83 (0.53 – 1.30) compared to allele G. *p* value: 0.418.


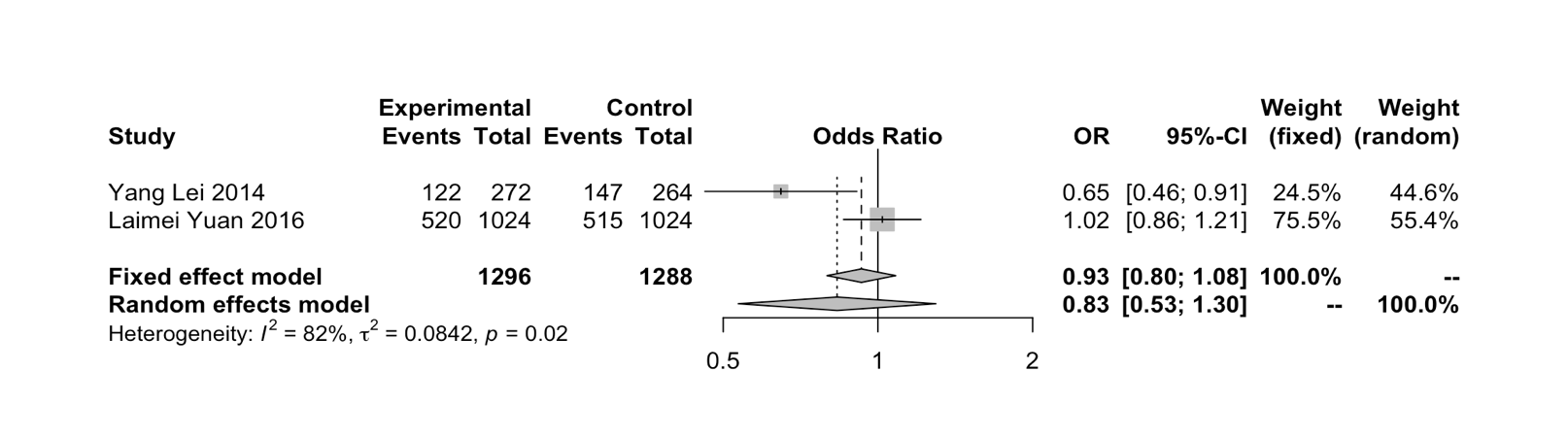


Appendix Figure 3.94 Allele model of *FGF20* rs1989754

**Dominant model**: The forest plot and result of dominant model of rs1989754 is shown below. Since there was heterogeneity observed (*I*^2^ 66%), results of random effect model were adopted. After meta-analysis to 648 PD patients and 644 controls, OR was 0.82 (0.47 – 1.40), *p* value: 0.460.


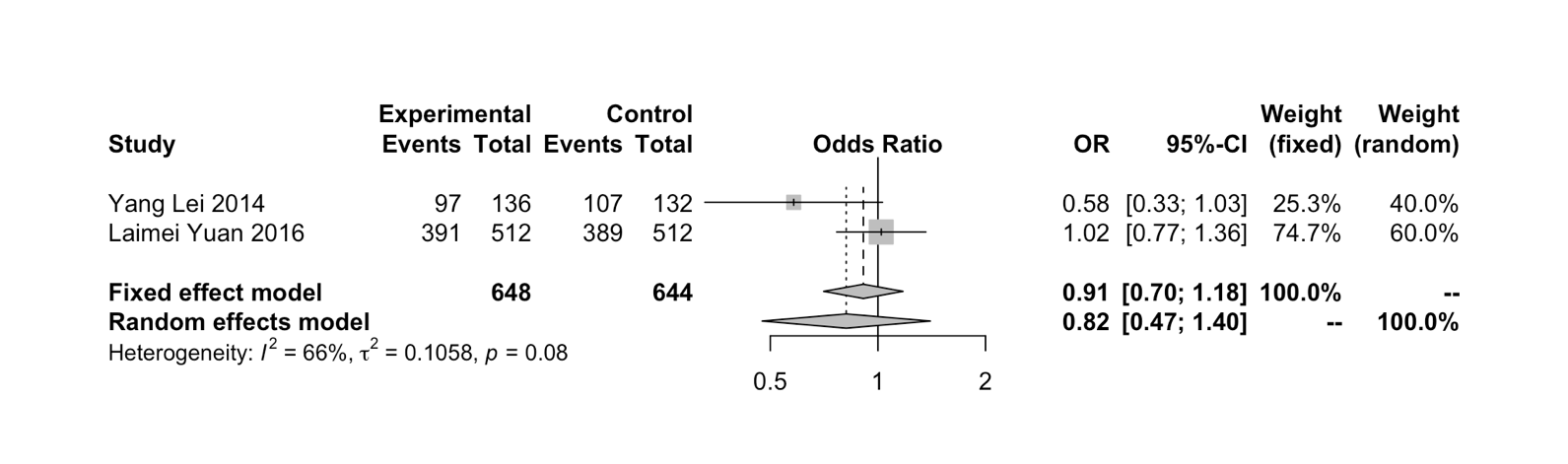


Appendix Figure 3.95 Dominant model of *FGF20* rs1989754

**Recessive model**: The forest plot and result of recessive model of rs1989754 is shown below. Since there was heterogeneity observed (*I*^2^ 78%), results of random effect model were adopted. After meta-analysis to 648 PD patients and 644 controls, OR was 0.77 (0.39 – 1.50), *p* value: 0.435.


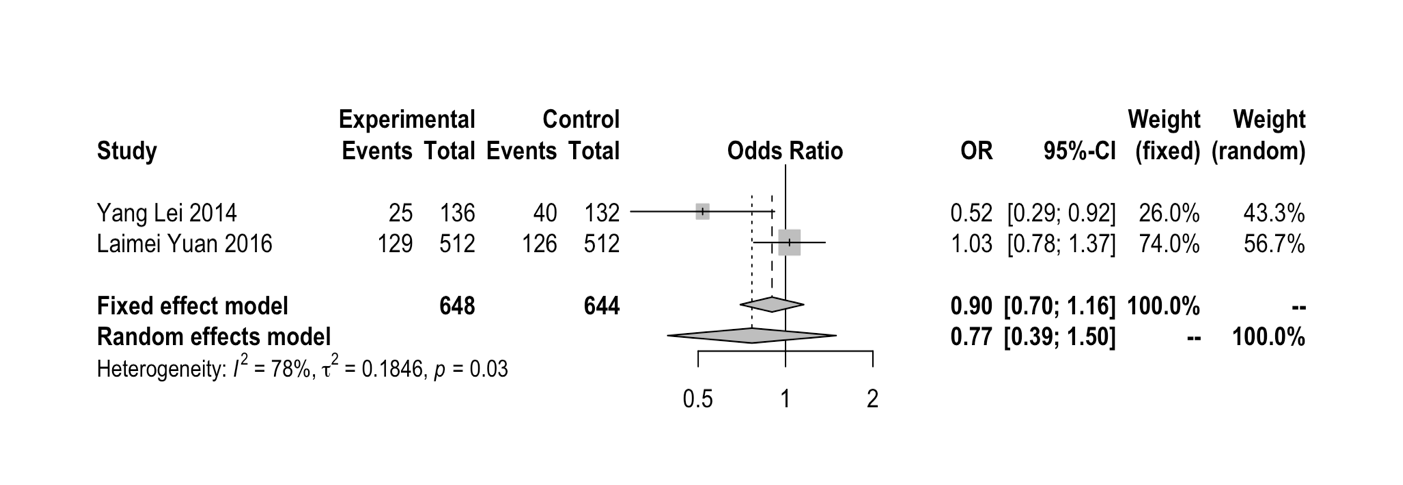


Appendix Figure 3.96 Recessive model of *FGF20* rs1989754

**Overdominant model**: The forest plot and result of recessive model of rs1989754 is shown below. Since there was no heterogeneity observed, results of fixed effect model were adopted. After meta-analysis to 648 PD patients and 644 controls, OR was 0.99 (0.79 – 1.23), *p* value: 0.915.


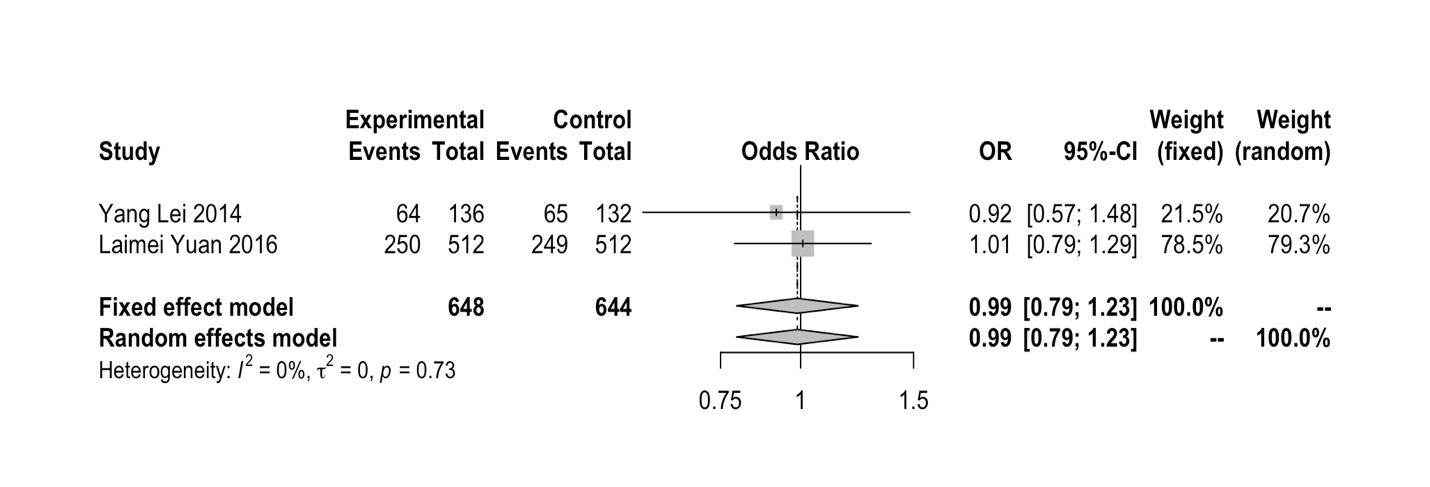


Appendix Figure 3.97 Overdominant model of *FGF20* rs1989754

1. ***FGF20*, rs12720208, C> A / G / T**

| Articles | PD subjects | | | Controls | | | Methods | Location of Population |
| --- | --- | --- | --- | --- | --- | --- | --- | --- |
|  | CC | CT | TT | CC | CT | TT |  |  |
| Jing Pan et al., 2012^a,51^ | 389 | 5 | 0 | 371 | 12 | 0 | PCR | Shanghai |
| Yang Lei et al., 2014^a,53^ | 97 | 27 | 12 | 86 | 31 | 15 | PCR | Dalian, Liaoning Province |
| PCR: Polymerase chain reaction; PD: Parkinson’s disease  a: diagnostic criteria: the United Kingdom brain bank criteria ^3^ | | | | | | | | |

**Allele model**: The forest plot and result of allele model of rs12720208 is shown below. We regarded allele T as risk allele. Since there was no heterogeneity observed, results of fixed effect model were adopted. After meta-analysis to 530 PD patients and 515 controls, OR was 0.70 (0.47 – 1.02) compared to allele C. *p* value: 0.066.


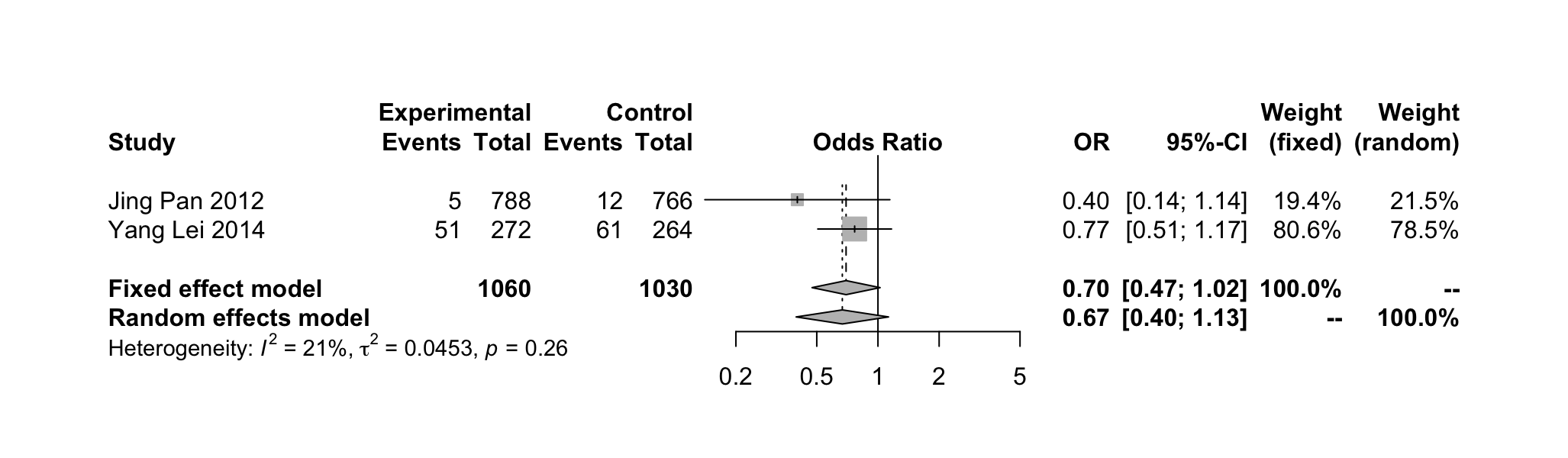


Appendix Figure 3.98 Allele model of *FGF20* rs12720208

**Dominant model**: The forest plot and result of dominant model of rs12720208 is shown below. Since there was no heterogeneity observed, results of fixed effect model were adopted. After meta-analysis to 530 PD patients and 515 controls, OR was 0.66 (0.42 – 1.04), *p* value: 0.074.


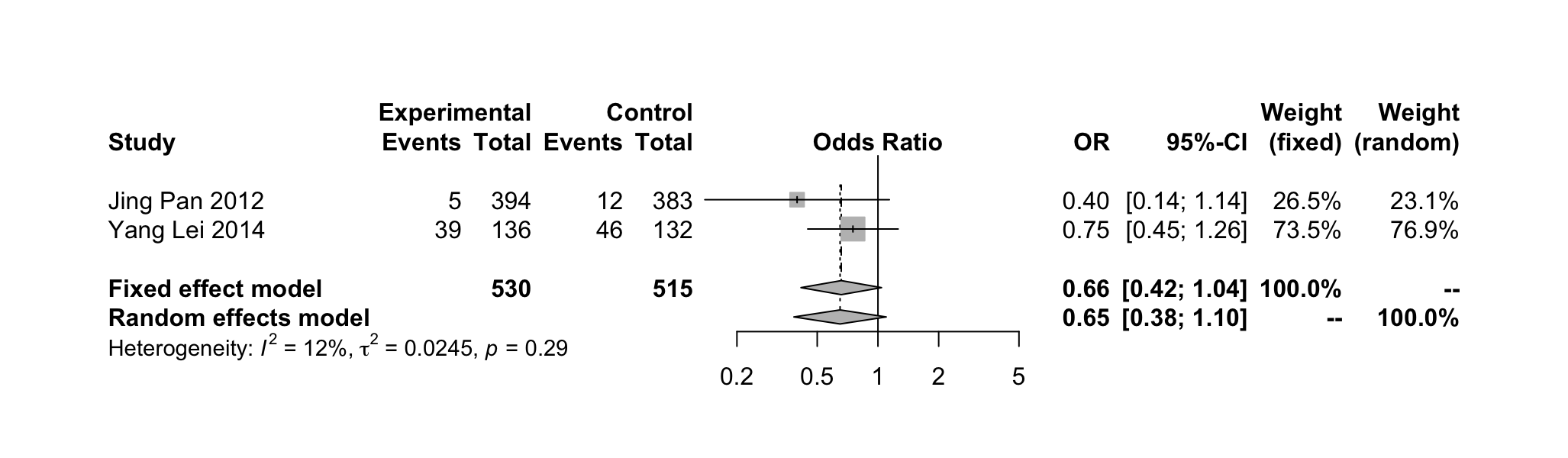


Appendix Figure 3.99 Dominant model of *FGF20* rs12720208

**Recessive model**: It was not applied to perform meta-analysis.

**Overdominant model**: The forest plot and result of recessive model of rs12720208 is shown below. Since there was no heterogeneity observed, results of fixed effect model were adopted. After meta-analysis to 530 PD patients and 515 controls, OR was 1.48 (0.90 – 2.45), *p* value: 0.126.


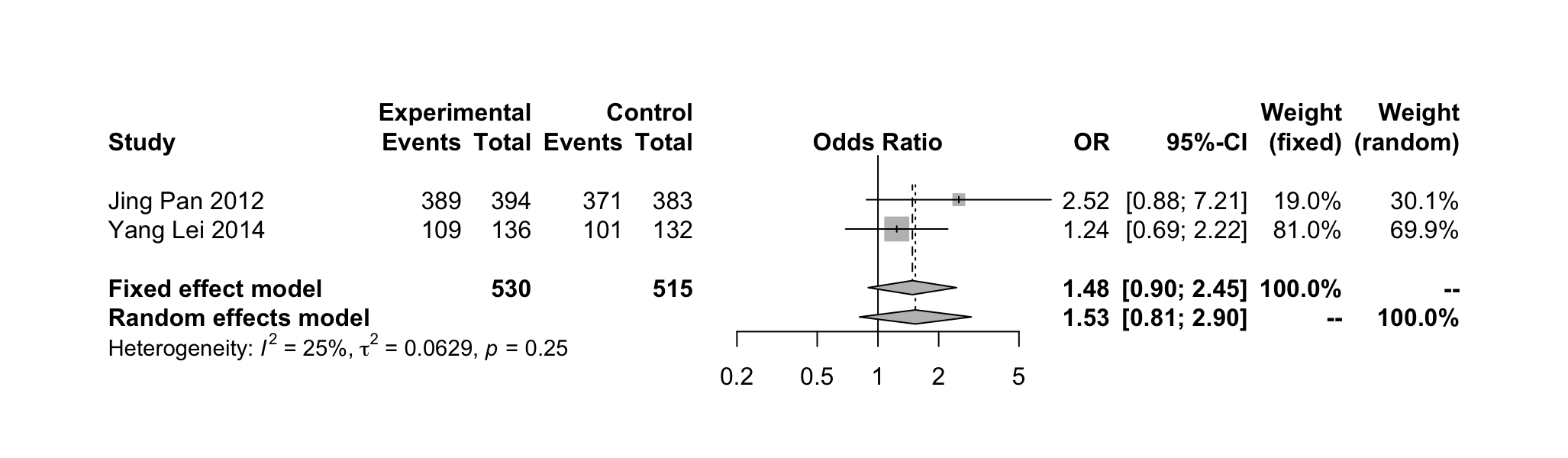


Appendix Figure 3.100 Overdominant model of *FGF20* rs12720208

1. ***GAK/PARK17*, rs11248051, C>T**

| Articles | PD subjects | | | Controls | | | Methods | Location of Population |
| --- | --- | --- | --- | --- | --- | --- | --- | --- |
|  | CC | CT | TT | CC | CT | TT |  |  |
| Li-Li Zhou et al., 2014^a,55^ | 245 | 69 | 9 | 283 | 60 | 2 | PCR | Wenzhou, Zhejiang Province |
| Ji-Feng Guo et al., 2015^a,22^ | 741 | 253 | 25 | 721 | 286 | 23 | PCR | Changsha, Hunan Province |
| PCR: Polymerase chain reaction; PD: Parkinson’s disease  a: diagnostic criteria: the United Kingdom brain bank criteria ^3^ | | | | | | | | |

**Allele model**: The forest plot and result of allele model of rs11248051 is shown below. We regarded allele T as risk allele. Since there was heterogeneity observed (*I*^2^ 86%), results of random effect model were adopted. After meta-analysis to 1342 PD patients and 1375 controls, OR was 1.15 (0.70 – 1.90) compared to allele C. *p* value: 0.586.

Appendix Figure 3.101 Allele model of *GAK/PARK17* rs11248051

**Dominant model**: The forest plot and result of dominant model of rs11248051 is shown below. Since there was heterogeneity observed (*I*^2^ 82%), results of random effect model were adopted. After meta-analysis to 1342 PD patients and 1375 controls, OR was 1.10 (0.67 – 1.80), *p* value: 0.710.

Appendix Figure 3.102 Dominant model of *GAK/PARK17* rs11248051

**Recessive model**: The forest plot and result of recessive model of rs11248051 is shown below. Since there was heterogeneity observed (*I*^2^ 69%), results of random effect model were adopted. After meta-analysis to 1342 PD patients and 1375 controls, OR was 1.95 (0.47 – 8.20), *p* value: 0.361.

Appendix Figure 3.103 Recessive model of *GAK/PARK17* rs11248051

**Overdominant model**: The forest plot and result of recessive model of rs11248051 is shown below. Since there was heterogeneity observed (*I*^2^ 71%), results of random effect model were adopted. After meta-analysis to 1342 PD patients and 1375 controls, OR was 0.98 (0.66 – 1.46), *p* value: 0.934.

Appendix Figure 3.104 Overdominant model of *GAK/PARK17* rs11248051

1. ***GAK/PARK17*, rs1564282, C>T**

| Articles | PD subjects | | | Controls | | | Methods | Location of Population |
| --- | --- | --- | --- | --- | --- | --- | --- | --- |
|  | CC | CT | TT | CC | CT | TT |  |  |
| Nan-Nan Li et al., 2012^a,56^ | 616 | 183 | 13 | 616 | 142 | 4 | MassARRAY | Chengdu, Sichuan Province |
| Yong Ping Chen et al., 2013^a,57^ | 285 | 81 | 10 | 227 | 48 | 2 | MassARRAY | Chengdu, Sichuan Province |
| Wei-En Johnny Tseng et al., 2013^a,58^ | 381 | 97 | 5 | 387 | 104 | 4 | MassARRAY | Taiwan Province |
| Ji-Feng Guo et al., 2015^a,22^ | 814 | 186 | 19 | 866 | 152 | 12 | PCR | Changsha, Hunan Province |
| Wen-Juan Yu et al., 2015a,^59^ | 385 | 132 | 12 | 331 | 89 | 1 | MassARRAY | Chengdu, Sichuan Province |
| PCR: Polymerase chain reaction; PD: Parkinson’s disease  a: diagnostic criteria: the United Kingdom brain bank criteria ^3^ | | | | | | | | |

**Allele model**: The forest plot and result of allele model of rs1564282 is shown below. We regarded allele T as risk allele. Since there was no heterogeneity observed, results of fixed effect model were adopted. After meta-analysis to 3219 PD patients and 2985 controls, OR was 1.30 (1.16 – 1.46) compared to allele C. *p* value: <0.0001.

Appendix Figure 3.105 Allele model of *GAK/PARK17* rs1564282

**Dominant model**: The forest plot and result of dominant model of rs1564282 is shown below. Since there was no heterogeneity observed, results of fixed effect model were adopted. After meta-analysis to 3219 PD patients and 2985 controls, OR was 1.28 (1.13 – 1.45), *p* value: <0.0001.

Appendix Figure 3.106 Dominant model of *GAK/PARK17* rs1564282

**Recessive model**: The forest plot and result of recessive model of rs1564282 is shown below. Since there was no heterogeneity observed, results of fixed effect model were adopted. After meta-analysis to 3219 PD patients and 2985 controls, OR was 2.41 (1.48 – 3.93), *p* value: 0.0004.

Appendix Figure 3.107 Recessive model of *GAK/PARK17* rs1564282

**Overdominant model**: The forest plot and result of recessive model of rs1564282 is shown below. Since there was no heterogeneity observed, results of fixed effect model were adopted. After meta-analysis to 3219 PD patients and 2985 controls, OR was 0.82 (0.73 – 0.94), *p* value: 0.0027.

Appendix Figure 3.108 Overdominant model of *GAK/PARK17* rs1564282

1. ***GBA,* L444P**

| Articles | PD subjects | | | Controls | | | Methods | Location of Population |
| --- | --- | --- | --- | --- | --- | --- | --- | --- |
|  | CC | CT | TT | CC | CT | TT |  |  |
| X.-Y. Mao et al., 2010^a,60^ | 0 | 20 | 596 | 0 | 1 | 410 | PCR-RFLP | Chengdu, Sichuan Province |
| Ji-Feng Guo et al., 2015^a,22^ | 0 | 26 | 993 | 0 | 1 | 1029 | PCR | Changsha, Hunan Province |
| PCR: Polymerase chain reaction; PD: Parkinson’s disease; RFLP: restricted fragment length polymorphism  a: diagnostic criteria: the United Kingdom brain bank criteria ^3^ | | | | | | | | |

**Allele model**: The forest plot and result of allele model of L444P is shown below. We regarded allele C as risk allele. Since there was no heterogeneity observed, results of fixed effect model were adopted. After meta-analysis to 1635 PD patients and 1441 controls, OR was 19.48 (4.75 – 79.93) compared to allele T. *p* value: <0.0001.

Appendix Figure 3.109 Allele model of *GBA* L444P

**Dominant model**: The forest plot and result of dominant model of L444P is shown below. Since there was no heterogeneity observed, results of fixed effect model were adopted. After meta-analysis to 1635 PD patients and 1441 controls, OR was 19.76 (4.81 – 81.16), *p* value: <0.0001.

Appendix Figure 3.110 Dominant model of *GBA* L444P

**Recessive model**: It was not applied to perform meta-analysis.

**Overdominant model**: The forest plot and result of recessive model of L444P is shown below. Since there was no heterogeneity observed, results of fixed effect model were adopted. After meta-analysis to 1635 PD patients and 1441 controls, OR was 0.05 (0.01 – 0.21), *p* value: <0.0001.

Appendix Figure 3.111 Overdominant model of *GBA* L444P

1. ***GBA*, rs2230288, E326K**

| Articles | PD subjects | | | Controls | | | Methods | Location of Population |
| --- | --- | --- | --- | --- | --- | --- | --- | --- |
|  | AA | AG | GG | AA | AG | GG |  |  |
| S. G. Ziegler et al., 2007^a, 61^ | 0 | 18 | 74 | 0 | 0 | 92 | - | Taiwan |
| Z. Yu et al., 2015^62^ | 0 | 1 | 183 | 0 | 0 | 130 | - | Taian, Shandong Province |
| PD: Parkinson’s disease  a: diagnostic criteria: the United Kingdom brain bank criteria ^3^ | | | | | | | | |

**Allele model**: The forest plot and result of allele model of rs2230288 is shown below. We regarded allele A as risk allele. Since there was heterogeneity observed (*I*^2^ 52%), results of random effect model were adopted. After meta-analysis to 276 PD patients and 222 controls, OR was 10.23 (0.47 – 221.01) compared to allele G. *p* value: 0.138.

Appendix Figure 3.112 Allele model of *GBA* rs2230288

**Dominant model**: The forest plot and result of dominant model of rs2230288 is shown below. Since there was heterogeneity observed (*I*^2^ 55%), results of random effect model were adopted. After meta-analysis to 276 PD patients and 222 controls, OR was 10.81 (0.46 – 256.66), *p* value: 0.141.

Appendix Figure 3.113 Dominant model of *GBA* rs2230288

**Recessive model**: It was not applied to perform meta-analysis.

**Overdominant model**: The forest plot and result of recessive model of rs2230288 is shown below. Since there was heterogeneity observed (*I*^2^ 55%), results of random effect model were adopted. After meta-analysis to 276 PD patients and 222 controls, OR was 0.09 (0.00 – 2.20), *p* value: 0.141.

Appendix Figure 3.114 Overdominant model of *GBA* rs2230288

1. ***GCH-1*, rs11158026, C>T**

| Articles | PD subjects | | | Controls | | | Methods | Location of Population |
| --- | --- | --- | --- | --- | --- | --- | --- | --- |
|  | CC | CT | TT | CC | CT | TT |  |  |
| Xinglong Yang et al., 2017^a,63^ | 142 | 293 | 154 | 148 | 325 | 161 | allele-specific ligation PCR | Chengdu, Sichuan Province |
| MingZou et al., 2018^a,64^ | 149 | 300 | 130 | 178 | 307 | 157 | PCR | Wenzhou, Zhejiang Province |
| PCR: Polymerase chain reaction; PD: Parkinson’s disease  a: diagnostic criteria: the United Kingdom brain bank criteria ^3^ | | | | | | | | |

**Allele model**: The forest plot and result of allele model of rs2230288 is shown below. We regarded allele T as risk allele. Since there was no heterogeneity observed, results of fixed effect model were adopted. After meta-analysis to 1168 PD patients and 1276 controls, OR was 1.00 (0.89 – 1.12) compared to allele C. *p* value: 0.997.

Appendix Figure 3.115 Allele model of *GCH-1* rs2230288

**Dominant model**: The forest plot and result of dominant model of rs2230288 is shown below. Since there was no heterogeneity observed, results of fixed effect model were adopted. After meta-analysis to 1168 PD patients and 1276 controls, OR was 1.03 (0.86 – 1.24), *p* value: 0.728.

Appendix Figure 3.116 Dominant model of *GCH-1* rs2230288

**Recessive model**: The forest plot and result of recessive model of rs2230288 is shown below. Since there was no heterogeneity observed, results of fixed effect model were adopted. After meta-analysis to 1168 PD patients and 1276 controls, OR was 0.97 (0.80 – 1.16), *p* value: 0.721.

Appendix Figure 3.117 Recessive model of *GCH-1* rs2230288

**Overdominant model**: The forest plot and result of recessive model of rs2230288 is shown below. Since there was no heterogeneity observed, results of fixed effect model were adopted. After meta-analysis to 1168 PD patients and 1276 controls, OR was 0.95 (0.81 – 1.12), *p* value: 0.542.

Appendix Figure 3.118 Overdominant model of *GCH-1* rs2230288

1. ***GSK3B*, rs334558, T>C**

| Articles | PD subjects | | | Controls | | | Methods | Location of Population |
| --- | --- | --- | --- | --- | --- | --- | --- | --- |
|  | CC | CT | TT | CC | CT | TT |  |  |
| Lan Yu et al., 2014^a,65^ | 74 | 112 | 25 | 97 | 136 | 29 | PCR-RFLP | Wuhan, Hubei Province |
| Dong-Mei Zhao et al. 2012^a, 66^ | 284 | 364 | 113 | 138 | 309 | 72 | MassARRAY | Chengdu, Sichuan Province |
| PCR: Polymerase chain reaction; PD: Parkinson’s disease; RFLP: restricted fragment length polymorphism  a: diagnostic criteria: the United Kingdom brain bank criteria ^3^ | | | | | | | | |

**Allele model**: The forest plot and result of allele model of rs334558 is shown below. We regarded allele C as risk allele. Since there was heterogeneity observed (*I*^2^ 63%), results of random effect model were adopted. After meta-analysis to 972 PD patients and 781 controls, OR was 1.10 (0.85 – 1.41) compared to allele T. *p* value: 0.4631.

Appendix Figure 3.119 Allele model of *GSK3B* rs334558

**Dominant model**: The forest plot and result of dominant model of rs334558 is shown below. Since there was no heterogeneity observed, results of fixed effect model were adopted. After meta-analysis to 972 PD patients and 781 controls, OR was 0.92 (0.70 – 1.22), *p* value: 0.579.

Appendix Figure 3.120 Dominant model of *GSK3B* rs334558

**Recessive model**: The forest plot and result of recessive model of rs334558 is shown below. Since there was heterogeneity observed (*I*^2^ 84%), results of random effect model were adopted. After meta-analysis to 972 PD patients and 781 controls, OR was 1.25 (0.71 – 2.21), *p* value: 0.439.

Appendix Figure 3.121 Recessive model of *GSK3B* rs334558

**Overdominant model**: The forest plot and result of recessive model of rs334558 is shown below. Since there was heterogeneity observed (*I*^2^ 82%), results of random effect model were adopted. After meta-analysis to 972 PD patients and 781 controls, OR was 1.26 (0.76 – 2.10), *p* value: 0.368.

Appendix Figure 3.122 Overdominant model of *GSK3B* rs334558

1. ***GPNMB*, rs156429, T>C**

| Articles | PD subjects | | | Controls | | | Methods | Location of Population |
| --- | --- | --- | --- | --- | --- | --- | --- | --- |
|  | CC | CT | TT | CC | CT | TT |  |  |
| Zhen-hua Liu et al., 2015^a,67^ | 22 | 156 | 282 | 21 | 187 | 265 | MassARRAY | Changsha, Hunan Province |
| YaQian Xu et al., 2016^a,68^ | 58 | 402 | 636 | 50 | 331 | 448 | MassARRAY | Chengdu, Sichuan Province |
| PD: Parkinson’s disease  a: diagnostic criteria: the United Kingdom brain bank criteria ^3^ | | | | | | | | |

**Allele model**: The forest plot and result of allele model of rs156429 is shown below. We regarded allele C as risk allele. Since there was no heterogeneity observed, results of fixed effect model were adopted. After meta-analysis to 1556 PD patients and 1302 controls, OR was 0.88 (0.78 – 0.99) compared to allele T. *p* value: 0.035.

Appendix Figure 3.123 Allele model of *GPNMB* rs156429

**Dominant model**: The forest plot and result of dominant model of rs156429 is shown below. Since there was no heterogeneity observed, results of fixed effect model were adopted. After meta-analysis to 1556 PD patients and 1302 controls, OR was 0.84 (0.72 – 0.97), *p* value: 0.018.

Appendix Figure 3.124 Dominant model of *GPNMB* rs156429

**Recessive model**: The forest plot and result of recessive model of rs156429 is shown below. Since there was no heterogeneity observed, results of fixed effect model were adopted. After meta-analysis to 1556 PD patients and 1302 controls, OR was 0.93 (0.67 – 1.29), *p* value: 0.651.

Appendix Figure 3.125 Recessive model of *GPNMB* rs156429

**Overdominant model**: The forest plot and result of recessive model of rs156429 is shown below. Since there was no heterogeneity observed, results of fixed effect model were adopted. After meta-analysis to 1556 PD patients and 1302 controls, OR was 1.19 (1.02 – 1.38), *p* value: 0.027.

Appendix Figure 3.126 Overdominant model of *GPNMB* rs156429

1. ***HNMT,* rs11558538, C314T, T105I**

| Articles | PD subjects | | | Controls | | | Methods | Location of Population |
| --- | --- | --- | --- | --- | --- | --- | --- | --- |
|  | CC | CT | TT | CC | CT | TT |  |  |
| Xinglong Yang et al., 2015^a,^ ^69^ | 537 | 27 | 0 | 452 | 43 | 1 | PCR-LDR | Chengdu, Sichuan Province  Guangzhou, Guangdong Province |
| Yongping Chen et al., 2018^a,70^ | 1153 | 84 | 0 | 772 | 62 | 2 | MassARRAY | Chengdu, Sichuan Province |
| LDR: ligase detection reaction; PCR: Polymerase chain reaction; PD: Parkinson’s disease  a: diagnostic criteria: the United Kingdom brain bank criteria ^3^ | | | | | | | | |

**Allele model**: The forest plot and result of allele model of rs11558538 is shown below. We regarded allele T as risk allele. Since there was heterogeneity observed (*I*^2^ 65%), results of random effect model were adopted. After meta-analysis to 1801 PD patients and 1332 controls, OR was 0.69 (0.42 - 1.12) compared to allele C. *p* value: 0.133.

Appendix Figure 3.127 Allele model of *HNMT* rs11558538

**Dominant model**: The forest plot and result of dominant model of rs11558538 is shown below. Since there was heterogeneity observed (*I*^2^ 67%), results of random effect model were adopted. After meta-analysis to 1801 PD patients and 1332 controls, OR was 0.70 (0.41 -1.17), *p* value: 0.169.

Appendix Figure 3.128 Dominant model of *HNMT* rs11558538

**Recessive model**: The forest plot and result of recessive model of rs11558538 is shown below. Since there was no heterogeneity observed, results of fixed effect model were adopted. After meta-analysis to 1801 PD patients and 1332 controls, OR was 0.19 (0.02 – 1.68), *p* value: 0.136.

Appendix Figure 3.129 Recessive model of *HNMT* rs11558538

**Overdominant model**: The forest plot and result of recessive model of rs11558538 is shown below. Since there was heterogeneity observed (*I*^2^ 68%), results of random effect model were adopted. After meta-analysis to 1801 PD patients and 1332 controls, OR was 1.40 (0.82 – 2.36), *p* value: 0.214.

Appendix Figure 3.130 Overdominant model of *HNMT* rs11558538

1. ***HLA-DRA*, rs3129882, G>A**

| Articles | PD subjects | | | Controls | | | Methods | Location of Population |
| --- | --- | --- | --- | --- | --- | --- | --- | --- |
|  | AA | AG | GG | AA | AG | GG |  |  |
| Yi Guo et al., 2011^a,71^ | 30 | 144 | 110 | 16 | 118 | 124 | PCR | Changsha, Hunan Province |
| Chiang HL et al., 2012^72^ | 66 | 248 | 224 | 76 | 240 | 216 | PCR-RFLP | Taiwan |
| Lin CH et al., 2013^73^ | 55 | 195 | 198 | 69 | 196 | 187 | TaqMan | Taiwan |
| Li-Li Zhou et al., 2014^b,55^ | 45 | 126 | 152 | 34 | 125 | 186 | PCR | Wenzhou, Zhejiang Province |
| Ji-Feng Guo et al., 2015^b,22^ | 157 | 470 | 392 | 162 | 481 | 387 | PCR | Changsha, Hunan Province |
| Mingshu Mo et al., 2015^b,^ ^74^ | 60 | 240 | 242 | 73 | 324 | 277 | PCR | Guangzhou, Guangdong Province |
| PCR: Polymerase chain reaction; PD: Parkinson’s disease; RFLP: restricted fragment length polymorphism  a: diagnostic criteria brought up by Jankovic et al.^75^  b: diagnostic criteria: the United Kingdom brain bank criteria ^3^ | | | | | | | | |

**Allele model**: The forest plot and result of allele model of rs3129882 is shown below. We regarded allele A as risk allele. Since there was heterogeneity observed (*I*^2^ 63%), results of random effect model were adopted. After meta-analysis to 3154 PD patients and 3291 controls, OR was 1.02 (0.90 – 1.16) compared to allele G. *p* value: 0.705.

Appendix Figure 3.131 Allele model of *HLA-DRA* rs3129882

**Dominant model**: The forest plot and result of dominant model of rs3129882 is shown below. Since there was heterogeneity observed (*I*^2^ 52%), results of random effect model were adopted. After meta-analysis to 3154 PD patients and 3291 controls, OR was 1.02 (0.88 – 1.19), *p* value: 0.768.

Appendix Figure 3.132 Dominant model of *HLA-DRA* rs3129882

**Recessive model**: The forest plot and result of recessive model of rs3129882 is shown below. Since there was no heterogeneity observed, results of fixed effect model were adopted. After meta-analysis to 3154 PD patients and 3291 controls, OR was 1.00 (0.86 - 1.15), *p* value: 0.979.

Appendix Figure 3.133 Recessive model of *HLA-DRA* rs3129882

**Overdominant model**: The forest plot and result of recessive model of rs3129882 is shown below. Since there was no heterogeneity observed, results of fixed effect model were adopted. After meta-analysis to 3154 PD patients and 3291 controls, OR was 1.00 (0.91 – 1.10), *p* value: 0.999.

Appendix Figure 3.134 Overdominant model of *HLA-DRA* rs3129882

1. ***IL-10*, −819 T/C**

| Articles | PD subjects | | | Controls | | | Methods | Location of Population |
| --- | --- | --- | --- | --- | --- | --- | --- | --- |
|  | CC | CT | TT | CC | CT | TT |  |  |
| Dequan Li et al., 2012^a,76^ | 31 | 161 | 163 | 30 | 78 | 92 | PCR | Qingdao, Shandong Province |
| Zhenhua Liu et al., 2016^b,77^ | 51 | 169 | 240 | 47 | 211 | 215 | MassARRAY | Changsha, Hunan Province |
| PCR: Polymerase chain reaction; PD: Parkinson’s disease  a: diagnostic criteria brought up by Calne et al.^78^  b: diagnostic criteria: the United Kingdom brain bank criteria ^3^ | | | | | | | | |

**Allele model**: The forest plot and result of allele model of -819T/C is shown below. We regarded allele C as risk allele. Since there was no heterogeneity observed, results of fixed effect model were adopted. After meta-analysis to 815 PD patients and 673 controls, OR was 0.87 (0.75 – 1.02) compared to allele T. *p* value: 0.094.

Appendix Figure 3.135 Allele model of *IL-10* -819T/C

**Dominant model**: The forest plot and result of dominant model of -819T/C is shown below. Since there was no heterogeneity observed, results of fixed effect model were adopted. After meta-analysis to 815 PD patients and 673 controls, OR was 0.84 (0.68 – 1.03), *p* value: 0.101.

Appendix Figure 3.136 Dominant model of *IL-10* -819T/C

**Recessive model**: The forest plot and result of recessive model of -819T/C is shown below. Since there was heterogeneity observed (*I*^2^ 78%), results of random effect model were adopted. After meta-analysis to 815 PD patients and 673 controls, OR was 0.80 (0.39 – 1.64), *p* value: 0.539.

Appendix Figure 3.137 Recessive model of *IL-10* -819T/C

**Overdominant model**: The forest plot and result of recessive model of -819T/C is shown below. Since there was heterogeneity observed (*I*^2^ 85%), results of random effect model were adopted. After meta-analysis to 815 PD patients and 673 controls, OR was 1.05 (0.59 – 1.86), *p* value: 0.877.

Appendix Figure 3.138 Overdominant model of *IL-10* -819T/C

1. ***IL18*, rs187238，-137G/C**

| Articles | PD subjects | | | Controls | | | Methods | Location of Population |
| --- | --- | --- | --- | --- | --- | --- | --- | --- |
|  | CC | CG | GG | CC | CG | GG |  |  |
| Xiang Xu et al., 2011^a, 79^ | 11 | 69 | 210 | 5 | 53 | 142 | SSP-PCR | Qingdao, Shandong Province; Beijing |
| Zhenhua Liu et al., 2016^b,77^ | 10 | 101 | 349 | 4 | 105 | 364 | MassARRAY | Changsha, Hunan Province |
| PCR: polymerase chain reaction; PD: Parkinson’s disease; SSP: sequence-specific primers  a: diagnosed by neurologists  b: diagnostic criteria: the United Kingdom brain bank criteria ^3^ | | | | | | | | |

**Allele model**: The forest plot and result of allele model of rs187238 is shown below. We regarded allele C as risk allele. Since there was no heterogeneity observed, results of fixed effect model were adopted. After meta-analysis to 750 PD patients and 673 controls, OR was 1.07 (0.86 – 1.33) compared to allele G. *p* value: 0.546.

Appendix Figure 3.139 Allele model of *IL18* rs187238

**Dominant model**: The forest plot and result of dominant model of rs187238 is shown below. Since there was no heterogeneity observed, results of fixed effect model were adopted. After meta-analysis to 750 PD patients and 673 controls, OR was 1.01 (0.80 – 1.29), *p* value: 0.916.

Appendix Figure 3.140 Dominant model of *IL18* rs187238

**Recessive model**: The forest plot and result of recessive model of rs187238 is shown below. Since there was no heterogeneity observed, results of fixed effect model were adopted. After meta-analysis to 750 PD patients and 673 controls, OR was 1.97 (0.90 – 4.33), *p* value: 0.092.

Appendix Figure 3.141 Recessive model of *IL18* rs187238

**Overdominant model**: The forest plot and result of recessive model of rs187238 is shown below. Since there was no heterogeneity observed, results of fixed effect model were adopted. After meta-analysis to 750 PD patients and 673 controls, OR was 1.06 (0.83 – 1.36), *p* value: 0.633.

Appendix Figure 3.142 Overdominant model of *IL18* rs187238

1. ***LINGO1*, rs9652490, A>G**

| Articles | PD subjects | | | Controls | | | Methods | Location of Population |
| --- | --- | --- | --- | --- | --- | --- | --- | --- |
|  | AA | AG | GG | AA | AG | GG |  |  |
| Xing Zuo et al., 2010^a,80^ | 274 | 105 | 21 | 255 | 133 | 22 | PCR | Changsha, Hunan Province |
| Yi Guo et al., 2011^a,81^ | 168 | 79 | 12 | 192 | 83 | 10 | PCR | Changsha, Hunan Province |
| Yongping Chen et al., 2015^a, 82^ | 656 | 354 | 45 | 495 | 278 | 37 | MassARRAY | Chengdu, Sichuan Province |
| PCR: Polymerase chain reaction; PD: Parkinson’s disease  a: diagnostic criteria: the United Kingdom brain bank criteria ^3^ | | | | | | | | |

**Allele model**: The forest plot and result of allele model of rs9652490 is shown below. We regarded allele G as risk allele. Since there was no heterogeneity observed, results of fixed effect model were adopted. After meta-analysis to 1714 PD patients and 1505 controls, OR was 0.95 (0.84 – 1.07) compared to allele A. *p* value: 0.374.

Appendix Figure 3.143 Allele model of *LINGO1* rs9652490

**Dominant model**: The forest plot and result of dominant model of rs9652490 is shown below. Since there was no heterogeneity observed, results of fixed effect model were adopted. After meta-analysis to 1714 PD patients and 1505 controls, OR was 0.93 (0.80 – 1.07), *p* value: 0.294.

Appendix Figure 3.144 Dominant model of *LINGO1* rs9652490

**Recessive model**: The forest plot and result of recessive model of rs9652490 is shown below. Since there was no heterogeneity observed, results of fixed effect model were adopted. After meta-analysis to 1714 PD patients and 1505 controls, OR was 1.00 (0.72 – 1.39), *p* value: 0.987.

Appendix Figure 3.145 Recessive model of *LINGO1* rs9652490

**Overdominant model**: The forest plot and result of recessive model of rs9652490 is shown below. Since there was no heterogeneity observed, results of fixed effect model were adopted. After meta-analysis to 1714 PD patients and 1505 controls, OR was 1.09 (0.94 – 1.26), *p* value: 0.282.

Appendix Figure 3.146 Overdominant model of *LINGO1* rs9652490

1. ***LINGO1*, rs11856808, C>T**

| Articles | PD subjects | | | Controls | | | Methods | Location of Population |
| --- | --- | --- | --- | --- | --- | --- | --- | --- |
|  | CC | CT | TT | CC | CT | TT |  |  |
| Xing Zuo et al., 2010^a,80^ | 197 | 173 | 30 | 185 | 178 | 47 | PCR | Changsha, Hunan Province |
| Yongping Chen et al., 2015^a, 82^ | 499 | 433 | 123 | 329 | 376 | 105 | MassARRAY | Chengdu, Sichuan Province |
| PCR: Polymerase chain reaction; PD: Parkinson’s disease  a: diagnostic criteria: the United Kingdom brain bank criteria ^3^ | | | | | | | | |

**Allele model**: The forest plot and result of allele model of rs11856808 is shown below. We regarded allele T as risk allele. Since there was no heterogeneity observed, results of fixed effect model were adopted. After meta-analysis to 1455 PD patients and 1220 controls, OR was 0.83 (0.74 – 0.94) compared to allele C. *p* value: 0.002.

Appendix Figure 3.147 Allele model of *LINGO1* rs11856808

**Dominant model**: The forest plot and result of dominant model of rs11856808 is shown below. Since there was no heterogeneity observed, results of fixed effect model were adopted. After meta-analysis to 1455 PD patients and 1220 controls, OR was 0.79 (0.68 – 0.92), *p* value: 0.002.

Appendix Figure 3.148 Dominant model of *LINGO1* rs11856808

**Recessive model**: The forest plot and result of recessive model of rs11856808 is shown below. Since there was no heterogeneity observed, results of fixed effect model were adopted. After meta-analysis to 1455 PD patients and 1220 controls, OR was 0.81 (0.64 – 1.03), *p* value: 0.086.

Appendix Figure 3.149 Recessive model of *LINGO1* rs11856808

**Overdominant model**: The forest plot and result of recessive model of rs11856808 is shown below. Since there was no heterogeneity observed, results of fixed effect model were adopted. After meta-analysis to 1455 PD patients and 1220 controls, OR was 1.17 (1.00 – 1.36), *p* value: 0.050.

Appendix Figure 3.150 Overdominant model of *LINGO1* rs11856808

1. ***LINGO2*, rs7033345, T>C**

| Articles | PD subjects | | | Controls | | | Methods | Location of Population |
| --- | --- | --- | --- | --- | --- | --- | --- | --- |
|  | CC | CT | TT | CC | CT | TT |  |  |
| Yi-Wen Wu et al., 2011^a,83^ | 48 | 120 | 76 | 46 | 152 | 109 | MassARRAY | Shanghai |
| Yongping Chen et al., 2015^a, 82^ | 199 | 507 | 349 | 132 | 415 | 263 | MassARRAY | Chengdu, Sichuan Province |
| PD: Parkinson’s disease  a: diagnostic criteria: the United Kingdom brain bank criteria ^3^ | | | | | | | | |

**Allele model**: The forest plot and result of allele model of rs7033345 is shown below. We regarded allele C as risk allele. Since there was no heterogeneity observed, results of fixed effect model were adopted. After meta-analysis to 1299 PD patients and 1117 controls, OR was 1.08 (0.96 – 1.21) compared to allele T. *p* value: 0.212.

Appendix Figure 3.151 Allele model of *LINGO2* rs7033345

**Dominant model**: The forest plot and result of dominant model of rs7033345 is shown below. Since there was no heterogeneity observed, results of fixed effect model were adopted. After meta-analysis to 1299 PD patients and 1117 controls, OR was 1.02 (0.86 – 1.22), *p* value: 0.785.

Appendix Figure 3.152 Dominant model of *LINGO2* rs7033345

**Recessive model**: The forest plot and result of recessive model of rs7033345 is shown below. Since there was no heterogeneity observed, results of fixed effect model were adopted. After meta-analysis to 1299 PD patients and 1117 controls, OR was 1.24 (1.00 – 1.53), *p* value: 0.051.

Appendix Figure 3.153 Recessive model of *LINGO2* rs7033345

**Overdominant model**: The forest plot and result of recessive model of rs7033345 is shown below. Since there was no heterogeneity observed, results of fixed effect model were adopted. After meta-analysis to 1299 PD patients and 1117 controls, OR was 1.11 (0.94 – 1.30), *p* value: 0.218.

Appendix Figure 3.154 Overdominant model of *LINGO2* rs7033345

1. ***LRRK2*, rs34594498, A419V, C1256T**

| Articles | PD subjects | | | Controls | | | Methods | Location of Population |
| --- | --- | --- | --- | --- | --- | --- | --- | --- |
|  | CC | CT | TT | CC | CT | TT |  |  |
| Di Fonzo A et al., 2006^84^ | 582 | 10 | 0 | 341 | 3 | 0 | PCR | Taiwan |
| Nan-Nan Li et al., 2012^a,85^ | 707 | 22 | 0 | 581 | 4 | 0 | PCR | Chengdu, Sichuan Province |
| Kai Li et al., 2015^a, 86^ | 482 | 18 | 0 | 565 | 9 | 0 | MassARRAY | Changsha, Hunan Province |
| PCR: Polymerase chain reaction; PD: Parkinson’s disease  a: diagnostic criteria: the United Kingdom brain bank criteria ^3^ | | | | | | | | |

**Allele model**: The forest plot and result of allele model of rs34594498 is shown below. We regarded allele T as risk allele. Since there was no heterogeneity observed, results of fixed effect model were adopted. After meta-analysis to 1821 PD patients and 1503 controls, OR was 2.81 (1.59 – 4.97) compared to allele C. *p* value: 0.0004.

Appendix Figure 3.155 Allele model of *LRRK2* rs34594498

**Dominant model**: The forest plot and result of dominant model of rs34594498 is shown below. Since there was no heterogeneity observed, results of fixed effect model were adopted. After meta-analysis to 1821 PD patients and 1503 controls, OR was 2.83 (1.60 – 5.03), *p* value: 0.0004.

Appendix Figure 3.156 Dominant model of *LRRK2* rs34594498

**Recessive model**: It was not applied to perform meta-analysis.

**Overdominant model**: The forest plot and result of recessive model of rs34594498 is shown below. Since there was no heterogeneity observed, results of fixed effect model were adopted. After meta-analysis to 1821 PD patients and 1503 controls, OR was 0.35 (0.20 – 0.63), *p* value: 0.0004.

Appendix Figure 3.157 Overdominant model of *LRRK2* rs34594498

1. ***LRRK2*, rs34410987, P755L, C2246T**

| Articles | PD subjects | | | Controls | | | Methods | Location of Population |
| --- | --- | --- | --- | --- | --- | --- | --- | --- |
|  | CC | CT | TT | CC | CT | TT |  |  |
| Di Fonzo A et al., 2006^84^ | 578 | 7 | 0 | 339 | 10 | 0 | PCR | Taiwan |
| Lingyan Yao et al., 2011ª^,87^ | 397 | 4 | 0 | 396 | 2 | 0 | PCR-RFLP | Changsha, Hunan Province |
| Yah-Huei Wu-Chou et al., 2013^a,88^ | 358 | 161 | 0 | 319 | 115 | 0 | TaqMan | Taiwan |
| PCR: Polymerase chain reaction; PD: Parkinson’s disease; RFLP: restricted fragment length polymorphism  a: diagnostic criteria: the United Kingdom brain bank criteria ^3^ | | | | | | | | |

**Allele model**: The forest plot and result of allele model of rs34410987 is shown below. We regarded allele T as risk allele. Since there was heterogeneity observed (*I*^2^ 58%), results of random effect model were adopted. After meta-analysis to 1505 PD patients and 1181 controls, OR was 0.94 (0.43 – 2.05) compared to allele C. *p* value: 0.875.

Appendix Figure 3.158 Allele model of *LRRK2* rs34410987

**Dominant model**: The forest plot and result of dominant model of rs34410987 is shown below. Since there was heterogeneity observed (*I*^2^ 60%), results of random effect model were adopted. After meta-analysis to 1505 PD patients and 1181 controls, OR was 0.95 (0.42 – 2.15), *p* value: 0.907.

Appendix Figure 3.159 Dominant model of *LRRK2* rs34410987

**Recessive model**: It was not applied to perform meta-analysis.

**Overdominant model**: The forest plot and result of recessive model of rs34410987 is shown below. Since there was heterogeneity observed (*I*^2^ 60%), results of random effect model were adopted. After meta-analysis to 1505 PD patients and 1181 controls, OR was 1.05 (0.47 – 2.36), *p* value: 0.907.

Appendix Figure 3.160 Overdominant model of *LRRK2* rs34410987

1. ***LRRK2,* rs34778348, G2385R, c.7153G>A**

| Articles | PD subjects | | | Controls | | | Methods | Location of Population |
| --- | --- | --- | --- | --- | --- | --- | --- | --- |
|  | AA | AG | GG | AA | AG | GG |  |  |
| Fung HC et al., 2006^a,89^ | 0 | 27 | 278 | 0 | 1 | 175 | PCR | Taiwan |
| Di Fonzo A et al., 2006^84^ | 0 | 61 | 547 | 0 | 18 | 355 | PCR | Taiwan |
| Li C et al., 2007^90^ | 0 | 5 | 128 | 0 | 0 | 214 | PCR-RFLP | Shanghai |
| X.-K. An et al., 2008^a,91^ | 1 | 70 | 529 | 0 | 11 | 323 | PCR-RFLP | Beijing |
| Yishu Zhou et al., 2012^b,92^ | 1 | 25 | 176 | 1 | 11 | 200 | PCR-RFLP | Shenyang, Liaoning province |
| Jia Liu et al., 2012^a,18^ | 2 | 62 | 400 | 0 | 26 | 523 | TaqMan | Beijing |
| Huiru Yan et al., 2012^a,93^ | 0 | 20 | 163 | 0 | 6 | 174 | PCR-RFLP | Urumqi, Xinjiang Province |
| Huiru Yan et al., 2012^a, *,93^ | 0 | 1 | 170 | 0 | 1 | 159 | PCR-RFLP | Urumqi, Xinjiang Province |
| Yah-Huei Wu-Chou et al., 2013^a,88^ | 0 | 78 | 863 | 0 | 37 | 581 | TaqMan | Taiwan |
| Qilin Ma et al., 2013^a,94^ | 0 | 24 | 213 | 0 | 4 | 186 | PCR-RFLP | Xiamen, Fujian Province |
| Xiaoli Fu et al., 2013^a,95^ | 1 | 36 | 409 | 0 | 16 | 387 | PCR-RFLP | Guangzhou, Guangdong Province |
| Ji-Feng Guo et al., 2015^a,22^ | 9 | 88 | 923 | 7 | 52 | 972 | PCR | Changsha, Hunan Province |
| PCR: Polymerase chain reaction; PD: Parkinson’s disease; RFLP: restricted fragment length polymorphism  a: diagnostic criteria: the United Kingdom brain bank criteria ^3^  b: diagnostic criteria: presenting at least two of the three cardinal signs for PD (tremor, rigidity, and bradykinesia), and had a positive response to levodopa therapy  *: population source was from Uygur ethnicity. We did not include this study into meta-analysis due to different ethnicity. | | | | | | | | |

**Allele model**: The forest plot and result of allele model of rs34778348 is shown below. We regarded allele A as risk allele. Since there was heterogeneity observed (*I*^2^ 56%), results of random effect model were adopted. After meta-analysis to 5139 PD patients and 4280 controls, OR was 2.49 (1.86 – 3.33) compared to allele G. *p* value: <0.0001.

Appendix Figure 3.161 Allele model of *LRRK2* rs34778348

**Dominant model**: The forest plot and result of dominant model of rs34778348 is shown below. Since there was heterogeneity observed (*I*^2^ 55%), results of random effect model were adopted. After meta-analysis to 5139 PD patients and 4280 controls, OR was 2.58 (1.92 – 3.46), *p* value: <0.0001.

Appendix Figure 3.162 Dominant model of *LRRK2* rs34778348

**Recessive model**: The forest plot and result of recessive model of rs34778348 is shown below. Since there was no heterogeneity observed, results of fixed effect model were adopted. After meta-analysis to 5139 PD patients and 4280 controls, OR was 1.60 (0.71 – 3.61), *p* value: 0.255. Some studies were fail to calculate because of absence of mutant homozygote.

Appendix Figure 3.163 Recessive model of *LRRK2* rs34778348

**Overdominant model**: The forest plot and result of recessive model of rs34778348 is shown below. Since there was heterogeneity observed (*I*^2^ 52%), results of random effect model were adopted. After meta-analysis to 5139 PD patients and 4280 controls, OR was 0.39 (0.29 – 0.52), *p* value: <0.0001.

Appendix Figure 3.164 Overdominant model of *LRRK2* rs34778348

1. ***LRRK2*, rs33949390, R1628P, G>C**

| Articles | PD subjects | | | Controls | | | Methods | Location of Population |
| --- | --- | --- | --- | --- | --- | --- | --- | --- |
|  | CC | CG | GG | CC | CG | GG |  |  |
| Lihua Yu et al., 2009^a,96^ | 0 | 17 | 311 | 0 | 6 | 294 | PCR | Chengdu, Sichuan Province; Guangzhou, Guangdong Province |
| Zijuan Zhang et al., 2009^a,97^ | 3 | 40 | 557 | 0 | 11 | 448 | PCR-RFLP | Chengdu, Sichuan Province |
| Yishu Zhou et al., 2012^b,92^ | 0 | 2 | 200 | 0 | 5 | 207 | PCR-RFLP | Shenyang, Liaoning province |
| Yah-Huei Wu-Chou et al., 2013^a,88^ | 2 | 56 | 689 | 0 | 18 | 443 | TaqMan | Taiwan |
| Xiaoli Fu et al., 2013^a,95^ | 0 | 47 | 399 | 0 | 21 | 382 | PCR-RFLP | Guangzhou, Guangdong Province |
| Ji-Feng Guo et al., 2015^a,22^ | 2 | 50 | 967 | 0 | 47 | 983 | PCR | Changsha, Hunan Province |
| PCR: Polymerase chain reaction; PD: Parkinson’s disease; RFLP: restricted fragment length polymorphism  a: diagnostic criteria: the United Kingdom brain bank criteria ^3^  b: diagnostic criteria: presenting at least two of the three cardinal signs for PD (tremor, rigidity, and bradykinesia), and had a positive response to levodopa therapy | | | | | | | | |

**Allele model**: The forest plot and result of allele model of rs33949390 is shown below. We regarded allele C as risk allele. Since there was heterogeneity observed (*I*^2^ 59%), results of random effect model were adopted. After meta-analysis to 3342 PD patients and 2865 controls, OR was 1.85 (1.23 – 2.80) compared to allele G. *p* value: 0.0033.

Appendix Figure 3.165 Allele model of *LRRK2* rs33949390

**Dominant model**: The forest plot and result of dominant model of rs33949390 is shown below. Since there was heterogeneity observed (*I*^2^ 59%), results of random effect model were adopted. After meta-analysis to 3342 PD patients and 2865 controls, OR was 1.83 (1.20 – 2.78), *p* value: 0.0047.

Appendix Figure 3.166 Dominant model of *LRRK2* rs33949390

**Recessive model**: The forest plot and result of recessive model of rs33949390 is shown below. Since there was no heterogeneity observed, results of fixed effect model were adopted. After meta-analysis to 3342 PD patients and 2865 controls, OR was 4.45 (0.78 – 25.26), *p* value: 0.092.

Appendix Figure 3.167 Recessive model of *LRRK2* rs33949390

**Overdominant model**: The forest plot and result of recessive model of rs33949390 is shown below. Since there was heterogeneity observed (*I*^2^ 58%), results of random effect model were adopted. After meta-analysis to 3342 PD patients and 2865 controls, OR was 0.56 (0.37 – 0.85), *p* value: 0.007.

Appendix Figure 3.168 Overdominant model of *LRRK2* rs33949390

1. ***LRRK2*, rs7304279, T>C**

| Articles | PD subjects | | | Controls | | | Methods | Location of Population |
| --- | --- | --- | --- | --- | --- | --- | --- | --- |
|  | CC | CT | TT | CC | CT | TT |  |  |
| Xue-Li Chang et al., 2011^a,21^ | 548 | 71 | 2 | 467 | 39 | 0 | MassARRAY | Chengdu, Sichuan Province |
| Wen-Juan Yu et al., 2015^a,59^ | 458 | 69 | 2 | 401 | 33 | 0 | MassARRAY | Chengdu, Sichuan Province |
| PD: Parkinson’s disease  a: diagnostic criteria: the United Kingdom brain bank criteria ^3^ | | | | | | | | |

**Allele model**: The forest plot and result of allele model of rs7304279 is shown below. We regarded allele C as risk allele. Since there was no heterogeneity observed, results of fixed effect model were adopted. After meta-analysis to 1150 PD patients and 940 controls, OR was 0.58 (0.43 – 0.77) compared to allele T. *p* value: 0.0002.

Appendix Figure 3.169 Allele model of *LRRK2* rs7304279

**Dominant model**: The forest plot and result of dominant model of rs7304279 is shown below. Since there was no heterogeneity observed, results of fixed effect model were adopted. After meta-analysis to 1150 PD patients and 940 controls, OR was 0.24 (0.03 – 2.09), *p* value: 0.198.

Appendix Figure 3.170 Dominant model of *LRRK2* rs7304279

**Recessive model**: The forest plot and result of recessive model of rs7304279 is shown below. Since there was no heterogeneity observed, results of fixed effect model were adopted. After meta-analysis to 1150 PD patients and 940 controls, OR was 0.58 (0.43 – 0.78), *p* value: 0.0003.

Appendix Figure 3.171 Recessive model of *LRRK2* rs7304279

**Overdominant model**: The forest plot and result of recessive model of rs7304279 is shown below. Since there was no heterogeneity observed, results of fixed effect model were adopted. After meta-analysis to 1150 PD patients and 940 controls, OR was 0.60 (0.44 – 0.81), *p* value: 0.0008.

Appendix Figure 3.172 Overdominant model of *LRRK2* rs7304279

1. ***LRRK2,* rs2046932, G>A**

| Articles | PD subjects | | | Controls | | | Methods | Location of Population |
| --- | --- | --- | --- | --- | --- | --- | --- | --- |
|  | AA | AG | GG | AA | AG | GG |  |  |
| Xue-Li Chang et al., 2011^a,21^ | 527 | 87 | 3 | 464 | 40 | 0 | MassARRAY | Chengdu, Sichuan Province |
| Wen-Juan Yu et al., 2015^a,59^ | 437 | 84 | 3 | 397 | 35 | 0 | MassARRAY | Chengdu, Sichuan Province |
| PD: Parkinson’s disease  a: diagnostic criteria: the United Kingdom brain bank criteria ^3^ | | | | | | | | |

**Allele model**: The forest plot and result of allele model of rs2046932 is shown below. We regarded allele A as risk allele. Since there was no heterogeneity observed, results of fixed effect model were adopted. After meta-analysis to 1141 PD patients and 936 controls, OR was 0.48 (0.36 – 0.63) compared to allele G. *p* value: <0.0001.

Appendix Figure 3.173 Allele model of *LRRK2* rs2046932

**Dominant model**: The forest plot and result of dominant model of rs2046932 is shown below. Since there was no heterogeneity observed, results of fixed effect model were adopted. After meta-analysis to 1141 PD patients and 936 controls, OR was 0.17 (0.02 – 1.41), *p* value: 0.101.

Appendix Figure 3.174 Dominant model of *LRRK2* rs2046932

**Recessive model**: The forest plot and result of recessive model of rs2046932 is shown below. Since there was no heterogeneity observed, results of fixed effect model were adopted. After meta-analysis to 1141 PD patients and 936 controls, OR was 0.47 (0.36 – 0.63), *p* value: <0.0001.

Appendix Figure 3.175 Recessive model of *LRRK2* rs2046932

**Overdominant model**: The forest plot and result of recessive model of rs2046932 is shown below. Since there was no heterogeneity observed, results of fixed effect model were adopted. After meta-analysis to 1141 PD patients and 936 controls, OR was 0.49 (0.37 – 0.66), *p* value: <0.0001.

Appendix Figure 3.176 Overdominant model of *LRRK2* rs2046932

1. ***MAPT,* rs242562, G>A**

| Articles | PD subjects | | | Controls | | | Methods | Location of Population |
| --- | --- | --- | --- | --- | --- | --- | --- | --- |
|  | AA | AG | GG | AA | AG | GG |  |  |
| Lan Yu et al., 2014^a,65^ | 57 | 116 | 36 | 59 | 107 | 64 | PCR-RFLP | Wuhan, Hubei Province |
| Ji-Feng Guo et al., 2015^a,22^ | 307 | 520 | 192 | 305 | 553 | 172 | PCR | Changsha, Hunan Province |
| PCR: Polymerase chain reaction; PD: Parkinson’s disease; RFLP: restricted fragment length polymorphism  a: diagnostic criteria: the United Kingdom brain bank criteria ^3^ | | | | | | | | |

**Allele model**: The forest plot and result of allele model of rs242562 is shown below. We regarded allele A as risk allele. Since there was heterogeneity observed, results of random effect model were adopted. After meta-analysis to 1228 PD patients and 1260 controls, OR was 1.08 (0.83 – 1.42) compared to allele G. *p* value: 0.558.

Appendix Figure 3.177 Allele model of *MAPT* rs242562

**Dominant model**: The forest plot and result of dominant model of rs242562 is shown below. Since there was heterogeneity observed, results of random effect model were adopted. After meta-analysis to 1228 PD patients and 1260 controls, OR was 1.23 (0.58 – 2.60), *p* value: 0.586.

Appendix Figure 3.178 Dominant model of *MAPT* rs242562

**Recessive model**: The forest plot and result of recessive model of rs242562 is shown below. Since there was no heterogeneity observed, results of fixed effect model were adopted. After meta-analysis to 1228 PD patients and 1260 controls, OR was 1.03 (0.87 – 1.23), *p* value: 0.697.

Appendix Figure 3.179 Recessive model of *MAPT* rs242562

**Overdominant model**: The forest plot and result of recessive model of rs242562 is shown below. Since there was heterogeneity observed, results of random effect model were adopted. After meta-analysis to 1228 PD patients and 1260 controls, OR was 0.91 (0.58 – 1.43), *p* value: 0.679.

Appendix Figure 3.180 Overdominant model of *MAPT* rs242562

1. ***MIR4697*, rs329648, T>C**

| Articles | PD subjects | | | Controls | | | Methods | Location of Population |
| --- | --- | --- | --- | --- | --- | --- | --- | --- |
|  | CC | CT | TT | CC | CT | TT |  |  |
| Xinglong Yang et al., 2017^a,^ ^63^ | 243 | 262 | 84 | 280 | 302 | 52 | allele-specific ligation PCR | Chengdu, Sichuan Province |
| MingZou et al., 2018^a, 64^ | 264 | 247 | 68 | 296 | 268 | 78 | SNaPShot | Wenzhou, Zhejiang Province |
| PD: Parkinson’s disease  a: diagnostic criteria: the United Kingdom brain bank criteria ^3^ | | | | | | | | |

**Allele model**: The forest plot and result of allele model of rs329648 is shown below. We regarded allele C as risk allele. Since there was heterogeneity observed, results of random effect model were adopted. After meta-analysis to 1168 PD patients and 1276 controls, OR was 0.75 (0.39 – 1.43) compared to allele T. *p* value: 0.304.

Appendix Figure 3.181 Allele model of *MIR4697* rs329648

**Dominant model**: The forest plot and result of dominant model of rs329648 is shown below. Since there was heterogeneity observed, results of random effect model were adopted. After meta-analysis to 1168 PD patients and 1276 controls, OR was 0.75 (0.39 – 1.43), *p* value: 0.382.

Appendix Figure 3.182 Dominant model of *MIR4697* rs329648

**Recessive model**: The forest plot and result of recessive model of rs329648 is shown below. Since there was no heterogeneity observed, results of fixed effect model were adopted. After meta-analysis to 1168 PD patients and 1276 controls, OR was 0.93 (0.80 – 1.09), *p* value: 0.395.

Appendix Figure 3.183 Recessive model of *MIR4697* rs329648

**Overdominant model**: The forest plot and result of recessive model of rs329648 is shown below. Since there was no heterogeneity observed, results of fixed effect model were adopted. After meta-analysis to 1168 PD patients and 1276 controls, OR was 1.05 (0.89 – 1.23), *p* value: 0.577.

Appendix Figure 3.184 Overdominant model of *MIR4697* rs329648

1. ***NMD3*, rs34016896, C>T**

| Articles | PD subjects | | | Controls | | | Methods | Location of Population |
| --- | --- | --- | --- | --- | --- | --- | --- | --- |
|  | CC | CT | TT | CC | CT | TT |  |  |
| Zhen-hua Liu et al., 2015^a,67^ | 68 | 238 | 154 | 64 | 233 | 176 | MassARRAY | Changsha, Hunan Province |
| Yongping Chen et al., 2018^a,70^ | 207 | 582 | 447 | 153 | 401 | 282 | MassARRAY | Chengdu, Sichuan Province |
| PD: Parkinson’s disease  a: diagnostic criteria: the United Kingdom brain bank criteria ^3^ | | | | | | | | |

**Allele model**: The forest plot and result of allele model of rs34016896 is shown below. We regarded allele T as risk allele. Since there was heterogeneity observed, results of random effect model were adopted. After meta-analysis to 1696 PD patients and 1309 controls, OR was 1.00 (0.84 – 1.20) compared to allele C. *p* value: 0.983.

Appendix Figure 3.185 Allele model of *NMD3* rs34016896

**Dominant model**: The forest plot and result of dominant model of rs34016896 is shown below. Since there was no heterogeneity observed, results of fixed effect model were adopted. After meta-analysis to 1696 PD patients and 1309 controls, OR was 1.05 (0.86 – 1.28), *p* value: 0.630.

Appendix Figure 3.186 Dominant model of *NMD3* rs34016896

**Recessive model**: The forest plot and result of recessive model of rs34016896 is shown below. Since there was heterogeneity observed, results of random effect model were adopted. After meta-analysis to 1696 PD patients and 1309 controls, OR was 0.99 (0.76 – 1.29), *p* value: 0.942.

Appendix Figure 3.187 Recessive model of *NMD3* rs34016896

**Overdominant model**: The forest plot and result of recessive model of rs34016896 is shown below. Since there was no heterogeneity observed, results of fixed effect model were adopted. After meta-analysis to 1696 PD patients and 1309 controls, OR was 0.99 (0.86 – 1.15), *p* value: 0.918.

Appendix Figure 3.188 Overdominant model of *NMD3* rs34016896

1. ***NUCKS1*, rs823128, G>A**

| Articles | PD subjects | | | Controls | | | Methods | Location of Population |
| --- | --- | --- | --- | --- | --- | --- | --- | --- |
|  | AA | AG | GG | AA | AG | GG |  |  |
| Yanyan Zhao et al., 2011^a,98^ | 168 | 52 | 6 | 276 | 76 | 10 | TaqMan | Jiangsu Province |
| Li-Li Zhou et al., 2014^a,55^ | 256 | 61 | 6 | 270 | 69 | 6 | PCR | Wenzhou, Zhejiang Province |
| Xue-Li Chang et al., 2011^a,21^ | 489 | 109 | 8 | 373 | 114 | 9 | MassARRAY | Chengdu, Sichuan Province |
| PCR: polymerase chain reaction; PD: Parkinson’s disease  a: diagnostic criteria: the United Kingdom brain bank criteria ^3^ | | | | | | | | |

**Allele model**: The forest plot and result of allele model of rs823128 is shown below. We regarded allele A as risk allele. Since there was no heterogeneity observed, results of fixed effect model were adopted. After meta-analysis to 1155 PD patients and 1203 controls, OR was 1.13 (0.95 – 1.35) compared to allele G. *p* value: 0.170.

Appendix Figure 3.189 Allele model of *NUCKS1* rs823128

**Dominant model**: The forest plot and result of dominant model of rs823128 is shown below. Since there was no heterogeneity observed, results of fixed effect model were adopted. After meta-analysis to 1155 PD patients and 1203 controls, OR was 1.13 (0.62 – 2.05), *p* value: 0.693.

Appendix Figure 3.190 Dominant model of *NUCKS1* rs823128

**Recessive model**: The forest plot and result of recessive model of rs823128 is shown below. Since there was no heterogeneity observed, results of fixed effect model were adopted. After meta-analysis to 1155 PD patients and 1203 controls, OR was 1.15 (0.94 – 1.40), *p* value: 0.164.

Appendix Figure 3.191 Recessive model of *NUCKS1* rs823128

**Overdominant model**: The forest plot and result of recessive model of rs823128 is shown below. Since there was no heterogeneity observed, results of fixed effect model were adopted. After meta-analysis to 1155 PD patients and 1203 controls, OR was 1.14 (0.93 – 1.40), *p* value: 0.192.

Appendix Figure 3.192 Overdominant model of *NUCKS1* rs823128

1. ***NUCKS1*, rs823114, G>A**

| Articles | PD subjects | | | Controls | | | Methods | Location of Population |
| --- | --- | --- | --- | --- | --- | --- | --- | --- |
|  | AA | AG | GG | AA | AG | GG |  |  |
| Wenqing Zhu et al., 2018^a,99^ | 133 | 141 | 48 | 116 | 181 | 66 | PCR-RFLP | Shenyang, Liaoning Province |
| Zhanyun Lv et al., 2017^a,100^ | 52 | 70 | 36 | 54 | 116 | 40 | TaqMan | Jining, Shandong Province |
| PCR: Polymerase chain reaction; PD: Parkinson’s disease; RFLP: restricted fragment length polymorphism  a: diagnostic criteria: the United Kingdom brain bank criteria ^3^ | | | | | | | | |

**Allele model**: The forest plot and result of allele model of rs823114 is shown below. We regarded allele A as risk allele. Since there was no heterogeneity observed, results of fixed effect model were adopted. After meta-analysis to 480 PD patients and 573 controls, OR was 1.22 (1.02 – 1.45) compared to allele G. *p* value: 0.029.

Appendix Figure 3.193 Allele model of *NUCKS1* rs823114

**Dominant model**: The forest plot and result of dominant model of rs823114 is shown below. Since there was no heterogeneity observed, results of fixed effect model were adopted. After meta-analysis to 480 PD patients and 573 controls, OR was 1.06 (0.77 – 1.45), *p* value: 0.721.

Appendix Figure 3.194 Dominant model of *NUCKS1* rs823114

**Recessive model**: The forest plot and result of recessive model of rs823114 is shown below. Since there was no heterogeneity observed, results of fixed effect model were adopted. After meta-analysis to 480 PD patients and 573 controls, OR was 1.47 (1.14 – 1.90), *p* value: 0.003.

Appendix Figure 3.195 Recessive model of *NUCKS1* rs823114

**Overdominant model**: The forest plot and result of recessive model of rs823114 is shown below. Since there was no heterogeneity observed, results of fixed effect model were adopted. After meta-analysis to 480 PD patients and 573 controls, OR was 1.37 (1.07 – 1.74), *p* value: 0.012.

Appendix Figure 3.196 Overdominant model of *NUCKS1* rs823114

1. ***PARK16*, rs823156, G>A**

| Articles | PD subjects | | | Controls | | | Methods | Location of Population |
| --- | --- | --- | --- | --- | --- | --- | --- | --- |
|  | AA | AG | GG | AA | AG | GG |  |  |
| Xue-Li Chang et al., 2011^a,21^ | 435 | 174 | 13 | 315 | 174 | 17 | MassARRAY | Chengdu, Sichuan Province |
| Ji-Feng Guo et al., 2015^a,22^ | 714 | 273 | 32 | 688 | 300 | 42 | PCR | Changsha, Hunan Province |
| PCR: Polymerase chain reaction; PD: Parkinson’s disease  a: diagnostic criteria: the United Kingdom brain bank criteria ^3^ | | | | | | | | |

**Allele model**: The forest plot and result of allele model of rs823156 is shown below. We regarded allele A as risk allele. Since there was no heterogeneity observed, results of fixed effect model were adopted. After meta-analysis to 1641 PD patients and 1536 controls, OR was 1.22 (1.07 – 1.39) compared to allele G. *p* value: 0.002.

Appendix Figure 3.197 Allele model of *PARK16* rs823156

**Dominant model**: The forest plot and result of dominant model of rs823156 is shown below. Since there was no heterogeneity observed, results of fixed effect model were adopted. After meta-analysis to 1641 PD patients and 1536 controls, OR was 1.40 (0.94 – 2.07), *p* value: 0.097.

Appendix Figure 3.198 Dominant model of *PARK16* rs823156

**Recessive model**: The forest plot and result of recessive model of rs823156 is shown below. Since there was no heterogeneity observed, results of fixed effect model were adopted. After meta-analysis to 1641 PD patients and 1536 controls, OR was 1.25 (1.07 – 1.45), *p* value: 0.004.

Appendix Figure 3.199 Recessive model of *PARK16* rs823156

**Overdominant model**: The forest plot and result of recessive model of rs823156 is shown below. Since there was no heterogeneity observed, results of fixed effect model were adopted. After meta-analysis to 1641 PD patients and 1536 controls, OR was 1.20 (1.03 – 1.40), *p* value: 0.019.

Appendix Figure 3.200 Overdominant model of *PARK16* rs823156

1. ***PARK16*, rs823128, G>A**

| Articles | PD subjects | | | Controls | | | Methods | Location of Population |
| --- | --- | --- | --- | --- | --- | --- | --- | --- |
|  | AA | AG | GG | AA | AG | GG |  |  |
| Xue-Li Chang et al., 2011^a,21^ | 489 | 109 | 8 | 373 | 114 | 9 | MassARRAY | Chengdu, Sichuan Province |
| Yanyan Zhao et al., 2011^a,98^ | 168 | 52 | 6 | 276 | 76 | 10 | TaqMan | Nanjing, Jiangsu Province |
| PD: Parkinson’s disease  a: diagnostic criteria: the United Kingdom brain bank criteria ^3^ | | | | | | | | |

**Allele model**: The forest plot and result of allele model of rs823128 is shown below. We regarded allele A as risk allele. Since there was heterogeneity observed, results of random effect model were adopted. After meta-analysis to 832 PD patients and 858 controls, OR was 1.13 (0.79 – 1.62) compared to allele G. *p* value: 0.496.

Appendix Figure 3.201 Allele model of *PARK16* rs823128

**Dominant model**: The forest plot and result of dominant model of rs823128 is shown below. Since there was no heterogeneity observed, results of fixed effect model were adopted. After meta-analysis to 832 PD patients and 858 controls, OR was 1.21 (0.60 – 2.44), *p* value: 0.595.

Appendix Figure 3.202 Dominant model of *PARK16* rs823128

**Recessive model**: The forest plot and result of recessive model of rs823128 is shown below. Since there was heterogeneity observed, results of random effect model were adopted. After meta-analysis to 832 PD patients and 858 controls, OR was 1.14 (0.75 – 1.72), *p* value: 0.540.

Appendix Figure 3.203 Recessive model of *PARK16* rs823128

**Overdominant model**: The forest plot and result of recessive model of rs823128 is shown below. Since there was heterogeneity observed, results of random effect model were adopted. After meta-analysis to 832 PD patients and 858 controls, OR was 1.12 (0.74 – 1.70), *p* value: 0.578.

Appendix Figure 3.204 Overdominant model of *PARK16* rs823128

1. ***PITX3*, rs2281983, C>T**

| Articles | PD subjects | | | Controls | | | Methods | Location of Population |
| --- | --- | --- | --- | --- | --- | --- | --- | --- |
|  | CC | CT | TT | CC | CT | TT |  |  |
| Jia Liu et al., 2011^a,101^ | 27 | 189 | 296 | 30 | 187 | 289 | PCR-RFLP | Changsha, Hunan Province |
| Yanning Cai et al., 2011^a,102^ | 29 | 287 | 483 | 24 | 180 | 290 | PCR-LDR | Beijing |
| Yaxing Gui et al., 2012^b, 103^ | 111 | 177 | 68 | 118 | 133 | 49 | PCR | Hangzhou, Zhejiang Province |
| LDR: ligase detection reaction; PCR: Polymerase chain reaction; PD: Parkinson’s disease; RFLP: restricted fragment length polymorphism  a: diagnostic criteria: the United Kingdom brain bank criteria ^3^  b: diagnostic criteria: brought up by Deuschl G et al.^104^ | | | | | | | | |

**Allele model**: The forest plot and result of allele model of rs2281983 is shown below. We regarded allele T as risk allele. Since there was no heterogeneity observed, results of fixed effect model were adopted. After meta-analysis to 1667 PD patients and 1300 controls, OR was 1.12 (0.99 – 1.25) compared to allele C. *p* value: 0.069.

Appendix Figure 3.205 Allele model of *PITX3* rs2281983

**Dominant model**: The forest plot and result of dominant model of rs2281983 is shown below. Since there was no heterogeneity observed, results of fixed effect model were adopted. After meta-analysis to 1667 PD patients and 1300 controls, OR was 1.35 (1.05 – 1.72), *p* value: 0.018.

Appendix Figure 3.206 Dominant model of *PITX3* rs2281983

**Recessive model**: The forest plot and result of recessive model of rs2281983 is shown below. Since there was no heterogeneity observed, results of fixed effect model were adopted. After meta-analysis to 1667 PD patients and 1300 controls, OR was 1.08 (0.92 – 1.26), *p* value: 0.358.

Appendix Figure 3.207 Recessive model of *PITX3* rs2281983

**Overdominant model**: The forest plot and result of recessive model of rs2281983 is shown below. Since there was no heterogeneity observed, results of fixed effect model were adopted. After meta-analysis to 1667 PD patients and 1300 controls, OR was 1.35 (1.05 – 1.72), *p* value: 0.018.

Appendix Figure 3.208 Overdominant model of *PITX3* rs2281983

1. ***PITX3*, rs3758549, C>T**

| Articles | PD subjects | | | Controls | | | Methods | Location of Population |
| --- | --- | --- | --- | --- | --- | --- | --- | --- |
|  | CC | CT | TT | CC | CT | TT |  |  |
| L.-H. Yu et al., 2011^a,105^ | 171 | 119 | 26 | 200 | 89 | 16 | PCR-RFLP | Guangzhou, Guangdong Province |
| Jia Liu et al., 2011^a,101^ | 330 | 167 | 15 | 366 | 125 | 15 | PCR-RFLP | Changsha, Hunan Province |
| Yanning Cai et al., 2011^a,102^ | 595 | 183 | 21 | 388 | 114 | 7 | PCR-LDR | Beijing |
| Yaxing Gui et al., 2012^b, 103^ | 59 | 154 | 143 | 61 | 122 | 117 | PCR | Hangzhou, Zhejiang Province |
| LDR: ligase detection reaction; PCR: Polymerase chain reaction; PD: Parkinson’s disease; RFLP: restricted fragment length polymorphism  a: diagnostic criteria: the United Kingdom brain bank criteria ^3^  b: diagnostic criteria: brought up by Deuschl G et al.^104^ | | | | | | | | |

**Allele model**: The forest plot and result of allele model of rs3758549 is shown below. We regarded allele T as risk allele. Since there was no heterogeneity observed, results of fixed effect model were adopted. After meta-analysis to 1983 PD patients and 1620 controls, OR was 1.24 (1.10 – 1.40) compared to allele C. *p* value: 0.0004.

Appendix Figure 3.209 Allele model of *PITX3* rs3758549

**Dominant model**: The forest plot and result of dominant model of rs3758549 is shown below. Since there was no heterogeneity observed, results of fixed effect model were adopted. After meta-analysis to 1983 PD patients and 1620 controls, OR was 1.33 (1.14 – 1.54), *p* value: 0.0002.

Appendix Figure 3.210 Dominant model of *PITX3* rs3758549

**Recessive model**: The forest plot and result of recessive model of rs3758549 is shown below. Since there was no heterogeneity observed, results of fixed effect model were adopted. After meta-analysis to 1983 PD patients and 1620 controls, OR was 1.18 (0.92 – 1.52), *p* value: 0.191.

Appendix Figure 3.211 Recessive model of *PITX3* rs3758549

**Overdominant model**: The forest plot and result of recessive model of rs3758549 is shown below. Since there was no heterogeneity observed, results of fixed effect model were adopted. After meta-analysis to 1983 PD patients and 1620 controls, OR was 0.81 (0.70 – 0.93), *p* value: 0.004.

Appendix Figure 3.212 Overdominant model of *PITX3* rs3758549

1. ***PITX3*, rs4919621, A>T**

| Articles | PD subjects | | | Controls | | | Methods | Location of Population |
| --- | --- | --- | --- | --- | --- | --- | --- | --- |
|  | AA | AT | TT | AA | AT | TT |  |  |
| Jia Liu et al., 2011^a,101^ | 27 | 188 | 297 | 34 | 182 | 290 | PCR-RFLP | Changsha, Hunan Province |
| Yanning Cai et al., 2011^a,102^ | 27 | 283 | 489 | 24 | 175 | 295 | PCR-LDR | Beijing |
| Yaxing Gui et al., 2012^b, 103^ | 111 | 177 | 68 | 118 | 133 | 49 | PCR | Hangzhou, Zhejiang Province |
| LDR: ligase detection reaction; PCR: Polymerase chain reaction; PD: Parkinson’s disease; RFLP: restricted fragment length polymorphism  a: diagnostic criteria: the United Kingdom brain bank criteria ^3^  b: diagnostic criteria: brought up by Deuschl G et al.^104^ | | | | | | | | |

**Allele model**: The forest plot and result of allele model of rs4919621 is shown below. We regarded allele T as risk allele. Since there was no heterogeneity observed, results of fixed effect model were adopted. After meta-analysis to 1667 PD patients and 1300 controls, OR was 1.12 (1.00 – 1.26) compared to allele A. *p* value: 0.052.

Appendix Figure 3.213 Allele model of *PITX3* rs4919621

**Dominant model**: The forest plot and result of dominant model of rs4919621 is shown below. Since there was no heterogeneity observed, results of fixed effect model were adopted. After meta-analysis to 1667 PD patients and 1300 controls, OR was 1.40 (1.10 – 1.80), *p* value: 0.007.

Appendix Figure 3.214 Dominant model of *PITX3* rs4919621

**Recessive model**: The forest plot and result of recessive model of rs4919621 is shown below. Since there was no heterogeneity observed, results of fixed effect model were adopted. After meta-analysis to 1667 PD patients and 1300 controls, OR was 1.07 (0.92 – 1.25), *p* value: 0.391.

Appendix Figure 3.215 Recessive model of *PITX3* rs4919621

**Overdominant model**: The forest plot and result of recessive model of rs4919621 is shown below. Since there was no heterogeneity observed, results of fixed effect model were adopted. After meta-analysis to 1667 PD patients and 1300 controls, OR was 0.94 (0.81 – 1.09), *p* value: 0.412.

Appendix Figure 3.216 Overdominant model of *PITX3* rs4919621

1. ***SCARB2*, rs6812193, C>T**

| Articles | PD subjects | | | Controls | | | Methods | Location of Population |
| --- | --- | --- | --- | --- | --- | --- | --- | --- |
|  | CC | CT | TT | CC | CT | TT |  |  |
| Kai Li et al., 2012^a,106^ | 444 | 57 | 0 | 433 | 69 | 0 | PCR-RFLP | Changsha, Hunan Province |
| Shuai Chen et al., 2012^a,107^ | 372 | 73 | 4 | 377 | 69 | 6 | PCR-RFLP | Shanghai |
| PCR: Polymerase chain reaction; PD: Parkinson’s disease; RFLP: restricted fragment length polymorphism  a: diagnostic criteria: the United Kingdom brain bank criteria ^3^ | | | | | | | | |

**Allele model**: The forest plot and result of allele model of rs6812193 is shown below. We regarded allele T as risk allele. Since there was no heterogeneity observed, results of fixed effect model were adopted. After meta-analysis to 950 PD patients and 954 controls, OR was 0.92 (0.72 – 1.17) compared to allele C. *p* value: 0.487.

Appendix Figure 3.217 Allele model of *SCARB2* rs6812193

**Dominant model**: The forest plot and result of dominant model of rs6812193 is shown below. Since there was no heterogeneity observed, results of fixed effect model were adopted. After meta-analysis to 950 PD patients and 954 controls, OR was 0.92 (0.72 – 1.19), *p* value: 0.543.

Appendix Figure 3.218 Dominant model of *SCARB2* rs6812193

**Recessive model**: It was not applied to perform meta-analysis because of lack of mutant homozygote in Kai Li’s study.

**Overdominant model**: The forest plot and result of recessive model of rs6812193 is shown below. Since there was no heterogeneity observed, results of fixed effect model were adopted. After meta-analysis to 950 PD patients and 954 controls, OR was 1.07 (0.82 – 1.38), *p* value: 0.626.

Appendix Figure 3.219 Overdominant model of *SCARB2* rs6812193

1. ***SEMA5A*, rs7702187, T>A**

| Articles | PD subjects | | | Controls | | | Methods | Location of Population |
| --- | --- | --- | --- | --- | --- | --- | --- | --- |
|  | AA | AT | TT | AA | AT | TT |  |  |
| Haixia Ding et al., 2008^a,108^ | 28 | 92 | 220 | 14 | 63 | 145 | PCR-RFLP | Jiangsu Province |
| Hairong Dong et al., 2009^a,109^ | 25 | 63 | 156 | 10 | 49 | 115 | PCR-RFLP | Nanjing, Jiangsu Province |
| PCR: Polymerase chain reaction; PD: Parkinson’s disease; RFLP: restricted fragment length polymorphism  a: diagnostic criteria: by Calne et al.^78^ | | | | | | | | |

**Allele model**: The forest plot and result of allele model of rs7702187 is shown below. We regarded allele A as risk allele. Since there was no heterogeneity observed, results of fixed effect model were adopted. After meta-analysis to 584 PD patients and 396 controls, OR was 1.14 (0.91 – 1.42) compared to allele T. *p* value: 0.255.

Appendix Figure 3.220 Allele model of *SEMA5A* rs7702187

**Dominant model**: The forest plot and result of dominant model of rs7702187 is shown below. Since there was no heterogeneity observed, results of fixed effect model were adopted. After meta-analysis to 584 PD patients and 396 controls, OR was 1.06 (0.81 – 1.38), *p* value: 0.681.

Appendix Figure 3.221 Dominant model of *SEMA5A* rs7702187

**Recessive model**: The forest plot and result of recessive model of rs7702187 is shown below. Since there was no heterogeneity observed, results of fixed effect model were adopted. After meta-analysis to 584 PD patients and 396 controls, OR was 1.55 (0.94 – 2.56), *p* value: 0.086.

Appendix Figure 3.222 Recessive model of *SEMA5A* rs7702187

**Overdominant model**: The forest plot and result of recessive model of rs7702187 is shown below. Since there was no heterogeneity observed, results of fixed effect model were adopted. After meta-analysis to 584 PD patients and 396 controls, OR was 1.09 (0.82 – 1.45), *p* value: 0.544.

Appendix Figure 3.223 Overdominant model of *SEMA5A* rs7702187

1. ***SEMA5A*, rs3798097, C>T**

| Articles | PD subjects | | | Controls | | | Methods | Location of Population |
| --- | --- | --- | --- | --- | --- | --- | --- | --- |
|  | CC | CT | TT | CC | CT | TT |  |  |
| Haixia Ding et al., 2008^a,108^ | 276 | 61 | 3 | 186 | 34 | 2 | PCR-RFLP | Jiangsu Province |
| Hairong Dong et al., 2009^a,109^ | 201 | 41 | 2 | 145 | 28 | 2 | PCR-RFLP | Nanjing, Jiangsu Province |
| PCR: Polymerase chain reaction; PD: Parkinson’s disease; RFLP: restricted fragment length polymorphism  a: diagnostic criteria: by Calne et al.^78^ | | | | | | | | |

**Allele model**: The forest plot and result of allele model of rs3798097 is shown below. We regarded allele T as risk allele. Since there was no heterogeneity observed, results of fixed effect model were adopted. After meta-analysis to 584 PD patients and 397 controls, OR was 1.10 (0.80 – 1.50) compared to allele C. *p* value: 0.564.

Appendix Figure 3.224 Allele model of *SEMA5A* rs3798097

**Dominant model**: The forest plot and result of dominant model of rs3798097 is shown below. Since there was no heterogeneity observed, results of fixed effect model were adopted. After meta-analysis to 584 PD patients and 397 controls, OR was 1.12 (0.80 – 1.58), *p* value: 0.496.

Appendix Figure 3.225 Dominant model of *SEMA5A* rs3798097

**Recessive model**: The forest plot and result of recessive model of rs3798097 is shown below. Since there was no heterogeneity observed, results of fixed effect model were adopted. After meta-analysis to 584 PD patients and 397 controls, OR was 0.85 (0.23 – 3.19), *p* value: 0.809.

Appendix Figure 3.226 Recessive model of *SEMA5A* rs3798097

**Overdominant model**: The forest plot and result of recessive model of rs3798097 is shown below. Since there was no heterogeneity observed, results of fixed effect model were adopted. After meta-analysis to 584 PD patients and 397 controls, OR was 0.88 (0.62 – 1.24), *p* value: 0.449.

Appendix Figure 3.227 Overdominant model of *SEMA5A* rs3798097

1. ***SIPA1L2*, rs10797576, C>T**

| Articles | PD subjects | | | Controls | | | Methods | Location of Population |
| --- | --- | --- | --- | --- | --- | --- | --- | --- |
|  | CC | CT | TT | CC | CT | TT |  |  |
| Xinglong Yang et al., 2017^a, 63^ | 414 | 162 | 13 | 475 | 141 | 18 | allele-specific ligation PCR | Chengdu, Sichuan Province |
| Ming Zou et al., 2018^a,^ ^64^ | 394 | 168 | 17 | 489 | 146 | 7 | SNaPShot | Wenzhou, Zhejiang Province |
| PCR: polymerase chain reaction; PD: Parkinson’s disease  a: diagnostic criteria: the United Kingdom brain bank criteria ^3^ | | | | | | | | |

**Allele model**: The forest plot and result of allele model of rs10797576 is shown below. We regarded allele T as risk allele. Since there was heterogeneity observed, results of random effect model were adopted. After meta-analysis to 1168 PD patients and 1276 controls, OR was 1.32 (1.04 – 1.66) compared to allele C. *p* value: 0.020.

Appendix Figure 3.228 Allele model of *SIPA1L2* rs10797576

**Dominant model**: The forest plot and result of dominant model of rs10797576 is shown below. Since there was no heterogeneity observed, results of fixed effect model were adopted. After meta-analysis to 1168 PD patients and 1276 controls, OR was 1.38 (1.15 – 1.64), *p* value: 0.0004.

Appendix Figure 3.229 Dominant model of *SIPA1L2* rs10797576

**Recessive model**: The forest plot and result of recessive model of rs10797576 is shown below. Since there was heterogeneity observed, results of random effect model were adopted. After meta-analysis to 1168 PD patients and 1276 controls, OR was 1.42 (0.41 – 4.91), *p* value: 0.583.

Appendix Figure 3.230 Recessive model of *SIPA1L2* rs10797576

**Overdominant model**: The forest plot and result of recessive model of rs10797576 is shown below. Since there was no heterogeneity observed, results of fixed effect model were adopted. After meta-analysis to 1168 PD patients and 1276 controls, OR was 0.74 (0.61 – 0.88), *p* value: 0.001.

Appendix Figure 3.231 Overdominant model of *SIPA1L2* rs10797576

1. ***SLC1A2*, rs3794087, C>A**

| Articles | PD subjects | | | Controls | | | Methods | Location of Population |
| --- | --- | --- | --- | --- | --- | --- | --- | --- |
|  | AA | AC | CC | AA | AC | CC |  |  |
| Yaqian Xu et al., 2016^a,110^ | 32 | 210 | 379 | 23 | 174 | 240 | MassARRAY | Chengdu, Sichuan Province |
| Yuan Cheng at al., 2018^a,111^ | 37 | 195 | 314 | 36 | 185 | 329 | PCR | Zhengzhou, Henan Province |
| PCR: Polymerase chain reaction; PD: Parkinson’s disease  a: diagnostic criteria: the United Kingdom brain bank criteria ^3^ | | | | | | | | |

**Allele model**: The forest plot and result of allele model of rs3794087 is shown below. We regarded allele A as risk allele. Since there was heterogeneity observed, results of random effect model were adopted. After meta-analysis to 1167 PD patients and 987 controls, OR was 0.95 (0.75 – 1.21) compared to allele C. *p* value: 0.680.

Appendix Figure 3.232 Allele model of *SLC1A2* rs3794087

**Dominant model**: The forest plot and result of dominant model of rs3794087 is shown below. Since there was heterogeneity observed, results of random effect model were adopted. After meta-analysis to 1167 PD patients and 987 controls, OR was 0.93 (0.66 – 1.30), *p* value: 0.658.

Appendix Figure 3.233 Dominant model of *SLC1A2* rs3794087

**Recessive model**: The forest plot and result of recessive model of rs3794087 is shown below. Since there was no heterogeneity observed, results of fixed effect model were adopted. After meta-analysis to 1167 PD patients and 987 controls, OR was 1.01 (0.71 – 1.45), *p* value: 0.949

Appendix Figure 3.234 Recessive model of *SLC1A2* rs3794087

**Overdominant model**: The forest plot and result of recessive model of rs3794087 is shown below. Since there was heterogeneity observed, results of random effect model were adopted. After meta-analysis to 1167 PD patients and 987 controls, OR was 1.09 (0.77 – 1.53), *p* value: 0.638.

Appendix Figure 3.235 Overdominant model of *SLC1A2* rs3794087

1. ***SREBF1/RAI1*, rs11868035, G>A**

| Articles | PD subjects | | | Controls | | | Methods | Location of Population |
| --- | --- | --- | --- | --- | --- | --- | --- | --- |
|  | AA | AG | GG | AA | AG | GG |  |  |
| Xiaoqin Yuan et al., 2018^a,112^ | 820 | 297 | 33 | 548 | 242 | 24 | MassARRAY | Chengdu, Sichuan Province |
| Kai Li et al., 2012^a,106^ | 353 | 125 | 23 | 359 | 122 | 21 | PCR-RFLP | Changsha, Hunan Province |
| Fan Lou et al., 2018^a,113^ | 467 | 168 | 14 | 312 | 40 | 3 | KASP assay | Shenyang, Liaoning Province |
| KASP: Kompetitive Allele-Specific PCR; PCR: Polymerase chain reaction; PD: Parkinson’s disease; RFLP: restricted fragment length polymorphism  a: diagnostic criteria: the United Kingdom brain bank criteria ^3^ | | | | | | | | |

**Allele model**: The forest plot and result of allele model of rs11969035 is shown below. We regarded allele A as risk allele. Since there was heterogeneity observed, results of random effect model were adopted. After meta-analysis to 2300 PD patients and 1671 controls, OR was 0.77 (0.44 – 1.33) compared to allele G. *p* value: 0.346.

Appendix Figure 3.236 Allele model of *SREBF1/RAI1* rs11868035

**Dominant model**: The forest plot and result of dominant model of rs11969035 is shown below. Since there was no heterogeneity observed, results of fixed effect model were adopted. After meta-analysis to 2300 PD patients and 1671 controls, OR was 0.87 (0.60 – 1.28), *p* value: 0.487.

Appendix Figure 3.237 Dominant model of *SREBF1/RAI1* rs11868035

**Recessive model**: The forest plot and result of recessive model of rs11969035 is shown below. Since there was heterogeneity observed, results of random effect model were adopted. After meta-analysis to 2300 PD patients and 1671 controls, OR was 0.75 (0.39 – 1.43), *p* value: 0.386.

Appendix Figure 3.238 Recessive model of *SREBF1/RAI1* rs11868035

**Overdominant model**: The forest plot and result of recessive model of rs11969035 is shown below. Since there was heterogeneity observed, results of random effect model were adopted. After meta-analysis to 2300 PD patients and 1671 controls, OR was 0.77 (0.40 – 1.45), *p* value: 0.413.

Appendix Figure 3.239 Overdominant model of *SREBF1/RAI1* rs11868035

1. ***USP40*, rs1048603, C>T**

| Articles | PD subjects | | | Controls | | | Methods | Location of Population |
| --- | --- | --- | --- | --- | --- | --- | --- | --- |
|  | CC | CT | TT | CC | CT | TT |  |  |
| Bi Zhao et al., 2012^a,114^ | 273 | 97 | 8 | 185 | 79 | 10 | MassARRAY | Chengdu, Sichuan Province |
| Yih-Ru Wu et al., 2010^b,115^ | 396 | 110 | 11 | 381 | 124 | 11 | PCR-RFLP | Taiwan |
| PCR: Polymerase chain reaction; PD: Parkinson’s disease; RFLP: restricted fragment length polymorphism  a: diagnostic criteria: the United Kingdom brain bank criteria ^3^  b: diagnostic criteria brought up by Gelb et al.^15^ | | | | | | | | |

**Allele model**: The forest plot and result of allele model of rs1048603 is shown below. We regarded allele T as risk allele. Since there was no heterogeneity observed, results of fixed effect model were adopted. After meta-analysis to 895 PD patients and 790 controls, OR was 0.85 (0.70 – 1.03) compared to allele C. *p* value: 0.094.

Appendix Figure 3.240 Allele model of *USP40* rs1048603

**Dominant model**: The forest plot and result of dominant model of rs1048603 is shown below. Since there was no heterogeneity observed, results of fixed effect model were adopted. After meta-analysis to 895 PD patients and 790 controls, OR was 0.84 (0.67 – 1.04), *p* value: 0.106.

Appendix Figure 3.241 Dominant model of *USP40* rs1048603

**Recessive model**: The forest plot and result of recessive model of rs1048603 is shown below. Since there was no heterogeneity observed, results of fixed effect model were adopted. After meta-analysis to 895 PD patients and 790 controls, OR was 0.78 (0.42 – 1.46), *p* value: 0.435.

Appendix Figure 3.242 Recessive model of *USP40* rs1048603

**Overdominant model**: The forest plot and result of recessive model of rs1048603 is shown below. Since there was no heterogeneity observed, results of fixed effect model were adopted. After meta-analysis to 895 PD patients and 790 controls, OR was 1.17 (0.94 – 1.47), *p* value: 0.165.

Appendix Figure 3.243 Overdominant model of *USP40* rs1048603

1. ***VPS13C*, rs2414739, G>A**

| Articles | PD subjects | | | Controls | | | Methods | Location of Population |
| --- | --- | --- | --- | --- | --- | --- | --- | --- |
|  | AA | AG | GG | AA | AG | GG |  |  |
| Xinglong Yang et al., 2017^a,63^ | 434 | 138 | 17 | 459 | 155 | 20 | allele-specific ligation PCR | Chengdu, Sichuan Province |
| MingZou et al., 2018^a,64^ | 438 | 133 | 8 | 441 | 187 | 14 | SNaPShot | Wenzhou, Zhejiang Province |
| PCR: Polymerase chain reaction; PD: Parkinson’s disease  a: diagnostic criteria: the United Kingdom brain bank criteria ^3^ | | | | | | | | |

**Allele model**: The forest plot and result of allele model of rs2414739 is shown below. We regarded allele A as risk allele. Since there was heterogeneity observed, results of random effect model were adopted. After meta-analysis to 1168 PD patients and 1276 controls, OR was 1.20 (0.94 – 1.53) compared to allele G. *p* value: 0.137.

Appendix Figure 3.244 Allele model of *VPS13C* rs2414739

**Dominant model**: The forest plot and result of dominant model of rs2414739 is shown below. Since there was no heterogeneity observed, results of fixed effect model were adopted. After meta-analysis to 1168 PD patients and 1276 controls, OR was 1.26 (0.74 – 2.12), *p* value: 0.391.

Appendix Figure 3.245 Dominant model of *VPS13C* rs2414739

**Recessive model**: The forest plot and result of recessive model of rs2414739 is shown below. Since there was heterogeneity observed, results of random effect model were adopted. After meta-analysis to 1168 PD patients and 1276 controls, OR was 1.23 (0.93 – 1.62), *p* value: 0.143.

Appendix Figure 3.246 Recessive model of *VPS13C* rs2414739

**Overdominant model**: The forest plot and result of recessive model of rs2414739 is shown below. Since there was no heterogeneity observed (*I*^2^ 49.7%), results of fixed effect model were adopted. After meta-analysis to 1168 PD patients and 1276 controls, OR was 1.21 (1.01 – 1.46), *p* value: 0.041.

Appendix Figure 3.247 Overdominant model of *VPS13C* rs2414739

1. ***PARK16*, rs947211, A>G**

| Articles | PD subjects | | | Controls | | | Methods | Location of Population |
| --- | --- | --- | --- | --- | --- | --- | --- | --- |
|  | AA | AG | GG | AA | AG | GG |  |  |
| Donghui Li et al., 2011^a, 116^ | 27 | 72 | 61 | 44 | 108 | 48 | RT-PCR | Suzhou, Jiangsu Province |
| Yanyan Zhao et al., 2011^a, 98^ | 45 | 104 | 77 | 84 | 192 | 86 | TaqMan | Jiangsu Province |
| Li-Li Zhou et al., 2014^a, 55^ | 59 | 147 | 117 | 71 | 160 | 114 | PCR | Wenzhou, Zhejiang Province |
| H. Xia et al., 2015^a, 117^ | 44 | 148 | 88 | 95 | 172 | 64 | PCR-RFLP | Urumqi, Xinjiang |
| Hanchun Long et al., 2016^a, 118^ | 11 | 28 | 21 | 14 | 32 | 14 | PCR | Xingyi, Guizhou Province |
| Hanchun Long et al., 2016^a,†,^ ^118^ | 12 | 28 | 22 | 15 | 31 | 14 | PCR | Xingyi, Guizhou Province |
| Hanchun Long et al., 2016^a,‡,^ ^118^ | 13 | 30 | 23 | 16 | 30 | 14 | PCR | Xingyi, Guizhou Province |
| PCR: Polymerase chain reaction; PD: Parkinson’s disease; RFLP: restricted fragment length polymorphism; RT-PCR: real time PCR  a: diagnostic criteria: the United Kingdom brain bank criteria ^3^  †: target population: Buyi ethnicity  ‡: target population: Miao ethnicity | | | | | | | | |

**Allele model**: The forest plot and result of allele model of rs947211 is shown below. We regarded allele G as risk allele. Since there was no heterogeneity observed, results of fixed effect model were adopted. After meta-analysis to 1049 PD patients and 1298 controls, OR was 1.36 (1.21 – 1.53) compared to allele A. *p* value: <0.0001.

Appendix Figure 3.248 Allele model of *PARK16* rs947211

**Dominant model**: The forest plot and result of dominant model of rs947211 is shown below. Since there was no heterogeneity observed, results of fixed effect model were adopted. After meta-analysis to 1049 PD patients and 1298 controls, OR was 1.44 (1.17 – 1.76), *p* value: 0.0005.

Appendix Figure 3.249 Dominant model of *PARK16* rs947211

**Recessive model**: The forest plot and result of recessive model of rs947211 is shown below. Since there was no heterogeneity observed, results of fixed effect model were adopted. After meta-analysis to 1049 PD patients and 1298 controls, OR was 1.57 (1.31 – 1.88), *p* value: <0.0001.

Appendix Figure 3.250 Recessive model of *PARK16* rs947211

**Overdominant model**: The forest plot and result of recessive model of rs947211 is shown below. Since there was no heterogeneity observed, results of fixed effect model were adopted. After meta-analysis to 1049 PD patients and 1298 controls, OR was 1.15 (0.97 – 1.35), *p* value: 0.098.

Appendix Figure 3.251 Overdominant model of *PARK16* rs947211

1. ***PPARGC1A*, rs17590046, T>C**

| Articles | PD subjects | | | Controls | | | Methods | Location of Population |
| --- | --- | --- | --- | --- | --- | --- | --- | --- |
|  | CC | CT | TT | CC | CT | TT |  |  |
| Yuan Zhang et al., 2017^a, 42^ | 5 | 91 | 414 | 8 | 77 | 451 | PCR | Changsha, Hunan Province |
| Chang-he Shi et al., 2018^a, 43^ | 2 | 89 | 455 | 2 | 110 | 438 | PCR | Chengdu, Sichuan Province |
| PCR: Polymerase chain reaction; PD: Parkinson’s disease  a: diagnostic criteria: the United Kingdom brain bank criteria ^3^ | | | | | | | | |

**Allele model**: The forest plot and result of allele model of rs17590046 is shown below. We regarded allele C as risk allele. Since there was heterogeneity observed, results of random effect model were adopted. After meta-analysis to 1056 PD patients and 1086 controls, OR was 0.96 (0.68 – 1.37) compared to allele T. *p* value: 0.838.

Appendix Figure 3.252 Allele model of *PPARGC1A* rs17590046

**Dominant model**: The forest plot and result of dominant model of rs17590046 is shown below. Since there was heterogeneity observed, results of random effect model were adopted. After meta-analysis to 1056 PD patients and 1086 controls, OR was 0.98 (0.63 – 1.53), *p* value: 0.923.

Appendix Figure 3.253 Dominant model of *PPARGC1A* rs17590046

**Recessive model**: The forest plot and result of recessive model of rs17590046 is shown below. Since there was no heterogeneity observed, results of fixed effect model were adopted. After meta-analysis to 1056 PD patients and 1086 controls, OR was 0.73 (0.27 – 1.92), *p* value: 0.518.

Appendix Figure 3.254 Recessive model of *PPARGC1A* rs17590046

**Overdominant model**: The forest plot and result of recessive model of rs17590046 is shown below. Since there was heterogeneity observed, results of random effect model were adopted. After meta-analysis to 1056 PD patients and 1086 controls, OR was 1.00 (0.61 – 1.64), *p* value: 0.998.

Appendix Figure 3.255 Overdominant model of *PPARGC1A* rs17590046

1. ***RIT2*, rs12456492, A>G**

| Articles | PD subjects | | | Controls | | | Methods | Location of Population |
| --- | --- | --- | --- | --- | --- | --- | --- | --- |
|  | AA | AG | GG | AA | AG | GG |  |  |
| Zhenhua Liu et al., 2015^a,^ ^119^ | 135 | 225 | 100 | 177 | 212 | 84 | MassArray | Changsha, Hunan Province |
| Kun Nie et al., 2015^a, 120^ | 171 | 267 | 86 | 210 | 259 | 52 | PCR-LDR | Guangzhou, Guangdong Province |
| Jianyong Wang et al., 2014^a,^ ^121^ | 184 | 257 | 96 | 214 | 229 | 74 | PCR-RFLP | Wenzhou, Zhejiang Province |
| Chin-Hsien Lin et al., 2013 ^a,^ ^122^ | 77 | 252 | 171 | 75 | 236 | 189 | TaqMan | Taipei, Taiwan Province |
| LDR: ligase detection reaction; PCR: Polymerase chain reaction; PD: Parkinson’s disease; RFLP: restricted fragment length polymorphism  a: diagnostic criteria: the United Kingdom brain bank criteria ^3^ | | | | | | | | |

**Allele model**: The forest plot and result of allele model of rs12456492 is shown below. We regarded allele G as risk allele. Since there was heterogeneity observed, results of random effect model were adopted. After meta-analysis to 1056 PD patients and 1086 controls, OR was 1.19 (1.00 – 1.41) compared to allele A. *p* value: 0.047.

Appendix Figure 3.256 Allele model of *RIT2* rs12456492

**Dominant model**: The forest plot and result of dominant model of rs12456492 is shown below. Since there was no heterogeneity observed, results of fixed effect model were adopted. After meta-analysis to 1056 PD patients and 1086 controls, OR was 1.32 (1.15 – 1.51), *p* value: <0.0001.

Appendix Figure 3.257 Dominant model of *RIT2* rs12456492

**Recessive model**: The forest plot and result of recessive model of rs12456492 is shown below. Since there was heterogeneity observed, results of random effect model were adopted. After meta-analysis to 1056 PD patients and 1086 controls, OR was 1.24 (0.91 – 1.69), *p* value: 0.169.

Appendix Figure 3.258 Recessive model of *RIT2* rs12456492

**Overdominant model**: The forest plot and result of recessive model of rs12456492 is shown below. Since there was no heterogeneity observed, results of fixed effect model were adopted. After meta-analysis to 1056 PD patients and 1086 controls, OR was 0.89 (0.78 - 1.00), *p* value: 0.057.

Appendix Figure 3.259 Overdominant model of *RIT2* rs12456492

1. ***SLC41A1*, rs11240569, G>A**

| Articles | PD subjects | | | Controls | | | Methods | Location of Population |
| --- | --- | --- | --- | --- | --- | --- | --- | --- |
|  | GG | GA | AA | GG | GA | AA |  |  |
| Ling Wang et al., 2015^a, 123^ | 132 | 499 | 432 | 166 | 591 | 417 | MassARRAY | Chengdu, Sichuan Province |
| Yaping Yan et al., 2011^a, 124^ | 74 | 90 | 41 | 61 | 111 | 38 | PCR | Hangzhou, Zhejiang Province |
| PCR: Polymerase chain reaction; PD: Parkinson’s disease  a: diagnostic criteria: the United Kingdom brain bank criteria ^3^ | | | | | | | | |

**Allele model**: The forest plot and result of allele model of rs11240569 is shown below. We regarded allele A as risk allele. Since there was heterogeneity observed, results of random effect model were adopted. After meta-analysis to 1268 PD patients and 1384 controls, OR was 0.95 (0.75 – 1.20) compared to allele G. *p* value: 0.668.

Appendix Figure 3.260 Allele model of *SLC41A1* rs11240569

**Dominant model**: The forest plot and result of dominant model of rs11240569 is shown below. Since there was no heterogeneity observed, results of fixed effect model were adopted. After meta-analysis to 1268 PD patients and 1384 controls, OR was 0.81 (0.69 – 0.96), *p* value: 0.0119.

Appendix Figure 3.261 Dominant model of *SLC41A1* rs11240569

**Recessive model**: The forest plot and result of recessive model of rs11240569 is shown below. Since there was heterogeneity observed, results of random effect model were adopted. After meta-analysis to 1268 PD patients and 1384 controls, OR was 1.06 (0.67 – 1.67), *p* value: 0.811.

Appendix Figure 3.262 Recessive model of *SLC41A1* rs11240569

**Overdominant model**: The forest plot and result of recessive model of rs11240569 is shown below. Since there was no heterogeneity observed, results of fixed effect model were adopted. After meta-analysis to 1268 PD patients and 1384 controls, OR was 1.19 (1.02 – 1.38), *p* value: 0.028.

Appendix Figure 3.263 Overdominant model of *SLC41A1* rs11240569

1. ***STK32B*, rs10937625, T>C**

| Articles | PD subjects | | | Controls | | | Methods | Location of Population |
| --- | --- | --- | --- | --- | --- | --- | --- | --- |
|  | CC | CT | TT | CC | CT | TT |  |  |
| Yuan Zhang et al., 2017^a, 42^ | 14 | 123 | 373 | 17 | 133 | 386 | PCR | Changsha, Hunan Province |
| Chang-he Shi et al., 2018^a, 43^ | 20 | 164 | 362 | 12 | 165 | 373 | PCR | Chengdu, Sichuan Province |
| PCR: Polymerase chain reaction; PD: Parkinson’s disease  a: diagnostic criteria: the United Kingdom brain bank criteria ^3^ | | | | | | | | |

**Allele model**: The forest plot and result of allele model of rs10937625 is shown below. We regarded allele C as risk allele. Since there was no heterogeneity observed, results of fixed effect model were adopted. After meta-analysis to 1056 PD patients and 1086 controls, OR was 1.03 (0.88 – 1.21) compared to allele T. *p* value: 0.732.

Appendix Figure 3.264 Allele model of *STK32B* rs10937625

**Dominant model**: The forest plot and result of dominant model of rs10937625 is shown below. Since there was no heterogeneity observed, results of fixed effect model were adopted. After meta-analysis to 1056 PD patients and 1086 controls, OR was 1.01 (0.84 – 1.22), *p* value: 0.908.

Appendix Figure 3.265 Dominant model of *STK32B* rs10937625

**Recessive model**: The forest plot and result of recessive model of rs10937625 is shown below. Since there was no heterogeneity observed, results of fixed effect model were adopted. After meta-analysis to 1056 PD patients and 1086 controls, OR was 1.21 (0.73 – 2.01), *p* value: 0.452.

Appendix Figure 3.266 Recessive model of *STK32B* rs10937625

**Overdominant model**: The forest plot and result of recessive model of rs10937625 is shown below. Since there was no heterogeneity observed, results of fixed effect model were adopted. After meta-analysis to 1056 PD patients and 1086 controls, OR was 1.02 (0.84 – 1.23), *p* value: 0.867.

Appendix Figure 3.267 Overdominant model of *STK32B* rs10937625

1. ***STK39*, rs2102808, G>T**

| Articles | PD subjects | | | Controls | | | Methods | Location of Population |
| --- | --- | --- | --- | --- | --- | --- | --- | --- |
|  | GG | GT | TT | GG | GT | TT |  |  |
| Nan-Nan Li et al., 2013^a,^ ^39^ | 335 | 346 | 102 | 302 | 355 | 68 | MassARRAY | Chengdu, Sichuan Province |
| Ya-Qin Wang et al., 2014^a,^ ^125^ | 207 | 294 | 0 | 226 | 265 | 0 | PCR | Changsha, Hunan Province |
| PCR: Polymerase chain reaction; PD: Parkinson’s disease  a: diagnostic criteria: the United Kingdom brain bank criteria ^3^ | | | | | | | | |

**Allele model**: The forest plot and result of allele model of rs2102808 is shown below. We regarded allele T as risk allele. Since there was no heterogeneity observed, results of fixed effect model were adopted. After meta-analysis to 1284 PD patients and 1216 controls, OR was 1.08 (0.96 – 1.22) compared to allele G. *p* value: 0.198.

Appendix Figure 3.268 Allele model of *STK39* rs2102808

**Dominant model**: The forest plot and result of dominant model of rs2102808 is shown below. Since there was heterogeneity observed, results of random effect model were adopted. After meta-analysis to 1284 PD patients and 1216 controls, OR was 1.06 (0.84 – 1.34), *p* value: 0.606.

Appendix Figure 3.269 Dominant model of *STK39* rs2102808

**Recessive model**: It was not applied to perform meta-analysis due to lack of mutant homozygote in Ya-Qin Wang’s study.

**Overdominant model**: The forest plot and result of recessive model of rs2102808 is shown below. Since there was heterogeneity observed, results of random effect model were adopted. After meta-analysis to 1284 PD patients and 1216 controls, OR was 1.01 (0.69 – 1.47), *p* value: 0.968.

Appendix Figure 3.270 Overdominant model of *STK39* rs2102808

1. ***NQO1*, C609T**

| Articles | PD subjects | | | Controls | | | Methods | Location of Population |
| --- | --- | --- | --- | --- | --- | --- | --- | --- |
|  | CC | CT | TT | CC | CT | TT |  |  |
| Ming Shao et al., 2001^a,126^ | 13 | 94 | 19 | 41 | 73 | 22 | PCR-RFLP | Guangzhou, Guangdong Province |
| X.H. Jiang et al., 2004^b,127^ | 73 | 139 | 62 | 36 | 106 | 19 | PCR-DHPLC | Beijing |
| DHPLC: Denaturing High Pressure Liquid Chromatography; PCR: Polymerase chain reaction; PD: Parkinson’s disease; RFLP: restricted fragment length polymorphism  a: diagnostic criteria: brought up on the first extrapyramidal disease conference in China.^35^  b: diagnostic criteria: CAPIT criteria^6^ | | | | | | | | |

**Allele model**: The forest plot and result of allele model of C609T is shown below. We regarded allele T as risk allele. Since there was no heterogeneity observed, results of fixed effect model were adopted. After meta-analysis to 400 PD patients and 297 controls, OR was 1.26 (1.01 – 1.56) compared to allele C. *p* value: 0.039.

Appendix Figure 3.271 Allele model of *NQO1* C609T

**Dominant model**: The forest plot and result of dominant model of C609T is shown below. Since there was heterogeneity observed, results of random effect model were adopted. After meta-analysis to 400 PD patients and 297 controls, OR was 1.69 (0.37 – 7.76), *p* value: 0.501.

Appendix Figure 3.272 Dominant model of *NQO1* C609T

**Recessive model**: The forest plot and result of recessive model of C609T is shown below. Since there was heterogeneity observed, results of random effect model were adopted. After meta-analysis to 400 PD patients and 297 controls, OR was 1.45 (0.62 – 3.38), *p* value: 0.392.

Appendix Figure 3.273 Recessive model of *NQO1* C609T

**Overdominant model**: The forest plot and result of recessive model of C609T is shown below. Since there was heterogeneity observed, results of random effect model were adopted. After meta-analysis to 400 PD patients and 297 controls, OR was 0.87 (0.19 – 3.99), *p* value: 0.855.

Appendix Figure 3.274 Overdominant model of *NQO1* C609T

1. ***SNCA*, rs2301134, T>C**

| Articles | PD subjects | | | Controls | | | Methods | Location of Population |
| --- | --- | --- | --- | --- | --- | --- | --- | --- |
|  | CC | CT | TT | CC | CT | TT |  |  |
| Jie Fang et al., 2016^a,128^ | 437 | 132 | 14 | 436 | 108 | 9 | SnapShot | Xiamen, Fujian Province |
| Yah-Huei Wu-Chou et al., 2013^a,88^ | 492 | 127 | 7 | 339 | 127 | 7 | TaqMan | Taiwan |
| PD: Parkinson’s disease  a: diagnostic criteria: the United Kingdom brain bank criteria ^3^ | | | | | | | | |

**Allele model**: The forest plot and result of allele model of rs2301134 is shown below. We regarded allele C as risk allele. Since there was heterogeneity observed, results of random effect model were adopted. After meta-analysis to 1209 PD patients and 1026 controls, OR was 1.06 (0.63 – 1.78) compared to allele T. *p* value: 0.838.

Appendix Figure 3.275 Allele model of *SNCA* rs2301134

**Dominant model**: The forest plot and result of dominant model of rs2301134 is shown below. Since there was no heterogeneity observed, results of fixed effect model were adopted. After meta-analysis to 1209 PD patients and 1026 controls, OR was 0.87 (0.45 – 1.68), *p* value: 0.686.

Appendix Figure 3.276 Dominant model of *SNCA* rs2301134

**Recessive model**: The forest plot and result of recessive model of rs2301134 is shown below. Since there was heterogeneity observed, results of random effect model were adopted. After meta-analysis to 1209 PD patients and 1026 controls, OR was 1.08 (0.60 – 1.93), *p* value: 0.795.

Appendix Figure 3.277 Recessive model of *SNCA* rs2301134

**Overdominant model**: The forest plot and result of recessive model of rs2301134 is shown below. Since there was heterogeneity observed, results of random effect model were adopted. After meta-analysis to 1209 PD patients and 1026 controls, OR was 1.09 (0.64 – 1.88), *p* value: 0.745.

Appendix Figure 3.278 Overdominant model of *SNCA* rs2301134

1. ***SNCA*, rs11931074, G>T**

| Articles | PD subjects | | | Controls | | | Methods | Location of Population |
| --- | --- | --- | --- | --- | --- | --- | --- | --- |
|  | GG | GT | TT | GG | GT | TT |  |  |
| Yah-Huei Wu-Chou et al., 2013^a,88^ | 103 | 301 | 229 | 87 | 228 | 129 | TaqMan | Taiwan |
| YongPing Chen et al., 2015^a,129^ | 205 | 600 | 464 | 178 | 437 | 225 | MassARRAY | Chengdu, Sichuan Province |
| Ji-Feng Guo et al., 2015^a,22^ | 148 | 532 | 341 | 225 | 545 | 262 | PCR | Changsha, Hunan Province |
| PCR: Polymerase chain reaction; PD: Parkinson’s disease  a: diagnostic criteria: the United Kingdom brain bank criteria ^3^ | | | | | | | | |

**Allele model**: The forest plot and result of allele model of rs11931074 is shown below. We regarded allele T as risk allele. Since there was no heterogeneity observed, results of fixed effect model were adopted. After meta-analysis to 2923 PD patients and 2316 controls, OR was 1.33 (1.23 – 1.44) compared to allele G. *p* value: <0.0001.

Appendix Figure 3.279 Allele model of *SNCA* rs11931074

**Dominant model**: The forest plot and result of dominant model of rs11931074 is shown below. Since there was no heterogeneity observed, results of fixed effect model were adopted. After meta-analysis to 2923 PD patients and 2316 controls, OR was 1.46 (1.26 – 1.68), *p* value: <0.0001.

Appendix Figure 3.280 Dominant model of *SNCA* rs11931074

**Recessive model**: The forest plot and result of recessive model of rs11931074 is shown below. Since there was no heterogeneity observed, results of fixed effect model were adopted. After meta-analysis to 2923 PD patients and 2316 controls, OR was 1.49 (1.32 – 1.68), *p* value: <0.0001.

Appendix Figure 3.281 Recessive model of *SNCA* rs11931074

**Overdominant model**: The forest plot and result of recessive model of rs11931074 is shown below. Since there was no heterogeneity observed, results of fixed effect model were adopted. After meta-analysis to 2923 PD patients and 2316 controls, OR was 1.12 (1.01 – 1.25), *p* value: 0.036.

Appendix Figure 3.282 Overdominant model of *SNCA* rs11931074

1. ***SNCA*, rs2301135, G>C**

| Articles | PD subjects | | | Controls | | | Methods | Location of Population |
| --- | --- | --- | --- | --- | --- | --- | --- | --- |
|  | CC | CG | GG | CC | CG | GG |  |  |
| Jie Fang et al., 2016^a,128^ | 544 | 0 | 39 | 548 | 0 | 5 | SnapShot | Xiamen, Fujian Province |
| Yah-Huei Wu-Chou et al., 2013^a,88^ | 4 | 125 | 497 | 13 | 128 | 332 | TaqMan | Taiwan |
| PCR: Polymerase chain reaction; PD: Parkinson’s disease  a: diagnostic criteria: the United Kingdom brain bank criteria ^3^ | | | | | | | | |

**Allele model**: The forest plot and result of allele model of rs2301135 is shown below. We regarded allele C as risk allele. Since there was heterogeneity observed, results of random effect model were adopted. After meta-analysis to 1209 PD patients and 2026 controls, OR was 0.29 (0.06 - 1.38) compared to allele G. *p* value: 0.119.

Appendix Figure 3.283 Allele model of *SNCA* rs2301135

**Dominant model**: The forest plot and result of dominant model of rs2301135 is shown below. Since there was heterogeneity observed, results of random effect model were adopted. After meta-analysis to 1209 PD patients and 2026 controls, OR was 0.30 (0.06 – 1.41), *p* value: 0.127.

Appendix Figure 3.284 Dominant model of *SNCA* rs2301135

**Recessive model**: The forest plot and result of recessive model of rs2301135 is shown below. Since there was no heterogeneity observed, results of fixed effect model were adopted. After meta-analysis to 1209 PD patients and 2026 controls, OR was 0.16 (0.08 – 0.32), *p* value: <0.0001.

Appendix Figure 3.285 Recessive model of *SNCA* rs2301135

**Overdominant model**: It was not applied to perform meta-analysis due to lack of heterozygote of Jie Fang’s study.

1. ***SNCA*, rs2736990, C>T**

| Articles | PD subjects | | | Controls | | | Methods | Location of Population |
| --- | --- | --- | --- | --- | --- | --- | --- | --- |
|  | CC | CT | TT | CC | CT | TT |  |  |
| Fenghua Pan et al.,2013^a,130^ | 230 | 230 | 55 | 180 | 196 | 74 | PCR-LDR | Jiangsu Province |
| Rong Gan et al., 2012^a,131^ | 80 | 87 | 22 | 79 | 78 | 32 | PCR-RFLP | Guangzhou, Guangdong Province |
| Xiao Yan Guo et al., 2014^a,132^ | 429 | 459 | 123 | 259 | 356 | 106 | PCR | Chengdu, Sichuan Province |
| LDR: ligase detection reaction; PCR: Polymerase chain reaction; PD: Parkinson’s disease; RFLP: restricted fragment length polymorphism  a: diagnostic criteria: the United Kingdom brain bank criteria ^3^ | | | | | | | | |

**Allele model**: The forest plot and result of allele model of rs2736990 is shown below. We regarded allele T as risk allele. Since there was no heterogeneity observed, results of fixed effect model were adopted. After meta-analysis to 1715 PD patients and 1360 controls, OR was 0.82 (0.74 – 0.91) compared to allele C. *p* value: 0.0002.

Appendix Figure 3.286 Allele model of *SNCA* rs2736990

**Dominant model**: The forest plot and result of dominant model of rs2736990 is shown below. Since there was no heterogeneity observed, results of fixed effect model were adopted. After meta-analysis to 1715 PD patients and 1360 controls, OR was 0.81 (0.70 – 0.93), *p* value: 0.004.

Appendix Figure 3.287 Dominant model of *SNCA* rs2736990

**Recessive model**: The forest plot and result of recessive model of rs2736990 is shown below. Since there was no heterogeneity observed, results of fixed effect model were adopted. After meta-analysis to 1715 PD patients and 1360 controls, OR was 0.72 (0.58 – 0.88), *p* value: 0.002.

Appendix Figure 3.288 Recessive model of *SNCA* rs2736990

**Overdominant model**: The forest plot and result of recessive model of rs2736990 is shown below. Since there was no heterogeneity observed, results of fixed effect model were adopted. After meta-analysis to 1715 PD patients and 1360 controls, OR was 1.05 (0.91 – 1.21), *p* value: 0.484.

Appendix Figure 3.289 Overdominant model of *SNCA* rs2736990

1. ***SNCA*, rs356165, G>A**

| Articles | PD subjects | | | Controls | | | Methods | Location of Population |
| --- | --- | --- | --- | --- | --- | --- | --- | --- |
|  | AA | AG | GG | AA | AG | GG |  |  |
| Yah-Huei Wu-Chou et al., 2013^a,88^ | 100 | 322 | 204 | 77 | 275 | 121 | TaqMan | Taiwan |
| F.-Y. Hu et al., 2010^a,133^ | 71 | 173 | 86 | 64 | 163 | 73 | PCR-RFLP | Chengdu, Sichuan Province |
| Ji-Feng Guo et al., 2015^a,22^ | 165 | 524 | 330 | 214 | 560 | 256 | PCR | Changsha, Hunan Province |
| PCR: Polymerase chain reaction; PD: Parkinson’s disease; RFLP: restricted fragment length polymorphism  a: diagnostic criteria: the United Kingdom brain bank criteria ^3^ | | | | | | | | |

**Allele model**: The forest plot and result of allele model of rs356165 is shown below. We regarded allele A as risk allele. Since there was no heterogeneity observed, results of fixed effect model were adopted. After meta-analysis to 1975 PD patients and 1803 controls, OR was 0.83 (0.76 – 0.91) compared to allele G. *p* value: <0.0001.

Appendix Figure 3.290 Allele model of *SNCA* rs356165

**Dominant model**: The forest plot and result of dominant model of rs356165 is shown below. Since there was no heterogeneity observed, results of fixed effect model were adopted. After meta-analysis to 1975 PD patients and 1803 controls, OR was 0.73 (0.63 – 0.84), *p* value: <0.0001.

Appendix Figure 3.291 Dominant model of *SNCA* rs356165

**Recessive model**: The forest plot and result of recessive model of rs356165 is shown below. Since there was no heterogeneity observed, results of fixed effect model were adopted. After meta-analysis to 1975 PD patients and 1803 controls, OR was 0.84 (0.71 – 0.99), *p* value: 0.042.

Appendix Figure 3.292 Recessive model of *SNCA* rs356165

**Overdominant model**: The forest plot and result of recessive model of rs356165 is shown below. Since there was no heterogeneity observed, results of fixed effect model were adopted. After meta-analysis to 1975 PD patients and 1803 controls, OR was 1.17 (1.03 – 1.33), *p* value: 0.018.

Appendix Figure 3.293 Overdominant model of *SNCA* rs356165

1. ***SNCA*, rs356219, G>A**

| Articles | PD subjects | | | Controls | | | Methods | Location of Population |
| --- | --- | --- | --- | --- | --- | --- | --- | --- |
|  | AA | AG | GG | AA | AG | GG |  |  |
| Yah-Huei Wu-Chou et al., 2013^a,88^ | 107 | 300 | 226 | 84 | 221 | 139 | TaqMan | Taiwan |
| Wen-Juan Yu et al., 2015^a,59^ | 51 | 235 | 223 | 73 | 243 | 109 | MassARRAY | Chengdu, Sichuan Province |
| Rong Gan et al., 2012^a,131^ | 31 | 89 | 69 | 48 | 82 | 59 | PCR-RFLP | Guangzhou, Guangdong Province |
| Nan-Nan Li et al., 2013^a,134^ | 72 | 301 | 312 | 109 | 312 | 148 | MassARRAY | Chengdu, Sichuan Province |
| Fenghua Pan et al., 2012^b,135^ | 54 | 179 | 170 | 71 | 142 | 102 | PCR-LDR | Jiangsu Province |
| LDR: ligase detection reaction; PCR: Polymerase chain reaction; PD: Parkinson’s disease; RFLP: restricted fragment length polymorphism  a: diagnostic criteria: the United Kingdom brain bank criteria ^3^  b: diagnostic criteria: brought up by Calne et al.^78^ | | | | | | | | |

**Allele model**: The forest plot and result of allele model of rs356219 is shown below. We regarded allele A as risk allele. Since there was heterogeneity observed, results of random effect model were adopted. After meta-analysis to 2419 PD patients and 1942 controls, OR was 0.67 (0.56 – 0.81) compared to allele G. *p* value: <0.0001.

Appendix Figure 3.294 Allele model of *SNCA* rs356219

**Dominant model**: The forest plot and result of dominant model of rs356219 is shown below. Since there was heterogeneity observed, results of random effect model were adopted. After meta-analysis to 2419 PD patients and 1942 controls, OR was 0.59 (0.44- 0.80), *p* value: 0.0005.

Appendix Figure 3.295 Dominant model of *SNCA* rs356219

**Recessive model**: The forest plot and result of recessive model of rs356219 is shown below. Since there was no heterogeneity observed, results of fixed effect model were adopted. After meta-analysis to 2419 PD patients and 1942 controls, OR was 0.60 (0.51 – 0.71), *p* value: <0.0001.

Appendix Figure 3.296 Recessive model of *SNCA* rs356219

**Overdominant model**: The forest plot and result of recessive model of rs356219 is shown below. Since there was heterogeneity observed, results of random effect model were adopted. After meta-analysis to 2419 PD patients and 1942 controls, OR was 1.22 (0.98 – 1.51), *p* value: 0.072.

Appendix Figure 3.297 Overdominant model of *SNCA* rs356219

1. ***SNCA*, rs356221, A>T**

| Articles | PD subjects | | | Controls | | | Methods | Location of Population |
| --- | --- | --- | --- | --- | --- | --- | --- | --- |
|  | AA | AT | TT | AA | AT | TT |  |  |
| Jie Fang et al., 2016^a,128^ | 210 | 282 | 91 | 231 | 244 | 78 | SnapShot | Xiamen, Fujian Province |
| Yah-Huei Wu-Chou et al., 2013^a,88^ | 275 | 282 | 69 | 172 | 240 | 61 | TaqMan | Taiwan |
| PD: Parkinson’s disease  a: diagnostic criteria: the United Kingdom brain bank criteria ^3^ | | | | | | | | |

**Allele model**: The forest plot and result of allele model of rs356221 is shown below. We regarded allele T as risk allele. Since there was heterogeneity observed, results of random effect model were adopted. After meta-analysis to 1209 PD patients and 1026 controls, OR was 0.98 (0.69 – 1.39) compared to allele A. *p* value: 0.890.

Appendix Figure 3.298 Allele model of *SNCA* rs356221

**Dominant model**: The forest plot and result of dominant model of rs356221 is shown below. Since there was heterogeneity observed, results of random effect model were adopted. After meta-analysis to 1209 PD patients and 1026 controls, OR was 0.96 (0.56 – 1.67), *p* value: 0.897.

Appendix Figure 3.299 Dominant model of *SNCA* rs356221

**Recessive model**: The forest plot and result of recessive model of rs356221 is shown below. Since there was no heterogeneity observed, results of fixed effect model were adopted. After meta-analysis to 1209 PD patients and 1026 controls, OR was 0.99 (0.77 – 1.26), *p* value: 0.922.

Appendix Figure 3.300 Recessive model of *SNCA* rs356221

**Overdominant model**: The forest plot and result of recessive model of rs356221 is shown below. Since there was heterogeneity observed, results of random effect model were adopted. After meta-analysis to 1209 PD patients and 1026 controls, OR was 1.03 (0.70 – 1.52), *p* value: 0.889.

Appendix Figure 3.301 Overdominant model of *SNCA* rs356221

1. ***SNCA*, rs3822086, C>T**

| Articles | PD subjects | | | Controls | | | Methods | Location of Population |
| --- | --- | --- | --- | --- | --- | --- | --- | --- |
|  | CC | CT | TT | CC | CT | TT |  |  |
| YongPing Chen et al., 2015^a,129^ | 208 | 598 | 465 | 181 | 432 | 226 | MassARRAY | Chengdu, Sichuan Province |
| Chong-Dong Jian et al., 2015^b,136^ | 33 | 76 | 37 | 49 | 68 | 27 | PCR-RFLP | Baise, Guangxi Province |
| Hanchun Long et al., 2016^a,118^ | 8 | 33 | 19 | 14 | 29 | 17 | PCR | Xingyi, Guizhou Province |
| Hanchun Long et al., 2016^a,*,118^ | 23 | 28 | 11 | 25 | 26 | 9 | PCR | Xingyi, Guizhou Province |
| Hanchun Long et al., 2016^a,†,118^ | 24 | 30 | 12 | 25 | 25 | 10 | PCR | Xingyi, Guizhou Province |
| PCR: Polymerase chain reaction; PD: Parkinson’s disease; RFLP: restricted fragment length polymorphism  a: diagnostic criteria: the United Kingdom brain bank criteria ^3^  b: diagnostic criteria: brought up by Brook DJ et al.^137^  *: Population source was from Buyi ethnicity. This study was not included into meta-analysis due to different population source.  †:Population source was from Miao ethnicity. This study was not included into meta-analysis due to different population source. | | | | | | | | |

**Allele model**: The forest plot and result of allele model of rs356221 is shown below. We regarded allele T as risk allele. Since there was no heterogeneity observed, results of fixed effect model were adopted. After meta-analysis to 1477 PD patients and 1043 controls, OR was 1.36 (1.22 – 1.52) compared to allele C. *p* value: <0.0001.

Appendix Figure 3.302 Allele model of *SNCA* rs356221

**Dominant model**: The forest plot and result of dominant model of rs356221 is shown below. Since there was no heterogeneity observed, results of fixed effect model were adopted. After meta-analysis to 1477 PD patients and 1043 controls, OR was 1.48 (1.21 – 1.80), *p* value: 0.0001.

Appendix Figure 3.303 Dominant model of *SNCA* rs356221

**Recessive model**: The forest plot and result of recessive model of rs356221 is shown below. Since there was no heterogeneity observed, results of fixed effect model were adopted. After meta-analysis to 1477 PD patients and 1043 controls, OR was 1.53 (1.29 – 1.83), *p* value: <0.0001.

Appendix Figure 3.304 Recessive model of *SNCA* rs356221

**Overdominant model**: The forest plot and result of recessive model of rs356221 is shown below. Since there was no heterogeneity observed, results of fixed effect model were adopted. After meta-analysis to 1477 PD patients and 1043 controls, OR was 1.12 (0.95 – 1.31), *p* value: 0.170.

Appendix Figure 3.305 Overdominant model of *SNCA* rs356221

1. ***UCH-L1*, rs5030732, C54A, S18Y**

| Articles | PD subjects | | | Controls | | | Methods | Location of Population |
| --- | --- | --- | --- | --- | --- | --- | --- | --- |
|  | AA | AC | CC | AA | AC | CC |  |  |
| Lei Wang et al., 2011^a,138^ | 108 | 198 | 102 | 112 | 200 | 86 | PCR-RFLP | Changsha, Hunan Province |
| Z.-J. Zhang et al., 2008^a,139^ | 152 | 336 | 112 | 76 | 197 | 61 | PCR-RFLP | Chengdu, Sichuan Province |
| Jian Wang et al., 2002^b,140^ | 38 | 82 | 40 | 35 | 80 | 45 | PCR-RFLP | Guangzhou, Guangdong Province |
| Yih-Ru Wu et al., 2010^b,115^ | 144 | 261 | 112 | 143 | 263 | 112 | PCR-RFLP | Taiwan |
| Yixin Hao et al., 2008^c,141^ | 20 | 84 | 60 | 34 | 98 | 40 | PCR-RFLP | Shanghai |
| PCR: Polymerase chain reaction; PD: Parkinson’s disease; RFLP: restricted fragment length polymorphism  a: diagnostic criteria: the United Kingdom brain bank criteria ^3^  b: diagnostic criteria: brought up by Gelb et al.^15^  c: diagnostic criteria: brought up on the first extrapyramidal disease conference in China.^35^ | | | | | | | | |

**Allele model**: The forest plot and result of allele model of rs5030732 is shown below. We regarded allele A as risk allele. Since there was heterogeneity observed, results of random effect model were adopted. After meta-analysis to 1849 PD patients and 1582 controls, OR was 0.94 (0.82 – 1.09) compared to allele C. *p* value: 0.425.

Appendix Figure 3.306 Allele model of *UCH-L1* rs5030732

**Dominant model**: The forest plot and result of dominant model of rs5030732 is shown below. Since there was no heterogeneity observed, results of fixed effect model were adopted. After meta-analysis to 1849 PD patients and 1582 controls, OR was 0.89 (0.76 – 1.05), *p* value: 0.279.

Appendix Figure 3.307 Dominant model of *UCH-L1* rs5030732

**Recessive model**: The forest plot and result of recessive model of rs5030732 is shown below. Since there was no heterogeneity observed, results of fixed effect model were adopted. After meta-analysis to 1849 PD patients and 1582 controls, OR was 0.99 (0.84 – 1.15), *p* value: 0.870.

Appendix Figure 3.308 Recessive model of *UCH-L1* rs5030732

**Overdominant model**: The forest plot and result of recessive model of rs5030732 is shown below. Since there was no heterogeneity observed, results of fixed effect model were adopted. After meta-analysis to 1849 PD patients and 1582 controls, OR was 1.07 (0.94 – 1.23), *p* value: 0.317.

Appendix Figure 3.309 Overdominant model of *UCH-L1* rs5030732

1. ***VDR*, rs4334089, G>A**

| Articles | PD subjects | | | Controls | | | Methods | Location of Population |
| --- | --- | --- | --- | --- | --- | --- | --- | --- |
|  | AA | AG | GG | AA | AG | GG |  |  |
| Zhanyun Lv et al., 2013^a,142^ | 87 | 249 | 147 | 98 | 244 | 156 | PCR-RFLP | Changsha, Hunan Province |
| Chin-Hsien Lin et al., 2014^a,143^ | 116 | 361 | 223 | 138 | 409 | 245 | TaqMan | Taiwan |
| PCR: Polymerase chain reaction; PD: Parkinson’s disease; RFLP: restricted fragment length polymorphism  a: diagnostic criteria: the United Kingdom brain bank criteria ^3^ | | | | | | | | |

**Allele model**: The forest plot and result of allele model of rs4334089 is shown below. We regarded allele A as risk allele. Since there was no heterogeneity observed, results of fixed effect model were adopted. After meta-analysis to 1183 PD patients and 1290 controls, OR was 0.97 (0.87 – 1.09) compared to allele G. *p* value: 0.625.

Appendix Figure 3.310 Allele model of *VDR* rs4334089

**Dominant model**: The forest plot and result of dominant model of rs4334089 is shown below. Since there was no heterogeneity observed, results of fixed effect model were adopted. After meta-analysis to 1183 PD patients and 1290 controls, OR was 0.99 (0.84 – 1.17), *p* value: 0.914.

Appendix Figure 3.311 Dominant model of *VDR* rs4334089

**Recessive model**: The forest plot and result of recessive model of rs4334089 is shown below. Since there was no heterogeneity observed, results of fixed effect model were adopted. After meta-analysis to 1183 PD patients and 1290 controls, OR was 0.92 (0.75 – 1.13), *p* value: 0.445.

Appendix Figure 3.312 Recessive model of *VDR* rs4334089

**Overdominant model**: The forest plot and result of recessive model of rs4334089 is shown below. Since there was no heterogeneity observed, results of fixed effect model were adopted. After meta-analysis to 1183 PD patients and 1290 controls, OR was 0.96 (0.82 – 1.13), *p* value: 0.628.

Appendix Figure 3.313 Overdominant model of *VDR* rs4334089

1. ***VDR*, rs731236, T>C**

| Articles | PD subjects | | | Controls | | | Methods | Location of Population |
| --- | --- | --- | --- | --- | --- | --- | --- | --- |
|  | CC | CT | TT | CC | CT | TT |  |  |
| Hongxin Liu et al., 2013^a,144^ | 0 | 33 | 252 | 0 | 30 | 255 | PCR-RFLP | Qingdao, Shandong Province |
| Zhanyun Lv et al., 2013^b, 142^ | 0 | 46 | 437 | 0 | 52 | 446 | PCR-RFLP | Changsha, Hunan Province |
| PCR: Polymerase chain reaction; PD: Parkinson’s disease; RFLP: restricted fragment length polymorphism  a: diagnostic criteria: brought up by CMDS^36^  b: diagnostic criteria: the United Kingdom brain bank criteria ^3^ | | | | | | | | |

**Allele model**: The forest plot and result of allele model of rs731236 is shown below. We regarded allele C as risk allele. Since there was no heterogeneity observed, results of fixed effect model were adopted. After meta-analysis to 768 PD patients and 783 controls, OR was 0.98 (0.71 – 1.35) compared to allele T. *p* value: 0.903.

Appendix Figure 3.314 Allele model of *VDR* rs731236

**Dominant model**: The forest plot and result of dominant model of rs731236 is shown below. Since there was no heterogeneity observed, results of fixed effect model were adopted. After meta-analysis to 768 PD patients and 783 controls, OR was 0.98 (0.71 – 1.36), *p* value: 0.901.

Appendix Figure 3.315 Dominant model of *VDR* rs731236

**Recessive model**: It was not applied to perform meta-analysis because of lack of mutant homozygote.

**Overdominant model**: The forest plot and result of recessive model of rs731236 is shown below. Since there was no heterogeneity observed, results of fixed effect model were adopted. After meta-analysis to 768 PD patients and 783 controls, OR was 1.02 (0.74 – 1.42), *p* value: 0.901.

Appendix Figure 3.316 Overdominant model of *VDR* rs731236

**Reference**

1. Zhang X, Cheng X, Hu YB, et al. Serotonin transporter polymorphic region 5-HTTLPR modulates risk for Parkinson's disease. *Neurobiology of aging* 2014; **35**(8): 1957.e9-.e14.

2. Liu W, Pan X, Ma A, Zhang Z, Lu K. The association between polymorphism of SLC6A4 gene and depression in Parkinson's disease in Chinese Han population. *Journal of Brain and Nervous Diseases* 2016; **24**(12): 739-43.

3. Hughes AJ, Daniel SE, Kilford L, Lees AJ. Accuracy of clinical diagnosis of idiopathic Parkinson's disease: a clinico-pathological study of 100 cases. 1992; **55**(3): 181-4.

4. Tang G, Zhang M, Xie H, et al. Alpha-2 macroglobulin I1000 V polymorphism in Chinese sporadic Alzheimer's disease and Parkinson's disease. *Neuroscience letters* 2002; **328**(2): 195-7.

5. Xiao Y, Zhang B. Association of the polymorphism in alpha-2 macroglobulin gene with essential tremor and Parkinson’s disease. *Chin J Med Genet,* 2006; **23**(1): 84-5.

6. Langston JW, Widner H, Goetz CG, et al. Core assessment program for intracerebral transplantations (CAPIT). *Movement disorders : official journal of the Movement Disorder Society* 1992; **7**(1): 2-13.

7. Hao Y, Xu L, Wu Q, et al. The association between both common polymorphisms of α2-macroglobulin gene and Parkinson disease. *Chinese Journal of Neurology* 2002; **35**(1): 13-5.

8. Zhang X, Ye YL, Wang YN, et al. Aldehyde dehydrogenase 2 genetic variations may increase susceptibility to Parkinson's disease in Han Chinese population. *Neurobiology of aging* 2015; **36**(9): 2660.e9-13.

9. Zhao CC, Cai HB, Wang H, Pan SY. Role of ADH2 and ALDH2 gene polymorphisms in the development of Parkinson's disease in a Chinese population. *Genetics and molecular research : GMR* 2016; **15**(3).

10. Lin CH, Tan EK, Chen ML, et al. Novel ATP13A2 variant associated with Parkinson disease in Taiwan and Singapore. 2008; **71**(21): 1727-32.

11. Fei QZ, Cao L, Xiao Q, et al. Lack of association between ATP13A2 Ala746Thr variant and Parkinson's disease in Han population of mainland China. *Neuroscience letters* 2010; **475**(2): 61-3.

12. Chen CM, Lin CH, Juan HF, et al. ATP13A2 variability in Taiwanese Parkinson's disease. *American journal of medical genetics Part B, Neuropsychiatric genetics : the official publication of the International Society of Psychiatric Genetics* 2011; **156b**(6): 720-9.

13. Chan AY, Baum L, Tang NL, et al. The role of the Ala746Thr variant in the ATP13A2 gene among Chinese patients with Parkinson's disease. *Journal of clinical neuroscience : official journal of the Neurosurgical Society of Australasia* 2013; **20**(5): 761-2.

14. Wang F. The polymorphism of ATP13A2 gene in Uygur and Han patients with Parkinson disease in Xinjiang: Xinjiang Medical University; 2013.

15. Gelb DJ, Oliver E, Gilman S. Diagnostic criteria for Parkinson disease. *Archives of neurology* 1999; **56**(1): 33-9.

16. Hu D, Liang Z, Ren X, Liu M, Wang T, Sun S. Genetic polymorphisms of brain-derived neurotrophic factor and sporadic Parkinson disease. *CHINESE JOURNAL OF CLINICAL REHABILITATION* 2005; **9**(17): 196-7.

17. Chen L, Wang Y, Xiao H, et al. The 712A/G polymorphism of Brain-derived neurotrophic factor is associated with Parkinson's disease but not Major Depressive Disorder in a Chinese Han population. *Biochemical and biophysical research communications* 2011; **408**(2): 318-21.

18. Liu J, Zhou Y, Wang C, Wang T, Zheng Z, Chan P. Brain-derived neurotrophic factor (BDNF) genetic polymorphism greatly increases risk of leucine-rich repeat kinase 2 (LRRK2) for Parkinson's disease. *Parkinsonism & related disorders* 2012; **18**(2): 140-3.

19. Liang Q, Pang G, Lv Z, et al. Association study between rs6265 and rs4680 on sporadic Parkinson’s disease in Zhuang population. *Shaanxi Medical Journal* 2015; (4): 419-20.

20. Xie D. The study of relationship between polymorphism in BST1 gene rs4698412 and sporadic Parkinson’s disease [硕士]: University of South China; 2011.

21. Chang XL, Mao XY, Li HH, et al. Association of GWAS loci with PD in China. *American journal of medical genetics Part B, Neuropsychiatric genetics : the official publication of the International Society of Psychiatric Genetics* 2011; **156b**(3): 334-9.

22. Guo JF, Li K, Yu RL, et al. Polygenic determinants of Parkinson's disease in a Chinese population. *Neurobiology of aging* 2015; **36**(4): 1765.e1-.e6.

23. Lu Q, Deng X, Song Z, Guo Y, Yang Y, Deng H. Mutation analysis of the CHCHD2 gene in Chinese Han patients with Parkinson's disease. *Parkinsonism & related disorders* 2016; **29**: 143-4.

24. Wu H. Genetic analysis of CHCHD2 gene in Chinese patients with Parkinson disease and essential Tremor [Master]: Zhejiang University; 2017.

25. Mao C. Genetic Analysis and Pathogenic Study of Parkinson’s Disease Associated with CHCHD2 [Doctorate]: Zhengzhou University; 2018.

26. Li NN, Wang L, Tan EK, et al. Genetic analysis of CHCHD2 gene in Chinese Parkinson's disease. *American journal of medical genetics Part B, Neuropsychiatric genetics : the official publication of the International Society of Psychiatric Genetics* 2016; **171**(8): 1148-52.

27. Xie T, Ho SL, Li LS, Ma OC. G/A1947 polymorphism in catechol-O-methyltransferase (COMT) gene in Parkinson's disease. *Movement disorders : official journal of the Movement Disorder Society* 1997; **12**(3): 426-7.

28. Shao M. Correlation between the genetic polymorphism of dopamine metabolic enzymes and the genetic susceptibility of Parkinson’s disease. [Doctorate]: Zhongshan University; 1998.

29. Wu RM, Cheng CW, Chen KH, et al. The COMT L allele modifies the association between MAOB polymorphism and PD in Taiwanese. 2001; **56**(3): 375-82.

30. Xu L. Associaation analysis between dopamine metabolism system genes and Parkinson's disease [硕士]: Fudan University; 2002.

31. Zhao X, Xie H, Tang G, et al. Relationship between polymorphisms of COMT, DBH, DAT, MAOB genes and susceptibility of Parkinson′s disease in Chinese. *BULLETIN OF THE ACADEMY OF MILITARY MEDICAL SCIENCES* 2003; **27**(3): 199-201,29.

32. Qi D, Dai Y, Chen X, Li J, Zhang C. Research on relationship between COMT gene exon four and Parkinson’s genetic susceptibility. *Clinical Journal of Chinese Medicine* 2013; (21): 87-8.

33. Zhang Y, Feng S, Nie K, et al. Association of the catechol-O-methyltransferase rs4680 polymorphism with Parkinson's disease in a Han Chinese cohort. *Chinese Journal of Neurology* 2015; **48**(1): 18-22.

34. Xiao Q, Qian Y, Liu J, Xu S, Yang X. Roles of functional catechol-O-methyltransferase genotypes in Chinese patients with Parkinson's disease. *Translational neurodegeneration* 2017; **6**: 11.

35. China Tedci. Diagnostic criteria and differential diagnosis of Parkinson's disease and Parkinson's syndrome. *Chin J Neuro* 1985; **18**: 256.

36. CMDS. Diagnosis of Parkinson’s disease. *Chinese Journal of Neurology* 2006; (6): 408-9.

37. Dai Y, Jin M, Cao L, Qi D, Zhang C, Fu Y. The association between Variations of COMT gene and the genetic susceptibility of Parkinson's disease. *World Health Digest* 2012; (52): 16-8.

38. Yin B, Chen Y, Zhang L. Association Between Catechol-O-Methyltransferase (COMT) Gene Polymorphisms, Parkinson's Disease, and Levodopa Efficacy. *Molecular diagnosis & therapy* 2013.

39. Li NN, Tan EK, Chang XL, et al. Genetic association study between STK39 and CCDC62/HIP1R and Parkinson's disease. *PloS one* 2013; **8**(11): e79211.

40. Liu RR, Zhou LL, Cheng X, et al. CCDC62 variant rs12817488 is associated with the risk of Parkinson's disease in a Han Chinese population. *European neurology* 2014; **71**(1-2): 77-83.

41. Yu RL, Guo JF, Wang YQ, et al. The single nucleotide polymorphism Rs12817488 is associated with Parkinson's disease in the Chinese population. *Journal of clinical neuroscience : official journal of the Neurosurgical Society of Australasia* 2015; **22**(6): 1002-4.

42. Zhang Y, Zhao Y, Zhou X, et al. Relationship between GWAS-linked three new loci in Essential tremor and risk of Parkinson's disease in Chinese population. *Parkinsonism & related disorders* 2017; **43**: 124-6.

43. Shi CH, Cheng Y, Tang MB, et al. Analysis of Single Nucleotide Polymorphisms of STK32B, PPARGC1A and CTNNA3 Gene With Sporadic Parkinson's Disease Susceptibility in Chinese Han Population. *Frontiers in neurology* 2018; **9**: 387.

44. Liu S, Guo J, Yi C, et al. Relationship between the monocyte of the g.168_185del polymorphism of the promoter in DJ-1 gene and Parkinson's disease. *JOURNAL OF CLINICAL NEUROLOGY* 2008; **21**(4): 267-9.

45. Chen W, Peng R, Li T, et al. [Association of the DJ-1 gene polymorphism with sporadic Parkinson's disease in Sichuan province of China]. *Zhonghua yi xue yi chuan xue za zhi = Zhonghua yixue yichuanxue zazhi = Chinese journal of medical genetics* 2008; **25**(5): 566-9.

46. Li H, Yan H, Yang X. Association between the polymorphism of DJ-1 gene g.168_185del and Parkinson’s disease in Xinjiang Uygurs and Hans. *Journal of Clinical Neurology* 2012; **25**(3): 189-91.

47. Zhu LH, Luo XG, Zhou YS, et al. Lack of association between three single nucleotide polymorphisms in the PARK9, PARK15, and BST1 genes and Parkinson's disease in the northern Han Chinese population. *Chinese medical journal* 2012; **125**(4): 588-92.

48. Chen CM, Chen IC, Huang YC, et al. FBXO7 Y52C polymorphism as a potential protective factor in Parkinson's disease. *PloS one* 2014; **9**(7): e101392.

49. Sun XY, Wang L, Cheng L, et al. Genetic analysis of FGF20 in Chinese patients with Parkinson's disease. *Neurological sciences : official journal of the Italian Neurological Society and of the Italian Society of Clinical Neurophysiology* 2017; **38**(5): 887-91.

50. Jing CC, Luo XG, Cui HG, et al. Screening of polymorphisms located in the FGF20 and TMEM175 genes in North Chinese Parkinson's disease patients. *Genetics and molecular research : GMR* 2015; **14**(4): 13679-87.

51. Pan J, Li H, Wang Y, et al. Fibroblast growth factor 20 (FGF20) polymorphism is a risk factor for Parkinson's disease in Chinese population. *Parkinsonism & related disorders* 2012; **18**(5): 629-31.

52. Xu X, Wang N, Xu H, Xie A, Jiang H, Xie J. Fibroblast growth factor 20 polymorphism in sporadic Parkinson's disease in Northern Han Chinese. *Journal of clinical neuroscience : official journal of the Neurosurgical Society of Australasia* 2013; **20**(11): 1588-90.

53. Lei Y, Qi Z, Mu W, Wang Z. Correlation analysis between single nucleotide polymorphisms of fibroblast growth factor 20 (FGF20) gene and Parkinson disease. *Medical Journal of Chinese People's Liberation Army* 2014; **39**(2): 129-32.

54. Yuan L, Song Z, Deng X, et al. Systematic analysis of genetic variants in Han Chinese patients with sporadic Parkinson's disease. *Scientific reports* 2016; **6**: 33850.

55. Zhou LL, Zhang X, Bao QQ, et al. Association analysis of PARK16-18 variants and Parkinson's disease in a Chinese population. *Journal of clinical neuroscience : official journal of the Neurosurgical Society of Australasia* 2014; **21**(6): 1029-32.

56. Li NN, Chang XL, Mao XY, et al. GWAS-linked GAK locus in Parkinson's disease in Han Chinese and meta-analysis. *Human genetics* 2012; **131**(7): 1089-93.

57. Chen YP, Song W, Huang R, et al. GAK rs1564282 and DGKQ rs11248060 increase the risk for Parkinson's disease in a Chinese population. *Journal of clinical neuroscience : official journal of the Neurosurgical Society of Australasia* 2013; **20**(6): 880-3.

58. Tseng WE, Chen CM, Chen YC, Yi Z, Tan EK, Wu YR. Genetic variations of GAK in two Chinese Parkinson's disease populations: a case-control study. *PloS one* 2013; **8**(6): e67506.

59. Yu WJ, Cheng L, Li NN, Wang L, Tan EK, Peng R. Interaction between SNCA, LRRK2 and GAK increases susceptibility to Parkinson's disease in a Chinese population. *eNeurologicalSci* 2015; **1**(1): 3-6.

60. Mao XY, Burgunder JM, Zhang ZJ, et al. Association between GBA L444P mutation and sporadic Parkinson's disease from Mainland China. *Neuroscience letters* 2010; **469**(2): 256-9.

61. Ziegler SG, Eblan MJ, Gutti U, et al. Glucocerebrosidase mutations in Chinese subjects from Taiwan with sporadic Parkinson disease. *Molecular genetics and metabolism* 2007; **91**(2): 195-200.

62. Yu Z, Wang T, Xu J, et al. Mutations in the glucocerebrosidase gene are responsible for Chinese patients with Parkinson's disease. *Journal of human genetics* 2015; **60**(2): 85-90.

63. Yang X, Zheng J, An R, et al. Polymorphism in MIR4697 but not VPS13C, GCH1, or SIPA1L2 is associated with risk of Parkinson's disease in a Han Chinese population. *Neuroscience letters* 2017; **650**: 8-11.

64. Zou M, Li R, Wang JY, et al. Association analyses of variants of SIPA1L2, MIR4697, GCH1, VPS13C, and DDRGK1 with Parkinson's disease in East Asians. *Quality of life research : an international journal of quality of life aspects of treatment, care and rehabilitation* 2018; **68**: 159.e7-.e14.

65. Yu L, Huang J, Zhai D, et al. MAPT rs242562 and GSK3B rs334558 are associated with Parkinson's Disease in central China. *BMC neuroscience* 2014; **15**: 54.

66. Zhao DM, Li NN, Zhang JH, et al. GSK3beta reduces risk of sporadic Parkinson's disease in ethnic Chinese. *American journal of medical genetics Part B, Neuropsychiatric genetics : the official publication of the International Society of Psychiatric Genetics* 2012; **159b**(6): 718-21.

67. Liu ZH, Guo JF, Li K, et al. Analysis of several loci from genome-wide association studies in Parkinson's disease in mainland China. *Neuroscience letters* 2015; **587**: 68-71.

68. Xu Y, Chen Y, Ou R, et al. No association of GPNMB rs156429 polymorphism with Parkinson's disease, amyotrophic lateral sclerosis and multiple system atrophy in Chinese population. *Neuroscience letters* 2016; **622**: 113-7.

69. Yang X, Liu C, Zhang J, et al. Association of histamine N-methyltransferase Thr105Ile polymorphism with Parkinson's disease and schizophrenia in Han Chinese: a case-control study. *PloS one* 2015; **10**(3): e0119692.

70. Chen Y, Cao B, Ou R, et al. Determining the Effect of the HNMT, STK39, and NMD3 Polymorphisms on the Incidence of Parkinson's Disease, Amyotrophic Lateral Sclerosis, and Multiple System Atrophy in Chinese Populations. *Molecular neurobiology* 2018; **64**(4): 574-80.

71. Guo Y, Deng X, Zheng W, et al. HLA rs3129882 variant in Chinese Han patients with late-onset sporadic Parkinson disease. *Neuroscience letters* 2011; **501**(3): 185-7.

72. Chiang HL, Lee-Chen GJ, Chen CM, et al. Genetic analysis of HLA-DRA region variation in Taiwanese Parkinson's disease. *Parkinsonism & related disorders* 2012; **18**(4): 391-3.

73. Lin CH, Chen ML, Tai YC, Yu CY, Wu RM. Reaffirmation of GAK, but not HLA-DRA, as a Parkinson's disease susceptibility gene in a Taiwanese population. *American journal of medical genetics Part B, Neuropsychiatric genetics : the official publication of the International Society of Psychiatric Genetics* 2013; **162b**(8): 841-6.

74. Mo MS, Xiao YS, Wu ZH, et al. Association analysis of HLA-DRA in Chinese patients with sporadic Parkinson's disease. *International journal of physiology, pathophysiology and pharmacology* 2015; **7**(4): 185-94.

75. Jankovic J. Parkinson's disease: clinical features and diagnosis. *Journal of neurology, neurosurgery, and psychiatry* 2008; **79**(4): 368-76.

76. Li D, He Q, Li R, Xu X, Chen B, Xie A. Interleukin-10 promoter polymorphisms in Chinese patients with Parkinson's disease. *Neuroscience letters* 2012; **513**(2): 183-6.

77. Liu Z, Guo J, Wang Y, et al. Lack of association between IL-10 and IL-18 gene promoter polymorphisms and Parkinson's disease with cognitive impairment in a Chinese population. *Scientific reports* 2016; **6**: 19021.

78. Calne DB, Snow BJ, Lee C. Criteria for diagnosing Parkinson's disease. *Annals of neurology* 1992; **32 Suppl**: S125-7.

79. Xu X, Li D, He Q, Gao J, Chen B, Xie A. Interleukin-18 promoter polymorphisms and risk of Parkinson's disease in a Han Chinese population. *Brain research* 2011; **1381**: 90-4.

80. Zuo X, Jiang H, Guo JF, et al. Screening for two SNPs of LINGO1 gene in patients with essential tremor or sporadic Parkinson's disease in Chinese population. *Neuroscience letters* 2010; **481**(2): 69-72.

81. Guo Y, Jankovic J, Song Z, et al. LINGO1 rs9652490 variant in Parkinson disease patients. *Neuroscience letters* 2011; **487**(2): 174-6.

82. Chen Y, Cao B, Yang J, et al. Analysis and meta-analysis of five polymorphisms of the LINGO1 and LINGO2 genes in Parkinson's disease and multiple system atrophy in a Chinese population. *Journal of neurology* 2015; **262**(11): 2478-83.

83. Wu YW, Prakash KM, Rong TY, et al. Lingo2 variants associated with essential tremor and Parkinson's disease. *Human genetics* 2011; **129**(6): 611-5.

84. Di Fonzo A, Wu-Chou Y-H, Lu C-S, et al. A common missense variant in the LRRK2 gene, Gly2385Arg, associated with Parkinson’s disease risk in Taiwan. 2006; **7**(3): 133-8.

85. Li NN, Tan EK, Chang XL, et al. Genetic analysis of LRRK2 A419V variant in ethnic Chinese. *Neurobiology of aging* 2012; **33**(8): 1849.e1-3.

86. Li K, Tang BS, Liu ZH, et al. LRRK2 A419V variant is a risk factor for Parkinson's disease in Asian population. *Neurobiology of aging* 2015; **36**(10): 2908.e11-5.

87. Yao LY, Guo JF, Wang L, et al. LRRK2 Pro755Leu variant in ethnic Chinese population with Parkinson's disease. *Neuroscience letters* 2011; **495**(1): 35-8.

88. Wu-Chou YH, Chen YT, Yeh TH, et al. Genetic variants of SNCA and LRRK2 genes are associated with sporadic PD susceptibility: a replication study in a Taiwanese cohort. *Parkinsonism & related disorders* 2013; **19**(2): 251-5.

89. Fung HC, Chen CM, Hardy J, Singleton AB, Wu YR. A common genetic factor for Parkinson disease in ethnic Chinese population in Taiwan. *BMC neurology* 2006; **6**: 47.

90. Li C, Ting Z, Qin X, et al. The prevalence of LRRK2 Gly2385Arg variant in Chinese Han population with Parkinson's disease. *Movement disorders : official journal of the Movement Disorder Society* 2007; **22**(16): 2439-43.

91. An XK, Peng R, Li T, et al. LRRK2 Gly2385Arg variant is a risk factor of Parkinson's disease among Han-Chinese from mainland China. *European journal of neurology* 2008; **15**(3): 301-5.

92. Zhou Y, Luo X, Li F, et al. Association of Parkinson's disease with six single nucleotide polymorphisms located in four PARK genes in the northern Han Chinese population. *Journal of clinical neuroscience : official journal of the Neurosurgical Society of Australasia* 2012; **19**(7): 1011-5.

93. Yan H, Ma Q, Yang X, Wang Y, Yao Y, Li H. Correlation between LRRK2 gene G2385R polymorphisms and Parkinson's disease. *Molecular medicine reports* 2012; **6**(4): 879-83.

94. Ma Q, An X, Li Z, et al. P268S in NOD2 associates with susceptibility to Parkinson's disease in Chinese population. *Behavioral and brain functions : BBF* 2013; **9**: 19.

95. Fu X, Zheng Y, Hong H, et al. LRRK2 G2385R and LRRK2 R1628P increase risk of Parkinson's disease in a Han Chinese population from Southern Mainland China. *Parkinsonism & related disorders* 2013; **19**(3): 397-8.

96. Yu L, Hu F, Zou X, et al. LRRK2 R1628P contributes to Parkinson's disease susceptibility in Chinese Han populations from mainland China. *Brain research* 2009; **1296**: 113-6.

97. Zhang Z, Burgunder JM, An X, et al. LRRK2 R1628P variant is a risk factor of Parkinson's disease among Han-Chinese from mainland China. *Movement disorders : official journal of the Movement Disorder Society* 2009; **24**(13): 1902-5.

98. Zhao Y, Lin X, Liu W, et al. Association between polymorphisms of PARK16 gene and susceptibility to Parkinson’s disease in Chinese Han population. *Chin J Neurol* 2011; **44**(5): 343-6.

99. Zhu W, Luo X, Adnan A, et al. Association analysis of NUCKS1 and INPP5K polymorphism with Parkinson's disease. *Genes & genetic systems* 2018; **93**(2): 59-64.

100. Lv Z, Tian W, Ma Q, Hao Y, Yang Y. Interactions between four gene polymorphisms and their association with patients with Parkinson's disease in a Chinese Han population. *The International journal of neuroscience* 2017; **127**(12): 1154-60.

101. Liu J, Sun QY, Tang BS, et al. PITX3 gene polymorphism is associated with Parkinson's disease in Chinese population. *Brain research* 2011; **1392**: 116-20.

102. Cai Y, Ding H, Gu Z, Baskys A, Ma J, Chan P. PITX3 polymorphism is not associated with Parkinson's disease in a Chinese population. *Neuroscience letters* 2011; **505**(3): 260-2.

103. Gui Y, Zhao Y, Liu H, Fu J, Xu Z, Hu X. A novel synonymous SNP in PITX3 is associated with Parkinson's disease in Chinese population. *Swiss medical weekly* 2012; **142**: w13521.

104. Deuschl G, Bain P, Brin M. Consensus statement of the Movement Disorder Society on Tremor. Ad Hoc Scientific Committee. *Movement disorders : official journal of the Movement Disorder Society* 1998; **13 Suppl 3**: 2-23.

105. Yu LH, Lin ZF, Liu Y, et al. The transcription factor Pitx3 is a risk modifier for Parkinson's disease in a Chinese Han population. *European journal of neurology* 2011; **18**(5): 778-83.

106. Li K, Tang BS, Yu RL, et al. Association study between two novel single nucleotide polymorphisms and sporadic Parkinson's disease in Chinese Han population. *Neuroscience letters* 2012; **517**(1): 56-9.

107. Chen S, Zhang Y, Chen W, et al. Association study of SCARB2 rs6812193 polymorphism with Parkinson's disease in Han Chinese. *Neuroscience letters* 2012; **516**(1): 21-3.

108. Ding H, Wang F, Ding X, et al. Association study of semaphorin 5A with risk of Parkinson's disease in a Chinese Han population. *Brain research* 2008; **1245**: 126-9.

109. Dong HR, Hua Y, Ding XS. [Correlation study of semaphorin 5a with risk of Parkinson's disease in a Chinese Han population]. *Zhonghua yi xue za zhi* 2009; **89**(23): 1589-92.

110. Xu Y, Cao B, Chen Y, et al. SLC1A2 rs3794087 are associated with susceptibility to Parkinson's disease, but not essential tremor, amyotrophic lateral sclerosis or multiple system atrophy in a Chinese population. *Journal of the neurological sciences* 2016; **365**: 96-100.

111. Cheng Y, Mao CY, Liu YT, et al. Analysis of variant rs3794087 in SLC1A2 and Parkinson's disease in a Chinese Han population: A case-control study and meta-analysis. *Neuroscience letters* 2018; **666**: 165-8.

112. Yuan X, Cao B, Wu Y, et al. Association analysis of SNP rs11868035 in SREBF1 with sporadic Parkinson's disease, sporadic amyotrophic lateral sclerosis and multiple system atrophy in a Chinese population. *Neuroscience letters* 2018; **664**: 128-32.

113. Lou F, Li M, Liu N, Li X, Ren Y, Luo X. The Polymorphism of SREBF1 Gene rs11868035 G/A Is Associated with susceptibility to Parkinson's disease in a Chinese Population. *The International journal of neuroscience* 2018: 1-18.

114. Zhao B, Song W, Chen YP, et al. Association analysis of single-nucleotide polymorphisms of USP24 and USP40 with Parkinson's disease in the Han Chinese population. *European neurology* 2012; **68**(3): 181-4.

115. Wu YR, Chen CM, Chen YC, et al. Ubiquitin specific proteases USP24 and USP40 and ubiquitin thiolesterase UCHL1 polymorphisms have synergic effect on the risk of Parkinson's disease among Taiwanese. *Clinica chimica acta; international journal of clinical chemistry* 2010; **411**(13-14): 955-8.

116. Li DH, Wang J, Mao CJ, et al. [Association of PARK 16 polymorphisms with Parkinson's disease in Han population of Suzhou area]. *Zhonghua yi xue za zhi* 2011; **91**(5): 296-300.

117. Xia H, Luo Q, Li XX, Yang XL. Association between PARK16 gene polymorphisms and susceptibility of Parkinson's disease in a Chinese population. *Genetics and molecular research : GMR* 2015; **14**(2): 2978-85.

118. Long H, Peng G, Zhou C, et al. Study on PARK alpha-synuclein gene mutation and polymorphism of minority patients with Parkinson. *Chin J Ctrl Endem Dis* 2016; **31**(01): 22-4.

119. Liu ZH, Guo JF, Wang YQ, et al. Assessment of RIT2 rs12456492 association with Parkinson's disease in Mainland China. *Neurobiology of aging* 2015; **36**(3): 1600.e9-11.

120. Nie K, Feng SJ, Tang HM, et al. RIT2 polymorphism is associated with Parkinson's disease in a Han Chinese population. *Neurobiology of aging* 2015; **36**(3): 1603.e15-7.

121. Wang JY, Gong MY, Ye YL, et al. The RIT2 and STX1B polymorphisms are associated with Parkinson's disease. *Parkinsonism & related disorders* 2015; **21**(3): 300-2.

122. Lin CH, Chen ML, Yu CY, Wu RM. RIT2 variant is not associated with Parkinson's disease in a Taiwanese population. *Neurobiology of aging* 2013; **34**(9): 2236.e1-3.

123. Wang L, Cheng L, Li NN, Yu WJ, Sun XY, Peng R. Genetic analysis of SLC41A1 in Chinese Parkinson's disease patients. *American journal of medical genetics Part B, Neuropsychiatric genetics : the official publication of the International Society of Psychiatric Genetics* 2015; **168**(8): 706-11.

124. Yan Y, Tian J, Mo X, et al. Genetic variants in the RAB7L1 and SLC41A1 genes of the PARK16 locus in Chinese Parkinson's disease patients. *The International journal of neuroscience* 2011; **121**(11): 632-6.

125. Wang YQ, Tang BS, Yu RL, et al. Association analysis of STK39, MCCC1/LAMP3 and sporadic PD in the Chinese Han population. *Neuroscience letters* 2014; **566**: 206-9.

126. Shao M, Liu Z, Tao E, Chen B. [Polymorphism of MAO-B gene and NAD(P)H: quinone oxidoreductase gene in Parkinson's disease]. *Zhonghua yi xue yi chuan xue za zhi = Zhonghua yixue yichuanxue zazhi = Chinese journal of medical genetics* 2001; **18**(2): 122-4.

127. Jiang XH, Yang H, Yang JF, Wang HT, Xu QY, Chen B. [A study on the relationship between polymorphism of human NAD(P)H: quinone oxidoreductase and Parkinson's disease in Chinese]. *Zhonghua yi xue yi chuan xue za zhi = Zhonghua yixue yichuanxue zazhi = Chinese journal of medical genetics* 2004; **21**(2): 120-3.

128. Fang J, Yi K. Analysis of LRRK2, SNCA, and ITGA8 Gene Variants with Sporadic Parkinson's Disease Susceptibility in Chinese Han Population. 2016; **2016**: 3474751.

129. Chen Y, Wei QQ, Ou R, et al. Genetic Variants of SNCA Are Associated with Susceptibility to Parkinson's Disease but Not Amyotrophic Lateral Sclerosis or Multiple System Atrophy in a Chinese Population. *PloS one* 2015; **10**(7): e0133776.

130. Pan F, Ding H, Dong H, et al. Association of polymorphism in rs2736990 of the alpha-synuclein gene with Parkinson's disease in a Chinese population. *Neurology India* 2013; **61**(4): 360-4.

131. Gan R, Zhang Y, Nie K, Wang Y. Relation of synuclein gene polymorphisms with Parkinson’s disease and its cognitive impairment. *Chin J Geriatr Heart Brain Vessel Dis,* 2012; **8**(14).

132. Guo XY, Chen YP, Song W, et al. SNCA variants rs2736990 and rs356220 as risk factors for Parkinson's disease but not for amyotrophic lateral sclerosis and multiple system atrophy in a Chinese population. *Neurobiology of aging* 2014; **35**(12): 2882.e1-.e6.

133. Hu FY, Hu WB, Liu L, et al. Lack of replication of a previously reported association between polymorphism in the 3'UTR of the alpha-synuclein gene and Parkinson's disease in Chinese subjects. *Neuroscience letters* 2010; **479**(1): 31-3.

134. Li NN, Mao XY, Chang XL, et al. SNCA rs356219 variant increases risk of sporadic Parkinson's disease in ethnic Chinese. *American journal of medical genetics Part B, Neuropsychiatric genetics : the official publication of the International Society of Psychiatric Genetics* 2013; **162b**(5): 452-6.

135. Pan F, Dong H, Ding H, et al. SNP rs356219 of the alpha-synuclein (SNCA) gene is associated with Parkinson's disease in a Chinese Han population. *Parkinsonism & related disorders* 2012; **18**(5): 632-4.

136. Jian CD, Huang JM, Meng LQ, et al. SNCA rs3822086 C>T Polymorphism Increases the Susceptibility to Parkinson's Disease in a Chinese Han Population. *Genetic testing and molecular biomarkers* 2015; **19**(9): 481-7.

137. Brooks DJJP, disorders r. Parkinson's disease: diagnosis. 2012; **18**: S31-S3.

138. Wang J, Zhao CY, Si YM, Liu ZL, Chen B, Yu L. ACT and UCH-L1 polymorphisms in Parkinson's disease and age of onset. *Movement disorders : official journal of the Movement Disorder Society* 2002; **17**(4): 767-71.

139. Wang L, Guo JF, Nie LL, et al. Case-control study of the UCH-L1 S18Y variant in sporadic Parkinson's disease in the Chinese population. *Journal of clinical neuroscience : official journal of the Neurosurgical Society of Australasia* 2011; **18**(4): 541-4.

140. Zhang ZJ, Burgunder JM, An XK, et al. Lack of evidence for association of a UCH-L1 S18Y polymorphism with Parkinson's disease in a Han-Chinese population. *Neuroscience letters* 2008; **442**(3): 200-2.

141. Hao YX, Zhang J, Fang CP, Zhang Q, Zhang JR, Shen Q. [Association of two polymorphisms in ubiquitin carboxy-terminal hydrolase-L1 gene with Parkinson's disease in Shanghai]. *Zhonghua yi xue yi chuan xue za zhi = Zhonghua yixue yichuanxue zazhi = Chinese journal of medical genetics* 2008; **25**(3): 272-5.

142. Lv Z, Tang B, Sun Q, Yan X, Guo J. Association study between vitamin d receptor gene polymorphisms and patients with Parkinson disease in Chinese Han population. *The International journal of neuroscience* 2013; **123**(1): 60-4.

143. Lin CH, Chen KH, Chen ML, Lin HI, Wu RM. Vitamin D receptor genetic variants and Parkinson's disease in a Taiwanese population. *Neurobiology of aging* 2014; **35**(5): 1212.e11-3.

144. Liu HX, Han X, Zheng XP, Li YS, Xie AM. [Association of vitamin D receptor gene polymorphisms with Parkinson disease]. *Zhonghua yi xue yi chuan xue za zhi = Zhonghua yixue yichuanxue zazhi = Chinese journal of medical genetics* 2013; **30**(1): 13-6.
